# Supplementary material for: Catalytic asymmetric C–Si bond activation via torsional strain-promoted Rh-catalyzed aryl-Narasaka acylation
Source: Nat Commun. 2020 Sep 7;11:4449. doi: 10.1038/s41467-020-18273-3 (PMC7477585; doi:10.1038/s41467-020-18273-3)
Supplement: Supplementary file 1 — Supplementary Information [file 41467_2020_18273_MOESM1_ESM.pdf]

Supplementary Information

Catalytic Asymmetric C-Si Bond Activation via Torsional Strain-Promoted Rh-Catalyzed Aryl-Narasaka Acylation

Feng *et al.*

Supplementary Methods

General Information

Nuclear magnetic resonances were recorded on Bruker-400 MHz and Bruker-500 MHz instruments. Reference values for residual solvents were taken as δ = 7.26 ppm ($CDCl_3$), 2.50 ppm (DMSO-d_6) for ^1H NMR; δ = 77.00 ppm ($CDCl_3$), δ = 39.00 ppm (DMSO-d_6) for ^{13}C NMR. All reactions were performed under an inert atmosphere of dry nitrogen in flame-dried glassware, unless otherwise stated. 1,4-Dioxane and tetrahydrofuran were distilled over sodium in the presence of benzophenone under an atmosphere of nitrogen. Toluene and dichloromethane were distilled over calcium hydride under an atmosphere of nitrogen.

Typical procedure for the preparation of the L7 (Typical Procedure A)

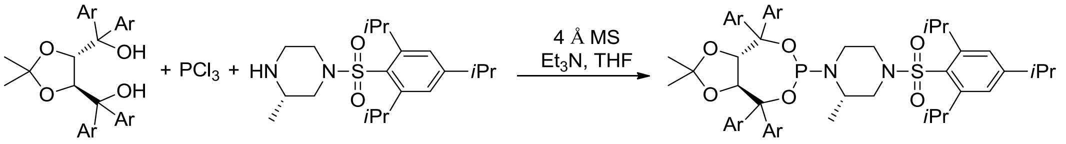

L7 : Ar = 4-PhC_6H_4

The 4-PhC_6H_4-TADDOL (2.313 g, 3.00 mmol, 1.0 equiv) was added to a flame-dried round bottom flask charged with 4 Å molecular sieves, followed by the addition of Et_3N (4.50 mL, 32.37 mmol, 11.0 equiv) and THF (30 mL). After the mixture being cooled to 0 ℃, phosphorus trichloride (0.31 mL, 3.60 mmol, 1.2 equiv) was added dropwise and the mixture was stirred at room temperature for 45 minutes. A mixture of (*S*)-3-methyl-1-((2,4,6-triisopropylphenyl)sulfonyl)piperazine (3.300 g, 9.00 mmol, 3.0 equiv) and Et_3N (1.80 mL, 12.95 mmol, 4.3 equiv) in THF (10 mL) was added slowly at 0 ℃ and the mixture was allowed to warm to room temperature and stirred overnight. Ethyl ether (10 mL) was added to the reaction flask and the mixture was filtered through a pad of Celite. The filtrate was concentrated in vacuum and the residue was purified by flash column chromatography (PE/EtOAc = 95:5) on silica gel which was pre-treated with Et_3N to afford **L7** (2.640 g, 76%). $[\alpha]_D^{20}$ + 54.19 (c 1.13, CH_2Cl_2). **^{1}H NMR** (500 MHz, $CDCl_3$) δ 7.89 (d, *J* = 8.5 Hz, 2H), 7.74 (d, *J* = 8.4 Hz, 2H), 7.67 – 7.62 (m, 4H), 7.62 – 7.61 (m, 4H), 7.61 – 7.59 (m, 4H), 7.59 – 7.56 (m, 4H), 7.56 – 7.49 (m, 4H), 7.48 – 7.45 (m, 4H), 7.45 – 7.41 (m, 4H), 7.39 – 7.32 (m, 4H), 7.23 – 7.18 (m, 2H), 5.29 (dd, *J* = 8.6, 3.4 Hz, 1H), 4.89 (d, *J* = 8.4 Hz, 1H), 4.23 (hept, *J* = 6.7 Hz, 2H), 4.04 – 3.89 (m, 1H), 3.63 – 3.53 (m, 1H), 3.53 – 3.44 (m, 1H), 3.40 (dd, *J* = 11.3, 3.1 Hz, 1H), 3.37 – 3.30 (m, 1H), 3.18 (dd, *J* = 11.5, 3.4 Hz, 1H), 3.14 – 3.06 (m, 1H), 2.93 (hept, *J* = 7.0 Hz, 1H), 1.42 (s, 3H), 1.41 (d, *J* = 4.7, 3H), 1.32 – 1.27 (m, 18H), 0.44 (s, 3H). **^{13}C NMR** (126 MHz, $CDCl_3$) δ 153.4, 151.9, 145.8, 145.28, 145.27, 141.0, 140.7, 140.63, 140.62, 140.5, 140.4, 140.3, 140.04, 140.00, 139.9, 129.5, 129.3, 129.14, 129.10, 128.73, 128.71, 128.70, 127.5, 127.4, 127.3, 127.2, 127.04, 127.00, 126.9 126.5, 126.3, 125.9, 123.9, 111.9,

82.5 (d, J = 2.8 Hz), 82.3 (d, J = 16.8 Hz), 82.0, 81.4 (d, J = 7.3 Hz), 49.7 (d, J = 3.7 Hz), 48.1 (d, J = 19.4 Hz), 46.1 (d, J = 1.6 Hz), 38.9 (d, J = 10.3 Hz), 34.2, 29.5, 27.6, 25.5, 25.1, 24.8, 23.54, 23.53, 17.6 (d, J = 7.7 Hz). **^{31}P NMR** (202 MHz, $CDCl_3$) δ 137.5. **HRMS (ESI)** calcd for $C_{75}H_{78}N_2O_6PS$ $[M+H]^+$ 1165.5318, found 1165.5313.

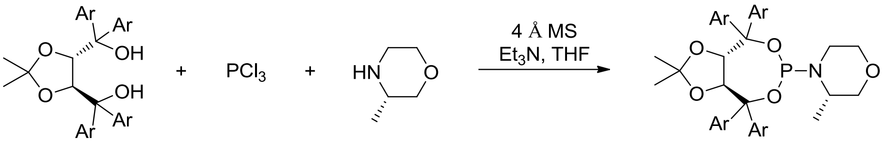

L2 : Ar = 4-FC_6H_4

L2 was prepared following the **Typical Procedure A**

The reaction of 4-FC_6H_4-TADDOL (0.538 g, 1.00 mmol), phosphorus trichloride (0.10 mL, 1.20 mmol), *(S)*-3-methylmorpholine (0.304 g, 3.00 mmol) and 4 Å molecular sieves afforded **L2** (0.374 g, 56%). $[\alpha]_D^{20}$ + 97.56 (c 1.18, CH_2Cl_2). **^{1}H NMR** (400 MHz, $CDCl_3$) δ 7.72 – 7.64 (m, 2H), 7.58 – 7.51 (m, 2H), 7.41 – 7.35 (m, 2H), 7.34 – 7.28 (m, 2H), 7.06 – 7.01(m, 2H), 7.01 – 6.99 (m, 2H), 6.99 – 6.97 (m, 2H), 6.97 – 6.91 (m, 2H), 5.01 (dd, J = 8.6, 3.4 Hz, 1H), 4.60 (d, J = 8.4 Hz, 1H), 3.81 (dt, J = 10.8, 3.2 Hz, 1H), 3.68 – 3.58 (m, 2H), 3.58 – 3.49 (m, 2H), 3.49 – 3.38 (m, 1H), 3.32 – 3.22 (m, 1H), 1.33 (d, J = 6.4 Hz, 3H), 1.29 (s, 3H), 0.37 (s, 3H). **^{13}C NMR** (100 MHz, $CDCl_3$) δ 162.1 (d, J = 245.4 Hz), 162.0 (d, J = 245.6 Hz), 161.9 (d, J = 245.4 Hz), 142.5 (d, J = 3.4 Hz), 142.0, 137.64, 137.63, 137.61, 137.60, 137.04, 137.03, 130.53, 130.51, 130.47, 130.45, 130.43, 130.39, 128.8, 128.7, 128.6, 115.1 (d, J = 21.2 Hz), 114.7 (d, J = 21.1 Hz), 114.5 (d, J = 20.8 Hz), 114.1 (d, J = 21.2 Hz), 111.9, 82.2 (d, J = 3.7 Hz), 82.0 (d, J = 21.1 Hz), 81.5, 80.6 (d, J = 9.2 Hz), 72.6 (d, J = 4.8 Hz), 68.2 (d, J = 3.3 Hz), 48.5 (d, J = 21.9 Hz), 39.5 (d, J = 12.0 Hz), 27.50, 25.48, 16.7 (d, J = 9.5 Hz). **^{31}P NMR** (162 MHz, $CDCl_3$) δ 137.1. **^{19}F NMR** (376 MHz, $CDCl_3$) δ -114.4, -114.7, -115.2, -115.3. **HRMS (ESI)** calcd for $C_{36}H_{35}F_4NO_5P$ $[M+H]^+$ 668.2189, found 668.2190.

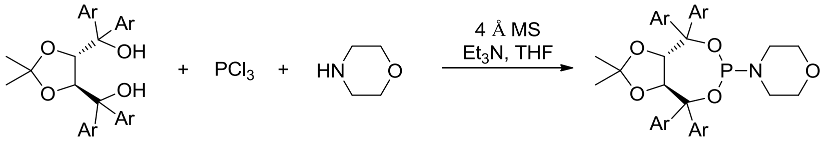

L3 : Ar = 4-PhC_6H_4

L3 was prepared following the **Typical Procedure A**

The reaction of 4-PhC_6H_4-TADDOL (0.280 g, 0.60 mmol), phosphorus trichloride (63 μL, 0.72 mmol), morpholine (0.375 g, 3.00 mmol) and 4 Å molecular sieves afforded **L3** (0.194 g, 52%). $[\alpha]_D^{20}$ + 91.18 (c 0.99, CH_2Cl_2). **^{1}H NMR** (400 MHz, $CDCl_3$) δ 7.90 (d, J = 8.4 Hz, 2H), 7.76 (d, J = 8.0 Hz, 2H), 7.65 – 7.61 (m, 4H), 7.61 – 7.59 (m, 8H), 7.59 – 7.56 (m, 4H), 7.56 – 7.51 (m, 4H), 7.47 – 7.44 (m, 2H), 7.44 – 7.42 (m, 4H), 7.42 – 7.40 (m, 2H), 7.34 (t, J = 7.2 Hz, 4H), 5.29 (dd, J = 8.6, 3.4 Hz, 1H), 4.90 (d, J = 8.4 Hz, 1H), 3.90 – 3.67 (m, 4H), 3.52 – 3.39 (m, 2H), 3.39 – 3.26 (m, 2H), 1.41 (s, 3H), 0.42 (s, 3H). **^{13}C NMR** (100 MHz, $CDCl_3$) δ 145.7, 145.2, 140.9, 140.69, 140.67, 140.63, 140.55, 140.4, 140.02, 139.96, 129.4, 129.14, 129.09, 128.7, 127.5, 127.4, 127.3, 127.08, 127.05, 127.01, 126.96, 126.6, 126.3, 125.9, 111.9, 82.6 (d, J = 2.8 Hz), 82.4 (d, J = 19.9 Hz), 81.9, 81.3 (d, J = 8.0 Hz), 67.9 (d, J = 5.8 Hz), 44.2 (d, J = 17.1 Hz), 27.7, 25.5. **^{31}P NMR** (162 MHz, $CDCl_3$) δ 138.0. **HRMS (ESI)** calcd for $C_{59}H_{53}NO_5P$ $[M+H]^+$ 886.3661, found 886.3671.

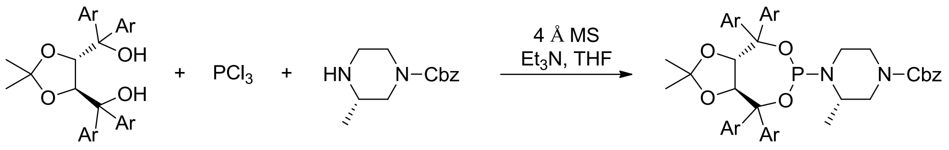

L4 : Ar = 4-PhC_6H_4

L4 was prepared following the **Typical Procedure A**

The reaction of 4-PhC_6H_4-TADDOL (0.771 g, 1.00 mmol), phosphorus trichloride (0.10 mL, 1.20 mmol), benzyl *(S)*-3-methylpiperazine-1-carboxylate (0.70 mL, 3.00 mmol) and 4 Å molecular sieves afforded **L4** (0.671 g, 65%). $[\alpha]_D^{20}$ + 70.31 (c 1.03, CH_2Cl_2). **^{1}H NMR** (500 MHz, $CDCl_3$) δ 7.92 (d, *J* = 8.0 Hz, 2H), 7.75 (d, *J* = 8.0 Hz, 2H), 7.66 – 7.65 (m, 1H), 7.65 – 7.63 (m, 2H), 7.63 – 7.61 (m, 4H), 7.61 – 7.60 (m, 4H), 7.60 – 7.58 (m, 4H), 7.58 – 7.56 (m, 2H), 7.56 – 7.54 (m, 2H), 7.48 – 7.45 (m, 4H), 7.45 – 7.42 (m, 4H), 7.42 – 7.40 (m, 2H), 7.40 – 7.38 (m, 2H), 7.38 – 7.36 (m, 2H), 7.36 – 7.35 (m, 2H), 7.35 – 7.32 (m, 2H), 5.31 (dd, *J* = 8.5, 3.0 Hz, 1H), 5.26 – 5.09 (m, 2H), 4.89 (d, *J* = 8.5 Hz, 1H), 4.21 – 3.86 (m, 2H), 3.80 (d, *J* = 13.0 Hz, 1H), 3.64 – 3.40 (m, 2H), 3.38 – 3.13 (m, 2H), 1.45 (s, 3H), 1.36 (s, 3H), 0.43 (s, 3H). **^{13}C NMR** (126 MHz, $CDCl_3$) δ 155.8, 145.9, 145.4, 145.3, 141.0, 140.68, 140.65, 140.6, 140.5, 140.3, 140.02, 140.00, 139.9, 136.7, 129.4, 129.14, 129.10, 128.73, 128.70, 128.5, 128.0, 127.9, 127.5, 127.4, 127.28, 127.26, 127.2, 127.1, 127.04, 127.00, 126.99, 126.9, 126.5, 126.3, 125.9, 111.8, 82.7 (d, *J* = 3.0 Hz), 82.4 (d, *J* = 21.4 Hz), 81.8, 81.3 (d, *J* = 8.8 Hz), 67.2, 50.2, 49.8, 48.3, 47.8, 45.5, 45.2, 38.8 (d, *J* = 22.2 Hz), 27.7, 25.4, 17.0. **^{31}P NMR** (202 MHz, $CDCl_3$) δ 138.15. **HRMS (ESI)** calcd for $C_{68}H_{62}N_2O_6P$ $[M+H]^+$ 1033.4345, found 1033.4331.

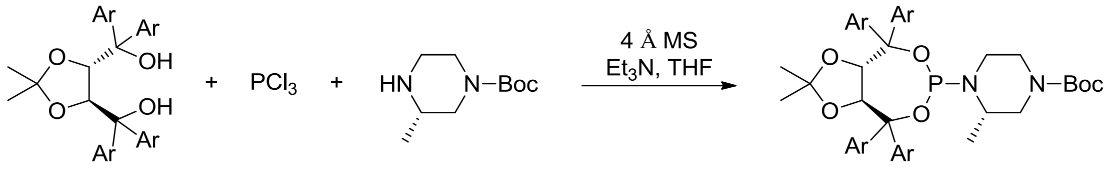

L5 : Ar = 4-PhC_6H_4

L5 was prepared following the **Typical Procedure A**

The reaction of 4-PhC_6H_4-TADDOL (0.771 g, 1.00 mmol), phosphorus trichloride (0.10 mL, 1.20 mmol), benzyl *tert*-butyl (*S*)-3-methylpiperazine-1-carboxylate (0.601 g, 3.00 mmol) and 4 Å molecular sieves afforded **L5** (0.383 g, 38%). $[\alpha]_D^{20}$ + 68.81 (c 0.89, CH_2Cl_2). **^{1}H NMR** (500 MHz, $CDCl_3$) δ 8.00 (d, *J* = 8.0 Hz, 2H), 7.82 (d, *J* = 8.0 Hz, 2H), 7.71 – 7.68 (m, 4H), 7.68 – 7.67 (m, 4H), 7.67 – 7.66 (m, 2H), 7.66 – 7.65 (m, 4H), 7.65 – 7.63 (m, 2H), 7.63 – 7.61 (m, 4H), 7.52 – 7.49 (m, 4H), 7.48 – 7.45 (m, 4H), 7.42 – 7.39 (m, 2H), 7.39 – 7.36 (m, 2H), 5.39 (d, *J* = 8.0 Hz, 1H), 4.95 (d, *J* = 8.0 Hz, 1H), 4.21 – 3.89 (m, 2H), 3.80 (d, *J* = 12.5 Hz, 1H), 3.67 – 3.45 (m, 2H), 3.34 – 3.11 (m, 2H), 1.57 (s, 9H), 1.52 (s, 3H), 1.42 (d, *J* = 7.0 Hz, 3H), 0.49 (s, 3H). **^{13}C NMR** (126 MHz, $CDCl_3$) δ 155.2, 145.9, 145.41, 145.39, 141.1, 140.7, 140.64, 140.59, 140.54, 140.49, 140.3, 140.0, 139.9, 129.4, 129.12, 129.08, 128.70, 128.68, 127.5, 127.4, 127.3, 127.23, 127.19, 127.03, 127.01, 126.97, 126.96, 126.9, 126.5, 126.3, 125.9, 111.7, 82.7 (d, *J* = 3.3 Hz), 82.4 (d, *J* = 21.4 Hz), 81.7, 81.3 (d, *J* = 8.7 Hz), 79.6, 50.4, 49.2, 48.2, 45.7, 44.5, 39.0, 28.4, 27.7, 25.4, 17.0. **^{31}P NMR** (202 MHz, $CDCl_3$) δ 138.19. **HRMS (ESI)** calcd for $C_{65}H_{64}N_2O_6P$ $[M+H]^+$ 999.4502, found 999.4504.

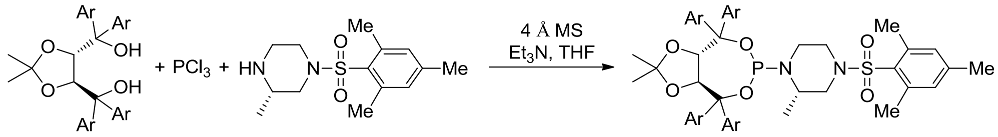

L6 : Ar = 4-PhC_6H_4

L6 was prepared following the **Typical Procedure A**

The reaction of 4-PhC_6H_4-TADDOL (0.385 g, 0.50 mmol), phosphorus trichloride (50 μL, 0.60 mmol), (*S*)-1-(mesitylsulfonyl)-3-methylpiperazine (0.424 g, 1.50 mmol) and 4 Å molecular sieves afforded **L6** (0.336 g, 62%). $[\alpha]_D^{20}$ + 97.70 (c 1.00, CH_2Cl_2). **^{1}H NMR** (400 MHz, $CDCl_3$) δ 7.87 (d, *J* = 8.4 Hz, 2H), 7.71 (d, *J* = 8.4 Hz, 2H), 7.65 – 7.63 (m, 2H), 7.63 – 7.62 (m, 2H), 7.62 – 7.61 (m, 2H), 7.61 – 7.60 (m, 2H), 7.60 – 7.59 (m, 2H), 7.59 – 7.57 (m, 4H), 7.56 – 7.53 (m, 2H), 7.53 – 7.50 (m, 2H), 7.50 – 7.46 (m, 2H), 7.46 – 7.44 (m, 4H), 7.44 – 7.43 (m, 2H), 7.43 – 7.40 (m, 2H), 7.39 – 7.36 (m, 2H), 7.36 – 7.31 (m, 2H), 6.96 (s, 2H), 5.27 (dd, *J* = 8.4, 3.6 Hz, 1H), 4.87 (d, *J* = 8.4 Hz, 1H), 3.99 – 3.84 (m, 1H), 3.65 – 3.54 (m, 1H), 3.53 – 3.41 (m, 2H), 3.25 – 3.16 (m, 1H), 3.16 – 3.00 (m, 2H), 2.67 (s, 6H), 2.29 (s, 3H), 1.41 (s, 3H), 1.31 (d, *J* = 6.8 Hz, 3H), 0.43 (s, 3H). **^{13}C NMR** (100 MHz, $CDCl_3$) δ 145.8, 145.28, 145.26, 142.7, 140.96, 140.95, 140.7, 140.63, 140.60, 140.51, 140.49, 140.40, 140.38, 140.3, 140.03, 140.00, 139.94, 131.93, 131.0, 129.3, 129.14, 129.09, 128.73, 128.70, 127.5, 127.4, 127.3, 127.2, 127.1, 127.0, 126.9, 126.6, 126.3, 125.9 111.9, 82.5 (d, *J* = 3.9 Hz), 82.3 (d, *J* = 21.3 Hz), 82.0, 81.3 (d, *J* = 8.8 Hz), 50.4 (d, *J* = 4.1 Hz), 47.8 (d, *J* = 23.2 Hz), 45.4 (d, *J* = 2.9 Hz), 38.8 (d, *J* = 14.3 Hz), 27.6, 25.5, 23.0, 20.9, 17.4 (d, *J* = 8.4 Hz). **^{31}P NMR** (162 MHz, $CDCl_3$) δ 137.7. **HRMS (ESI)** calcd for $C_{69}H_{66}N_2O_6PS$ $[M+H]^+$ 1081.4379, found 1081.4391.

Typical procedure for the preparation of diiodides [1](Typical Procedure B)

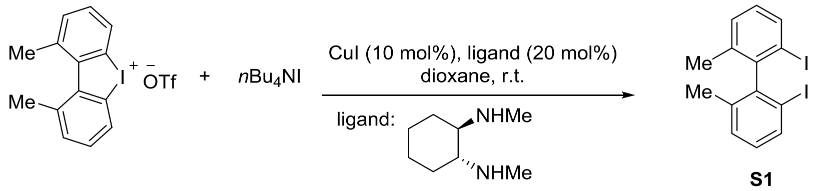

S1

A mixture of dimethyl cyclic diaryliodonium[2] (2.980 g, 6.50 mmol, 1.0 equiv), tetrabutylammonium iodide (4.800 g, 13.00 mmol, 2.0 equiv), CuI (0.124 g, 0.65 mmol, 10 mol%), *trans*-N,N'- dimethylcyclohexane-1,2-diamine (0.185 g, 1.30 mmol, 20 mol%) in dioxane (15 mL) was stirred at room temperature for 24 hours. After complete consumption of starting material, the mixture was filtered through Celite and the filtrate was concentrated in vacuum and purified by flash column chromatography (PE/EtOAc = 95:5) on silica gel to afford **S1** (2.590 g, 92%). **^{1}H NMR** (500 MHz, $CDCl_3$) δ 7.81 (d, *J* = 7.9 Hz, 2H), 7.28 (d, *J* = 7.6 Hz, 2H), 7.00 (t, *J* = 7.8 Hz, 2H), 2.01 (s, 6H). **^{13}C NMR** (126 MHz, $CDCl_3$) δ 147.4, 137.5, 136.7, 130.0, 129.4, 100.6, 21.4. **HRMS (ESI)** calcd for $C_{14}H_{12}I_2Na$ $[M+Na]^+$ 456.8926, found 456.8897.

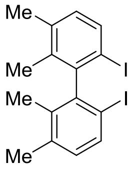

S2

S2 was prepared following the **Typical Procedure B**

The reaction of tetramethyl cyclic diaryliodonium[2] (4.380 g, 9.05 mmol, 1.0 equiv) and tetrabutylammonium iodide (6.690 g, 18.10 mmol, 2.0 equiv) afforded **S2** (3.747 g, 90%). **^{1}H NMR** (400 MHz, $CDCl_3$) δ 7.69 (d, *J* = 8.0 Hz, 2H), 6.91 (d, *J* = 8.0 Hz, 2H), 2.29 (s, 6H), 1.92 (s, 6H). **^{13}C NMR** (100 MHz, $CDCl_3$) δ 147.8, 137.1, 136.3, 136.0, 130.8, 97.5, 20.3, 17.8. **HRMS (ESI)** calcd for $C_{16}H_{16}I_2Na$ $[M+Na]^+$ 484.9239, found 484.9247.

Typical procedure for the preparation of anhydrides[3] (Typical Procedure C)

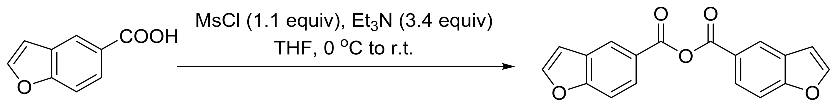

S3

To a flask charged with benzofuran-5-carboxylic acid (0.972 g, 6.00 mmol, 2.0 equiv) and MsCl (0.26 mL, 3.30 mmol, 1.1 equiv) in THF (18 mL) at 0 °C was added a solution of Et_3N (1.40 mL, 10.26 mmol, 3.4 equiv) in THF (36 mL) dropwise. The mixture was warmed to room temperature and stirred for 1 hour. Then the mixture was concentrated under vacuum, diluted with ethyl acetate and $NaHCO_3$(aq.) and extracted with ethyl acetate for three times, and the combined organic layer was washed with brine, dried over Na_2SO_4, filtered and concentrated to afford the crude **S3** (0.933 g, 61%) without further purification. **^{1}H NMR** (400 MHz, $CDCl_3$) δ 8.49 (s, 2H), 8.16 (d, *J* = 8.4 Hz, 2H), 7.80 – 7.71 (m, 2H), 7.62 (d, *J* = 8.8 Hz, 2H), 6.94 – 6.84 (m, 2H). **^{13}C NMR** (100 MHz, $CDCl_3$) δ 162.7, 158.2, 146.8, 127.9, 127.0, 125.0, 123.8, 111.9, 107.2. **HRMS (ESI)** calcd for $C_{18}H_{10}O_5Na$ $[M+Na]^+$ 329.0426, found 329.0422.

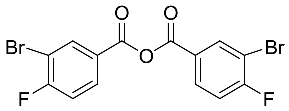

S4

S4 was prepared following the **Typical Procedure C**

The reaction of 3-bromo-4-fluorobenzoic acid (2.190 g, 10.00 mmol, 2.0 equiv) with MsCl (0.43 mL, 5.50 mmol, 1.1 equiv) afforded **S4** (0.735 g, 35%). **^{1}H NMR** (400 MHz, $CDCl_3$) δ 8.38 – 8.32 (m, 2H), 8.14 – 8.07 (m, 2H), 7.29 (d, *J* = 8.0 Hz, 2H). **^{13}C NMR** (100 MHz, $CDCl_3$) δ 163.2 ($^1J_{C\text{-}F}$ = 259 Hz), 159.9, 136.4 ($^4J_{C\text{-}F}$ = 2.1 Hz), 131.9 ($^3J_{C\text{-}F}$ = 9.0 Hz), 126.0 ($^3J_{C\text{-}F}$ = 3.5 Hz), 117.3 ($^2J_{C\text{-}F}$ = 23.3 Hz), 110.2 ($^2J_{C\text{-}F}$ = 22.1 Hz). **^{19}F NMR** (376 MHz, $CDCl_3$) δ -95.9. **HRMS (ESI)** calcd for $C_{14}H_6Br_2F_2O_3Na$ $[M+Na]^+$ 440.8549, found 440.8555.

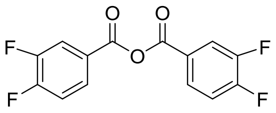

S5

S5 was prepared following the **Typical Procedure C**

The reaction of 3,4-difluorobenzoic acid (1.580 g, 10.00 mmol, 2.0 equiv) with MsCl (0.43 mL, 5.50 mmol, 1.1 equiv) afforded **S5** (0.960 g, 64%). **^{1}H NMR** (400 MHz, $CDCl_3$) δ 8.01 – 7.95 (m, 2H), 7.95 – 7.85 (m, 2H), 7.40 – 7.29 (m, 2H). **^{13}C** NMR (100 MHz, $CDCl_3$) δ 159.9, 154.8 (dd, $J_{C\text{-}F}$ = 261, 12.7 Hz), 150.4 (dd, $J_{C\text{-}F}$ = 253, 13.2 Hz), 127.8 (dd, $J_{C\text{-}F}$ = 7.8, 3.7 Hz), 125.4 (dd, $J_{C\text{-}F}$ = 5.6, 3.6 Hz), 119.9 (dd, $J_{C\text{-}F}$ = 19.0, 2.0 Hz), 118.2 (d, $^{2}J_{C\text{-}F}$ = 18.3 Hz). **^{19}F NMR** (376 MHz, $CDCl_3$) δ - 126.0, - 134.7. **HRMS (ESI)** calcd for $C_{14}H_6F_4O_3Na$ $[M+Na]^+$ 321.0151, found 321.0150.

Typical procedure for the preparation of silafluorene (Typical Procedure D)

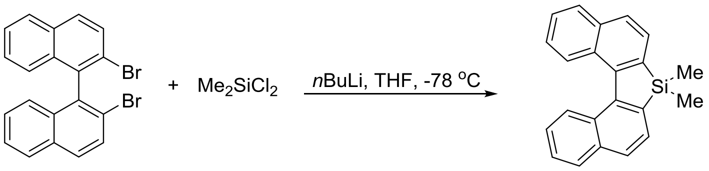

4a

Under nitrogen atmosphere, to a mixture of 2,2'-dibromo-1,1'-binaphthalene (2.060 g, 5.00 mmol, 1.0 equiv) in THF (50 mL) was added *n*BuLi (2.4 M in hexanes, 4.8 mL, 11.50 mmol, 2.3 equiv) dropwise at -78 °C. After the mixture was stirred under the same temperature for 2 hours, a solution of dichlorodimethylsilane (0.73 mL, 7.50 mmol, 1.50 equiv) in THF (5 mL) was added dropwise. The resulting mixture was stirred for additional 12 hours at -78 °C before it was allowed to warm to room temperature. The mixture was quenched with water (10 mL) and extracted with ethyl acetate (15 mL*3). The combined organic layer was washed with brine and dried with Na_2SO_4, concentrated and purified by flash column chromatography (PE/EtOAc = 99:1) on silica gel which was pretreated by Et_3N to give the desired product **4a** (1.301 g, 84%). **^{1}H NMR** (400 MHz, $CDCl_3$) δ 8.01 (d, *J* = 8.8 Hz, 2H), 7.95 (d, *J* = 8.0 Hz, 2H), 7.90 (d, *J* = 7.6 Hz, 2H), 7.82 (d, *J* = 8.0 Hz, 2H), 7.52 (t, *J* = 7.4 Hz, 2H), 7.43 – 7.34 (m, 2H), 0.48 (s, 6H). **^{13}C NMR** (100 MHz, $CDCl_3$) δ 147.1, 139.9, 135.7, 129.7, 128.5, 128.3, 127.9, 127.8, 125.7, 124.3, -3.32. **HRMS (ESI)** calcd for $C_{22}H_{19}Si$ $[M+H]^+$ 311.1256, found 311.1256.

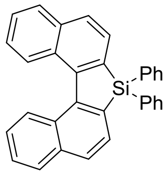

4b

4b was prepared following the **Typical Procedure D**

The reaction of 2,2'-dibromo-1,1'-binaphthalene (4.121 g, 10.00 mmol, 1.0 equiv), *n*BuLi (2.4

M in hexanes, 9.6 mL, 23.00 mmol, 2.3 equiv) and dichlorodiphenylsilane (3.15 mL, 15.00 mmol, 1.5 equiv) afforded **4b** (3.201 g, 73%). **^{1}H NMR** (400 MHz, $CDCl_3$) δ 8.04 (d, *J* = 8.8 Hz, 2H), 7.99 – 7.89 (m, 6H), 7.68 (d, *J* = 7.2 Hz, 4H), 7.54 (t, *J* = 7.4 Hz, 2H), 7.45 – 7.38 (m, 4H), 7.34 (t, *J* = 7.4 Hz, 4H). **^{13}C NMR** (100 MHz, $CDCl_3$) δ 148.5, 136.8, 136.0, 135.6, 132.4, 130.2, 129.8, 129.3, 128.32, 128.26, 128.2, 128.0, 126.1, 124.4. **HRMS (ESI)** calcd for $C_{32}H_{23}Si$ $[M+H]^+$ 435.1569, found 435.1563.

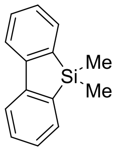

4c

4c (known compound)[4] was prepared following the **Typical Procedure D**

The reaction of 2,2'-dibromo-1,1'-biphenyl (1.560 g, 5.00 mmol, 1.0 equiv), *n*BuLi (2.4 M in hexanes, 4.4 mL, 10.50 mmol, 2.1 equiv) and dichlorodimethylsilane (0.97 mL, 10.00 mmol, 2.0 equiv) afforded **4c** (0.879 g, 84%). **^{1}H NMR** (500 MHz, $CDCl_3$) δ 7.83 (d, *J* = 7.5 Hz, 2H), 7.64 (d, *J* = 7.0 Hz, 2H), 7.44 (t, *J* = 7.5 Hz, 2H), 7.31 – 7.27 (m, 2H), 0.43 (s, 6H). **^{13}C NMR** (126 MHz, $CDCl_3$) δ 148.4, 137.4, 134.6, 134.4, 133.3, 130.5, 129.8, 128.0, 127.6, 120.9, -5.1.

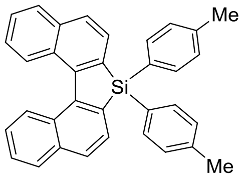

4d

4d was prepared following the **Typical Procedure D**

The reaction of 2,2'-dibromo-1,1'-binaphthalene (0.680 g, 1.65 mmol, 1.1 equiv), *n*BuLi (2.4 M in hexanes, 1.6 mL, 3.75 mmol, 2.5 equiv) and dimethoxydi-*p*-tolylsilane (0.408 g, 1.50 mmol, 1.0 equiv) afforded **4d** (0.434 g, 63%). **^{1}H NMR** (400 MHz, $CDCl_3$) δ 8.03 (d, *J* = 8.4 Hz, 2H), 7.93 (t, *J* = 7.2 Hz, 4H), 7.90 (d, *J* = 7.6 Hz, 2H), 7.57 (d, *J* = 8.0 Hz, 4H), 7.53 (t, *J* = 7.6 Hz, 2H), 7.40 (t, *J* = 7.6 Hz, 2H), 7.17 (d, *J* = 7.6 Hz, 4H), 2.34 (s, 6H). **^{13}C NMR** (100 MHz, $CDCl_3$) δ 148.4, 140.3, 137.3, 136.0, 135.6, 129.8, 129.4, 129.0, 128.8, 128.3, 128.2, 128.0, 126.0, 124.3, 21.6. **HRMS (ESI)** calcd for $C_{34}H_{27}Si$ $[M+H]^+$ 463.1882, found 463.1880.

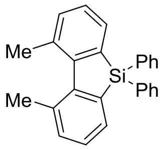

4e

4e was prepared following the **Typical Procedure D**

The reaction of 2,2'-diiodo-6,6'-dimethyl-1,1'-biphenyl (0.651 g, 1.50 mmol, 1.0 equiv), *n*BuLi (2.4 M in hexanes, 1.40 mL, 3.45 mmol, 2.3 equiv) and dichlorodiphenylsilane (0.48 mL, 2.25 mmol, 1.5 equiv) afforded **4e** (0.163 g, 30%). **^{1}H NMR** (500 MHz, $CDCl_3$) δ 7.62 (d, *J* = 7.0 Hz,

2H), 7.59 (d, J = 7.0 Hz, 4H), 7.38 (t, J = 7.3 Hz, 2H), 7.31 (t, J = 7.0 Hz, 6H), 7.25 – 7.23 (m, 2H), 2.40 (s, 6H). **^{13}C NMR** (126 MHz, $CDCl_3$) δ 149.7, 138.2, 135.4, 134.0, 133.8, 133.5, 131.1, 129.9, 128.0, 126.9, 22.7. **HRMS (ESI)** calcd for $C_{26}H_{23}Si$ $[M+H]^+$ 363.1569, found 363.1570.

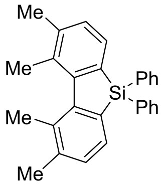

4f

4f was prepared following the **Typical Procedure D**

The reaction of 6,6'-diiodo-2,2',3,3'-tetramethyl-1,1'-biphenyl (1.620 g, 3.50 mmol, 1.0 equiv), *n*BuLi (2.4 M in hexanes, 3.40 mL, 8.05 mmol, 2.3 equiv) and dichlorodiphenylsilane (1.10 mL, 5.25 mmol, 1.5 equiv) afforded **4f** (0.167 g, 12%). **^{1}H NMR** (500 MHz, $CDCl_3$) δ 7.64 – 7.58 (m, 4H), 7.54 (d, J = 7.0 Hz, 2H), 7.37 (t, J = 7.3 Hz, 2H), 7.31 (t, J = 7.3 Hz, 4H), 7.14 (d, J = 7.0 Hz, 2H), 2.36 (s, 6H), 2.26 (s, 6H). **^{13}C NMR** (126 MHz, $CDCl_3$) δ 150.6, 140.4, 135.6, 135.4, 134.1, 133.3, 130.8, 129.8, 128.6, 127.9, 20.6, 20.0. **HRMS (ESI)** calcd for $C_{28}H_{27}Si$ $[M+H]^+$ 391.1882, found 391.1877.

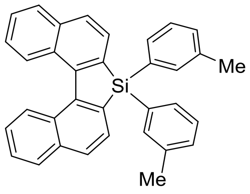

4g

4g was prepared following the **Typical Procedure D**

The reaction of 2,2'-dibromo-1,1'-binaphthalene (0.618 g, 1.50 mmol, 1.0 equiv), *n*BuLi (2.4 M in hexanes, 1.40 mL, 3.45 mmol, 2.3 equiv) and dichlorodi(*m*-tolyl)silane (0.506 g, 1.80 mmol, 1.2 equiv) afforded **4g** (0.208 g, 30%). **^{1}H NMR** (400 MHz, $CDCl_3$) δ 8.02 (d, J = 8.8 Hz, 2H), 7.94 (d, J = 8.0 Hz, 4H), 7.90 (d, J = 7.6 Hz, 2H), 7.56 – 7.50 (m, 2H), 7.50 – 7.44 (m, 4H), 7.43 – 7.35 (m, 2H), 7.26 – 7.18 (m, 4H), 2.28 (s, 6H). **^{13}C NMR** (100 MHz, $CDCl_3$) δ 148.4, 137.6, 137.0, 136.02, 135.98, 132.7, 132.3, 131.1, 129.8, 129.4, 128.3, 128.2, 128.0, 126.0, 124.3, 21.5. **HRMS (ESI)** calcd for $C_{34}H_{27}Si$ $[M+H]^+$ 463.1882, found 463.1886.

Typical procedure for the preparation of silafluorene (Typical Procedure E)

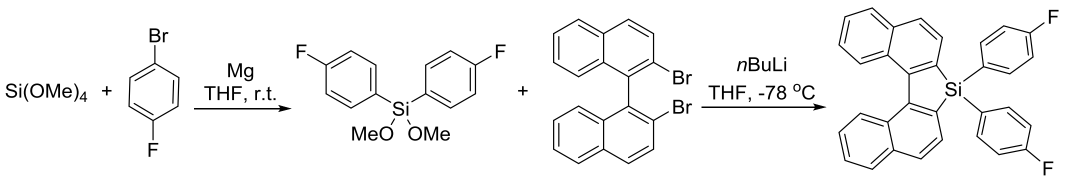

To an oven-dried flask charged with magnesium powder (0.225 g, 9.38 mmol, 2.5 equiv) and a

piece of iodine was added 1/3 amount of solution of 1-bromo-4-fluorobenzene (1.320 g, 7.50 mmol, 2.0 equiv) in THF (5 mL) to initiate the reaction. After initiation, the rest of the solution was added dropwise. After stirring at room temperature for 30 minutes, a solution of tetramethyl silicate (0.571 g, 3.75 mmol, 1.0 equiv) in THF (2 mL) was added dropwise and stirred for additional 30 minutes. The mixture was filtered via syringe and the filtrate was used directly without further purification.

To the mixture of 2,2'-dibromo-1,1'-binaphthalene (1.030 g, 2.50 mmol, 0.7 equiv) in THF (50 mL) was added *n*BuLi (2.4 M in hexanes, 2.20 mL, 5.25 mmol, 1.4 equiv) dropwise at -78 ^{o}C. After stirring at the same temperature for 2 hours, a solution of bis(4-fluorophenyl)dimethoxysilane (**S6**) in THF was added dropwise. The mixture was stirred for additional 12 hours at -78 ^{o}C before it was allowed to warm to room temperature. The reaction was quenched with water (10 mL), the resulting mixture was extracted with ethyl acetate (15 mL*3). The combined organic layer was washed with brine and dried with Na_2SO_4, concentrated and purified by flash column chromatography (PE/EtOAc = 99:1) on silica gel which was pretreated by Et_3N to give the desired product **4h** (0.599 g, 51%). **^{1}H NMR** (400 MHz, $CDCl_3$) δ 8.02 (d, *J* = 8.4 Hz, 2H), 7.94 (t, *J* = 9.2 Hz, 4H), 7.88 (d, *J* = 7.6 Hz, 2H), 7.62 (t, *J* = 7.2 Hz, 4H), 7.55 (t, *J* = 7.4 Hz, 2H), 7.41 (t, *J* = 7.6 Hz, 2H), 7.05 (t, *J* = 8.8 Hz, 4H). **^{13}C NMR** (100 MHz, $CDCl_3$) δ 164.5 (d, $^1J_{C\text{-}F}$ = 249 Hz), 148.6, 137.6 (d, $^3J_{C\text{-}F}$ = 5.7 Hz), 136.2, 136.1, 129.8, 129.0, 128.5, 128.4, 128.0, 127.7 (d, $^4J_{C\text{-}F}$ = 3.8 Hz), 126.3, 124.6, 115.6 (d, $^2J_{C\text{-}F}$ = 19.9 Hz). **^{19}F NMR** (376 MHz, $CDCl_3$) δ -109.4. **HRMS (ESI)** calcd for $C_{32}H_{21}F_2Si$ $[M+H]^+$ 471.1381, found 471.1376.

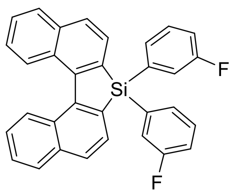

4i

4i was prepared following the **Typical Procedure E**

The reaction of 2,2'-dibromo-1,1'-binaphthalene (1.030 g, 2.50 mmol, 0.7 equiv), tetramethyl silicate (0.571 g, 3.75 mmol, 1.0 equiv) and 1-bromo-3-fluorobenzene (1.320 g, 7.50 mmol, 2.0 equiv) afforded **4i** (0.448 g, 38%). **^{1}H NMR** (500 MHz, $CDCl_3$) δ 8.05 (d, *J* = 8.5 Hz, 2H), 7.98 (d, *J* = 5.0 Hz, 2H), 7.97 – 7.92 (m, 4H), 7.59 – 7.55 (m, 2H), 7.47 – 7.42 (m, 4H), 7.38 (dd, *J* = 8.5, 3.0 Hz, 2H), 7.34 (dd, *J* = 7.5, 5.5 Hz, 2H), 7.15 – 7.10 (m, 2H). **^{13}C NMR** (126 MHz, $CDCl_3$) δ 162.6 (d, $^1J_{C\text{-}F}$ = 250.0 Hz), 148.8, 136.1, 135.3, 134.9 (d, $^3J_{C\text{-}F}$ = 4.3 Hz), 131.1 (d, $^4J_{C\text{-}F}$ = 3.2 Hz), 130.2 (d, $^3J_{C\text{-}F}$ = 7.1 Hz), 129.8, 129.0, 128.6, 128.4, 128.0, 126.4, 124.7, 121.8 (d, $^2J_{C\text{-}F}$ = 19.3 Hz), 117.5 (d, $^2J_{C\text{-}F}$ = 21.0 Hz). **^{19}F NMR** (471 MHz, $CDCl_3$) δ - 112.4. **HRMS (ESI)** calcd for $C_{32}H_{21}F_2Si$ $[M+H]^+$ 471.1381, found 471.1380.

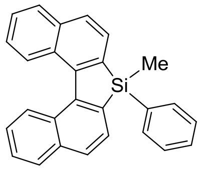

4j

4j was prepared following the **Typical Procedure D**

The reaction of 2,2'-dibromo-1,1'-binaphthalene (0.618 g, 1.50 mmol, 1.0 equiv), *n*BuLi (2.4 M in hexanes, 1.40 mL, 3.45 mmol, 2.3 equiv) and dichloro(methyl)(phenyl)silane (0.344 g, 1.80 mmol, 1.2 equiv) afforded **4j** (0. 415 g, 74%). **^{1}H NMR** (500 MHz, $CDCl_3$) δ 8.02 (d, *J* = 8.5 Hz, 2H), 7.94 (t, *J* = 8.5 Hz, 2H), 7.89 (d, *J* = 7.5 Hz, 1H), 7.87 (d, *J* = 7.5 Hz, 1H), 7.82 (d, *J* = 7.5 Hz, 1H), 7.79 (d, *J* = 7.5 Hz, 1H), 7.55 – 7.52 (m, 1H), 7.52- 7.51 (m, 1H), 7.51 – 7.49 (m, 2H), 7.42 – 7.37 (m, 2H), 7.35 (d, *J* = 7.5 Hz, 1H), 7.32 – 7.27 (m, 2H), 0.79 (s, 3H). **^{13}C NMR** (126 MHz, $CDCl_3$) δ 148.1, 147.9, 138.6, 138.2, 135.91, 135.86, 134.5, 134.3, 130.0, 129.8, 129.7, 129.0, 128.8, 128.3, 128.08, 128.05, 128.0, 127.9, 125.94, 125.92, 124.4, 124.3, -5.3. **HRMS (ESI)** calcd for $C_{27}H_{21}Si$ $[M+H]^+$ 373.1413, found 373.1411.

Typical procedure for Rh-catalyzed ring-opening/acylation reaction (Typical Procedure F)

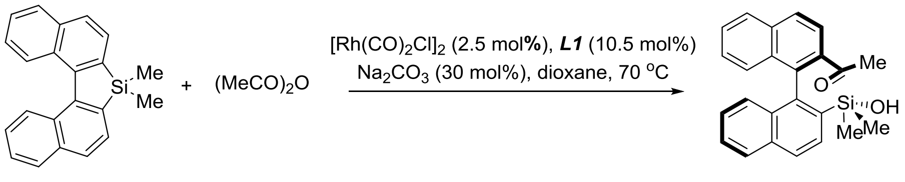

Under nitrogen atmosphere, to a Schleck tube was added $[Rh(CO)_2Cl]_2$ (2.0 mg, 0.005 mmol, 2.5 mol%), **L1** (24.5 mg, 0.021 mmol, 10.5 mol%) and dioxane (2 mL) at room temperature and was stirred for 30 minutes. The solution was transfer via cannula carefully to another Schlenk tube charged with **4a** (62 mg, 0.200 mmol, 1.0 equiv), acetic anhydride (28 μL, 0.300 mmol, 1.5 equiv), Na_2CO_3 (6.4 mg, 0.060 mmol, 30 mol%) and dioxane (2 mL). The tube was capped with a screw cap and stirred at 70 °C for 24 h. After being cooled to room temperature, the mixture was filtered through Celite and the filtrate was concentrated in vacuum and purified by flash column chromatography (PE/EtOAc = 90:10) on silica gel to afford **6a** (73 mg, 98%, 90% ee). $[\alpha]_D^{20}$ –2.25 (c 1.13, CH_2Cl_2). HPLC conditions: Chiralcel AD-H, isopropanol/hexane = 10:90, flow: 1.0 mL/min, λ = 254 nm. **^{1}H NMR** (500 MHz, $CDCl_3$) δ 8.04 (d, *J* = 9.0 Hz, 1H), 7.99 (d, *J* = 8.0 Hz, 1H), 7.94 (d, *J* = 8.5 Hz, 1H), 7.92 (d, *J* = 8.5 Hz, 1H), 7.87 (d, *J* = 8.0 Hz, 1H), 7.80 (d, *J* = 9.0 Hz, 1H), 7.55 – 7.50 (m, 1H), 7.49 – 7.44 (m, 1H), 7.31 – 7.26 (m, 1H), 7.26 – 7.22 (m, 1H), 7.17 (d, *J* = 8.5 Hz, 1H), 7.13 (d, *J* = 8.5 Hz, 1H), 2.70 (s, 1H), 1.94 (s, 3H), 0.13 (s, 3H), -0.47 (s, 3H). **^{13}C NMR** (126 MHz, $CDCl_3$) δ 205.2, 141.5, 138.3, 137.9, 137.4, 134.2, 133.7, 133.5, 132.7, 130.4, 128.7, 128.2, 128.0, 127.6, 127.5, 127.0, 126.6, 126.5, 126.2, 123.9, 30.5, 0.6. **HRMS (ESI)** calcd for $C_{24}H_{22}O_2SiNa$ $[M+Na]^+$ 393.1287, found 393.1288.

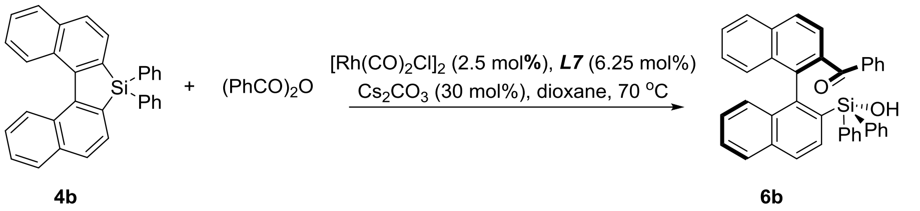

Compound **6b** was prepared following the **Typical Procedure F**

The reaction of **4b** (87 mg, 0.20 mmol) and benzoic anhydride (68 mg, 0.30 mmol) with $[Rh(CO)_2Cl]_2$ (2.0 mg, 0.005 mmol, 2.5 mol%), **L7** (14.6 mg, 0.013 mmol, 6.25 mol%) afforded **6b** (100 mg, 90%, 91% ee). $[\alpha]_D^{20}$ – 45.31 (c 1.05, CH_2Cl_2). HPLC conditions: Chiralcel AD-H, isopropanol/hexane = 20:80, flow: 1.0 mL/min, λ = 254 nm. **^{1}H NMR** (400 MHz, $CDCl_3$) δ 7.88 (d, *J* = 8.4 Hz, 1H), 7.82 – 7.77 (m, 2H), 7.74 (d, *J* = 4.8 Hz, 1H), 7.73 – 7.69 (m, 2H), 7.66 – 7.60 (m, 3H), 7.52 (d, *J* = 8.4 Hz, 1H), 7.45 – 7.40 (m, 2H), 7.40 – 7.34 (m, 2H), 7.34 – 7.27 (m, 2H), 7.20 (t, *J* = 7.8 Hz, 2H), 7.11 – 7.06 (m, 2H), 7.05 – 7.01 (m, 2H), 7.01 – 6.95 (m, 1H), 6.93 – 6.86 (m, 1H), 6.85 – 6.78 (m, 3H), 5.33 (bs, 1H). **^{13}C NMR** (100 MHz, $CDCl_3$) δ 200.8, 142.0, 137.4, 136.73, 136.66, 136.3, 136.0, 135.2, 134.5, 133.7, 133.6, 133.5, 133.4, 132.9, 132.5, 132.0, 130.5, 129.5, 128.5, 127.9, 127.84, 127.82, 127.7, 127.5, 127.0, 126.9, 126.8, 126.6, 126.5, 126.3, 125.7, 124.0. **HRMS (ESI)** calcd for $C_{39}H_{28}O_2SiNa$ $[M+Na]^+$ 579.1756, found 579.1752.

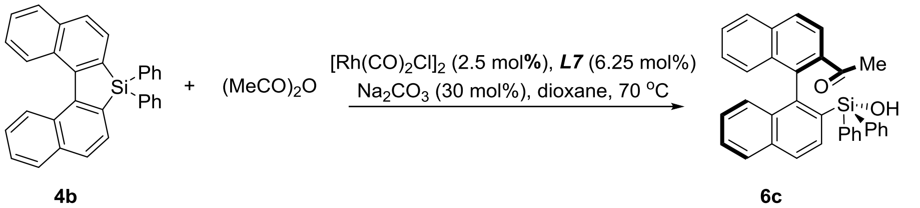

Compound **6c** was prepared following the **Typical Procedure F**

The reaction of **4b** (87 mg, 0.20 mmol) and acetic anhydride (28 μL, 0.30 mmol) with $[Rh(CO)_2Cl]_2$ (2.0 mg, 0.005 mmol, 2.5 mol%), **L7** (14.6 mg, 0.013 mmol, 6.25 mol%) afforded **6c** (97 mg, 98%, 92% ee). $[\alpha]_D^{20}$ + 17.60 (c 0.98, CH_2Cl_2). HPLC conditions: Chiralcel AD-H, isopropanol/hexane = 15:85, flow: 1.0 mL/min, λ = 254 nm. **^{1}H NMR** (400 MHz, $CDCl_3$) δ 7.92 – 7.88 (m, 2H), 7.86 (d, *J* = 8.4 Hz, 1H), 7.71 (d, *J* = 8.4 Hz, 1H), 7.68 (d, *J* = 8.4 Hz, 1H), 7.65 (d, *J* = 8.4 Hz, 1H), 7.61 (t, *J* = 1.4 Hz, 1H), 7.61 – 7.59 (m, 1H), 7.49 – 7.44 (m, 1H), 7.42 – 7.37 (m, 1H), 7.36 – 7.33 (m, 2H), 7.33 – 7.30 (m, 1H), 7.25 – 7.19 (m, 1H), 7.09 – 7.06 (m, 2H), 7.06 – 7.01 (m, 2H), 6.99 – 6.93 (m, 1H), 6.92 – 6.89 (m, 2H), 6.89 – 6.85 (m, 1H), 3.43 (s, 1H), 1.99 (s, 3H). **^{13}C NMR** (100 MHz, CDC_3) δ 205.8, 143.2, 138.0, 136.5, 135.8, 134.9, 134.7, 134.4, 133.9, 133.8, 133.7, 132.9, 132.8, 132.2, 129.6, 128.9, 128.8, 128.1, 128.0, 127.7, 127.6, 127.2, 127.1, 127.0, 126.8, 126.6, 126.5, 126.2, 123.2, 30.4. **HRMS (ESI)** calcd for $C_{34}H_{26}O_2SiNa$ $[M+Na]^+$ 517.1600, found 517.1600.

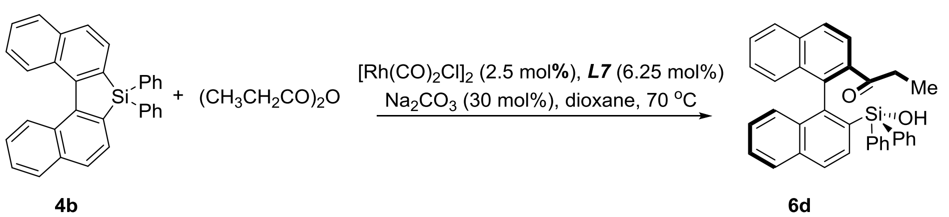

Compound **6d** was prepared following the **Typical Procedure F**

The reaction of **4b** (87 mg, 0.20 mmol) and propionic anhydride (28 μL, 0.30 mmol) with $[Rh(CO)_2Cl]_2$ (2.0 mg, 0.005 mmol, 2.5 mol%), **L7** (14.6 mg, 0.013 mmol, 6.25 mol%) afforded **6d** (97 mg, 98%, 92% ee). $[\alpha]_D^{20}$ + 19.33 (c 0.97, CH_2Cl_2). HPLC conditions: Chiralcel AD-H, isopropanol/hexane = 15:85, flow: 1.0 mL/min, λ = 254 nm. **^{1}H NMR** (400 MHz, $CDCl_3$) δ 7.92 – 7.87 (m, 2H), 7.86 (d, *J* = 8.4 Hz, 1H), 7.73 – 7.68 (m, 2H), 7.68 – 7.64 (m, 2H), 7.59 (d, *J* = 8.8 Hz, 1H), 7.46 (t, *J* = 7.4 Hz, 1H), 7.42 – 7.37 (m, 2H), 7.37 – 7.34 (m, 1H), 7.31 (t, *J* = 7.6 Hz, 1H), 7.22 (t, *J* = 7.8 Hz, 1H), 7.08 (d, *J* = 8.8 Hz, 2H), 7.05 – 6.98 (m, 2H), 6.94 (t, *J* = 7.8 Hz, 1H), 6.87 (t, *J* = 7.8 Hz, 3H), 4.07 (s, 1H), 2.55 – 2.41 (m, 1H), 2.39 – 2.22 (m, 1H), 0.76 (t, *J* = 7.0 Hz, 3H). **^{13}C NMR** (100 MHz, $CDCl_3$) δ 209.7, 143.0, 138.4, 136.1, 135.8, 135.0, 134.9, 134.4, 133.9, 133.6, 133.5, 132.81, 132.78, 132.2, 129.6, 128.73, 128.71, 128.2, 127.8, 127.7, 127.5, 127.1, 126.9, 126.7, 126.5, 126.3, 126.2, 122.7, 36.1, 7.9. **HRMS (ESI**) calcd for $C_{35}H_{28}O_2SiNa$ $[M+Na]^+$ 531.1756, found 531.1757.

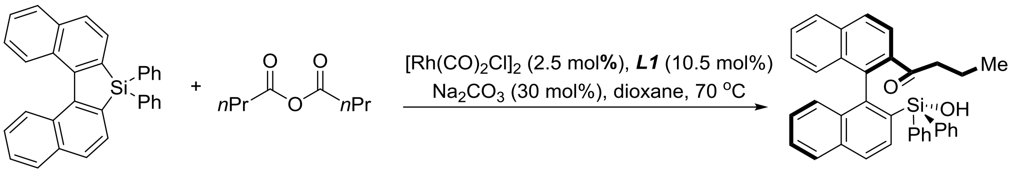

4b **6e**

Compound **6e** was prepared following the **Typical Procedure F**

The reaction of **4b** (87 mg, 0.20 mmol) and butyric anhydride (49 μL, 0.30 mmol) with $[Rh(CO)_2Cl]_2$ (2.0 mg, 0.005 mmol, 2.5 mol%), **L1** (18.9 mg, 0.021 mmol, 10.5 mol%) afforded **6e** (61 mg, 59%, 85% ee). $[\alpha]_D^{20}$ + 14.26 (c 1.00, CH_2Cl_2). HPLC conditions: Chiralcel AD-H, isopropanol/hexane = 10:90, flow: 1.0 mL/min, λ = 254 nm. **^{1}H NMR** (400 MHz, $CDCl_3$) δ 7.88 (d, *J* = 7.6 Hz, 1H), 7.85 (d, *J* = 7.2 Hz, 2H), 7.72 – 7.68 (m, 2H), 7.68 – 7.64 (m, 2H), 7.54 (d, *J* = 8.4 Hz, 1H), 7.45 (t, *J* = 7.6 Hz, 1H), 7.42 – 7.36 (m, 2H), 7.36 – 7.33 (m, 1H), 7.29 (t, *J* = 7.6 Hz, 1H), 7.22 (t, *J* = 7.6 Hz, 1H), 7.07 (d, *J* = 8.8 Hz, 1H), 7.02 (d, *J* = 7.6 Hz, 2H), 6.98 (d, *J* = 7.6 Hz, 1H), 6.92 (t, *J* = 7.6 Hz, 1H), 6.84 (d, *J* = 8.0 Hz, 2H), 6.83 – 6.71 (m, 1H), 4.27 (s, 1H), 2.41 (dt, *J* = 17.6, 7.0 Hz, 1H), 2.26 (dt, *J* = 17.6, 7.0 Hz, 1H), 1.36 – 1.20 (m, 2H), 0.44 (t, *J* = 7.4 Hz, 3H). **^{13}C NMR** (100 MHz, $CDCl_3$) δ 210.0, 142.8, 138.9, 136.2, 135.5, 135.40, 135.0, 134.4, 133.9, 133.6, 133.4, 132.8, 132.7, 132.2, 129.6, 128.72, 128.68, 128.1, 127.8, 127.7, 127.5, 127.1, 126.84, 126.82, 126.7, 126.5, 126.33, 126.27, 122.5, 44.8, 16.9, 13.1. **HRMS (ESI)** calcd for $C_{36}H_{30}O_2SiNa$ $[M+Na]^+$545.1913, found 545.1906.

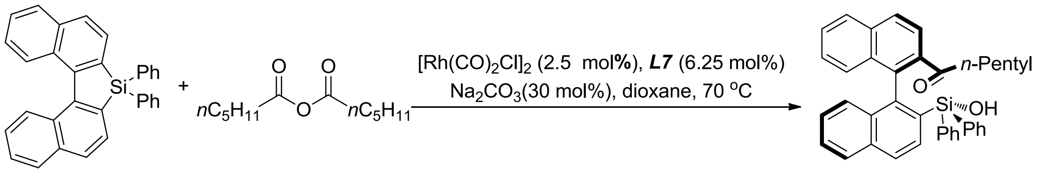

4b **6f**

Compound **6f** was prepared following the **Typical Procedure F**

The reaction of **4b** (87 mg, 0.20 mmol) and hexanoic anhydride (69 μL, 0.30 mmol) with $[Rh(CO)_2Cl]_2$ (2.0 mg, 0.005 mmol, 2.5 mol%), **L7** (14.6 mg, 0.013 mmol, 6.25 mol%) afforded

6f (49 mg, 45%, 86% ee). $[\alpha]_D^{20}$ + 8.33 (c 0.93, CH_2Cl_2). HPLC conditions: Chiralcel AD-H, isopropanol/hexane = 10:90, flow: 1.0 mL/min, λ = 254 nm. **^{1}H NMR** (400 MHz, $CDCl_3$) δ 7.88 (d, *J* = 7.6 Hz, 1H), 7.85 (d, *J* = 7.2 Hz, 1H), 7.71 – 7.67 (m, 2H), 7.67 – 7.63 (m, 2H), 7.53 (d, *J* = 8.8 Hz, 1H), 7.45 (t, *J* = 7.2 Hz, 1H), 7.43 – 7.37 (m, 2H), 7.35 (d, *J* = 6.8 Hz, 2H), 7.29 (t, *J* = 7.6 Hz, 1H), 7.21 (t, *J* = 7.6 Hz, 1H), 7.07 (d, *J* = 8.4 Hz, 1H), 7.02 (d, *J* = 7.2 Hz, 2H), 6.97 (d, *J* = 7.6 Hz, 1H), 6.92 (t, *J* = 8.0 Hz, 1H), 6.84 (d, *J* = 8.0 Hz, 2H), 6.83 – 6.80 (m, 1H), 4.27 (s, 1H), 2.48 – 2.34 (m, 1H), 2.34 – 2.22 (m, 1H), 1.28 – 1.14 (m, 2H), 1.00 – 0.88 (m, 2H), 0.81 – 0.70 (m, 2H), 0.68 (t, *J* = 7.2 Hz, 3H). **^{13}C NMR** (100 MHz, $CDCl_3$) δ 210.2, 142.8, 139.0, 136.2, 135.5, 135.0, 134.4, 133.9, 133.6, 133.4, 132.8, 132.7, 132.2, 129.6, 128.73, 128.68, 128.1, 127.8, 127.7, 127.5, 127.1, 126.9, 126.8, 126.7, 126.5, 126.4, 126.3, 122.5, 42.9, 34.8, 23.1, 22.1, 13.8. **HRMS (ESI)** calcd for $C_{38}H_{34}O_2SiNa$ $[M+Na]^+$ 573.2226, found 573.2219.

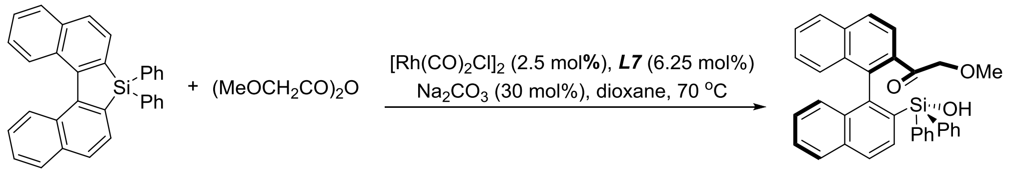

4b **6g**

Compound **6g** was prepared following the **Typical Procedure F**

The reaction of **4b** (87 mg, 0.20 mmol) and 2-methoxyacetic anhydride (49 mg, 0.30 mmol) with $[Rh(CO)_2Cl]_2$ (2.0 mg, 0.005 mmol, 2.5 mol%), **L7** (14.6 mg, 0.013 mmol, 6.25 mol%) afforded **6g** (97 mg, 93%, 88% ee). $[\alpha]_D^{20}$ + 16.34 (c 0.98, CH_2Cl_2). HPLC conditions: Chiralcel AD-H, isopropanol/hexane = 20:80, flow: 1.0 mL/min, λ = 254 nm. **^{1}H NMR** (400 MHz, $CDCl_3$) δ 7.89 (d, *J* = 8.4 Hz, 2H), 7.88 (d, *J* = 8.4 Hz, 1H), 7.73 (d, *J* = 8.0 Hz, 1H), 7.68 (d, *J* = 8.0 Hz, 1H), 7.66 – 7.61 (m, 2H), 7.60 (d, *J* = 8.4 Hz, 1H), 7.49 – 7.43 (m, 1H), 7.43 – 7.38 (m, 1H), 7.38 – 7.34 (m, 2H), 7.34 – 7.30 (m, 1H), 7.25 – 7.20 (m, 1H), 7.09 – 7.05 (m, 2H), 7.05 – 7.00 (m, 2H), 6.97 – 6.91 (m, 1H), 6.91 – 6.88 (m, 2H), 6.88 – 6.84 (m, 1H), 4.01 (d, *J* = 17.6 Hz, 1H), 3.78 (d, *J* = 17.6 Hz, 1H), 3.72 (bs, 1H), 2.95 (s, 3H). **^{13}C NMR** (126 MHz, DMSO-d_6) δ 198.2, 143.2, 137.2, 136.3, 135.4, 133.7, 133.6, 133.5, 133.3, 133.2, 132.8, 132.34, 132.33, 131.4, 128.6, 128.2, 127.7, 127.4, 127.2, 127.0, 126.7, 126.6, 126.3, 126.2, 125.9, 125.8, 125.6, 125.5, 123.4, 75.4, 57.5. **HRMS (ESI)** calcd for $C_{35}H_{28}O_3SiNa$ $[M+Na]^+$ 547.1705, found 547.1699.

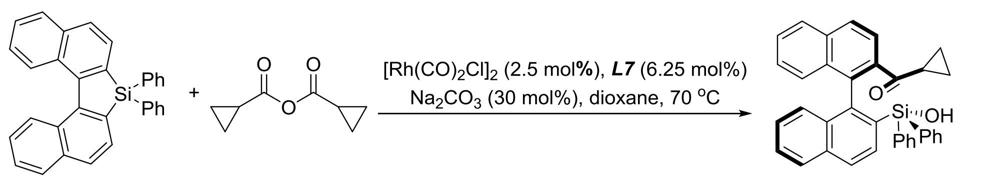

4b **6h**

Compound **6h** was prepared following the **Typical Procedure F**

The reaction of **4b** (87 mg, 0.20 mmol) and cyclopropanecarboxylic anhydride (46 mg, 0.30 mmol) with $[Rh(CO)_2Cl]_2$ (2.0 mg, 0.005 mmol, 2.5 mol%), **L7** (14.6 mg, 0.013 mmol, 6.25 mol%) afforded **6h** (84 mg, 81%, 85% ee). $[\alpha]_D^{20}$ + 40.51 (c 0.98, CH_2Cl_2). HPLC conditions: Chiralcel AD-H, isopropanol/hexane = 20:80, flow: 1.0 mL/min, λ = 254 nm. **^{1}H NMR** (500 MHz, $CDCl_3$) δ 7.88 (d, *J* = 8.5 Hz, 1H), 7.84 (d, *J* = 8.5 Hz, 2H), 7.72 – 7.68 (m, 2H), 7.68 – 7.64 (m,

2H), 7.63 (d, J = 8.5 Hz, 1H), 7.45 (t, J = 7.3 Hz, 1H), 7.40 – 7.38 (m, 1H), 7.38 – 7.33 (m, 2H), 7.29 (t, J = 7.5 Hz, 1H), 7.24 – 7.20 (m, 1H), 7.16 (d, J = 8.5 Hz, 1H), 7.02 – 6.97 (m, 2H), 6.94 (t, J = 7.5 Hz, 2H), 6.89 (d, J = 8.5 Hz, 1H), 6.79 (t, J = 7.5 Hz, 2H), 4.38 (s, 1H), 1.96 – 1.79 (m, 1H), 1.15 – 1.03 (m, 1H), 0.76 – 0.60 (m, 1H), 0.48 – 0.32 (m, 1H), 0.21 – 0.12 (m, 1H). ^{13}**C NMR** (126 MHz, $CDCl_3$) δ 210.5, 143.2, 139.9, 136.3, 135.6, 135.5, 135.0, 134.3, 133.8, 133.5, 133.4, 133.1, 132.6, 132.4, 129.5, 128.8, 128.6, 128.0, 127.8, 127.7, 127.6, 127.1, 126.8, 126.6, 126.50, 126.46, 126.2, 122.7, 22.4, 13.5, 12.5. **HRMS (ESI)** calcd for $C_{36}H_{28}O_2SiNa$ $[M+Na]^+$ 543.1756, found 543.1759.

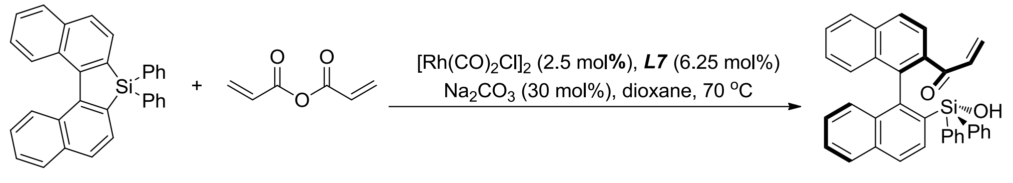

4b **6i**

Compound **6i** was prepared following the **Typical Procedure F**

The reaction of **4b** (87 mg, 0.20 mmol) and acrylic anhydride (36 μL, 0.30 mmol) with $[Rh(CO)_2Cl]_2$ (2.0 mg, 0.005 mmol, 2.5 mol%), **L7** (14.6 mg, 0.013 mmol, 6.25 mol%) afforded **6i** (83 mg, 82%, 93% ee). $[\alpha]_D^{20}$ + 12.12 (c 0.96, CH_2Cl_2). HPLC conditions: Chiralcel AD-H, isopropanol/hexane = 15:85, flow: 1.0 mL/min, λ = 254 nm. ^{1}**H NMR** (500 MHz, $CDCl_3$) δ 7.89 – 7.85 (m, 1H), 7.84 (d, J = 4.5 Hz, 2H), 7.73 – 7.71 (m, 1H), 7.71 – 7.69 (m, 2H), 7.66 (d, J = 8.0 Hz, 1H), 7.56 (d, J = 8.5 Hz, 1H), 7.45 – 7.39 (m, 2H), 7.39 – 7.37 (m, 1H), 7.37 – 7.34 (m, 1H), 7.34 – 7.29 (m, 1H), 7.21 – 7.14 (m, 1H), 7.08 – 7.03 (m, 2H), 7.03 – 6.97 (m, 2H), 6.95 – 6.88 (m, 1H), 6.87 – 6.85 (m, 1H), 6.85 – 6.84 (m, 1H), 6.84 – 6.82 (m, 1H), 6.27 (dd, J = 17.5, 10.5 Hz, 1H), 5.99 (dd, J = 17.5, 1.0 Hz, 1H), 5.68 (dd, J = 10.5, 1.0 Hz, 1H), 4.52 (s, 1H). ^{13}**C NMR** (126 MHz, $CDCl_3$) δ 199.5, 142.3, 137.1, 136.7, 136.4, 135.72, 135.69, 135.0, 134.4, 133.9, 133.6, 132.8, 132.72, 132.69, 132.1, 129.5, 128.7, 128.3, 128.1, 127.8, 127.7, 127.6, 127.1, 127.0, 126.9, 126.6, 126.50, 126.45, 126.1, 123.5. **HRMS (ESI)** calcd for $C_{35}H_{26}O_2SiNa$ $[M+Na]^+$ 529.1600, found 529.1597.

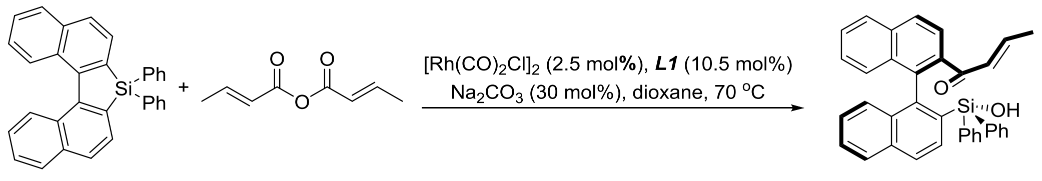

4b **6j**

Compound **6j** was prepared following the **Typical Procedure F**

The reaction of **4b** (87 mg, 0.20 mmol) and *(E)*-but-2-enoic anhydride (45 μL, 0.30 mmol) with $[Rh(CO)_2Cl]_2$ (2.0 mg, 0.005 mmol, 2.5 mol%), **L1** (18.9 mg, 0.021 mmol, 10.5 mol%) afforded **6j** (104 mg, 99%, 85% ee). $[\alpha]_D^{20}$ – 0.41 (c 1.00, CH_2Cl_2). HPLC conditions: Chiralcel AD-H, isopropanol/hexane = 20:80, flow: 1.0 mL/min, λ = 254 nm. ^{1}**H NMR** (400 MHz, $CDCl_3$) δ 7.89 – 7.85 (m, 1H), 7.85 – 7.80 (m, 2H), 7.76 – 7.72 (m, 2H), 7.70 (d, J = 8.4 Hz, 1H), 7.63 (d, J = 8.0 Hz, 1H), 7.50 (d, J = 8.4 Hz, 1H), 7.45 – 7.41 (m, 1H), 7.41 – 7.38 (m, 2H), 7.38 – 7.34 (m, 1H), 7.29 (d, J = 7.6 Hz, 1H), 7.16 (t, J = 7.6 Hz, 1H), 7.07 – 7.03 (m, 1H), 7.03 – 6.99 (m, 2H), 6.99 – 6.93 (m, 1H),

6.91 – 6.84 (m, 1H), 6.84 – 6.81 (m, 2H), 6.81 – 6.77 (m, 1H), 6.63 – 6.51 (m, 1H), 5.99 (d, *J* = 15.6 Hz, 1H), 5.18 (s, 1H), 1.56 (d, *J* = 6.8 Hz, 3H). **^{13}C NMR** (100 MHz, $CDCl_3$) δ 200.3, 149.8, 142.3, 137.5, 136.6, 136.3, 135.9, 135.1, 134.4, 133.9, 133.6, 133.3, 132.68, 132.65, 132.3, 131.8, 129.5, 128.5, 128.2, 128.1, 127.7, 127.6, 127.5, 127.0, 126.8, 126.7, 126.64, 126.62, 126.43, 126.38, 125.8, 123.3, 18.3. **HRMS (ESI)** calcd for $C_{36}H_{28}O_2SiNa$ $[M+Na]^+$ 543.1756, found 543.1754.

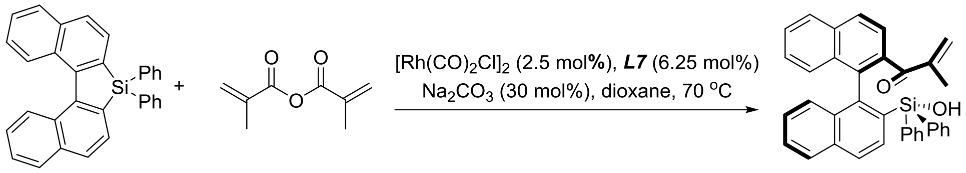

4b **6k**

Compound **6k** was prepared following the **Typical Procedure F**

The reaction of **4b** (87 mg, 0.20 mmol) and methacrylic anhydride (45 μL, 0.30 mmol) with $[Rh(CO)_2Cl]_2$ (2.0 mg, 0.005 mmol, 2.5 mol%), **L7** (14.6 mg, 0.013 mmol, 6.25 mol%) afforded **6k** (69 mg, 66%, 92% ee). $[\alpha]_D^{20}$ + 10.04 (c 1.00, CH_2Cl_2). HPLC conditions: Chiralcel AD-H, isopropanol/hexane = 20:80, flow: 1.0 mL/min, λ = 230 nm. **^{1}H NMR** (400 MHz, $CDCl_3$) δ 7.88 – 7.84 (m, 1H), 7.84 – 7.80 (m, 2H), 7.80 – 7.75 (m, 2H), 7.71 (d, *J* = 8.4 Hz, 1H), 7.64 (d, *J* = 8.4 Hz, 1H), 7.47 (d, *J* = 8.4 Hz, 1H), 7.44 – 7.41 (m, 2H), 7.41 – 7.35 (m, 2H), 7.32 – 7.27 (m, 1H), 7.14 (t, *J* = 7.8 Hz, 1H), 7.05 – 7.00 (m, 2H), 7.00 – 6.95 (m, 2H), 6.88 – 6.84 (m, 1H), 6.84 – 6.79 (m, 2H), 6.76 (d, *J* = 8.8 Hz, 1H), 5.70 (s, 1H), 5.61 (s, 1H), 5.37 (s, 1H), 1.59 (s, 3H). **^{13}C NMR** (100 MHz, $CDCl_3$) δ 203.3, 143.9, 142.1, 137.1, 136.8, 136.4, 136.1, 135.1, 134.4, 133.8, 133.6, 133.2, 132.65, 132.55, 132.1, 132.0, 129.5, 128.5, 128.0, 127.74, 127.70, 127.6, 127.4, 127.0, 126.9, 126.8, 126.6, 126.4, 126.3, 125.8, 123.7, 16.8. **HRMS (ESI)** calcd for $C_{36}H_{28}O_2SiNa$ $[M+Na]^+$ 543.1756, found 543.1758.

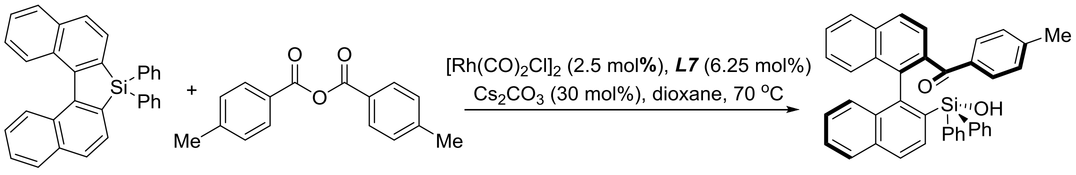

4b **6l**

Compound **6l** was prepared following the **Typical Procedure F**

The reaction of **4b** (87 mg, 0.20 mmol) and 4-methylbenzoic anhydride (76 mg, 0.30 mmol) with $[Rh(CO)_2Cl]_2$ (2.0 mg, 0.005 mmol, 2.5 mol%), **L7** (14.6 mg, 0.013 mmol, 6.25 mol%) afforded **6l** (63 mg, 55%, 88% ee). $[\alpha]_D^{20}$ – 88.03 (c 1.04, CH_2Cl_2). HPLC conditions: Chiralcel AD-H, isopropanol/hexane = 20:80, flow: 1.0 mL/min, λ = 254 nm. **^{1}H NMR** (400 MHz, $CDCl_3$) δ 7.86 (d, *J* = 8.4 Hz, 1H), 7.82 – 7.79 (m, 1H), 7.79 – 7.76 (m, 1H), 7.74 (d, *J* = 8.4 Hz, 1H), 7.71 (d, *J* = 8.0 Hz, 2H), 7.62 (d, *J* = 8.0 Hz, 1H), 7.57 – 7.52 (m, 2H), 7.50 (d, *J* = 8.4 Hz, 1H), 7.45 – 7.40 (m, 2H), 7.40 – 7.35 (m, 1H), 7.31 – 7.26 (m, 2H), 7.10 – 7.05 (m, 2H), 7.05 – 7.02 (m, 2H), 7.02 – 6.99 (m, 2H), 6.99 – 6.93 (m, 1H), 6.89 – 6.84 (m, 1H), 6.83 – 6.78 (m, 2H), 6.78 – 6.75 (m, 1H), 5.59 (s, 1H), 2.28 (s, 3H).**^{13}C NMR** (100 MHz, $CDCl_3$) δ 200.3, 144.6, 142.0, 137.3, 136.82, 136.77, 136.0, 135.2, 134.5, 133.8, 133.7, 133.5, 133.4, 133.0, 132.5, 132.0, 130.8, 129.5, 128.7, 128.5, 127.84, 127.81, 127.7, 127.5, 126.9, 126.8, 126.6, 126.4, 126.3, 125.7, 124.1, 21.7. **HRMS**

(ESI) calcd for $C_{40}H_{30}O_2SiNa$ $[M+Na]^+$ 593.1914, found 593.1913.

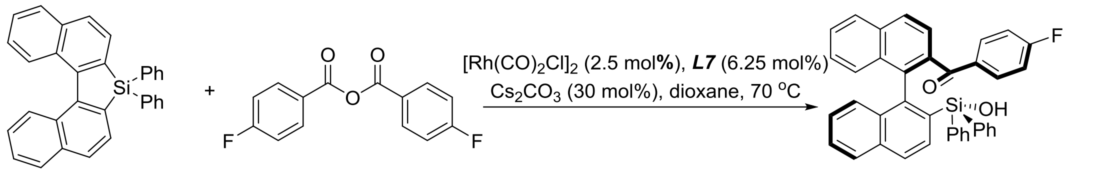

Compound **6m** was prepared following the **Typical Procedure F**

The reaction of **4b** (87 mg, 0.20 mmol) and 4-fluorobenzoic anhydride (79 mg, 0.30 mmol) with $[Rh(CO)_2Cl]_2$ (2.0 mg, 0.005 mmol, 2.5 mol%), **L7** (14.6 mg, 0.013 mmol, 6.25 mol%) afforded **6m** (76 mg, 67%, 92% ee). $[\alpha]_D^{20}$ – 67.08 (c 1.01, CH_2Cl_2). HPLC conditions: Chiralcel AD-H, isopropanol/hexane = 20:80, flow: 1.0 mL/min, λ = 254 nm. **^{1}H NMR** (400 MHz, $CDCl_3$) δ 7.88 (d, *J* = 8.4 Hz, 1H), 7.78 – 7.76 (m, 1H), 7.76 – 7.74 (m, 2H), 7.74 – 7.70 (m, 2H), 7.66 – 7.64 (m, 1H), 7.64 – 7.59 (m, 2H), 7.50 (d, *J* = 8.4 Hz, 1H), 7.44 – 7.38 (m, 2H), 7.38 – 7.35 (m, 1H), 7.34 – 7.31 (m, 1H), 7.31 – 7.27 (m, 1H), 7.11 – 7.05 (m, 2H), 7.05 – 7.01 (m, 2H), 7.00 – 6.95 (m, 1H), 6.94 – 6.88 (m, 1H), 6.88 – 6.85 (m, 1H), 6.85 – 6.83 (m, 2H), 6.83 – 6.79 (m, 2H), 5.15 (s, 1H). **^{13}C NMR** (100 MHz, $CDCl_3$) δ 199.3, 165.8 (d, $^1J_{C-F}$ = 254.7 Hz), 141.9, 137.5, 136.6, 136.4, 135.9, 135.1, 134.4, 133.7, 133.6, 133.5, 133.2, 133.1, 132.9, 132.7 (d, $^4J_{C-F}$ = 2.8 Hz), 132.5, 132.0, 129.5, 128.6, 128.0 (d, $^3J_{C-F}$ = 4.7 Hz), 127.8, 127.7, 127.6, 127.1, 127.0, 126.8, 126.6, 126.5, 126.4, 125.8, 123.8, 115.1 (d, $^2J_{C-F}$ = 21.8 Hz). **^{19}F NMR** (471 MHz, $CDCl_3$) δ -103.9. **HRMS (ESI)** calcd for $C_{39}H_{27}FO_2SiNa$ $[M+Na]^+$ 597.1662, found 597.1672.

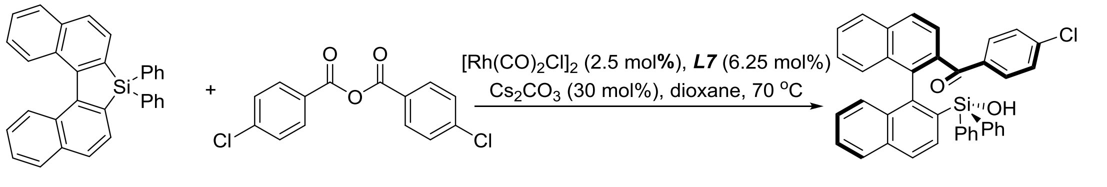

Compound **6n** was prepared following the **Typical Procedure F**

The reaction of **4b** (87 mg, 0.20 mmol) and 4-chlorobenzoic anhydride (89 mg, 0.30 mmol) with $[Rh(CO)_2Cl]_2$ (2.0 mg, 0.005 mmol, 2.5 mol%), **L7** (14.6 mg, 0.013 mmol, 6.25 mol%) afforded **6n** (114 mg, 96%, 88% ee). $[\alpha]_D^{20}$ – 98.06 (c 1.00, CH_2Cl_2). HPLC conditions: Chiralcel AD-H, isopropanol/hexane = 20:80, flow: 1.0 mL/min, λ = 274 nm. **^{1}H NMR** (400 MHz, $CDCl_3$) δ 7.89 (d, *J* = 8.4 Hz, 1H), 7.78 – 7.75 (m, 2H), 7.75 – 7.74 (m, 2H), 7.74 – 7.71 (m, 1H), 7.62 (d, *J* = 8.4 Hz, 1H), 7.54 (d, *J* = 8.4 Hz, 2H), 7.50 (d, *J* = 8.4 Hz, 1H), 7.45 – 7.38 (m, 2H), 7.38 – 7.36 (m, 1H), 7.36 – 7.29 (m, 2H), 7.14 (d, *J* = 8.4 Hz, 2H), 7.12 – 7.06 (m, 2H), 7.04 (d, *J* = 7.6 Hz, 2H), 6.99 (t, *J* = 7.4 Hz, 1H), 6.93 (t, *J* = 7.6 Hz, 1H), 6.90 – 6.86 (m, 1H), 6.83 (t, *J* = 7.4 Hz, 2H), 4.97 (s, 1H). **^{13}C NMR** (100 MHz, $CDCl_3$) δ 199.5, 141.7, 139.8, 137.7, 136.5, 136.3, 135.9, 135.1, 134.7, 134.5, 133.7, 133.6, 133.5, 132.9, 132.5, 132.0, 131.7, 129.6, 128.6, 128.2, 128.00, 127.97, 127.8, 127.7, 127.6, 127.1, 126.8, 126.6, 126.5, 126.4, 125.9, 123.8. **HRMS (ESI)** calcd for $C_{39}H_{27}ClO_2SiNa$ $[M+Na]^+$ 613.1367, found 613.1360.

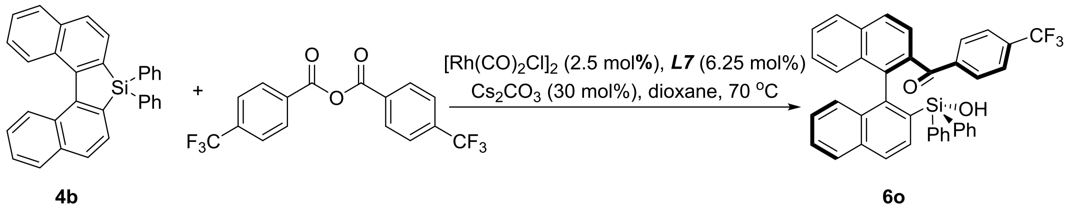

Compound **6o** was prepared following the **Typical Procedure F**

The reaction of **4b** (87 mg, 0.20 mmol) and 4-(trifluoromethyl)benzoic anhydride (109 mg, 0.30 mmol) with $[Rh(CO)_2Cl]_2$ (2.0 mg, 0.005 mmol, 2.5 mol%), **L7** (14.6 mg, 0.013 mmol, 6.25 mol%) afforded **6o** (109 mg, 87%, 87% ee). $[\alpha]_D^{20}$ – 61.55 (c 1.05, CH_2Cl_2). HPLC conditions: Chiralcel AD-H, isopropanol/hexane = 20:80, flow: 1.0 mL/min, λ = 254 nm. **^{1}H NMR** (500 MHz, $CDCl_3$) δ 7.90 (d, *J* = 8.0 Hz, 1H), 7.77 (d, *J* = 8.0 Hz, 1H), 7.75 – 7.72 (m, 2H), 7.72 – 7.68 (m, 2H), 7.61 (d, *J* = 8.5 Hz, 1H), 7.58 (d, *J* = 8.0 Hz, 2H), 7.54 (d, *J* = 8.5 Hz, 1H), 7.43 – 7.38 (m, 2H), 7.38 – 7.36 (m, 2H), 7.35 – 7.33 (m, 2H), 7.33 – 7.30 (m, 1H), 7.14 – 7.10 (m, 1H), 7.10 – 7.06 (m, 1H), 7.06 – 7.03 (m, 2H), 7.03 – 6.98 (m, 2H), 6.98 – 6.95 (m, 1H), 6.85 (t, *J* = 7.5 Hz, 2H), 4.33 (s, 1H). **^{13}C NMR** (126 MHz, $CDCl_3$) δ 200.0, 142.0, 139.4, 138.3, 136.3, 136.2, 135.7, 135.0, 134.5, 133.84 (q, *J* = 32.6 Hz) 133.75, 133.6, 132.8, 132.6, 132.0, 130.0, 129.7, 128.8, 128.2, 128.0, 127.9, 127.7, 127.3, 127.2, 126.9, 126.8, 126.6, 126.5, 126.0, 124.7 (q, *J* = 3.7 Hz), 124.0, 121.2 (q, *J* = 273.3 Hz). **^{19}F NMR** (471 MHz, $CDCl_3$) δ -63.2. **HRMS (ESI)** calcd for $C_{40}H_{27}F_3O_2SiNa$ $[M+Na]^+$ 647.1630, found 647.1639.

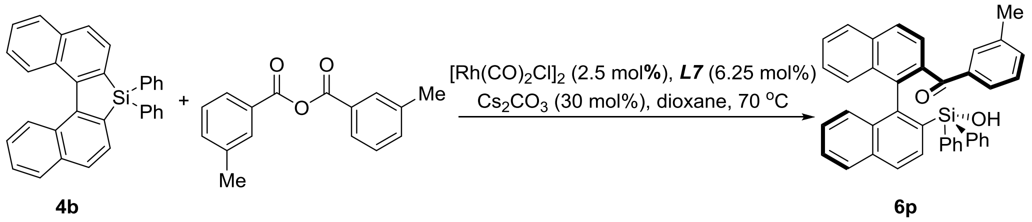

Compound **6p** was prepared following the **Typical Procedure F**

The reaction of **4b** (87 mg, 0.20 mmol) and 3-methylbenzoic anhydride (76 mg, 0.30 mmol) with $[Rh(CO)_2Cl]_2$ (2.0 mg, 0.005 mmol, 2.5 mol%), **L7** (14.6 mg, 0.013 mmol, 6.25 mol%) afforded **6p** (58 mg, 52%, 89% ee). $[\alpha]_D^{20}$ – 74.96 (c 1.10, CH_2Cl_2). HPLC conditions: Chiralcel AD-H, isopropanol/hexane = 20:80, flow: 1.0 mL/min, λ = 254 nm. **^{1}H NMR** (500 MHz, $CDCl_3$) δ 7.90 (d, *J* = 8.5 Hz, 1H), 7.84 – 7.82 (m, 1H), 7.82 – 7.80 (m, 1H), 7.75 (d, *J* = 8.0 Hz, 2H), 7.71 (d, *J* = 8.0 Hz, 1H), 7.64 (d, *J* = 8.0 Hz, 1H), 7.53 (d, *J* = 8.5 Hz, 1H), 7.46 – 7.43 (m, 2H), 7.43 – 7.42 (m, 2H), 7.42 – 7.39 (m, 1H), 7.34 – 7.30 (m, 1H), 7.30 – 7.27 (m, 1H), 7.17 (d, *J* = 7.5 Hz, 1H), 7.14 – 7.11 (m, 1H), 7.11 – 7.09 (m, 1H), 7.09 – 7.07 (m, 1H), 7.07 – 7.04 (m, 2H), 6.99 (t, *J* = 7.5 Hz, 1H), 6.93 – 6.87 (m, 1H), 6.86 – 6.83 (m, 2H), 6.83 – 6.81 (m, 1H), 5.47 (bs, 1H), 2.21 (s, 3H). **^{13}C NMR** (126 MHz, $CDCl_3$) δ 201.2, 142.0, 137.6, 137.2, 137.0, 136.8, 136.3, 136.0, 135.1, 134.5, 134.2, 133.7, 133.6, 133.4, 132.8, 132.5, 132.0, 131.0, 129.5, 128.5, 127.84, 127.83, 127.78, 127.7, 127.5, 127.0, 126.80, 126.77, 126.7, 126.4, 126.3, 125.5, 124.0, 21.1. **HRMS (ESI)** calcd for $C_{40}H_{30}O_2SiNa$ $[M+Na]^+$ 593.1913, found 593.1918.

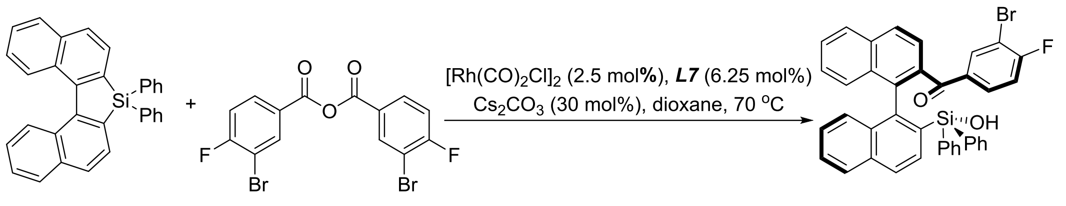

Compound **6q** was prepared following the **Typical Procedure F**

The reaction of **4b** (87 mg, 0.20 mmol) and **S4** (126 mg, 0.30 mmol) with $[Rh(CO)_2Cl]_2$ (2.0 mg, 0.005 mmol, 2.5 mol%), **L7** (14.6 mg, 0.013 mmol, 6.25 mol%) afforded **6q** (75 mg, 58%, 95% ee). $[\alpha]_D^{20}$ – 100.42 (c 0.94, CH_2Cl_2). HPLC conditions: Chiralcel AD-H, isopropanol/hexane = 20:80, flow: 1.0 mL/min, λ = 254 nm **^{1}H NMR** (500 MHz, $CDCl_3$) δ 7.92 (d, *J* = 8.5 Hz, 1H), 7.77 (d, *J* = 8.5 Hz, 1H), 7.76 – 7.73 (m, 2H), 7.73 – 7.72 (s, 2H), 7.72 – 7.70 (m, 1H), 7.61 (d, *J* = 8.0 Hz, 1H), 7.53 – 7.47 (m, 2H), 7.44 – 7.38 (m, 2H), 7.38 – 7.34 (m, 2H), 7.34 – 7.30 (m, 1H), 7.15 (t, *J* = 7.8 Hz, 1H), 7.07 – 7.04 (m, 1H), 7.04 – 7.01 (m, 2H), 6.99 (d, *J* = 7.5 Hz, 1H), 6.96 (t, *J* = 7.8 Hz, 1H), 6.91 (d, *J* = 8.5 Hz, 1H), 6.88 – 6.84 (m, 2H), 6.84 -6.80 (m, 1H), 4.64 (s, 1H). **^{13}C NMR** (126 MHz, $CDCl_3$) δ 198.4, 161.8 (d, $^1J_{C\text{-}F}$ = 255.1 Hz), 141.8, 137.8, 136.4, 136.1, 135.91, 135.90, 135.8, 135.0, 134.5, 133.99, 133.96, 133.6 (d, $^3J_{C\text{-}F}$ = 3.3 Hz), 132.8, 132.5, 132.0, 131.2, 131.1, 129.6, 128.7, 128.3, 128.0, 127.8, 127.7(d, $^4J_{C\text{-}F}$ = 3.0 Hz), 127.21, 127.19, 126.9, 126.8, 126.6, 126.5, 126.0, 123.5, 115.9 (d, $^2J_{C\text{-}F}$ = 23.2 Hz), 109.0 (d, $^2J_{C\text{-}F}$ = 21.9 Hz). **^{19}F NMR** (471 MHz, $CDCl_3$) δ - 98.8. **HRMS (ESI)** calcd for $C_{39}H_{26}BrFO_2SiNa$ $[M+Na]^+$ 675.0767, found 675.0760.

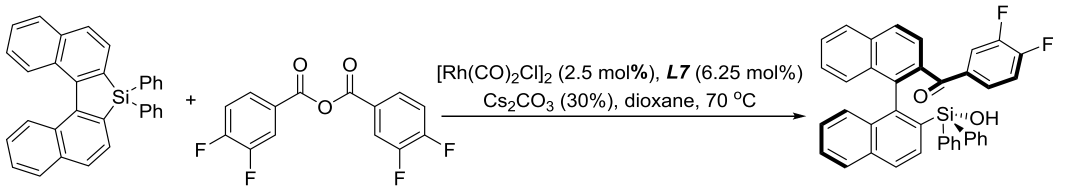

Compound **6r** was prepared following the **Typical Procedure F**

The reaction of **4b** (87 mg, 0.20 mmol) and **S5** (90 mg, 0.30 mmol) with $[Rh(CO)_2Cl]_2$ (2.0 mg, 0.005 mmol, 2.5 mol%), **L7** (14.6 mg, 0.013 mmol, 6.25 mol%) afforded **6r** (102 mg, 86%, 94% ee). $[\alpha]_D^{20}$ – 61.38 (c 0.99, CH_2Cl_2). HPLC conditions: Chiralcel AD-H, isopropanol/hexane = 20:80, flow: 1.0 mL/min, λ = 254 nm. **^{1}H NMR** (500 MHz, $CDCl_3$) δ 7.90 (d, *J* = 8.0 Hz, 1H), 7.78 – 7.75 (m, 1H), 7.75 – 7.73 (m, 2H), 7.73 – 7.68 (m, 2H), 7.61 (d, *J* = 8.5 Hz, 1H), 7.50 (d, *J* = 8.5 Hz, 1H), 7.43 – 7.39 (m, 2H), 7.39 – 7.36 (m, 2H), 7.36 – 7.33 (m, 2H), 7.33 – 7.30 (m, 1H), 7.14 – 7.09 (m, 1H), 7.07 – 7.04 (m, 2H), 7.04 – 7.02 (m, 1H), 7.02 – 6.98 (m, 1H), 6.98 – 6.93 (m, 1H), 6.93 – 6.88 (m, 2H), 6.84 (t, *J* = 7.5 Hz, 2H), 4.67 (s, 1H). **^{13}C NMR** (126 MHz, $CDCl_3$) δ 198.3, 153.6 (dd, $J_{C\text{-}F}$ = 258.8, 13.1 Hz), 149.7 (dd, $J_{C\text{-}F}$ = 251.6, 10.3 Hz), 141.8, 137.8, 136.3, 136.0, 135.8, 135.1, 134.5, 133.7, 133.63, 133.60, 133.4, 132.8, 132.5, 132.0, 129.6, 128.7, 128.2, 128.0, 127.8, 127.7, 127.6, 127.5 (dd, $J_{C\text{-}F}$ = 7.3, 3.3 Hz), 127.22, 127.16, 126.9, 126.8, 126.53, 126.49, 125.9, 123.6, 119.2 (dd, $J_{C\text{-}F}$ = 16.8 Hz), 116.8 (d, $^2J_{C\text{-}F}$ = 17.9 Hz). **^{19}F NMR** (471 MHz, $CDCl_3$) δ - 128.8 (d), - 136.2 (d). **HRMS (ESI)** calcd for $C_{39}H_{26}F_2O_2SiNa$ $[M+Na]^+$ 615.1568, found 615.1575.

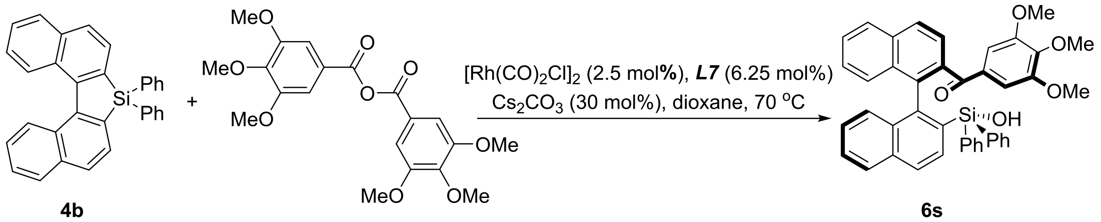

4b **6s**

Compound **6s** was prepared following the **Typical Procedure F**

The reaction of **4b** (87 mg, 0.20 mmol) and 3,4,5-trimethoxybenzoic anhydride (122 mg, 0.30 mmol) with $[Rh(CO)_2Cl]_2$ (2.0 mg, 0.005 mmol, 2.5 mol%), **L7** (14.6 mg, 0.013 mmol, 6.25 mol%) afforded **6s** (69 mg, 53%, 87% ee). $[\alpha]_D^{20}$ – 114.06 (c 1.10, CH_2Cl_2). HPLC conditions: Chiralcel AD-H, isopropanol/hexane = 20:80, flow: 1.0 mL/min, λ = 230 nm. **^{1}H NMR** (400 MHz, $CDCl_3$) δ 7.90 (d, J = 8.0 Hz, 1H), 7.81 – 7.77 (m, 2H), 7.77 – 7.75 (m, 2H), 7.75 – 7.73 (m, 1H), 7.64 (d, J = 8.4 Hz, 1H), 7.54 (d, J = 8.4 Hz, 1H), 7.45 – 7.39 (m, 2H), 7.49 – 7.35 (m, 1H), 7.39 – 7.29 (m, 2H), 7.12 – 7.07 (m, 2H), 7.07 – 7.02 (m, 2H), 7.02 – 6.97 (m, 1H), 6.93 – 6.88 (m, 2H), 6.88 – 6. 85 (m, 2H), 6.85 – 6.80 (m, 2H), 5.30 (s, 1H), 3.82 (s, 3H), 3.72 (s, 6H). **^{13}C NMR** (100 MHz, $CDCl_3$) δ 199.7, 152.3, 142.7, 142.0, 137.4, 136.7, 136.6, 135.9, 135.1, 134.5, 133.8, 133.6, 133.4, 132.8, 132.6, 132.0, 131.6, 129.5, 128.6, 128.0, 127.8, 127.73, 127.67, 127.6, 127.0, 126.9, 126.8, 126.5, 126.4, 125.7, 123.9, 108.1, 60.8, 56.0. **HRMS (ESI)** calcd for $C_{42}H_{35}O_5Si$ $[M+H]^+$ 647.2254, found 647.2249.

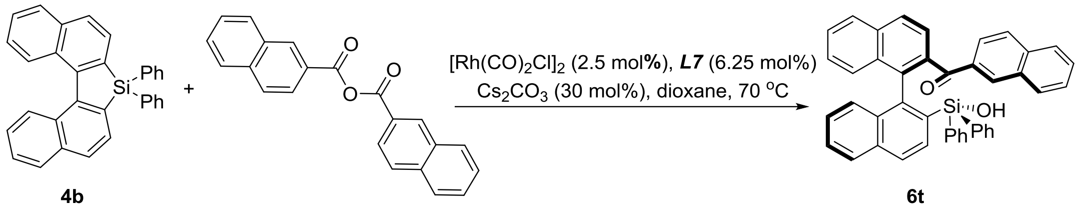

4b **6t**

Compound **6t** was prepared following the **Typical Procedure F**

The reaction of **4b** (87 mg, 0.20 mmol) and 2-naphthoic anhydride (98 mg, 0.30 mmol) with $[Rh(CO)_2Cl]_2$ (2.0 mg, 0.005 mmol, 2.5 mol%), **L7** (14.6 mg, 0.013 mmol, 6.25 mol%) afforded **6t** (108 mg, 89%, 90% ee). $[\alpha]_D^{20}$ – 260.01 (c 1.10, CH_2Cl_2). HPLC conditions: Chiralcel AD-H, isopropanol/hexane = 20:80, flow: 1.0 mL/min, λ = 254 nm. **^{1}H NMR** (400 MHz, $CDCl_3$) δ 8.11 (s, 1H), 7.93 (d, J = 8.4 Hz, 1H), 7.86 – 7.80 (m, 2H), 7.80 – 7.76 (m, 2H), 7.76 – 7.72 (m, 2H), 7.72 – 7.70 (m, 1H), 7.70 – 7.64 (m, 2H), 7.64 – 7.58 (m, 2H), 7.54 (t, J = 7.6 Hz, 1H), 7.49 – 7.44 (m, 1H), 7.44 – 7.41 (m, 2H), 7.41 – 7.37 (m, 1H), 7.35 (t, J = 7.6 Hz, 1H), 7.21 (d, J = 8.8 Hz, 1H), 7.16 (t, J = 7.6 Hz, 1H), 7.09 (d, J = 7.6 Hz, 2H), 7.01 (t, J = 8.2 Hz, 2H), 6.92 (t, J = 7.6 Hz, 1H), 6.89 – 6.87 (m, 1H), 6.87 – 6.80 (m, 2H), 5.47 (s, 1H). **^{13}C NMR** (100 MHz, $CDCl_3$) δ 200.7, 142.0, 137.5, 136.9, 136.7, 136.0, 135.6, 135.1, 134.5, 133.8, 133.7, 133.62, 133.60, 133.5, 132.9, 132.5, 132.0, 131.7, 129.6, 129.5, 128.8, 128.6, 128.0, 127.9, 127.83, 127.78, 127.7, 127.61, 127.56, 127.0, 126.9, 126.8, 126.6, 126.53, 126.50, 126.3, 125.7, 124.9, 124.1. **HRMS (ESI)** calcd for $C_{43}H_{30}O_2SiNa$ $[M+Na]^+$ 629.1913, found 629.1911.

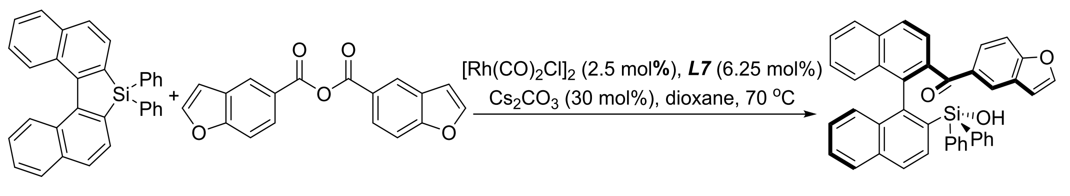

4b **S3** **6u**

Compound **6u** was prepared following the **Typical Procedure F**

The reaction of **4b** (87 mg, 0.20 mmol) and **S3** (92 mg, 0.30 mmol) with $[Rh(CO)_2Cl]_2$ (2.0 mg, 0.005 mmol, 2.5 mol%), **L7** (14.6 mg, 0.013 mmol, 6.25 mol%) afforded **6u** (75 mg, 63%, 90% ee). $[\alpha]_D^{20}$ – 156.59 (c 0.98, CH_2Cl_2). HPLC conditions: Chiralcel AD-H, isopropanol/hexane = 20:80, flow: 1.0 mL/min, λ = 254 nm. **^{1}H NMR** (500 MHz, $CDCl_3$) δ 7.91 (d, *J* = 10.5 Hz, 2H), 7.82 (d, *J* = 4.5 Hz, 2H), 7.75 (d, *J* = 9.0 Hz, 1H), 7.74 – 7.70 (m, 1H), 7.70 – 7.65 (m, 2H), 7.65 – 7.61 (m, 1H), 7.57 (d, *J* = 9.0 Hz, 2H), 7.47 – 7.37 (m, 3H), 7.32 (t, *J* = 9.0 Hz, 2H), 7.22 (t, *J* = 7.0 Hz, 1H), 7.15 (d, *J* = 8.5 Hz, 1H), 7.10 – 7.06 (m, 2H), 7.03 (d, *J* = 8.0 Hz, 1H), 6.99 (t, *J* = 7.0 Hz, 1H), 6.90 (t, *J* = 7.5 Hz, 1H), 6.88 – 6.85 (m, 1H), 6.85 – 6.80 (m, 2H), 6.66 (s, 1H), 5.64 (s, 1H). **^{13}C NMR** (126 MHz, $CDCl_3$) δ 200.4, 157.6, 146.3, 142.0, 137.2, 137.1, 136.8, 136.0, 135.2, 134.5, 133.7, 133.6, 133.4, 132.9, 132.4, 132.0, 131.7, 129.5, 128.5, 127.83, 127.79, 127.77, 127.7, 127.5, 127.04, 126.95, 126.9, 126.8, 126.6, 126.5, 126.3, 125.6, 125.5, 124.0, 111.0, 107.1. **HRMS (ESI)** calcd for $C_{41}H_{28}O_3SiNa$ $[M+Na]^+$ 619.1705, found 619.1706.

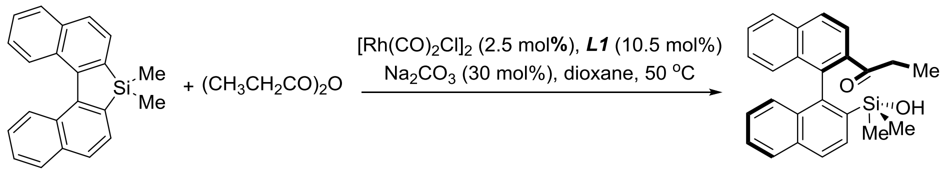

4a **6v**

Compound **6v** was prepared following the **Typical Procedure F**

The reaction of **4a** (62 mg, 0.20 mmol) and propionic anhydride (38 μL, 0.30 mmol) with $[Rh(CO)_2Cl]_2$ (2.0 mg, 0.005 mmol, 2.5 mol%), **L1** (18.9 mg, 0.021 mmol, 10.5 mol%) afforded **6v** (71 mg, 92%, 85% ee). $[\alpha]_D^{20}$ + 4.20 (c 0.98, CH_2Cl_2). HPLC conditions: Chiralcel AD-H, isopropanol/hexane = 8:92, flow: 1.0 mL/min, λ = 230 nm. **^{1}H NMR** (500 MHz, $CDCl_3$) δ 8.04 (d, *J* = 8.5 Hz, 1H), 7.97 (d, *J* = 8.0 Hz, 1H), 7.94 (d, *J* = 8.0 Hz, 1H), 7.91 (d, *J* = 8.0 Hz, 1H), 7.85 (d, *J* = 8.5 Hz, 1H), 7.69 (d, *J* = 8.5 Hz, 1H), 7.54 – 7.49 (m, 1H), 7.49 – 7.43 (m, 1H), 7.30 – 7.26 (m, 1H), 7.26 – 7.21 (m, 1H), 7.17 – 7.10 (m, 2H), 3.22 (s, 1H), 2.48 (dq, *J* = 18.5, 7.0 Hz, 1H), 2.55 – 2.40 (dq, *J* = 18.5, 7.0 Hz, 1H), 0.74 (t, *J* = 7.0 Hz, 3H), 0.22 (s, 3H), -0.53 (s, 3H). **^{13}C NMR** (126 MHz, $CDCl_3$) δ 209.3, 141.3, 138.7, 138.1, 136.5, 133.9, 133.7, 133.5, 132.6, 130.4, 128.6, 128.1, 128.0, 127.9, 127.4, 127.3, 127.0, 126.5, 126.4, 126.2, 123.4, 36.0, 7.9, 0.7, 0.6. **HRMS (ESI)** calcd for $C_{25}H_{24}O_2SiNa$ $[M+Na]^+$ 407.1443, found 407.1455.

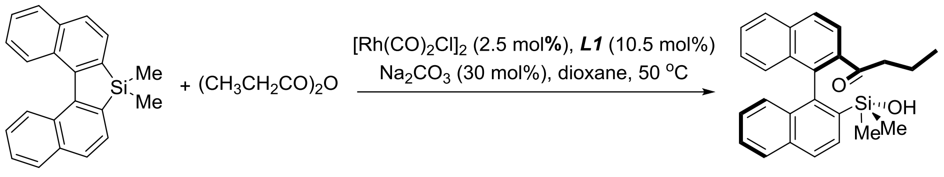

Compound **6w** was prepared following the **Typical Procedure F**

The reaction of **4a** (62 mg, 0.20 mmol) and butyric anhydride (38 μL, 0.30 mmol) with $[Rh(CO)_2Cl]_2$ (2.0 mg, 0.005 mmol, 2.5 mol%), **L1** (18.9 mg, 0.021 mmol, 10.5 mol%) afforded **6w** (43 mg, 53%, 90% ee). $[\alpha]_D^{20}$ + 0.29 (c 1.13, CH_2Cl_2). HPLC conditions: Chiralcel AD-H, isopropanol/hexane = 4:96, flow: 1.0 mL/min, λ = 254 nm. **^{1}H NMR** (400 MHz, $CDCl_3$) δ 8.03 (d, *J* = 8.4 Hz, 1H), 7.95 (t, *J* = 7.4 Hz, 2H), 7.90 (d, *J* = 8.0 Hz, 1H), 7.83 (d, *J* = 8.4 Hz, 1H), 7.63 (d, *J* = 8.4 Hz, 1H), 7.55 – 7.49 (m, 1H), 7.49 – 7.42 (m, 1H), 7.32 – 7.27 (m, 1H), 7.25 – 7.21 (m, 1H), 7.17 (d, *J* = 8.0 Hz, 1H), 7.12 (d, *J* = 8.4 Hz, 1H), 3.29 (s, 1H), 2.48 – 2.32 (m, 1H), 2.28 – 2.09 (m, 1H), 1.30 – 1.20 (m, 2H), 0.41 (t, *J* = 7.4 Hz, 3H), 0.24 (s, 3H), -0.56 (s, 3H). **^{13}C NMR** (100 MHz, $CDCl_3$) δ 209.3, 141.1, 139.2, 138.3, 136.2, 133.80, 133.76, 133.5, 132.6, 130.4, 128.6, 128.1, 128.0, 127.8, 127.4, 127.3, 127.0, 126.5, 126.34, 126.30, 123.2, 44.7, 16.9, 13.1, 0.68, 0.65. **HRMS (ESI)** calcd for $C_{26}H_{26}O_2SiNa$ $[M+Na]^+$ 421.1600, found 407.1606.

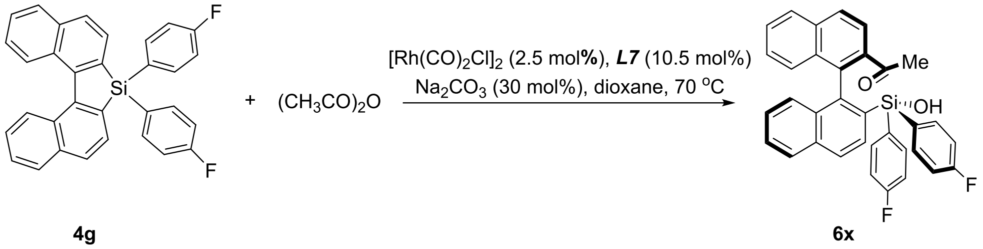

Compound **6x** was prepared following the **Typical Procedure F**

The reaction of **4g** (94 mg, 0.20 mmol) and acetic anhydride (28 μL, 0.30 mmol) with $[Rh(CO)_2Cl]_2$ (2.0 mg, 0.005 mmol, 2.5 mol%), **L7** (24.5 mg, 0.021 mmol, 10.5 mol%) afforded **6x** (99 mg, 96%, 91% ee). $[\alpha]_D^{20}$ + 24.07 (c 0.97, CH_2Cl_2). HPLC conditions: Chiralcel AD-H, isopropanol/hexane = 20:80, flow: 1.0 mL/min, λ = 254 nm. **^{1}H NMR** (400 MHz, $CDCl_3$) δ 7.90 (d, *J* = 8.0, 1H), 7.89 (d, *J* = 8.4, 1H), 7.86 (d, *J* = 8.8 Hz, 1H), 7.72 (d, *J* = 8.0 Hz, 1H), 7.66 – 7.61 (m, 2H), 7.61 – 7.56 (m, 2H), 7.50 – 7.44 (m, 1H), 7.40 – 7.32 (m, 1H), 7.25 – 7.19 (m, 1H), 7.09 – 7.05 (m, 2H), 7.04 – 7.02 (m, 1H), 7.02 – 6.96 (m, 2H), 6.96 – 6.93 (m, 1H), 6.83 (d, *J* = 8.4 Hz, 1H), 6.56 – 6.47 (m, 2H), 4.12 (s, 1H), 2.06 (s, 3H). **^{13}C NMR** (100 MHz, $CDCl_3$) δ 207.0, 164.1 (d, $^1J_{C-F}$ = 247.6 Hz), 163.4 (d, $^1J_{C-F}$ = 247.6 Hz), 143.0, 138.3, 136.9 (d, $^3J_{C-F}$ = 7.6 Hz), 135.9, 135.4 (d, $^3J_{C-F}$ = 7.6 Hz), 134.6, 134.0, 133.7, 132.7, 131.9, 131.4 (d, $^4J_{C-F}$ = 3.8 Hz), 129.8 (d, $^4J_{C-F}$ = 3.6 Hz), 128.9, 128.2, 127.8, 127.6, 127.3, 127.1, 127.0, 126.7, 126.6, 126.1, 122.7, 115.0 (d, $^2J_{C-F}$ = 19.6 Hz), 114.1 (d, $^2J_{C-F}$ = 19.8 Hz), 30.6. **^{19}F NMR** (376 MHz, $CDCl_3$) δ - 110.7, - 111.8. **HRMS (ESI)** calcd for $C_{34}H_{24}F_2O_2SiNa$ $[M+Na]^+$ 553.1411, found 553.1414.

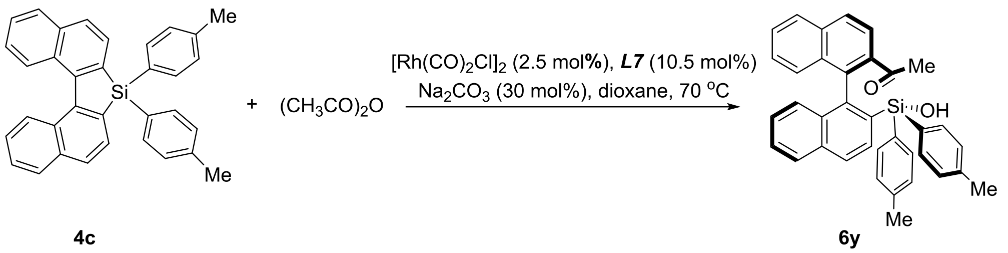

4c **6y**

Compound **6y** was prepared following the **Typical Procedure F**

The reaction of **4c** (93 mg, 0.20 mmol) and acetic anhydride (28 µL, 0.30 mmol) with $[Rh(CO)_2Cl]_2$ (2.0 mg, 0.005 mmol, 2.5 mol%), **L7** (24.5 mg, 0.021 mmol, 10.5 mol%) afforded **6y** (81 mg, 78%, 92% ee). $[\alpha]_D^{20}$ + 4.23 (c 1.07, CH_2Cl_2). HPLC conditions: Chiralcel 0AD-H, isopropanol/hexane = 20:80, flow: 1.0 mL/min, λ = 254 nm. **^{1}H NMR** (400 MHz, $CDCl_3$) δ 7.92 – 7.88 (m, 2H), 7.88 – 7.83 (m, 1H), 7.72 (d, *J* = 8.4 Hz, 2H), 7.67 (dd, *J* = 8.4 Hz, 1.4 Hz, 1H), 7.51 (d, *J* = 6.8 Hz, 2H), 7.49 – 7.43 (m, 1H), 7.38 – 7.32 (m, 1H), 7.25 – 7.19 (m, 1H), 7.16 (d, *J* = 7.6 Hz, 2H), 7.07 (d, *J* = 8.8 Hz, 1H), 7.00 – 6.95 (m, 1H), 6.95 – 6.92 (m, 2H), 6.92 – 6.87 (m, 1H), 6.69 (d, *J* = 7.2 Hz, 2H), 3.33 (s, 1H), 2.36 (s, 3H), 2.17 (s, 3H), 1.98 (s, 3H). **^{13}C NMR** (100 MHz, $CDCl_3$) δ 205.6, 143.1, 139.5, 138.5, 138.0 ,136.7, 135.3, 134.9, 133.8, 133.7, 132.93, 132.87, 132.29, 132.26, 131.0, 128.6, 128.5, 128.14, 128.10, 127.8, 127.5, 127.0, 126.9, 126.7, 126.5, 126.4, 126.2, 123.3, 30.4, 21.6, 21.4. **HRMS (ESI)** calcd for $C_{36}H_{30}O_2SiNa$ $[M+Na]^+$ 545.1913, found 545.1921.

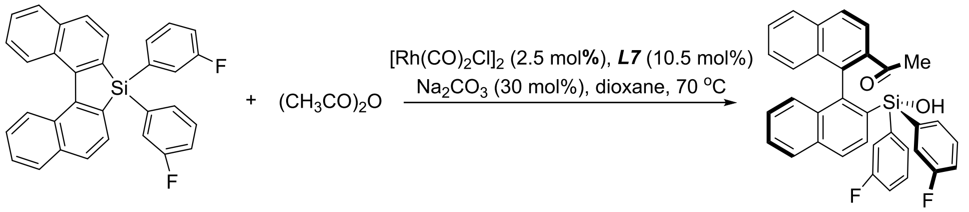

4h **6z**

Compound **6z** was prepared following the **Typical Procedure F**

The reaction of **4h** (94 mg, 0.20 mmol) and acetic anhydride (28 µL, 0.30 mmol) with $[Rh(CO)_2Cl]_2$ (2.0 mg, 0.005 mmol, 2.5 mol%), **L7** (24.5 mg, 0.021 mmol, 10.5 mol%) afforded **6z** (100 mg, 94%, 94% ee). $[\alpha]_D^{20}$ + 20.87 (c 0.99, CH_2Cl_2). HPLC conditions: Chiralcel AD-H, isopropanol/hexane = 20:80, flow: 1.0 mL/min, λ = 254 nm. **^{1}H NMR** (400 MHz, $CDCl_3$) δ 7.90 (dd, *J* = 8.6, 2.4 Hz, 2H), 7.86 (d, *J* = 8.8 Hz, 1H), 7.69 (d, *J* = 8.2 Hz, 1H), 7.62 (d, *J* = 5.6 Hz, 1H), 7.60 (d, *J* = 5.6 Hz, 1H), 7.51 – 7.46 (m, 1H), 7.46 – 7.41 (m, 1H), 7.39 – 7.35 (m, 1H), 7.35 – 7.30 (m, 2H), 7.25 – 7.20 (m, 1H), 7.13 – 7.08 (m, 1H), 7.08 – 7.04 (m, 1H), 7.04 -6.98 (m, 1H), 6.84 (d, *J* = 8.4 Hz, 1H), 6.81 – 6.74 (m, 2H), 6.65 – 6.57 (m, 2H), 4.42 (s, 1H), 2.08 (s, 3H). **^{13}C NMR** (100 MHz, $CDCl_3$) δ 207.4, 162.5 (d, $^1J_{C-F}$ = 246.9 Hz), 161.6 (d, $^1J_{C-F}$ = 246.1 Hz) 138.5 (d, $^3J_{C-F}$ = 4.3 Hz), 138.4, 136.8 (d, $^3J_{C-F}$ = 4.3 Hz), 135.7, 134.1, 133.6, 132.7, 132.7, 131.9, 130.4 (d, $^4J_{C-F}$ = 2.9 Hz), 129.74, 129.68, 129.02, 128.97 (d, $^4J_{C-F}$ = 2.9 Hz), 128.8, 128.7, 128.3, 127.7, 127.6, 127.5, 127.2, 127.0, 126.72, 126.68, 126.1, 122.6, 121.2 (d, $^2J_{C-F}$ = 19 Hz), 119.6 (d, $^2J_{C-F}$ = 19.2 Hz), 116.8 (d, $^2J_{C-F}$ = 20.8 Hz), 115.9 (d, $^2J_{C-F}$ = 21 Hz), 30.7. **^{19}F NMR** (376 MHz, $CDCl_3$) δ - 113.2, - 113.8. **HRMS (ESI)** calcd for $C_{34}H_{24}F_2O_2SiNa$ $[M+Na]^+$ 553.1411, found 553.1411.

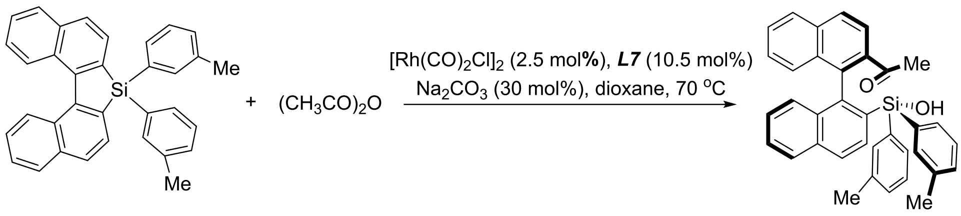

4f **6aa**

Compound **6aa** was prepared following the **Typical Procedure F**

The reaction of **4f** (92 mg, 0.20 mmol) and acetic anhydride (28 µL, 0.30 mmol) with $[Rh(CO)_2Cl]_2$ (2.0 mg, 0.005 mmol, 2.5 mol%), **L7** (24.5 mg, 0.021 mmol, 10.5 mol%) afforded **6aa** (101 mg, 96%, 92% ee). $[\alpha]_D^{20}$ + 20.99 (c 1.05, CH_2Cl_2). HPLC conditions: Chiralcel AD-H, isopropanol/hexane = 20:80, flow: 1.0 mL/min, λ = 254 nm. **^{1}H NMR** (400 MHz, $CDCl_3$) δ 7.92 – 7.87 (m, 2H), 7.85 (d, *J* = 8.4 Hz, 1H), 7.70 (d, *J* = 8.4 Hz, 2H), 7.65 (d, *J* = 8.4 Hz, 1H), 7.49 – 7.44 (m, 1H), 7.44 – 7.39 (m, 2H), 7.34 – 7.29 (m, 1H), 7.24 – 7.20 (m, 2H), 7.20 – 7.15 (m, 1H), 7.05 (d, *J* = 8.4 Hz, 1H), 7.00 – 6.94 (m, 1H), 6.92 – 6.86 (m, 2H), 6.83 – 6.78 (m, 2H), 6.78 – 6.75 (m, 1H), 3.53 (s, 1H), 2.32 (s, 3H), 2.03 (s, 3H), 1.99 (s, 3H). **^{13}C NMR** (100 MHz, $CDCl_3$) δ 205.8, 143.2, 138.0, 137.0, 136.6, 136.1, 135.6, 135.4, 135.2, 134.2, 134.1, 133.9, 133.8, 132.9, 132.8, 132.3, 132.0, 130.9, 130.4, 129.7, 128.7, 128.2, 127.9, 127.6, 127.4, 127.10, 127.08, 126.9, 126.7, 126.43, 126.39, 126.2, 123.1, 30.5, 21.6, 21.4. **HRMS (ESI)** calcd for $C_{36}H_{30}O_2SiNa$ $[M+Na]^+$ 545.1913, found 545.1913.

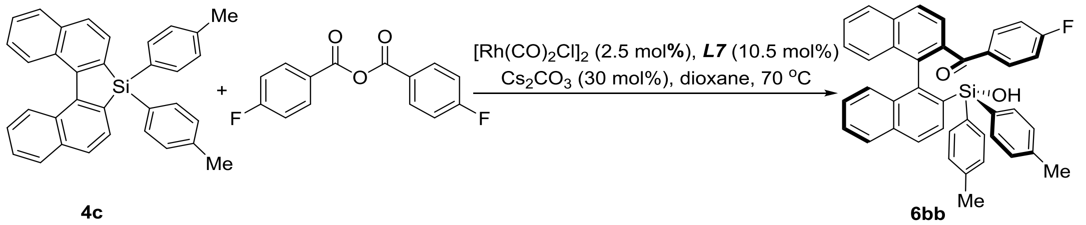

4c **6bb**

Compound **6bb** was prepared following the **Typical Procedure F**

The reaction of **4c** (92 mg, 0.20 mmol) and 4-fluorobenzoic anhydride (79 mg, 0.30 mmol) with $[Rh(CO)_2Cl]_2$ (2.0 mg, 0.005 mmol, 2.5 mol%), **L7** (24.5 mg, 0.021 mmol, 10.5 mol%) afforded **6bb** (78 mg, 65%, 91% ee). $[\alpha]_D^{20}$ – 65.65 (c 1.10, CH_2Cl_2). HPLC conditions: Chiralcel AD-H, isopropanol/hexane = 20:80, flow: 1.0 mL/min, λ = 254 nm. **^{1}H NMR** (500 MHz, $CDCl_3$) δ 7.89 (d, *J* = 8.5 Hz, 1H), 7.76 – 7.74 (m, 1H), 7.74 – 7.71 (m, 2H), 7.67 – 7.64 (m, 2H), 7.64 – 7.63 (m, 1H), 7.63 – 7.62 (m, 1H), 7.62 – 7.59 (m, 1H), 7.50 (d, *J* = 8.5 Hz, 1H), 7.37 – 7.33 (m, 1H), 7.33 – 7.29 (m, 1H), 7.19 (d, *J* = 7.5 Hz, 2H), 7.11 – 7.02 (m, 2H), 6.96 – 6.92 (m, 1H), 6.92 – 6.90 (m, 2H), 6.88 – 6.85 (m, 1H), 6.85 – 6.80 (m, 2H), 6.62 (d, *J* = 7.5 Hz, 2H), 4.99 (s, 1H), 2.37 (s, 3H), 2.15 (s, 3H). **^{13}C NMR** (126 MHz, $CDCl_3$) δ 199.0, 165.7 (d, $^1J_{C-F}$ = 254.6 Hz), 141.8, 139.4, 138.1, 137.7, 136.4 (d, $^3J_{C-F}$ = 10.8 Hz), 135.2, 133.7, 133.6, 133.5, 133.1, 133.01, 133.00, 132.97, 132.80, 132.77, 132.5, 132.1, 131.2, 128.5, 127.9 (d, $^4J_{C-F}$ = 3.5 Hz), 127.8, 127.7, 127.5, 127.0, 126.7, 126.6, 126.5, 126.3, 125.7, 123.9, 115.0 (d, $^2J_{C-F}$ = 22.1 Hz), 21.5, 21.3. **^{19}F NMR** (471 MHz, $CDCl_3$) δ -104.2. **HRMS (ESI)** calcd for $C_{41}H_{31}FO_2SiNa$ $[M+Na]^+$ 625.1975, found 625.1960.

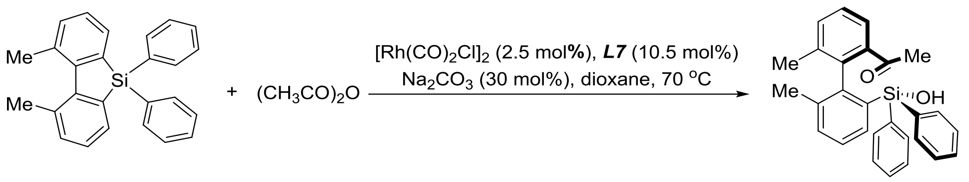

4d **6cc**

Compound **6cc** was prepared following the **Typical Procedure F**

The reaction of **4d** (73 mg, 0.20 mmol) and acetic anhydride (28 μL, 0.30 mmol) with $[Rh(CO)_2Cl]_2$ (2.0 mg, 0.005 mmol, 2.5 mol%), **L7** (24.5 mg, 0.021 mmol, 10.5 mol%) afforded **6cc** (70 mg, 83%, 88% ee). $[\alpha]_D^{20}$ + 14.03 (c 1.00, CH_2Cl_2). HPLC conditions: Chiralcel AD-H, isopropanol/hexane = 20:80, flow: 1.0 mL/min, λ = 254 nm. **^{1}H NMR** (400 MHz, $CDCl_3$) δ 7.61 – 7.57 (m, 2H), 7.45 – 7.41 (m, 1H), 7.40 – 7.37 (m, 1H), 7.37 – 7.34 (m, 2H), 7.34 – 7.33 (m, 2H), 7.33 – 7.31 (m, 2H), 7.31 – 7.30 (m, 1H), 7.30 – 7.27 (m, 1H), 7.25 – 7.22 (m, 2H), 7.22 – 7.19 (m, 1H), 7.10 (d, *J* = 8.4 Hz, 1H), 3.21 (s, 1H), 2.09 (s, 3H), 1.92 (s, 3H), 1.51 (s, 3H). **^{13}C NMR** (100 MHz, $CDCl_3$) δ 205.0, 145.0, 139.6, 138.8, 138.1, 136.2, 135.7, 135.4, 135.0, 134.7, 134.54, 134.49, 132.8, 131.7, 129.6, 129.3, 127.7, 127.6, 127.4, 126.7, 124.9, 29.7, 19.9, 19.6. **HRMS (ESI)** calcd for $C_{28}H_{26}FO_2SiNa$ $[M+Na]^+$ 445.1600, found 445.1602.

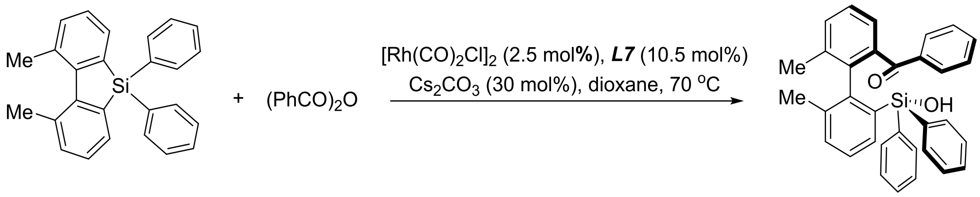

4d **6dd**

Compound **6dd** was prepared following the **Typical Procedure F**

The reaction of **4d** (73 mg, 0.20 mmol) and benzoic anhydride (68 mg, 0.30 mmol) with $[Rh(CO)_2Cl]_2$ (2.0 mg, 0.005 mmol, 2.5 mol%), **L7** (24.5 mg, 0.021 mmol, 10.5 mol%) afforded **6dd** (68 mg, 70%, 85% ee). $[\alpha]_D^{20}$ – 72.17 (c 1.00, CH_2Cl_2). HPLC conditions: Chiralcel AD-H, isopropanol/hexane = 20:80, flow: 1.0 mL/min, λ = 254 nm. **^{1}H NMR** (500 MHz, $CDCl_3$) δ 7.81 – 7.77 (m, 2H), 7.75 (d, *J* = 7.5 Hz, 2H), 7.55 (t, *J* = 7.5 Hz, 1H), 7.42 – 7.39 (m, 2H), 7.39 – 7.38 (m, 2H), 7.38 – 7.37 (m, 2H), 7.37 – 7.35 (m, 1H), 7.35 – 7.33 (m, 1H), 7.32 – 7.28 (m, 1H), 7.28 – 7.24 (m, 2H), 7.21 (t, *J* = 7.5 Hz, 2H), 7.10 (t, *J* = 7.5 Hz, 1H), 7.09 – 7.05 (m, 1H), 7.04 – 6.99 (m, 1H), 5.41 (s, 1H), 1.75 (s, 3H), 1.34 (s, 3H). **^{13}C NMR** (126 MHz, $CDCl_3$) δ 200.5, 143.7, 139.6, 138.4, 137.8, 137.1, 136.5, 136.4, 135.6, 135.24, 135.17, 134.5, 133.6, 132.2, 131.6, 130.8, 129.4, 129.1, 128.2, 127.6, 127.2, 126.7, 126.5, 125.7, 20.1, 19.5. **HRMS (ESI)** calcd for $C_{33}H_{28}O_2SiNa$ $[M+Na]^+$507.1756, found 507.1763.

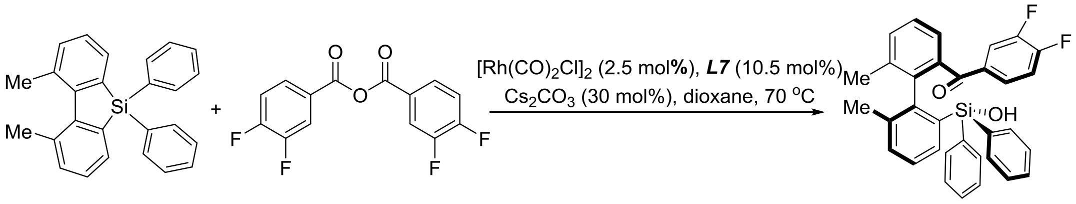

4d **S5** **6ee**

Compound **6ee** was prepared following the **Typical Procedure F**

The reaction of **4d** (73 mg, 0.20 mmol) and **S5** (89 mg, 0.30 mmol) with $[Rh(CO)_2Cl]_2$ (2.0 mg, 0.005 mmol, 2.5 mol%), **L7** (24.5 mg, 0.021 mmol, 10.5 mol%) afforded **6ee** (69 mg, 66%, 88% ee). $[\alpha]_D^{20}$ – 50.35 (c 0.90, CH_2Cl_2). HPLC conditions: Chiralcel AD-H, isopropanol/hexane = 20:80, flow: 1.0 mL/min, λ = 254 nm. **^{1}H NMR** (400 MHz, $CDCl_3$) δ 7.62 (d, *J* = 6.4 Hz, 2H), 7.53 – 7.45 (m, 1H), 7.43 – 7.37 (m, 1H), 7.33 – 7.28 (m, 2H), 7.28 – 7.26 (m, 2H), 7.26 – 7.24 (m, 2H), 7.24 – 7.19 (m, 2H), 7.18 – 7.16 (m, 1H), 7.16 – 7.10 (m, 2H), 7.10 – 7.05 (m, 2H), 7.05 – 7.02 (m, 1H), 7.01 (d, *J* = 7.6 Hz, 1H), 4.60 (s, 1H), 1.70 (s, 3H), 1.34 (s, 3H). **^{13}C NMR** (100 MHz, $CDCl_3$) δ 197.4, 153.9 (dd, $J_{C\text{-}F}$ = 256.9, 12.8 Hz), 150.0 (dd, $J_{C\text{-}F}$ = 249.7, 12.9 Hz), 143.5, 139.9, 138.7, 136.9, 136.7, 135.9, 135.4, 135.3, 135.1, 134.6, 134.5, 133.69, 133.65, 133.6, 132.7, 131.6, 129.5, 129.2, 128.0 (d, $J_{C\text{-}F}$ = 3.6 Hz), 127.9 (d, $J_{C\text{-}F}$ = 3.1 Hz), 127.6, 127.3, 126.8, 126.7, 125.6, 119.5 (d, $J_{C\text{-}F}$ = 18.0 Hz), 117.1 (d, $J_{C\text{-}F}$ = 17.6 Hz), 20.1, 19.5. **^{19}F NMR** (376 MHz, $CDCl_3$) δ - 128.5, - 135.8. **HRMS (ESI)** calcd for $C_{33}H_{26}F_2O_2SiNa$ $[M+Na]^+$ 543.1568, found 543.1579.

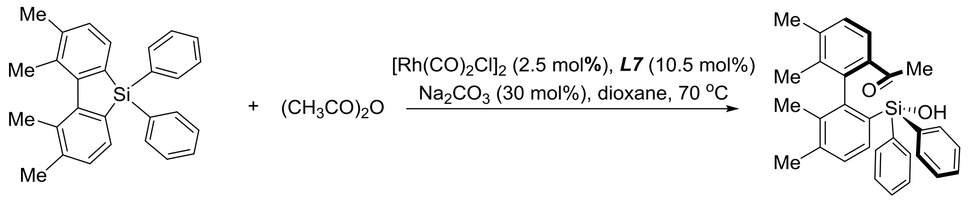

4e **6ff**

Compound **6E** was prepared following the **Typical Procedure F**

The reaction of **4e** (78 mg, 0.20 mmol) and acetic anhydride (28 µL, 0.30 mmol) with $[Rh(CO)_2Cl]_2$ (2.0 mg, 0.005 mmol, 2.5 mol%), **L7** (24.5 mg, 0.021 mmol, 10.5 mol%) afforded **6ff** (40 mg, 45%, 87% ee). $[\alpha]_D^{20}$ + 35.74 (c 0.77, CH_2Cl_2). HPLC conditions: Chiralcel AD-H, isopropanol/hexane = 20:80, flow: 1.0 mL/min, λ = 230 nm. **^{1}H NMR** (400 MHz, $CDCl_3$) δ 7.61 (d, *J* = 7.2 Hz, 2H), 7.42 – 7.37 (m 1H), 7.36 – 7.34 (m, 1H), 7.34 – 7.31 (m, 2H), 7.31 – 7.27 (m, 2H), 7.27 – 7.26 (m, 1H), 7.22 (d, *J* = 8.0 Hz, 2H), 7.18 (d, *J* = 7.2 Hz, 1H), 7.14 (d, *J* = 7.6 Hz, 1H), 7.09 (d, *J* = 7.6 Hz, 1H), 3.42 (s, 1H), 2.30 (s, 3H), 2.06 (s, 3H), 2.04 (s, 3H), 1.82 (s, 3H), 1.27 (s, 3H). **^{13}C NMR** (100 MHz, $CDCl_3$) δ 205.7, 145.5, 140.6, 139.0, 138.8, 138.1, 136.7, 136.4, 135.8, 135.0, 134.6, 134.4, 134.3, 132.4, 129.4, 129.1, 128.9, 128.3, 127.6, 127.2, 124.5, 29.9, 20.8, 20.7, 16.1, 16.0. **HRMS (ESI)** calcd for $C_{30}H_{30}O_2SiNa$ $[M+Na]^+$ 473.1913, found 473.1918.

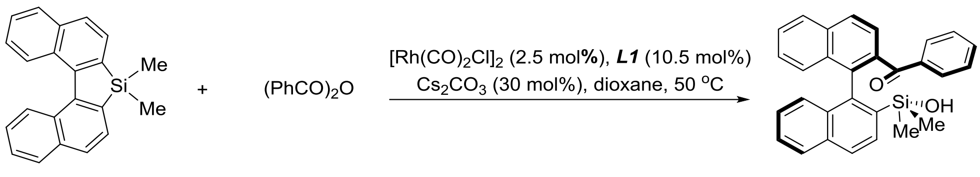

4a **S6**

Compound **S6** was prepared following the **Typical Procedure F**

The reaction of **4a** (62 mg, 0.20 mmol) and benzoic anhydride (68 mg, 0.30 mmol) with $[Rh(CO)_2Cl]_2$ (2.0 mg, 0.005 mmol, 2.5 mol%), **L1** (18.9 mg, 0.021 mmol, 10.5 mol%) afforded **S6** (43 mg, 54%, 75% ee). $[\alpha]_D^{20}$ + 64.22 (c 1.07, CH_2Cl_2). HPLC conditions: Chiralcel AD-H,

isopropanol/hexane = 20:80, flow: 1.0 mL/min, λ = 254 nm. **^{1}H NMR** (400 MHz, $CDCl_3$) δ 8.05 (d, *J* = 8.4 Hz, 1H), 7.99 (d, *J* = 8.0 Hz, 1H), 7.83 (d, *J* = 8.0 Hz, 1H), 7.79 (d, *J* = 8.0 Hz, 1H), 7.72 (d, *J* = 8.4 Hz, 1H), 7.61 – 7.60 (m, 1H), 7.60 – 7.59 (m, 1H), 7.58 – 7.57 (m, 1H), 7.56 – 7.52 (m, 1H), 7.38 – 7.34 (m, 1H), 7.34 – 7.31 (m, 1H), 7.31 – 7.28 (m, 1H), 7.23 – 7.20 (m, 1H), 7.20 – 7.16 (m, 2H), 7.16 – 7.12 (m, 1H), 7.11 – 7.05 (m, 1H), 4.24 (s, 1H), 0.40 (s, 3H), -0.59 (s, 3H). **^{13}C NMR** (100 MHz, $CDCl_3$) δ 200.1, 140.3, 138.7, 138.2, 136.9, 136.4, 133.8, 133.6, 133.2, 132.2, 130.3, 130.1, 128.1, 127.9, 127.8, 127.7, 127.4, 127.3, 127.0, 126.6, 126.1, 125.7, 124.7, 0.83, 0.75. **HRMS (ESI)** calcd for $C_{29}H_{24}O_2SiNa$ $[M+Na]^+$ 455.1443, found 455.1440.

Control Experiments

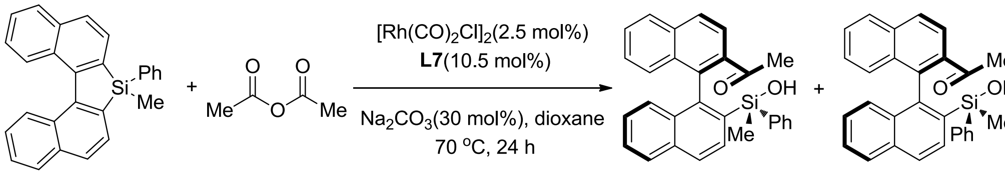

4i **6gg**, 90% ee **6gg'**, 92% ee

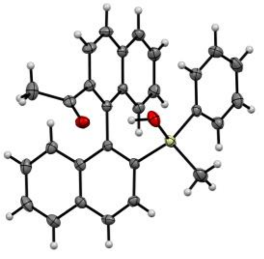

structure of **6gg**

Under nitrogen atmosphere, to a Schleck tube was added $[Rh(CO)_2Cl]_2$ (2.0 mg, 0.005 mmol, 2.5 mol%), **L7** (24.5 mg, 0.021 mmol, 10.5 mol%) and dioxane (2 mL) at room temperature and was stirred for 30 minutes. The solution was transfer via cannula carefully to another Schlenk tube charged with **4i** (75 mg, 0.200 mmol, 1.0 equiv), acetic anhydride (28 μL, 0.300 mmol, 1.5 equiv), Na_2CO_3 (6.4 mg, 0.060 mmol, 30 mol%) and dioxane (2 mL). The tube was capped with a screw cap and stirred at 70 °C for 24 h. After being cooled to room temperature, the mixture was filtered through Celite and the filtrate was concentrated in vacuum and purified by flash column chromatography (PE/EtOAc = 90:10) on silica gel to afford a mixture of **6gg** and **6gg'** (85 mg, 99%). A small amount of **6gg** and **6gg'** could be separated by preparative TLC, and the related configuration was determined by single crystal X-ray diffraction analysis. The ee value was determined by the mixture of **6gg** and **6gg'**.

6gg: $[\alpha]_D^{20}$ + 7.17 (c 1.06, CH_2Cl_2). HPLC conditions: Chiralcel AD-H, isopropanol/hexane = 10:90, flow: 1.0 mL/min, λ = 254 nm. **^{1}H NMR** (500 MHz, $CDCl_3$) δ 8.00 (d, *J* = 8.0 Hz, 1H), 7.95 (d, *J* = 8.5 Hz, 1H), 7.93 – 7.87 (m, 2H), 7.74 (d, *J* = 8.0 Hz, 1H), 7.69 (d, *J* = 9.0 Hz, 1H), 7.48 – 7.43 (m, 1H), 7.36 – 7.30 (m, 1H), 7.23 – 7.18 (m, 1H), 7.04 – 7.00 (m, 1H), 7.00 – 6.97 (m, 1H), 6.97 – 6.94 (m, 1H), 6.92 – 6.88 (m, 2H), 6.88 – 6.86 (m, 1H), 6.86 – 6.84 (m, 1H), 6.84 – 6.80 (m, 1H), 3.33 (s, 1H), 1.92 (s, 3H), 0.40 (s, 3H). **^{13}C NMR** (126 MHz, $CDCl_3$) δ 205.6, 142.4, 138.0, 136.74, 136.69, 136.2, 133.93, 133.89, 133.1, 132.82, 132.79, 130.9, 128.70, 128.69, 128.1, 127.9, 127.6, 127.5, 127.2,

127.0, 126.7, 126.6, 126.5, 126.1, 123.4, 30.4, 0.5. **HRMS (ESI)** calcd for $C_{29}H_{24}O_2SiNa$ $[M+Na]^+$ 455.1443, found 455.1440.

6gg': $[\alpha]_D^{20}$ + 55.71 (c 0.42, CH_2Cl_2). HPLC conditions: Chiralcel AD-H, isopropanol/hexane = 10:90, flow: 1.0 mL/min, λ = 254 nm. **^{1}H NMR** (500 MHz, $CDCl_3$) δ 8.03 (d, *J* = 8.5 Hz, 1H), 7.93 (d, *J* = 8.0 Hz, 1H), 7.88 (d, *J* = 8.5 Hz, 1H), 7.85 (d, *J* = 8.5 Hz, 1H), 7.77 (d, *J* = 8.5 Hz, 1H), 7.58 (d, *J* = 8.0 Hz, 1H), 7.54 – 7.49 (m, 1H), 7.48 – 7.45 (m, 1H), 7.45 – 7.41 (m, 2H), 7.37 – 7.32 (m, 1H), 7.32 – 7.27 (m, 2H), 7.27 – 7.26 (m, 1H), 7.25 – 7.20 (m, 1H), 7.16 (d, *J* = 8.5 Hz, 1H), 7.11 (d, *J* = 8.5 Hz, 1H), 3.28 (s, 1H), 1.98 (s, 3H), -0.27 (s, 3H). **^{13}C NMR** (126 MHz, $CDCl_3$) δ 205.4, 142.3, 138.3, 137.7, 137.0, 136.5, 134.1, 133.8, 133.5, 132.7, 131.6, 129.4, 128.7, 128.2, 128.00, 127.98, 127.7, 127.6, 127.3, 127.0, 126.7, 126.5, 126.1, 123.7, 30.4, 0.7. **HRMS (ESI)** calcd for $C_{29}H_{24}O_2SiNa$ $[M+Na]^+$ 455.1443, found 455.1440.

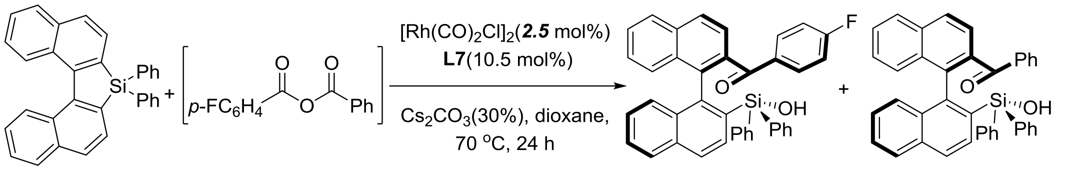

6m, 93% ee **6b**, 91% ee

Under nitrogen atmosphere, a Schlenk tube was sequentially charged with Cs_2CO_3 (3.0 equiv), benzoic acid (18.0 mg, 0.150 mmol, 1.5 equiv), 4-fluorobenzoyl chloride (18 μL, 0.150 mmol, 1.5 equiv) and 1,4-dioxane (2.0 mL). After stirred at room temperature for 2 h, the mixture was filtered by syringe and transferred to a Schlenk tubu charged with $[Rh(CO)_2Cl]_2$ (1.0 mg, 0.0025 mmol, 2.5 mol%), **L7** (7.3 mg, 0.0625 mmol, 6.25 mol%) and Cs_2CO_3 (10 mg, 0.030 mmol, 30 mol%). The mixture was stirred at 70 °C for 24 hour. After being cooled to room temperature, the mixture was filtered through Celite and the filtrate was concentrated in vacuum and purified by flash column chromatography (PE/EtOAc = 90:10) on silica gel to afford a mixture of **6m** and **6b** (46.6 mg, 83%). The ee value was determined by the mixture of **6m** and **6b**. HPLC conditions: Chiralcel AD-H, isopropanol/hexane = 20:80, flow: 1.0 mL/min, λ = 254 nm. The spectra data was listed as followed.

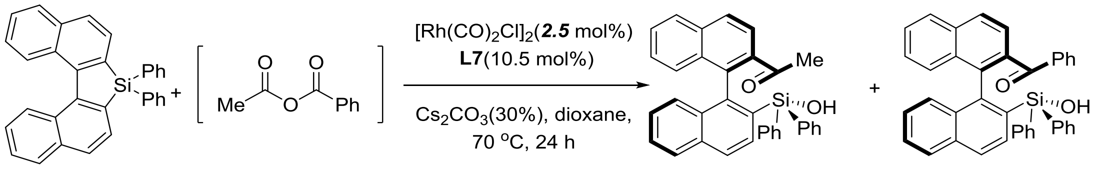

6c, 93% ee **6b**, 92% ee

Under nitrogen atmosphere, a Schlenk tube was sequentially charged with Cs_2CO_3 (3.0 equiv), benzoic acid (18.0 mg, 0.150 mmol, 1.5 equiv), Acetyl chloride (11 μL, 0.150 mmol, 1.5 equiv) and 1,4-dioxane (2.0 mL). After stirred at room temperature for 2 h, the mixture was filtered by syringe and transferred to a Schlenk tubu charged with $[Rh(CO)_2Cl]_2$ (1.0 mg, 0.0025 mmol, 2.5 mol%), **L7** (7.3 mg, 0.0625 mmol, 6.25 mol%) and Cs_2CO_3 (10 mg, 0.030 mmol, 30 mol%). The mixture was stirred at 70 °C for 24 hour. After being cooled to room temperature, the mixture was filtered through Celite and the filtrate was concentrated in vacuum and purified by flash column

chromatography (PE/EtOAc = 90:10) on silica gel to afford **6c** (6.0 mg, 11%, 92% ee) and **6b** (36.4 mg, 74%, 93% ee). **6c** HPLC conditions: Chiralcel AD-H, isopropanol/hexane = 20:80, flow: 1.0 mL/min, λ = 254 nm. **6b** HPLC conditions: Chiralcel AD-H, isopropanol/hexane = 15:85, flow: 1.0 mL/min, λ = 254 nm.

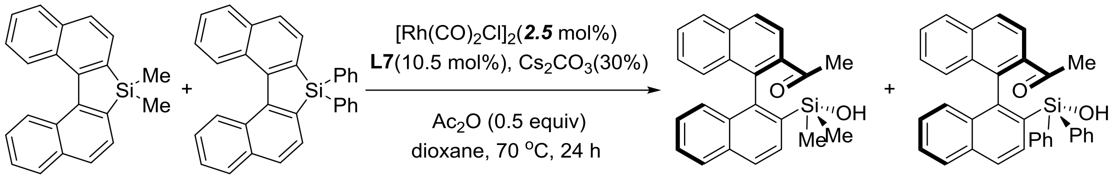

4a (1.0 equiv) **4b** (1.0 equiv) **6a**, 90% ee **6c**, 88% ee

Under nitrogen atmosphere, to a Schleck tube was added $[Rh(CO)_2Cl]_2$ (1.0 mg, 0.0025 mmol, 2.5 mol%), **L7** (12.2 mg, 0.0105 mmol, 10.5 mol%) and dioxane (2 mL) at room temperature and was stirred for 30 minutes. The solution was transfer via cannula carefully to another Schlenk tube charged with **4a** (31 mg, 0.100 mmol, 1.0 equiv), **4b** (44 mg, 0.100 mmol, 1.0 equiv), acetic anhydride (5 μL, 0.050 mmol, 0.5 equiv), Na_2CO_3 (3.2 mg, 0.030 mmol, 30 mol%). The tube was capped with a screw cap and stirred at 70 °C for 24 h. After being cooled to room temperature, the mixture was filtered through Celite and the filtrate was concentrated in vacuum and purified by flash column chromatography (PE/EtOAc = 90:10) on silica gel to afford **6a** (9.4 mg, 51%, 90% ee) and **6c** (8.4 mg, 34%, 88% ee). **6a** HPLC conditions: Chiralcel AD-H, isopropanol/hexane = 10:90, flow: 1.0 mL/min, λ = 254 nm. **6c** HPLC conditions: Chiralcel AD-H, isopropanol/hexane = 20:80, flow: 1.0 mL/min, λ = 254 nm.

Procedure for the synthesis of Hydroxysilanol 7

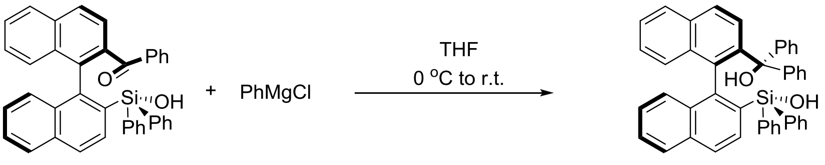

6b **7**

To the mixture of **6b** (28 mg, 0.05 mmol) in THF (2.0 mL) was added PhMgCl (75 μL, 0.15 mmol, 2.0 M in THF) at room temperature. Then the mixture was stirred over night before being quenched with saturated NH_4Cl (aq.). The mixture was extracted with ethyl acetate (5 mL*3), and the combined organic layer was washed with brine, dried over Na_2SO_4, filtered, concentrated and purified by flash column chromatography (PE/EtOAc/DCM = 80:10:10) on silica gel to afford **7** (30 mg, 94%, 91% ee). $[\alpha]_D^{20}$ + 110.45 (c 0.92, CH_2Cl_2). HPLC conditions: Chiralcel AD-H, isopropanol/hexane = 10:90, flow: 1.0 mL/min, λ = 254 nm. **^{1}H NMR** (500 MHz, $CDCl_3$) δ 7.85 (d, *J* = 8.5 Hz, 1H), 7.76 – 7.73 (m, 2H), 7.73 – 7.70 (m, 2H), 7.70 – 7.68 (m, 1H), 7.66 (d, *J* = 8.0 Hz, 1H), 7.42 (d, *J* = 7.0 Hz, 1H), 7.38 (t, *J* = 7.3 Hz, 2H), 7.34 – 7.31 (m, 2H), 7.31 – 7.30 (m, 2H), 7.30 – 7.28 (m, 1H), 7.24 – 7.19 (m, 2H), 7.19 – 7.16 (m, 1H), 7.12 – 7.09 (m, 2H), 7.09 –

7.07 (m, 1H), 7.00 – 6.96 (m, 1H), 6.96 – 6.93 (m, 2H), 6.93 – 6.90 (m, 2H), 6.85 (d, *J* = 7.5 Hz, 2H), 6.69 (t, *J* = 7.8 Hz, 1H), 6.58 (d, *J* = 8.5 Hz, 1H), 6.52 (t, *J* = 7.5 Hz, 1H), 6.25 (d, *J* = 8.5 Hz, 1H), 3.24 (s, 1H), 3.21 (s, 1H). **^{13}C NMR** (125 MHz, $CDCl_3$) δ 147.2, 144.1, 143.9, 142.0, 136.1, 136.0, 135.3, 134.8, 134.2, 133.8, 133.73, 133.65, 132.0, 131.9, 131.6, 129.9, 129.0, 128.7, 128.3, 128.2, 128.1, 127.9, 127.6, 127.4, 127.30, 127.25, 127.2, 127.1, 126.99, 126.95, 126.9, 126.6, 126.4, 126.1, 125.9, 125.4, 84.7. **HRMS (ESI)** calcd for $C_{45}H_{35}O_2Si$ $[M+H]^+$ 635.2406, found 635.2407.

Procedure for the synthesis of Bromides 9

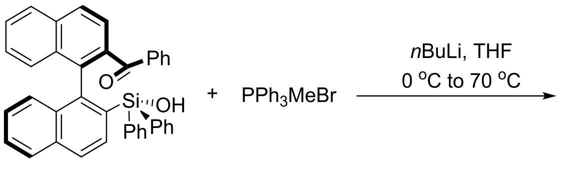

6b 8

To the Flask tube charged with methyltriphenylphosphonium bromide (1.79 g, 5.00 mmol) in THF (8 mL) was added nBuLi (2.10 mL, 5.00 mmol, 2.4 M in hexanes) dropwise at 0 °C. Then the mixture was allowed to warm to room temperature and stirred for 1 hour. A mixture of **6b** (0.278 g, 0.50 mmol) in THF (2 mL) was added slowly at room temperature and the mixture was stirred at 70 °C for 12 hours. After being cooled to room temperature, the reaction was quenched with water (5 mL) and extracted with ethyl acetate (5 mL*3), and the combined organic layer was washed with brine, dried over Na_2SO_4, filtered, concentrated and purified by flash column chromatography (PE/EtOAc = 90:10) on silica gel to afford **8** (0.259 g, 93%, 91% ee). $[\alpha]_D^{20}$ + 9.89 (c 0.93, CH_2Cl_2). HPLC conditions: Chiralcel AD-H, isopropanol/hexane = 10:90, flow: 1.0 mL/min, λ = 254 nm. **^{1}H NMR** (500 MHz, $CDCl_3$) δ 7.96 (d, *J* = 8.5 Hz, 1H), 7.86 (d, *J* = 8.0 Hz, 1H), 7.70 (d, *J* = 8.5 Hz, 1H), 7.65 (d, *J* = 8.5 Hz, 1H), 7.62 (d, *J* = 8.5 Hz, 1H), 7.58 – 7.56 (m, 1H), 7.56 – 7.52 (m, 2H), 7.40 (t, *J* = 7.3 Hz, 1H), 7.37 – 7.33 (m, 2H), 7.33 – 7.31 (m, 1H), 7.30 – 7.26 (m, 1H), 7.20 (t, *J* = 7.5 Hz, 1H), 7.11 – 7.08 (m, 2H), 7.08 – 7.04 (m, 2H), 7.02 – 6.99 (m, 2H), 6.99 – 6.96 (m, 1H), 6.92 (t, *J* = 7.5 Hz, 1H), 6.83 – 6.77 (m, 2H), 6.77 – 6.76 (m, 2H), 6.76 – 6.73 (m, 1H), 5.19 (d, *J* = 9.0 Hz, 2H). **^{13}C NMR** (125 MHz, $CDCl_3$) δ 149.8, 144.1, 140.9, 139.3, 136.4, 135.9, 135.5, 135.1, 134.2, 133.4, 133.3, 132.8, 132.59, 132.57, 131.4, 129.8, 129.2, 128.4, 128.2, 127.6, 127.38, 127.35, 127.24, 127.23, 126.8, 126.5, 126.4, 126.3, 126.2, 126.1, 125.4, 118.5. **HRMS (ESI)** calcd for $C_{40}H_{30}OSiNa$ $[M+Na]^+$ 577.1964, found 577.1969.

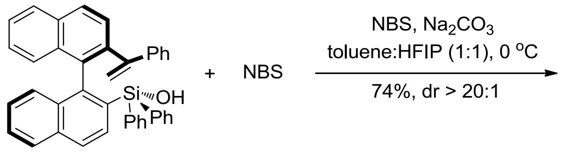

8 9

To the mixture of **8** (54 mg, 0.10 mmol) and Na_2CO_3 (42 mg, 0.40 mmol) in toluene (5.0 mL) and hexafluoroisopropanol (5.0 mL) was added NBS (19 mg, 0.105 mmol) in one portion at 0 °C. Then the mixture was stirred at the same temperature until the complete consumption of **8**. Then mixture was allowed to warm to room temperature and filter through Celite, concentrated and purified by flash column chromatography (PE/DCM = 90:10) on silica gel to afford **9** (47 mg, 74%, 90% ee). $[\alpha]_D^{20}$ + 86.77 (c 1.13, CH_2Cl_2). HPLC conditions: Chiralcel AD-H, isopropanol/hexane = 6:94, flow: 1.0 mL/min, λ = 273 nm. **^{1}H NMR** (500 MHz, $CDCl_3$) δ 7.98 (d, *J* = 9.0 Hz, 1H), 7.82 (d, *J* = 7.0 Hz, 2H), 7.65 (d, *J* = 9.0 Hz, 1H), 7.58 (d, *J* = 8.0 Hz, 2H), 7.57 – 7.53 (m, 1H), 7.53 – 7.49 (m, 2H), 7.49 – 7.45 (m, 1H), 7.33 (d, *J* = 8.0 Hz, 1H), 7.30 – 7.28 (m, 1H), 7.28 – 7.24 (m, 2H), 7.08 – 7.05 (m, 2H), 7.05 – 6.99 (m, 2H), 6.99 – 6.95 (m, 1H), 6.89 – 6.85 (m, 1H), 6.85 – 6.81 (m, 1H), 6.81 – 6.76 (m, 2H), 6.62 (t, *J* = 7.5 Hz, 2H), 6.58 – 6.43 (m, 1H), 6.39 (t, *J* = 7.0 Hz, 1H), 4.27 (d, *J* = 10.5 Hz, 1H), 4.19 (d, *J* = 10.5 Hz, 1H). **^{13}C NMR** (125 MHz, $CDCl_3$) δ 144.6, 143.3, 140.2, 137.6, 135.5, 134.0, 133.9, 133.7, 133.54, 133.46, 132.8, 132.6, 131.4, 130.2, 129.6, 128.2, 128.1, 127.8, 127.6, 127.5, 127.3, 127.1, 126.2, 126.1, 126.0, 125.8, 125.7, 125.0, 124.9, 123.9, 80.5, 44.4. **HRMS (ESI)** calcd for $C_{40}H_{29}OSiBrNa$ $[M+Na]^+$ 655.1069, found 655.1058.

Procedure for the application of silanol on cross-coupling reaction

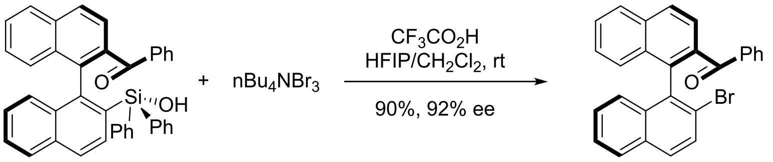

6b, 91% ee   **10**

To the solution of **6b** (56 mg, 0.10 mmol, 91% ee) in CF_3COOH (0.2 mL), hexafluoroisopropanol (4 mL) and DCM (2.0 mL) was added nBu_4NBr_3 (72 mg, 0.15 mmol, 1.50 equiv) at room temperature. Then the mixture was stirred at the same temperature until the complete consumption of **6b**. Then mixture was diluted with ethyl acetate (5.0 mL), extracted with ethyl acetate (5.0 mL*3), the combined organic layer was washed with brine, dried over Na_2SO_4, concentrated and purified by flash column chromatography (PE/EA = 95:5) on silica gel to afford **10** (39 mg, 90%, 92% ee). $[\alpha]_D^{20}$ + 28.2 (c 0.98, CH_2Cl_2). HPLC conditions: Chiralcel OD-H, isopropanol/hexane = 5:95, flow: 1.0 mL/min, λ = 254 nm. **^{1}H NMR** (400 MHz, $CDCl_3$) δ 8.19 (d, *J* = 8.4 Hz, 1H), 8.06 (d, *J* = 8.4 Hz, 1H), 8.00 (d, *J* = 8.4 Hz, 1H), 7.70 – 7.64 (m, 2H), 7.60 – 7.53 (m, 1H), 7.53 – 7.50 (m, 1H), 7.50 – 7.46 (m, 2H), 7.38 – 7.34 (m, 1H), 7.34 – 7.32 (m, 2H), 7.32 – 7.28 (m, 2H), 7.18 – 7.15 (m, 1H), 7.15 – 7.08 (m, 2H). **^{13}C NMR** (100 MHz, $CDCl_3$) δ 198.4, 137.8, 137.5, 136.2, 135.4, 134.1, 133.9, 132.6, 132.5, 131.5, 129.3, 129.1, 129.0, 128.2, 128.1, 127.8, 127.4, 127.28, 127.26, 127.21, 127.16, 127.1, 126.8, 124.9, 123.2. **HRMS (ESI)** calcd for $C_{27}H_{18}OBr$ $[M+H]^+$ 437.0541, found 437.0543.

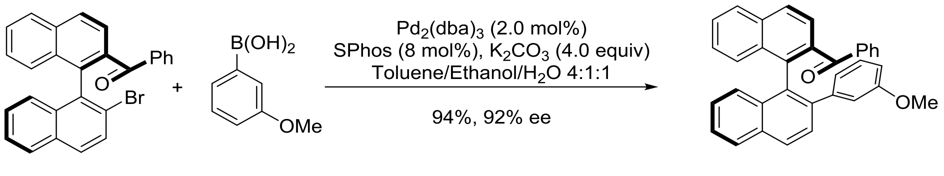

10, 92% ee **11**

The mixture of **10** (22 mg, 0.050 mmol, 1.0 equiv, 92% ee), 3-methoxyphenylboronic acid (12 mg, 0.075 mmol, 1.5 equiv), $Pd_2(dba)_3$ (0.9 mg, 0.001 mmol, 2.0 mol%), SPhos (1.6 mg, 0.004 mmol, 8.0 mol%) and K_2CO_3 (28 mg, 0.200 mmol, 4.0 equiv) in toluene (0.8 mL), ethanol (0.2 mL) and H_2O (0.2 mL) was stirred at 90 ^{o}C for 3 hours. After being cooled to room temperature, the reaction was diluted with ethyl acetate (5 mL) and extracted with ethyl acetate (5 mL*3), and the combined organic layer was washed with brine, dried over Na_2SO_4, filtered, concentrated and purified by flash column chromatography (PE/EtOAc = 90:10) on silica gel to afford **11** (22 mg, 94%, 92% ee). $[\alpha]_D^{20}$ + 50.0 (c 0.87, CH_2Cl_2). HPLC conditions: Chiralcel OD-H, isopropanol/hexane = 5:95, flow: 1.0 mL/min, λ = 254 nm. **^{1}H NMR** (400 MHz, $CDCl_3$) δ 8.07 (d, *J* = 8.4 Hz, 1H), 8.02 (d, *J* = 8.0 Hz, 1H), 7.82 (d, *J* = 8.4 Hz, 1H), 7.73 (d, *J* = 8.4 Hz, 1H), 7.61 – 7.54 (m, 1H), 7.50 (d, *J* = 8.4 Hz, 1H), 7.45 – 7.42 (m, 2H), 7.42 – 7.40 (m, 2H), 7.40 – 7.35 (m, 2H), 7.35 – 7.30 (m, 2H), 7.29 – 7.27 (m, 1H), 7.25 – 7.20 (m, 1H), 7.13 – 7.05 (m, 2H), 7.04 – 7.00 (m, 1H), 7.00 – 6.94 (m, 2H), 3.87 (s, 3H). **^{13}C NMR** (100 MHz, $CDCl_3$) δ 199.2, 159.4, 143.3, 142.0, 140.2, 138.0, 137.8, 137.4, 134.78, 134.75, 134.1, 133.1, 132.7, 132.0, 131.2, 130.5, 129.1, 129.03, 128.96, 128.5, 128.4, 128.1, 127.6, 127.5, 127.2, 126.9, 126.2, 125.8, 125.7, 125.4, 125.2, 122.5, 115.6, 112.9, 55.3. **HRMS (ESI)** calcd for $C_{34}H_{25}O$ $[M+H]^+$ 465.1855, found 465.1847.

Procedure for the synthesis of Rhodium-Ligand Complex

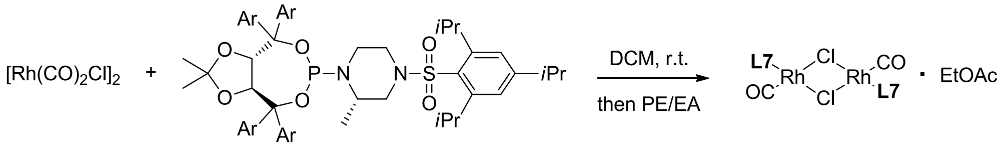

Ar = 4-PhC$_6$H$_4$:**L7** **Rh-1**

A mixture of $[Rh(CO)_2Cl]_2$ (5.8 mg, 0.015 mmol, 1.0 equiv) and **L7** (35.0 mg, 0.03 mmol, 2.0 equiv) in DCM (2 mL) was stirred at room temperature for 30 mins. Then the solvent was removed under vacuum and crystallized in mixture solvent (PE:EtOAc = 10:1) under an atmosphere of nitrogen at – 20 ^{o}C to afford orange crystal **Rh-1** (30 mg, 80%). **^{1}H NMR** (400 MHz, $CDCl_3$) δ 7.72 – 7.67 (m, 4H), 7.67 – 7.63 (m, 4H), 7.63 – 7.60 (m, 2H), 7.58 – 7.55 (m, 2H), 7.55 – 7.52 (m, 2H), 7.52 – 7.50 (m, 2H), 7.50 – 7.47 (m, 4H), 7.47 – 7.44 (m, 2H), 7.43 – 7.38 (m, 4H), 7.38 – 7.34 (m, 4H), 7.34 – 7.32 (m, 4H), 7.32 – 7.29 (m, 2H), 7.05 (s, 2H), 5.76 (d, *J* = 8.0 Hz, 1H), 5.66 – 5.51 (m, 1H), 5.36 (d, *J* = 8.0 Hz, 1H), 4.08 – 3.94 (m, 2H), 3.65 – 3.50 (m, 1H), 3.35 – 3.26 (m, 1H), 3.13 – 3.02 (m, 1H), 2.92 – 2.78 (m, 3H), 1.99 – 1.83 (m, 1H), 1.21 (d, *J* = 6.8 Hz, 6H), 1.16 (d, *J* = 6.8 Hz, 3H), 1.12 (d, *J* = 6.4 Hz, 6H), 1.00 (d, *J* = 6.8 Hz, 6H), 0.59 (s, 3H), 0.52 (s, 3H). **^{13}C NMR** (126 MHz, $CDCl_3$) δ 153.2, 152.0, 142.3, 142.2, 141.21, 141.17, 140.5, 140.4, 140.2, 139.9, 129.7, 129.1, 128.9, 128.83, 128.78, 128.72, 128.68, 128.4, 127.9,

127.8, 127.7, 127.4, 127.3, 127.2, 127.1, 127.01, 126.96, 125.6, 123.8, 115.1, 90.7 (d, *J* = 18.9 Hz), 86.8, 78.6, 78.3, 50.8, 50.6, 48.4, 45.3, 40.4, 34.1, 29.3, 26.7, 26.5, 25.0, 24.6, 23.5, 17.2. **^{31}P NMR** (162 MHz, $CDCl_3$) δ 115.9, 114.2.

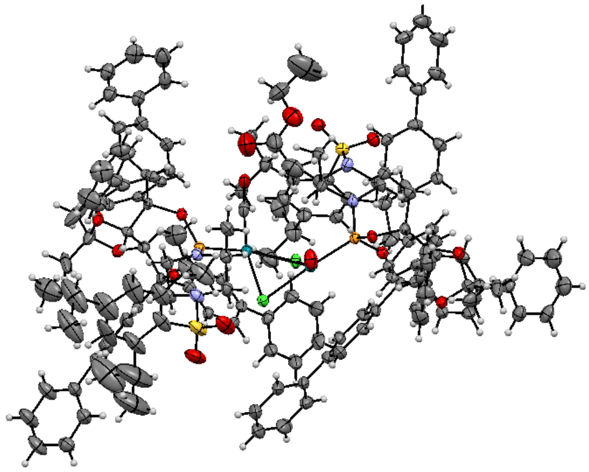

Structure of Rh-1·EtOAc

Crystal data and structure refinement for Rh-1.

Identification code	11-15
Empirical formula	$C_{156}H_{162}Cl_2N_4O_{16}P_2Rh_2S_2$
Formula weight	2751.67
Temperature/K	99.99(10)
Crystal system	monoclinic
Space group	$P2_1$
a/Å	14.9883(2)
b/Å	34.9555(5)
c/Å	15.2977(2)
α/°	90
β/°	100.2050(10)
γ/°	90
Volume/Å^3	7888.03(19)
Z	2
ρ_{calc}g/cm^3	1.159
μ/mm^{-1}	2.902
F(000)	2880.0
Crystal size/mm^3	0.12 × 0.11 × 0.1
Radiation	Cu Kα (λ = 1.54184)
2Θ range for data collection/°	5.056 to 147.232
Index ranges	-18 ≤ h ≤ 16, -37 ≤ k ≤ 42, -18 ≤ l ≤ 17
Reflections collected	31702
Independent reflections	22516 [R_{int} = 0.0496, R_{sigma} = 0.0696]

Data/restraints/parameters	22516/8/1671
Goodness-of-fit on F^2	1.029
Final R indexes [I>=2σ (I)]	R_1 = 0.0481, wR_2 = 0.1213
Final R indexes [all data]	R_1 = 0.0517, wR_2 = 0.1245
Largest diff. peak/hole / e $Å^{-3}$	0.90/-0.97
Flack/Hooft parameter	-0.009(5)/0.008(5)

Supplementary Table 1. Optimization of Reaction Conditions for Bromocyclization

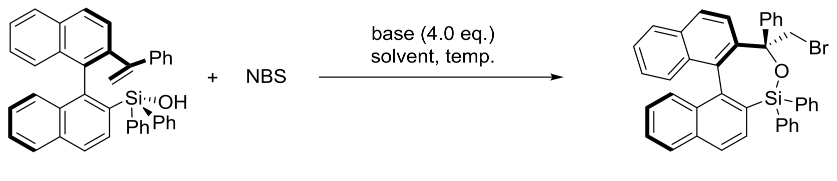

8 1.5 eq. **9**

Entry	base	solvent	temp. (°C)	yield/%	d.r.
1	$NaHCO_3$	THF	0	95	2.4:1
2	$NaHCO_3$	DMF	0	95	2.6:1
3	$NaHCO_3$	Mesitylene	0	99	2.5:1
4	$NaHCO_3$	o-Xylene	0	58	1.8:1
5	$NaHCO_3$	CH_3OH	0	80	1:1.2
6	$NaHCO_3$	$CHCl_3$	0	41	1.6:1
7	$NaHCO_3$	toluene	0	35	2.8:1
8	$NaHCO_3$	toluene	-78	<10	5.3:1
9	Na_2CO_3	toluene	-78	<10	6.0:1
10	Na_2CO_3	toluene:HFIP 2:1	0	91	16.6:1
11[a]	Na_2CO_3	toluene:HFIP 1:1	0	74	>20:1

[a] 1.05 eq. NBS was used;

DFT Calculations:

Computational Methods: B2PLYPD3/def2-TZVP // B3LYP-D3BJ/6-311G(d,p)
Energies are given in kcal/mol

All of the DFT calculations were performed with Gaussian 16 software packages[5]. The optimization calculations were employed at B3LYP level of theory[6] at 298.15 K with the D3 version of Grimme's dispersion (with Becke-Johnson damping)[7]. The 6-311G(d,p) basis sets[8] were employed for the C, H, Si atoms. Vibrational frequency analysis were calculated at the same level of theory to verify whether each optimized structure is an energy minimum and to evaluate its zero-point vibrational energy. All of the product structures were fully optimized without any symmetric restrictions. For each transition state, the intrinsic reaction coordinate (IRC) analysis was conducted to ensure that it connects the right reactant and product.[9] To obtain more accurate energies, single-point energy calculations were performed on all optimized structures applying the def2-TZVP basis set[10] at the B2PLYPD3 level of theory.[11] A standard state of 298.15 K and 1 atm was used. All discussed energies are Gibbs free energies in gas phase (ΔG_g).

Supplementary Table 2. Thermal correction of Gibbs free energy (TCG, hartree) and single-point energies (E, hartree) in 298.15 K and 1 atm for all species involved in this study.

Compounds	TCG	E	Compounds	TCG	E
**4c**	0.194088	-830.318409	**4c”**	0.072747	-231.817104
**H_2**	-0.001444	-1.159449	**4a’**	0.297727	-1138.239765
**4c-HH**	0.211558	-831.490834	**4a’-H**	0.179531	-754.224730
(*R*)-**4a**	0.281270	-1137.034077	**4a”**	0.115796	-385.185538
**4a-HH**	0.298668	-1138.226462	(*S*)-**4a**	0.281286	-1137.034072
**4c’**	0.210317	-831.501312	**TS-rs**	0.280614	-1137.000971
**4c’-H**	0.136044	-600.855605			

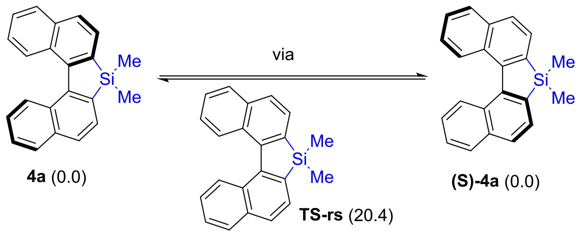

According to the Eyring equation:

$$k = \frac{k_B T}{h} e^{\frac{-\Delta G}{RT}}$$

The reaction rate for the racemization of **4a**:

$$k = 7.4E\text{-}3\ s^{-1}$$

For this rotation process is a first-order reaction, Equation relates half-life to rate constant for first order reactions:

$$t_{1/2} = \frac{ln2}{k} \approx 94\ s$$

Copies of NMR Spectroscopies

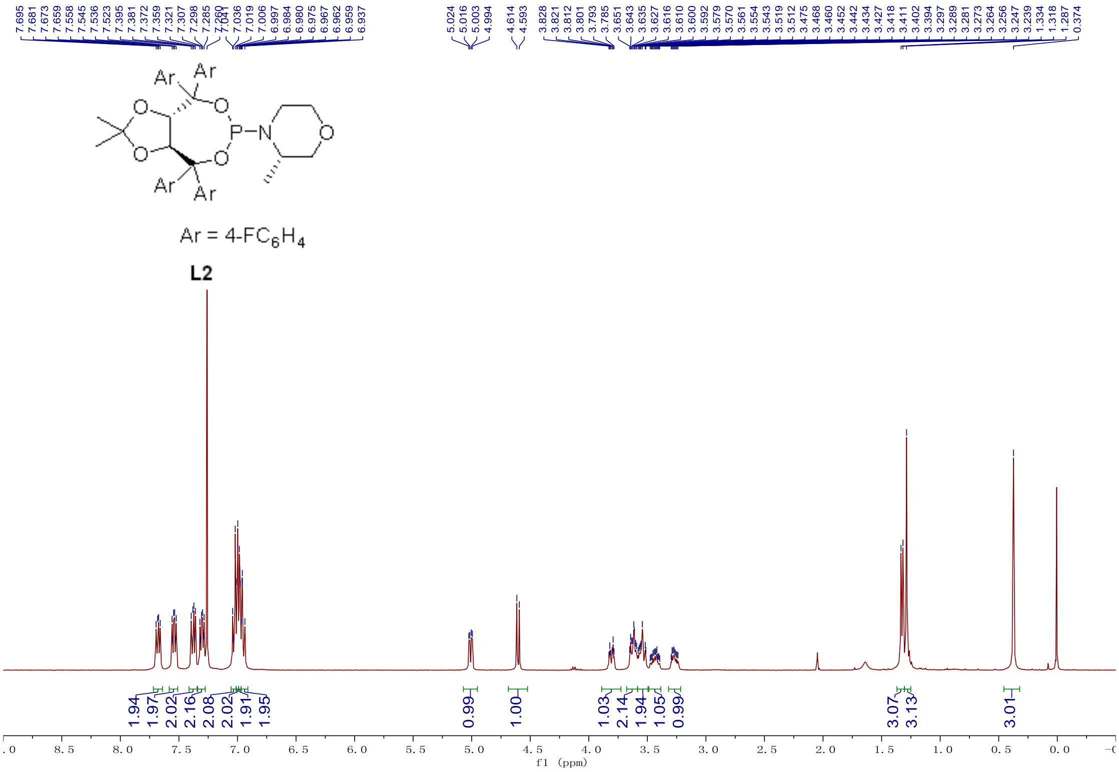

Supplementary Figure 1. ^{1}**H NMR** spectroscopy of **L2**

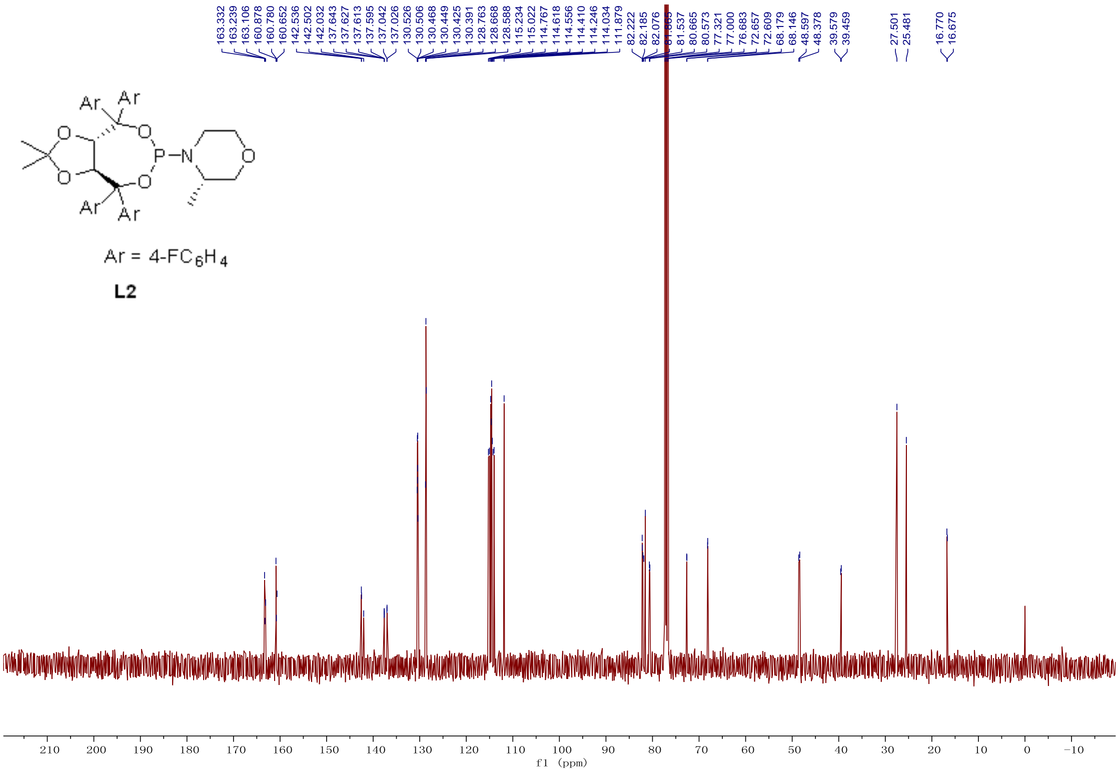

Supplementary Figure 2. ^{13}**C NMR** spectroscopy of **L2**

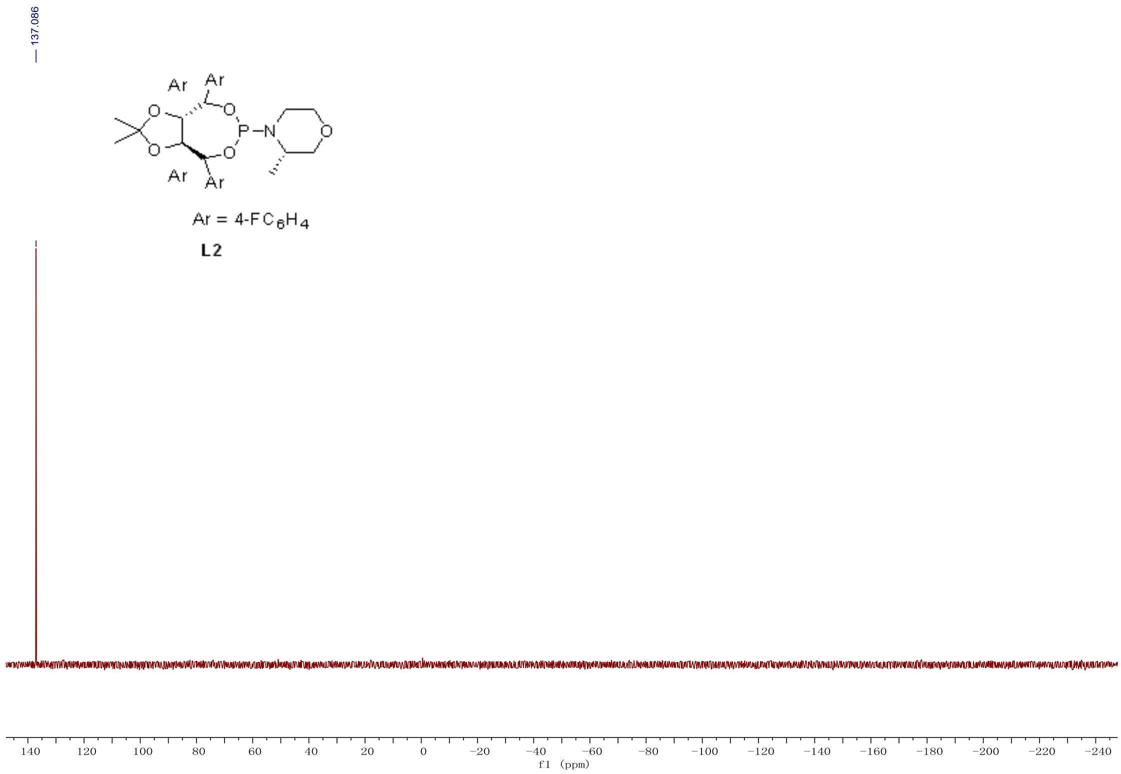

Supplementary Figure 3. **^{31}P NMR** spectroscopy of **L2**

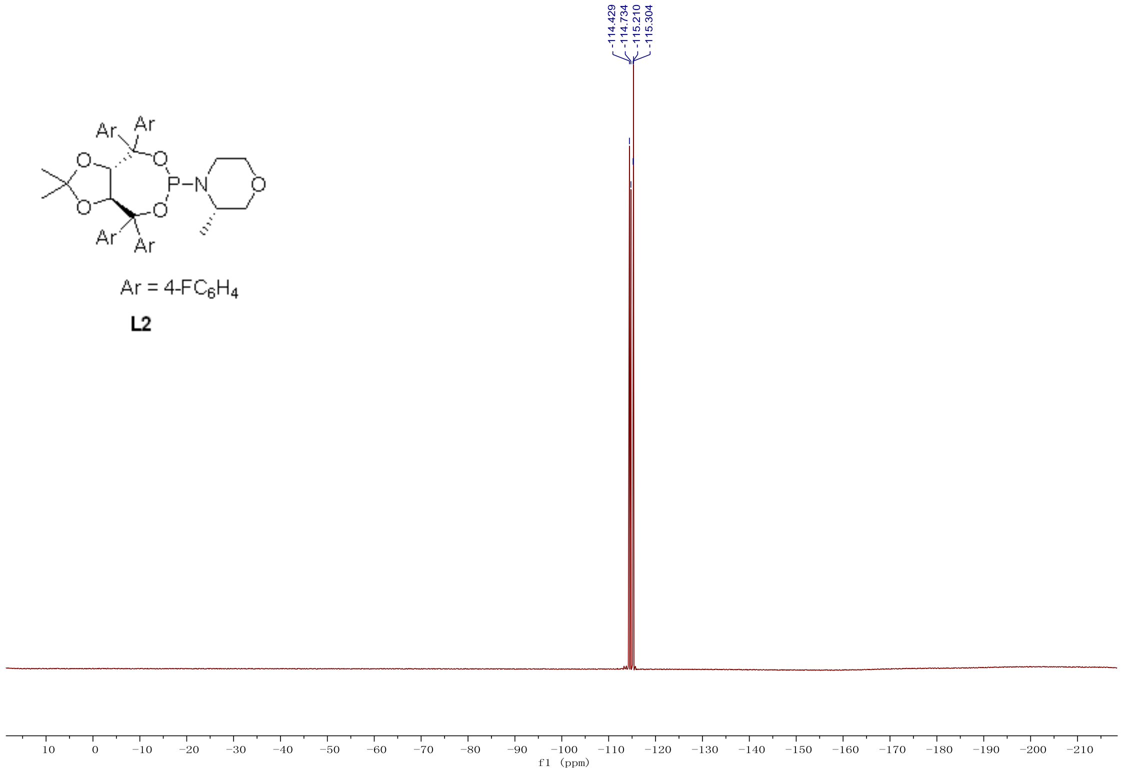

Supplementary Figure 4. **^{19}F NMR** spectroscopy of **L2**

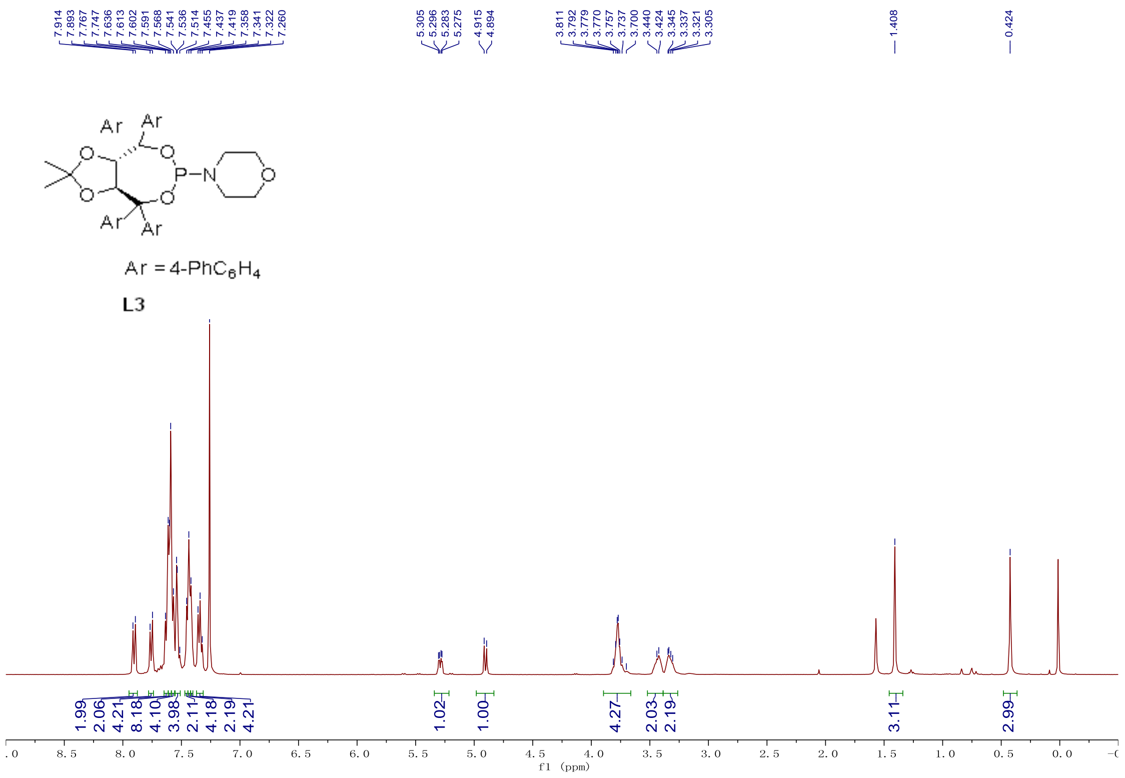

Supplementary Figure 5. ^{1}**H NMR** spectroscopy of **L3**

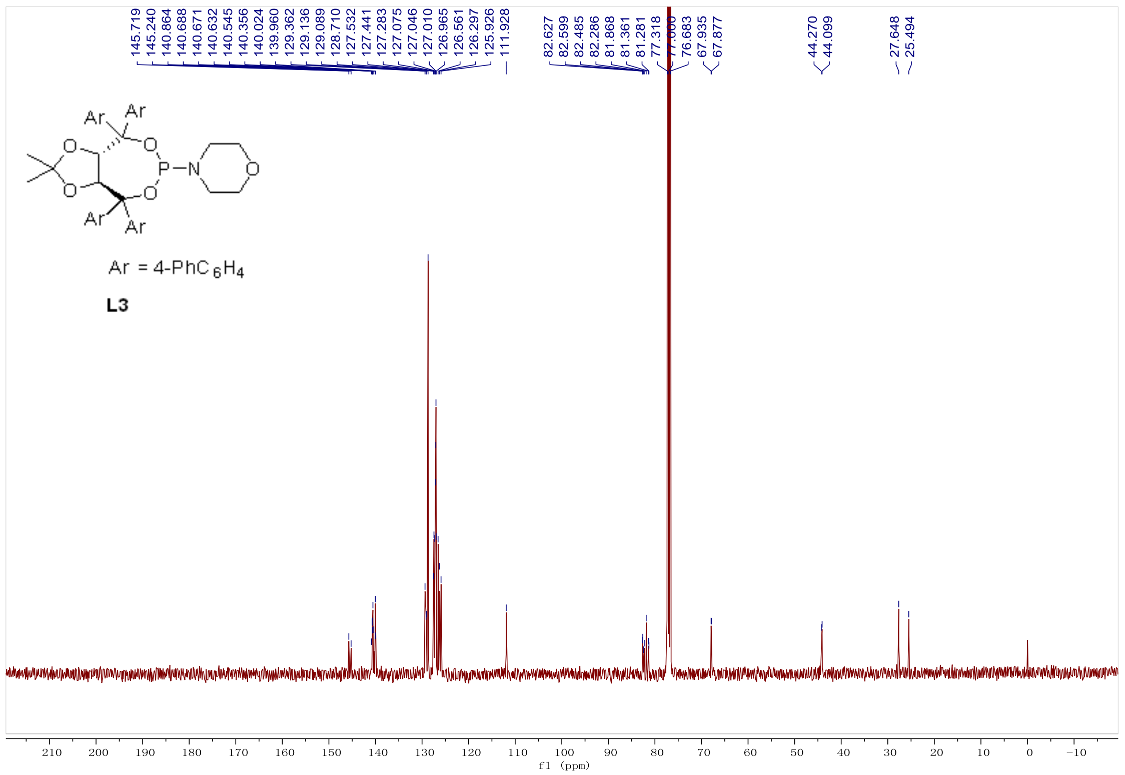

Supplementary Figure 6. ^{13}**C NMR** spectroscopy of **L3**

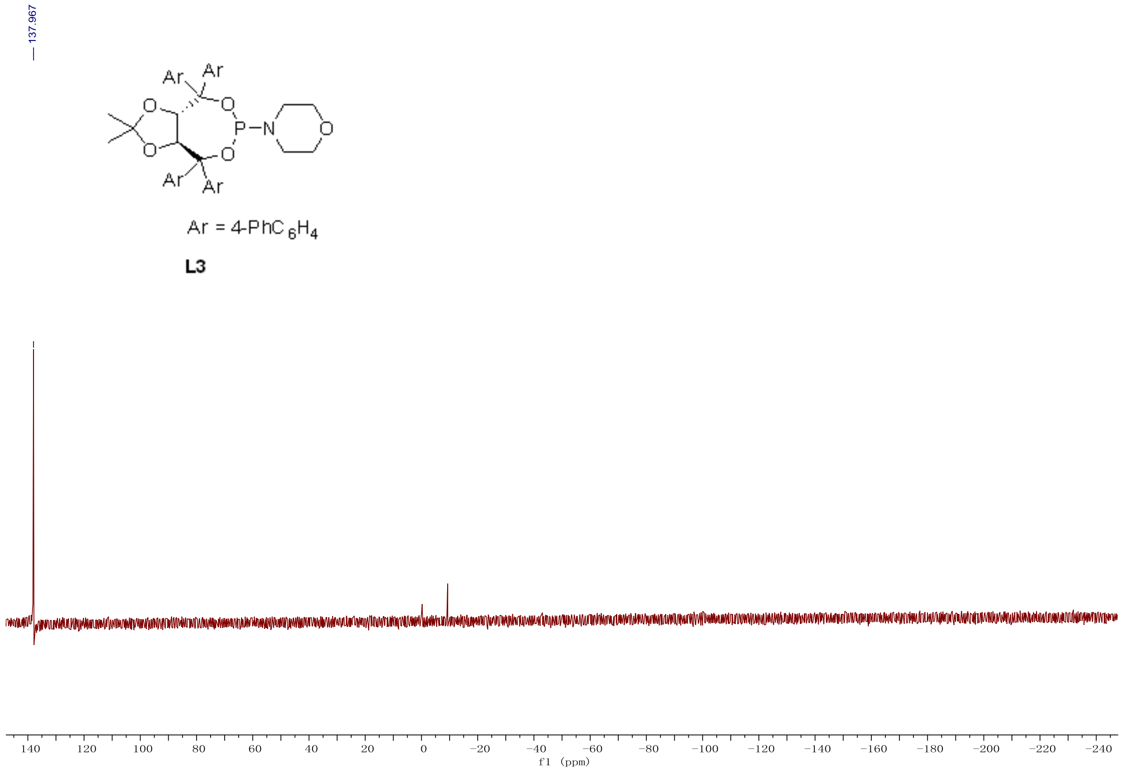

Supplementary Figure 7. ^{31}**P NMR** spectroscopy of **L3**

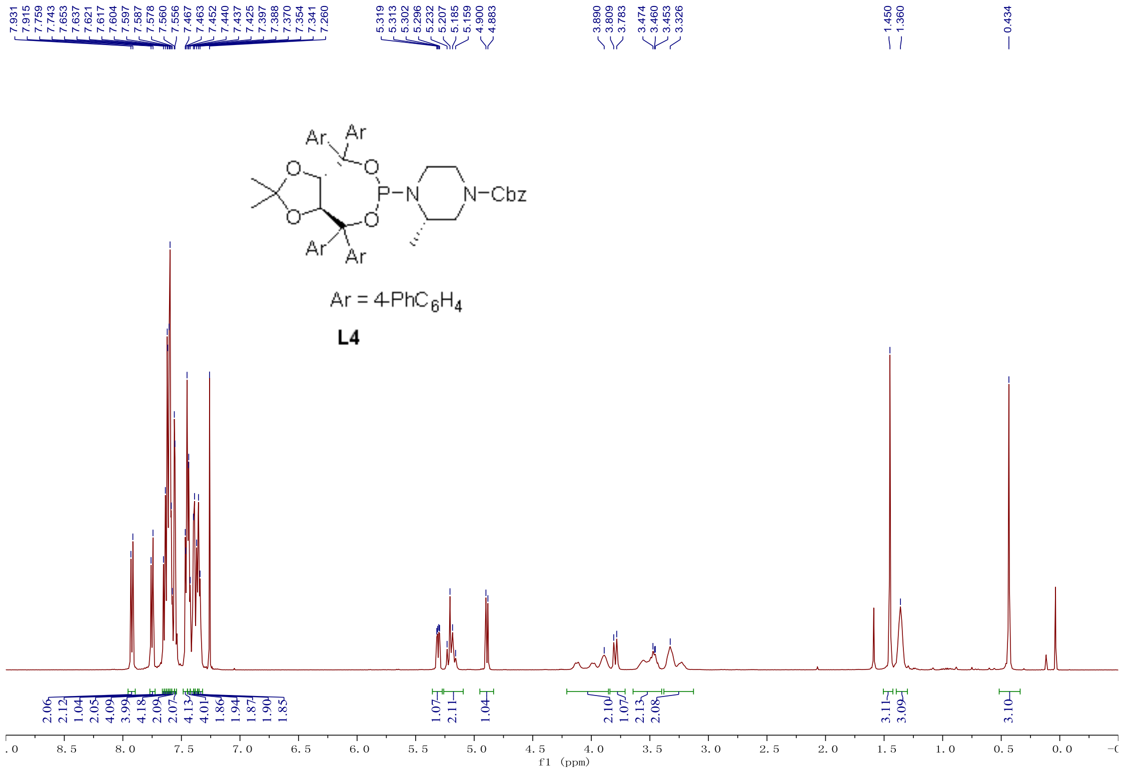

Supplementary Figure 8. ^{1}**H NMR** spectroscopy of **L4**

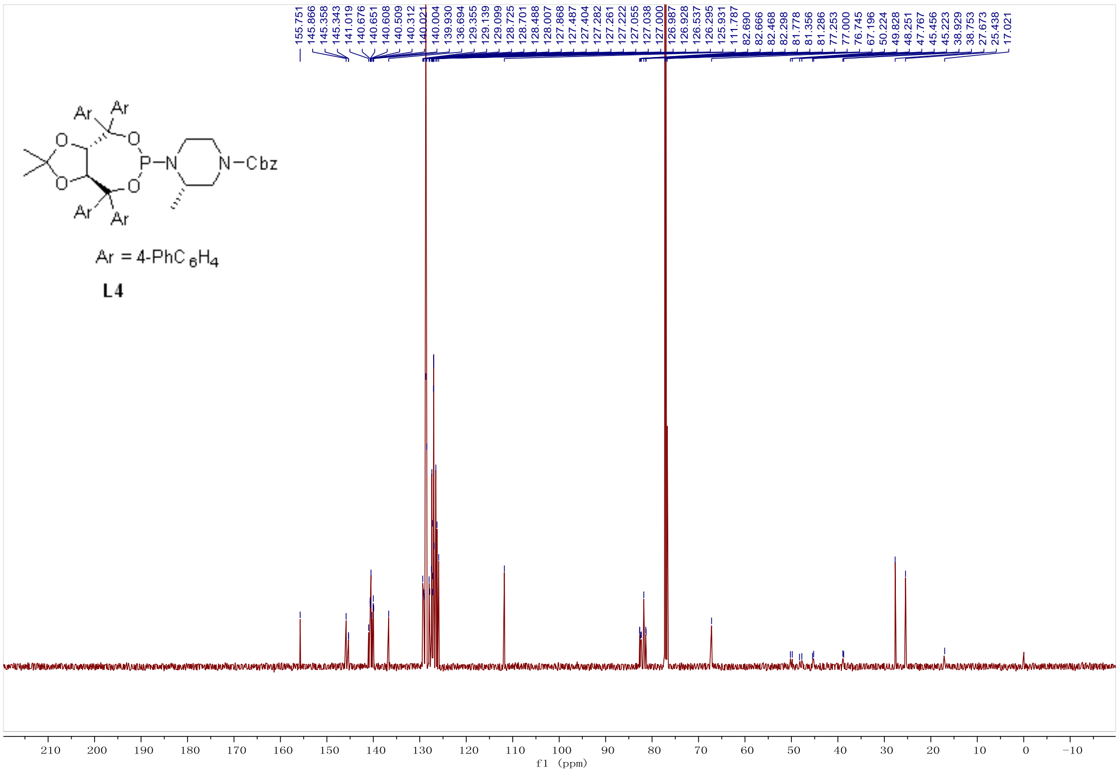

Supplementary Figure 9. ^{13}**C NMR** spectroscopy of **L4**

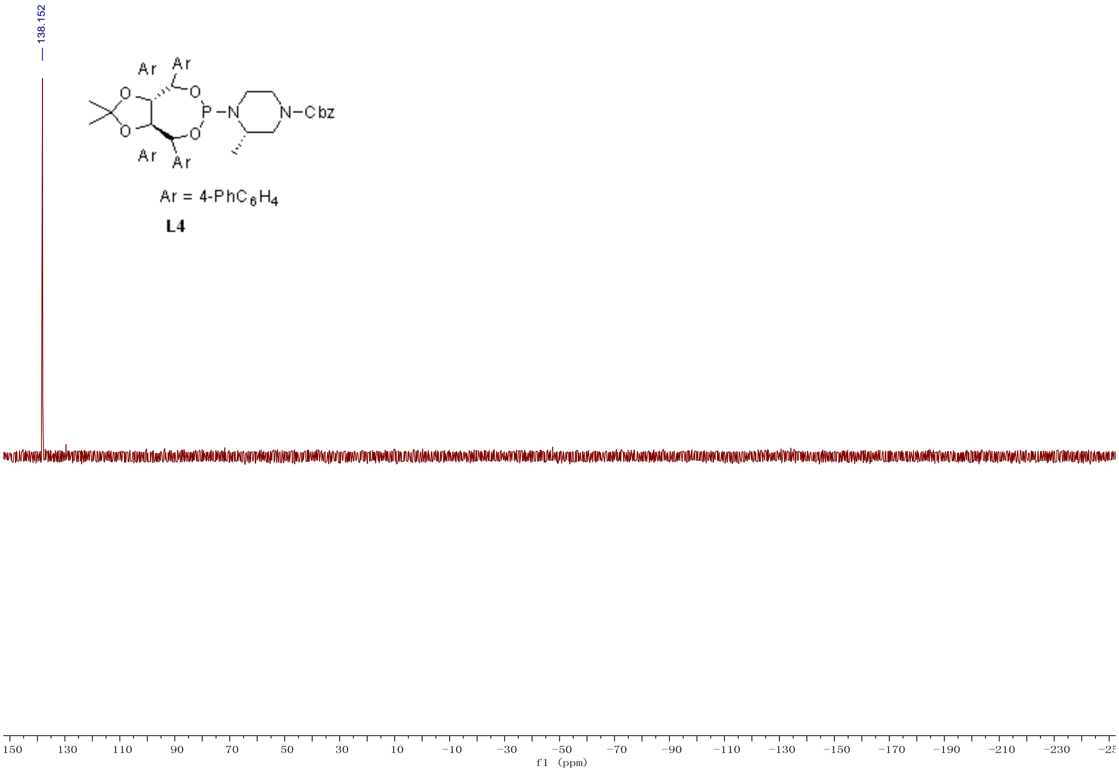

Supplementary Figure 10. ^{31}**P NMR** spectroscopy of **L4**

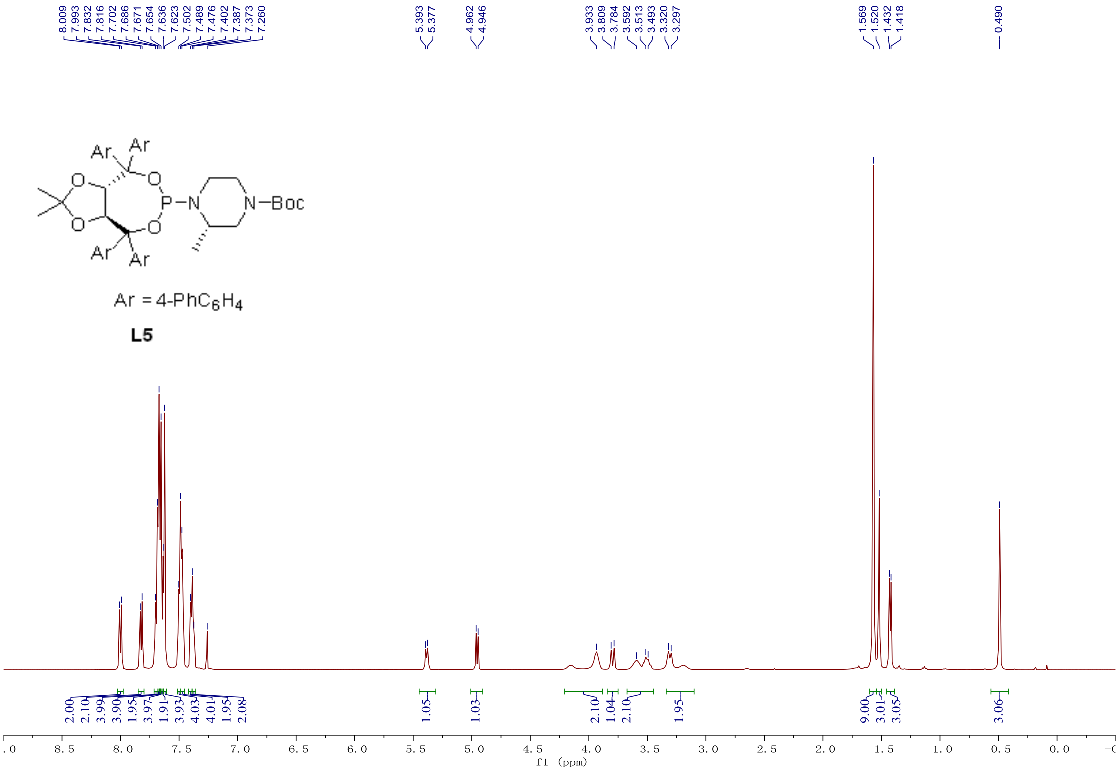

Supplementary Figure 11. ^{1}**H NMR** spectroscopy of **L5**

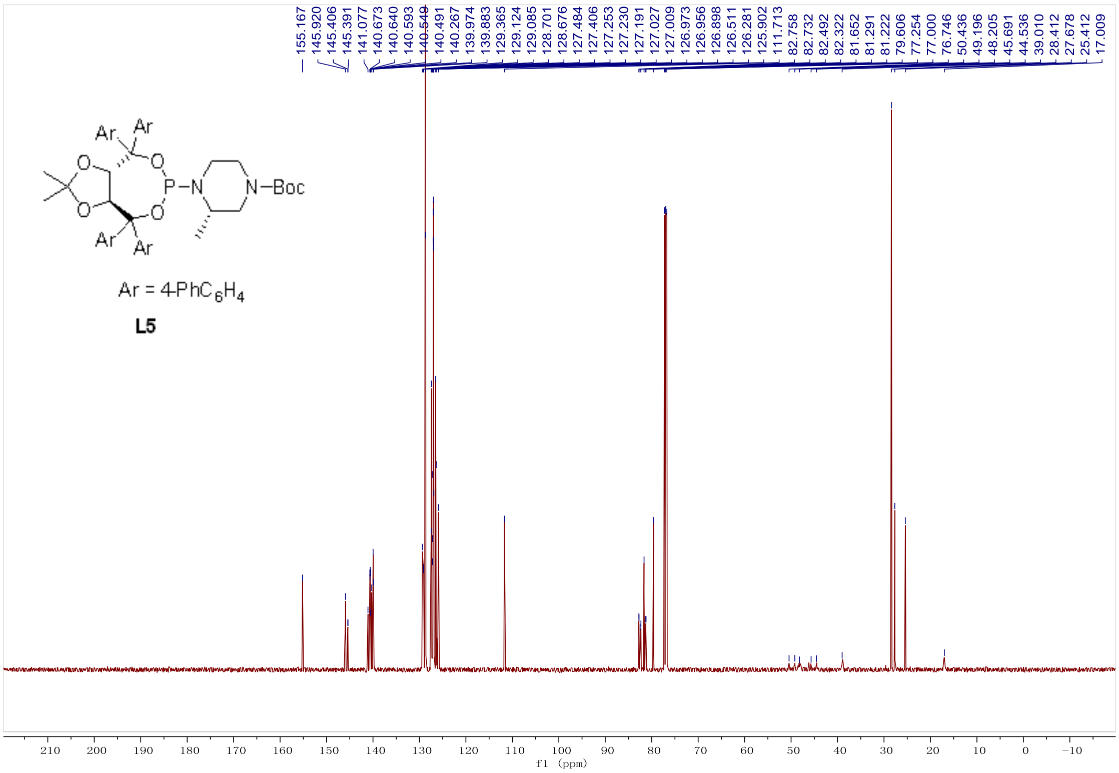

Supplementary Figure 12. ^{13}**C NMR** spectroscopy of **L5**

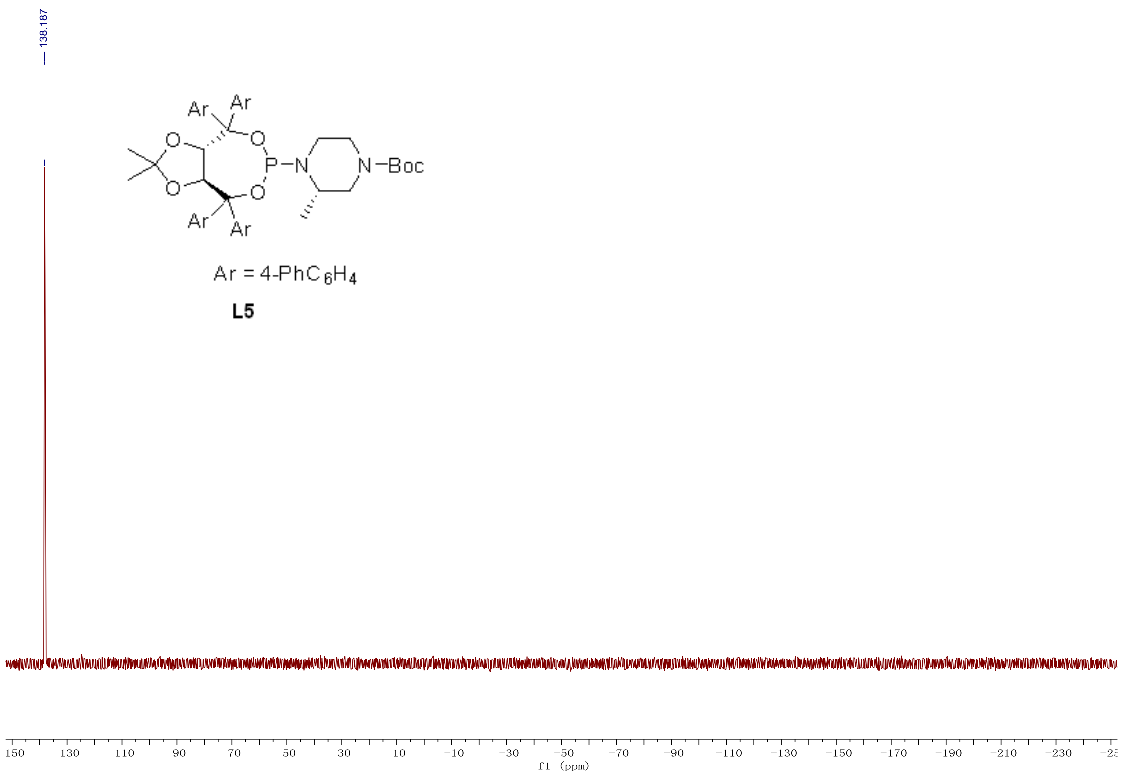

Supplementary Figure 13. ^{31}**P NMR** spectroscopy of **L5**

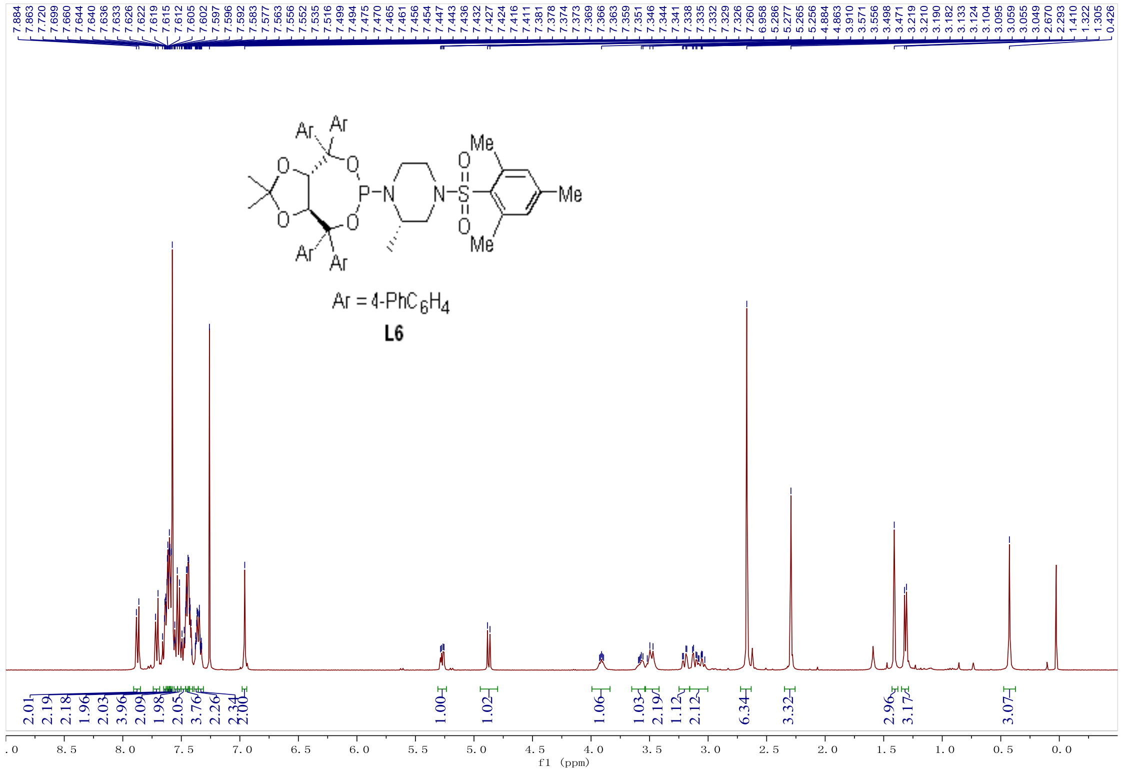

Supplementary Figure 14. ^{1}**H NMR** spectroscopy of **L6**

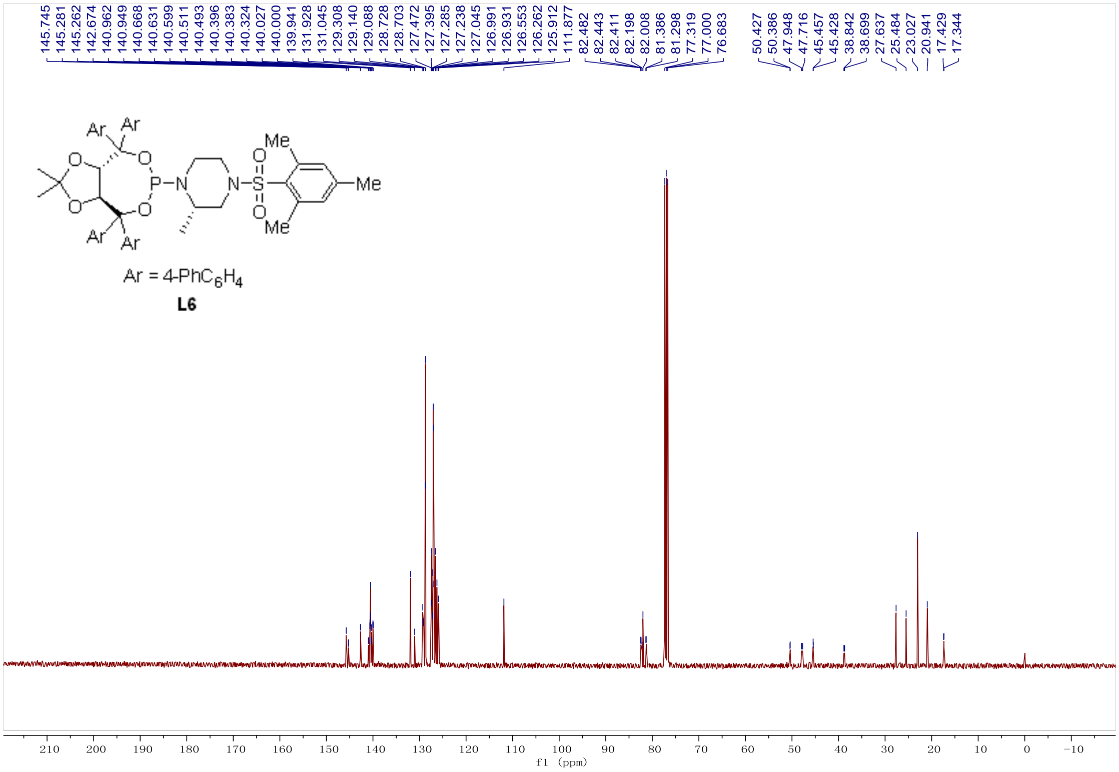

Supplementary Figure 15. ^{13}**C NMR** spectroscopy of **L6**

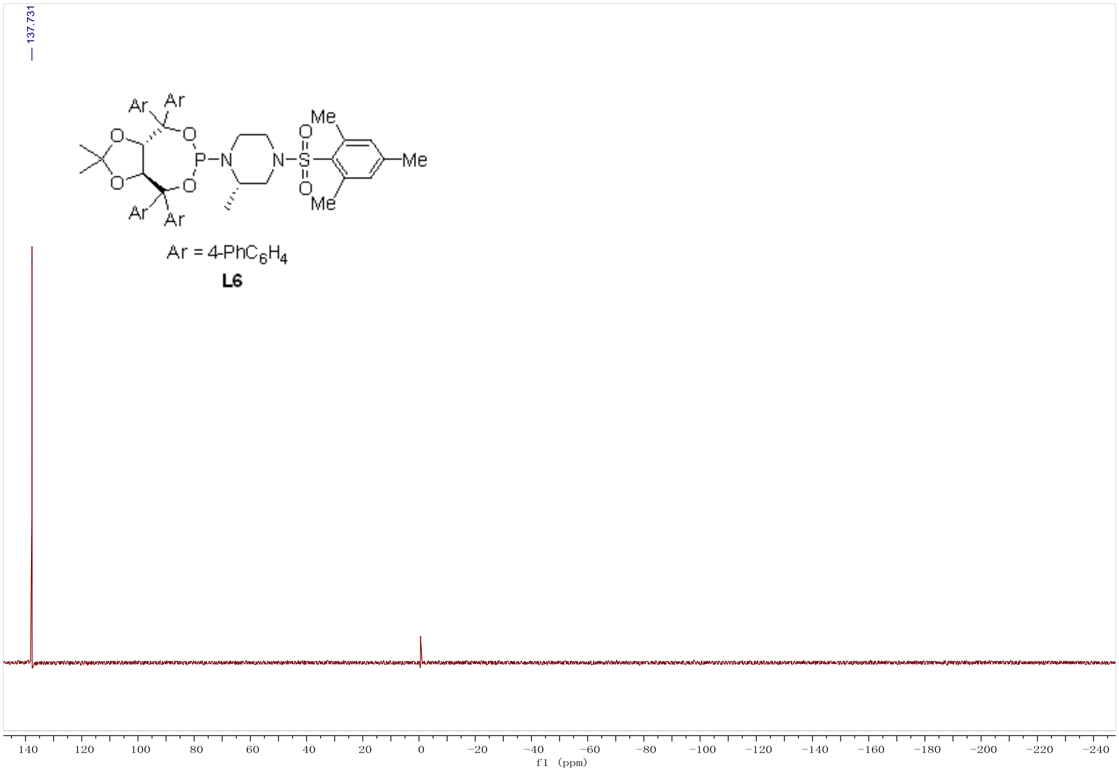

Supplementary Figure 16. ^{31}**P NMR** spectroscopy of **L6**

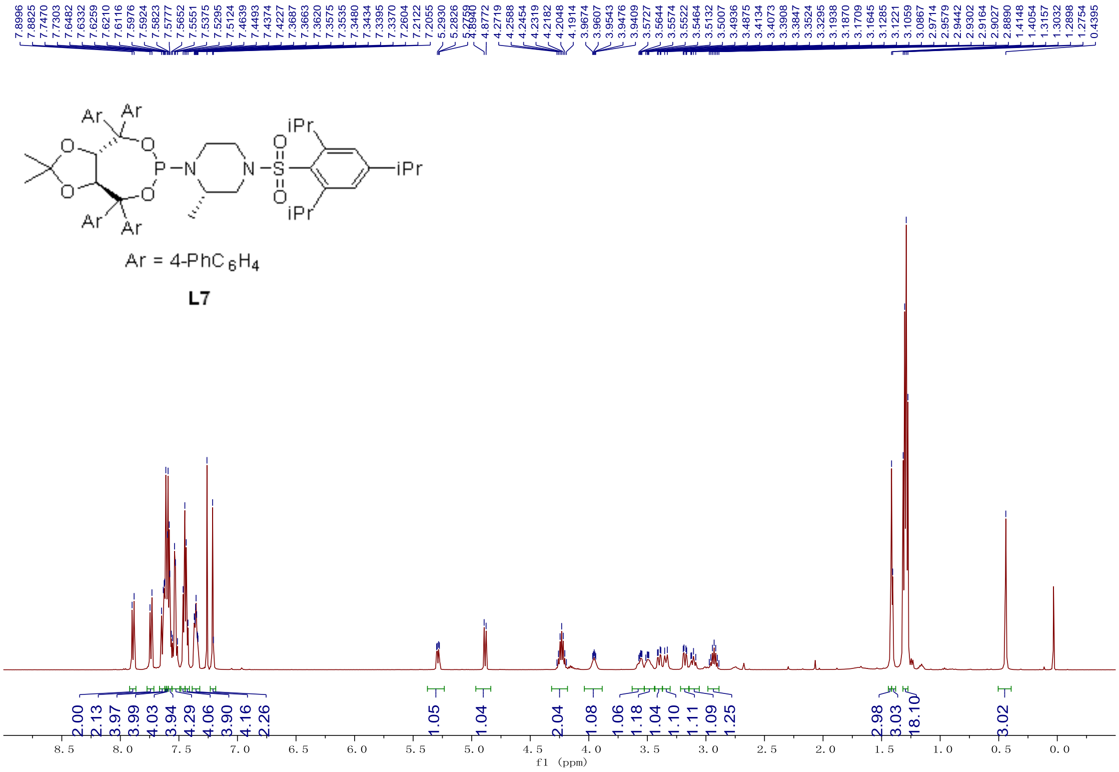

Supplementary Figure 17. ^{1}**H NMR** spectroscopy of **L7**

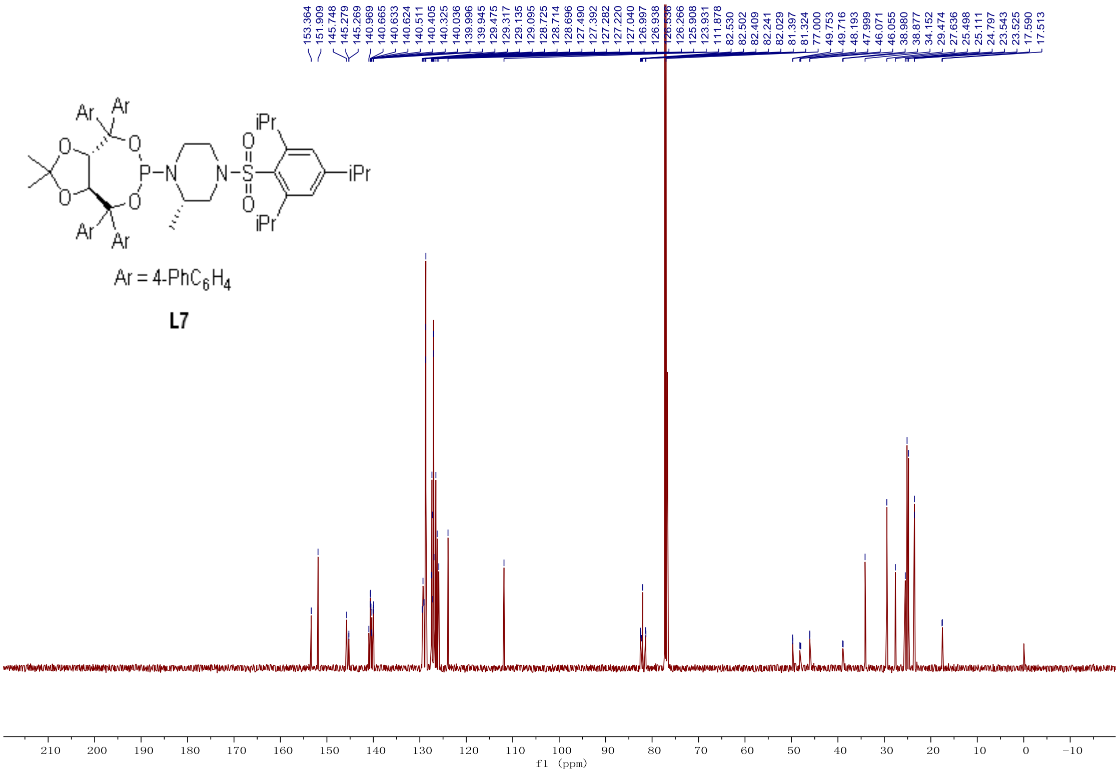

Supplementary Figure 18. ^{13}**C NMR** spectroscopy of **L7**

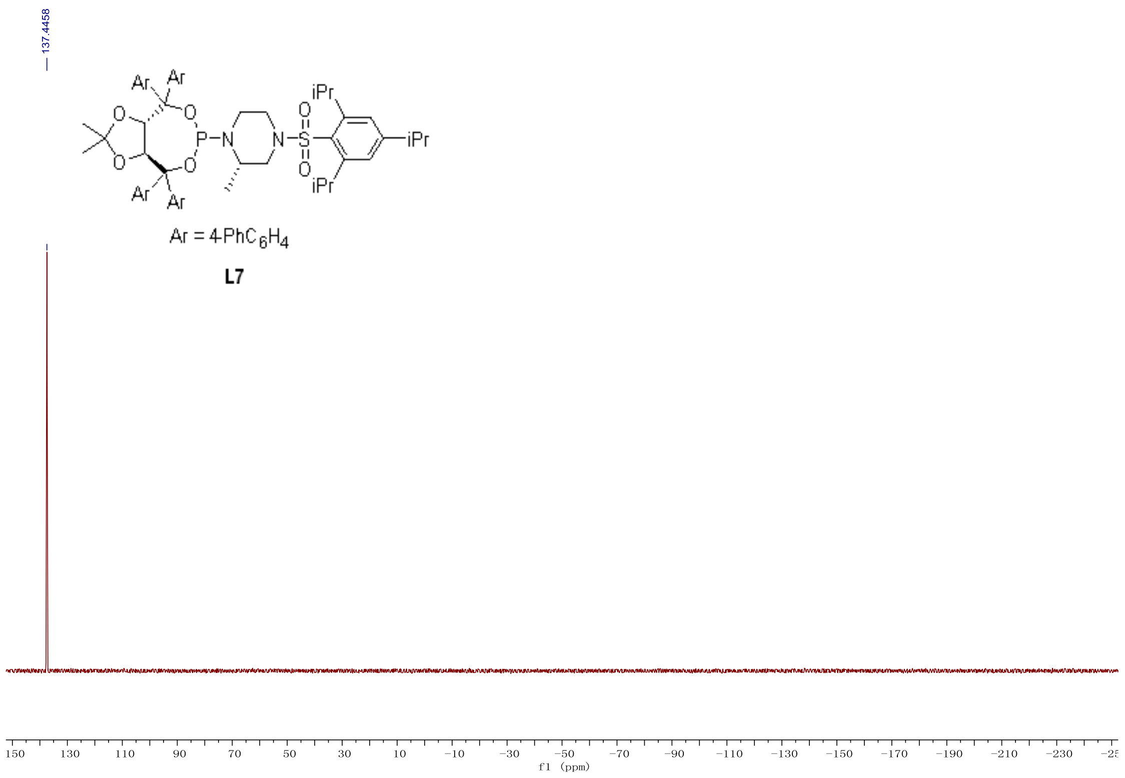

Supplementary Figure 19. ^{31}**P NMR** spectroscopy of **L7**

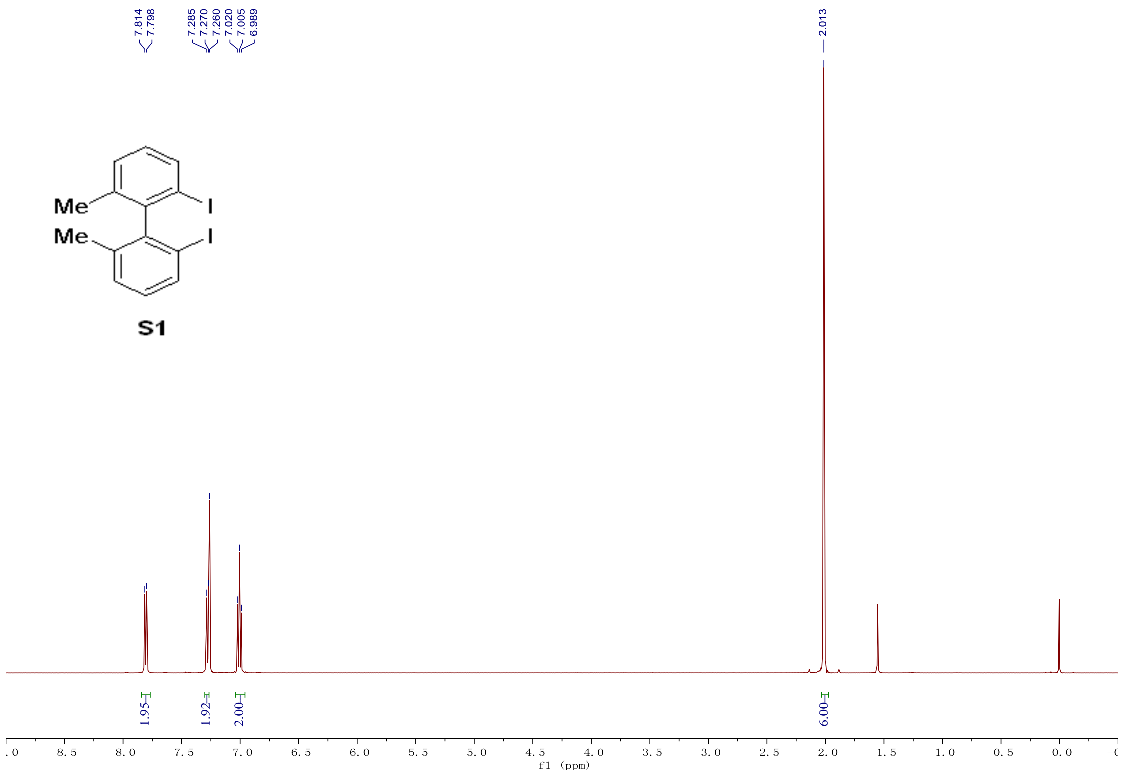

Supplementary Figure 20. ^{1}**H NMR** spectroscopy of **S1**

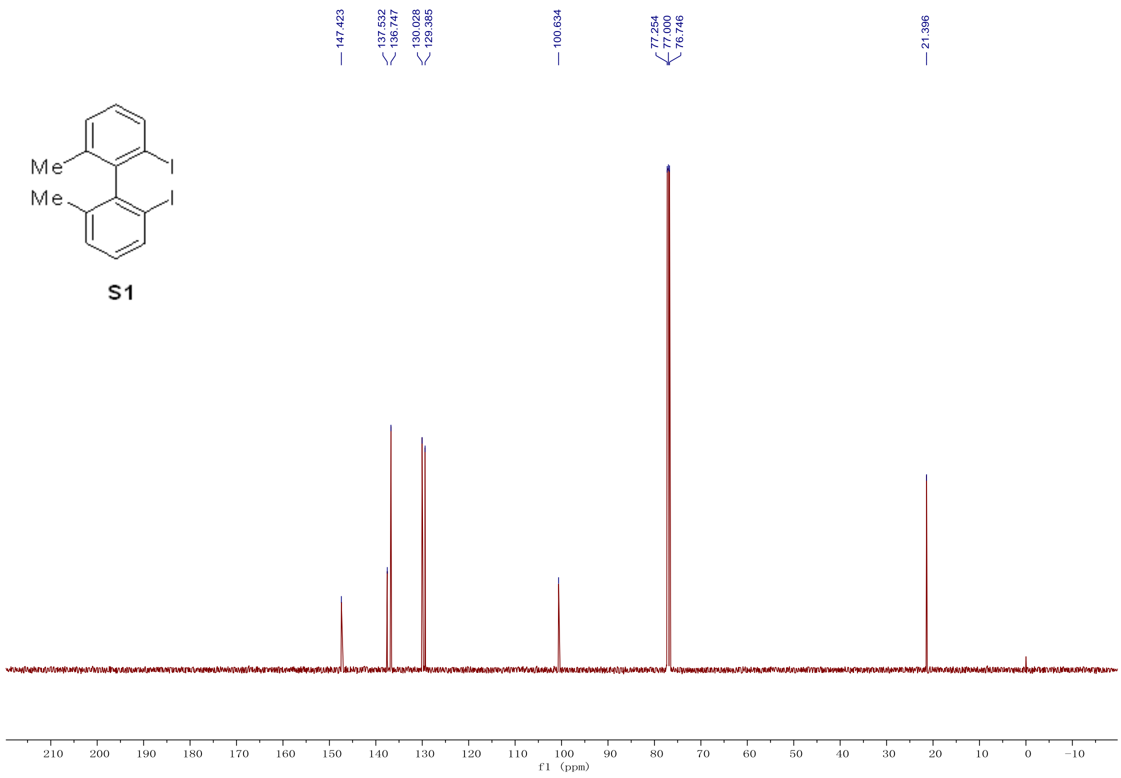

Supplementary Figure 21. ^{13}**C NMR** spectroscopy of **S1**

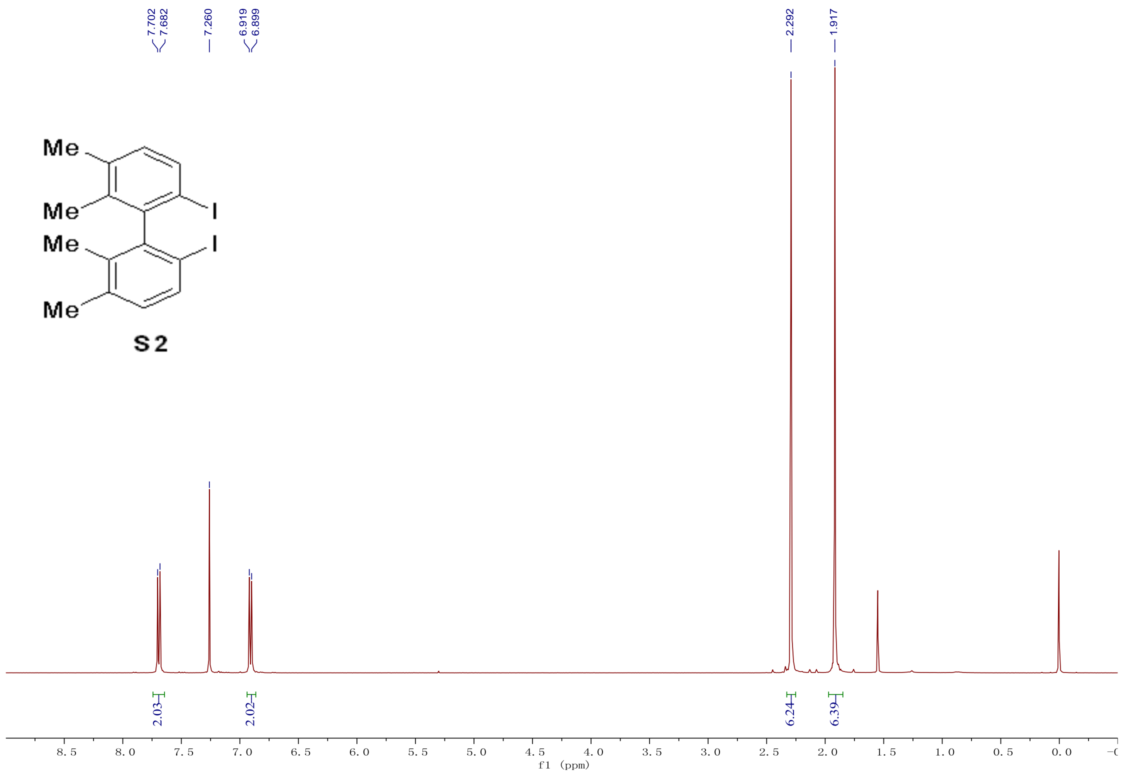

Supplementary Figure 22. ^{1}**H NMR** spectroscopy of **S2**

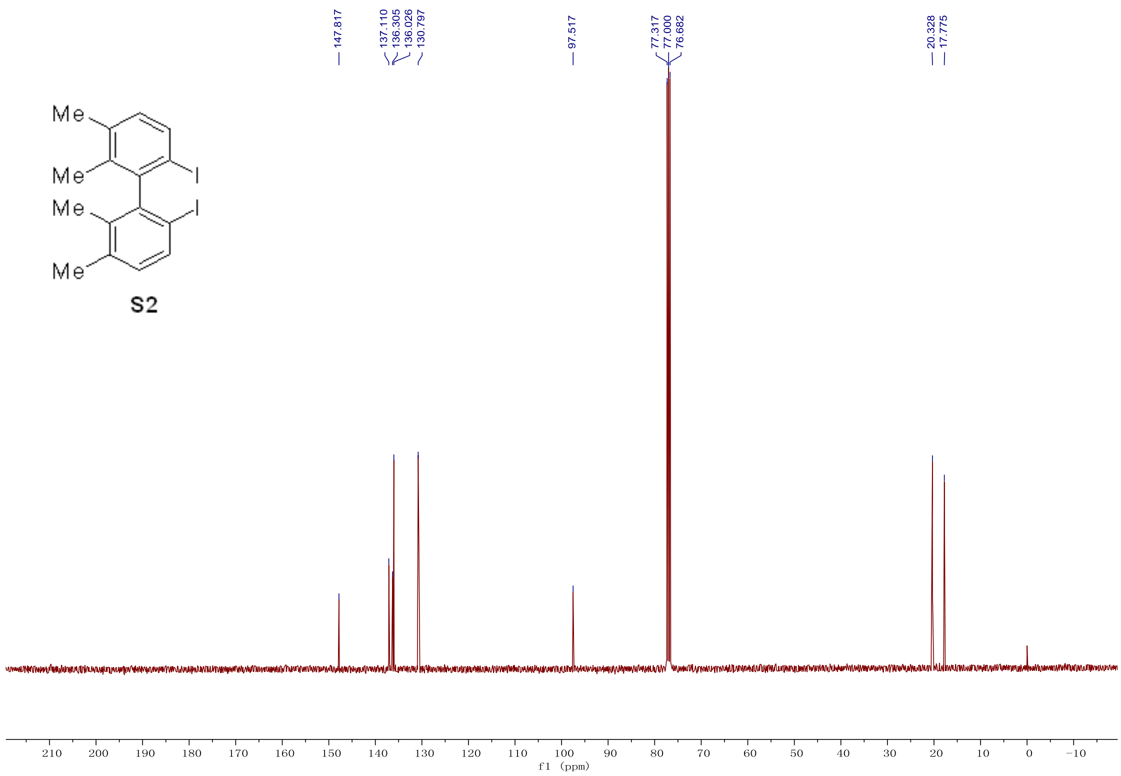

Supplementary Figure 23. ^{13}**C NMR** spectroscopy of **S2**

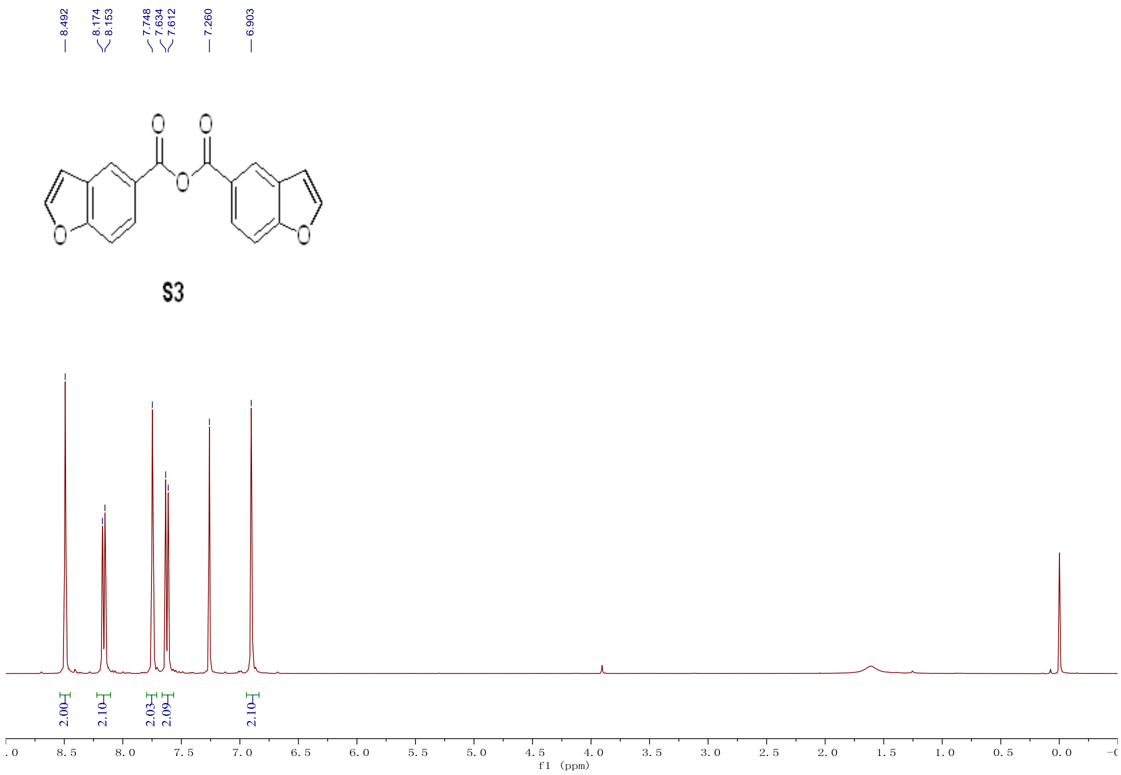

Supplementary Figure 24. ^{1}**H NMR** spectroscopy of **S3**

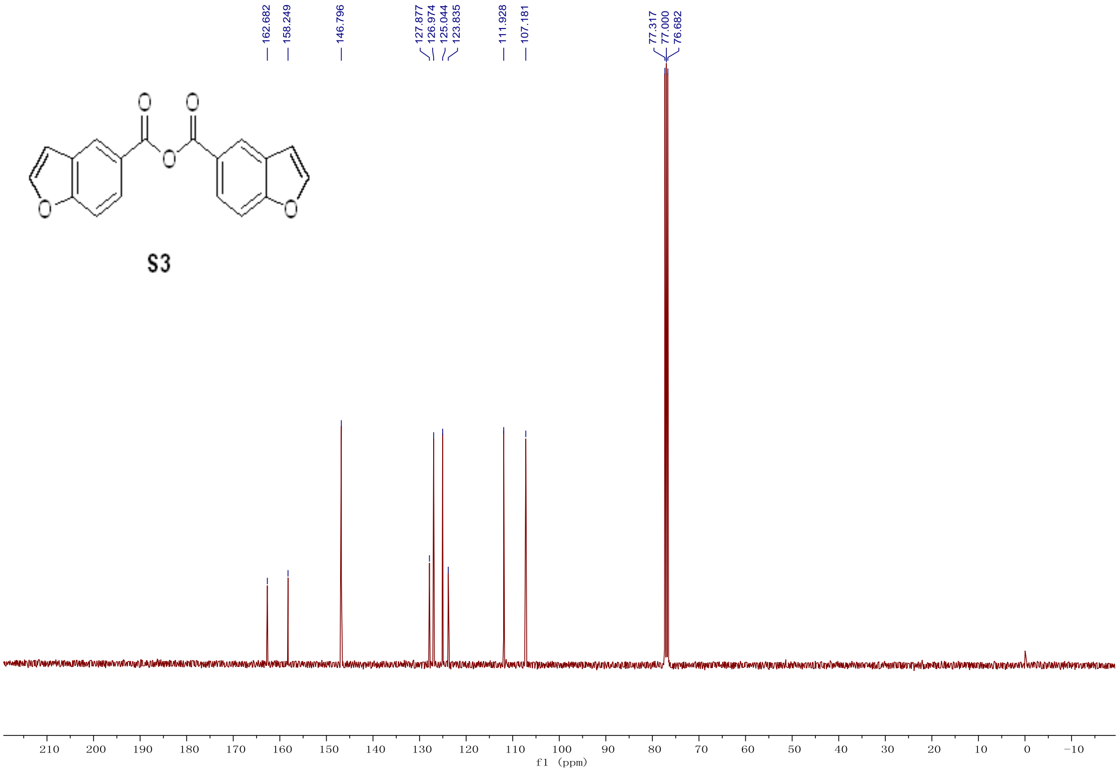

Supplementary Figure 25. ^{13}**C NMR** spectroscopy of **S3**

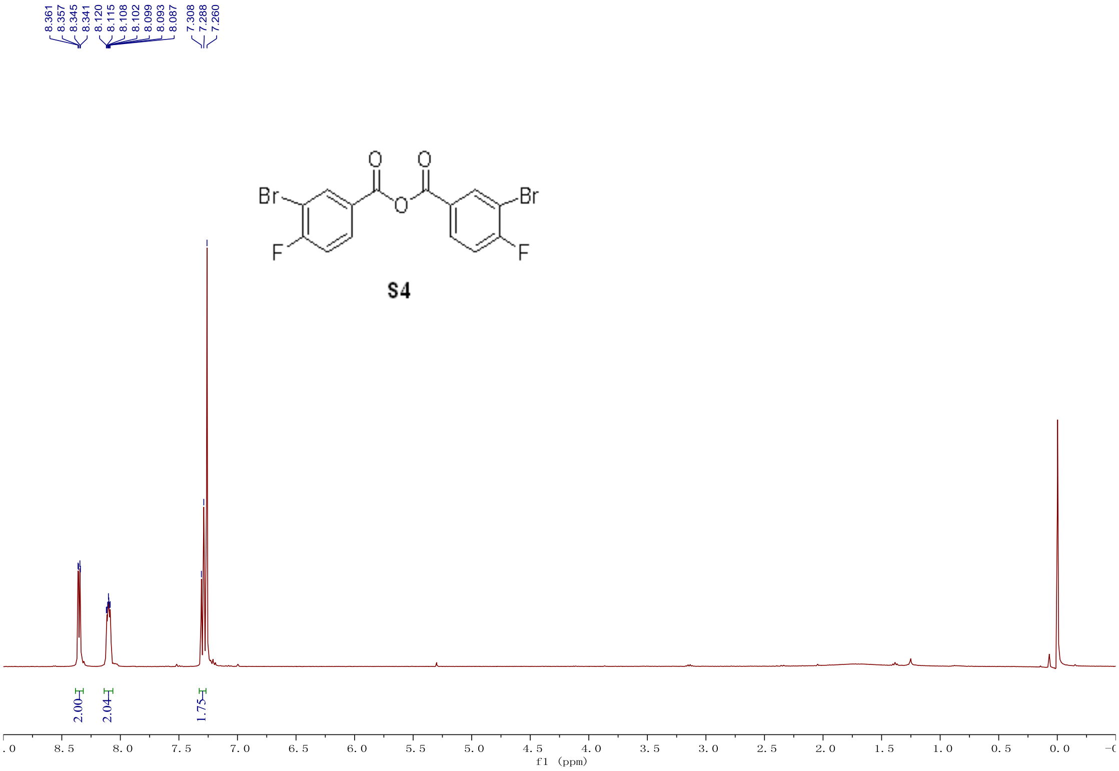

Supplementary Figure 26. ^{1}**H NMR** spectroscopy of **S4**

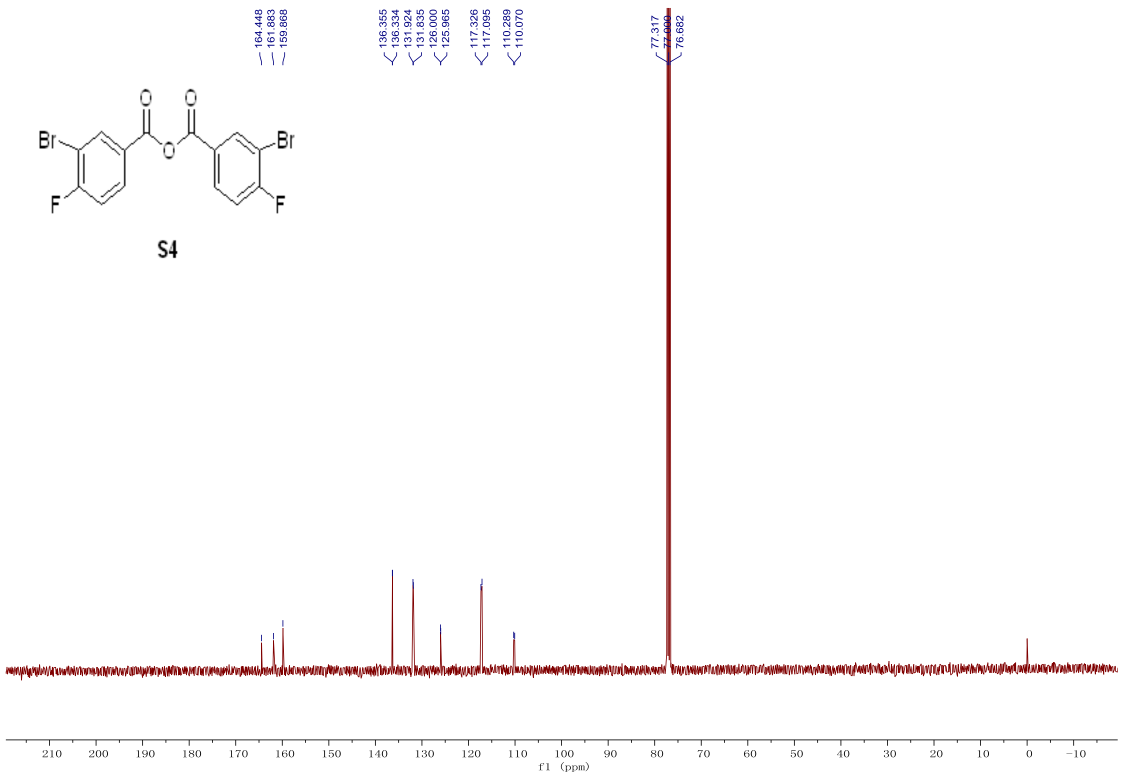

Supplementary Figure 27. ^{13}**C NMR** spectroscopy of **S4**

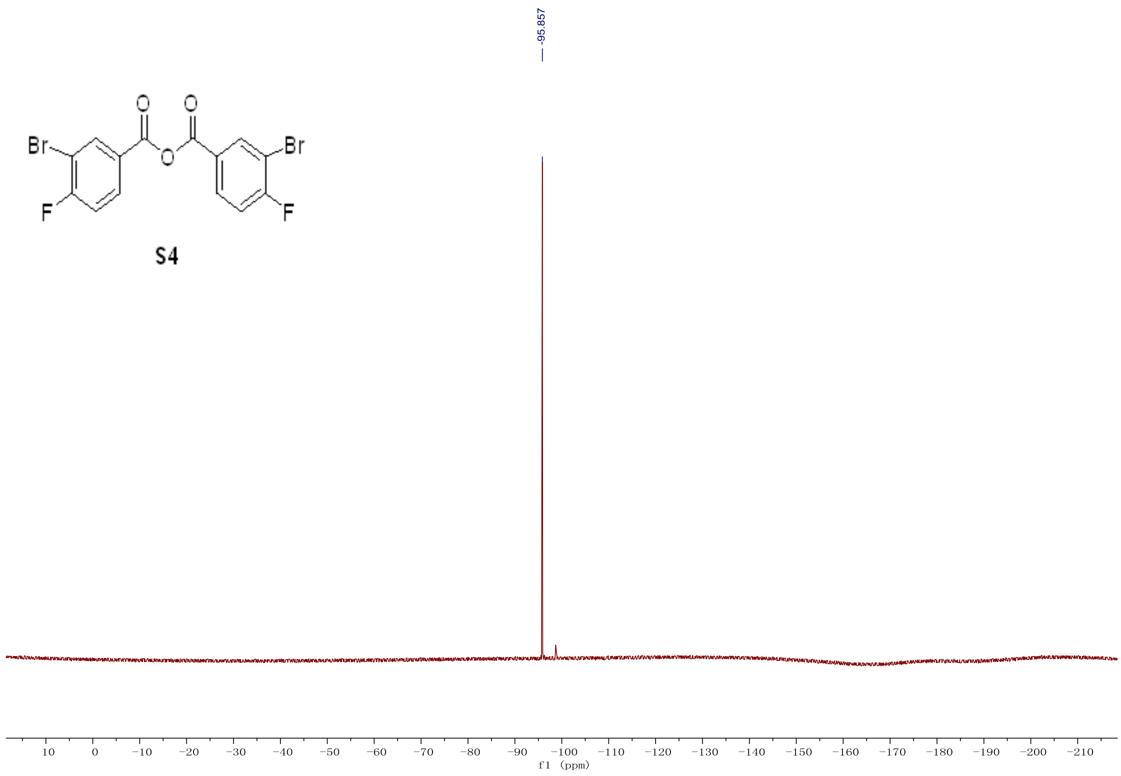

Supplementary Figure 28. ^{19}**F NMR** spectroscopy of **S4**

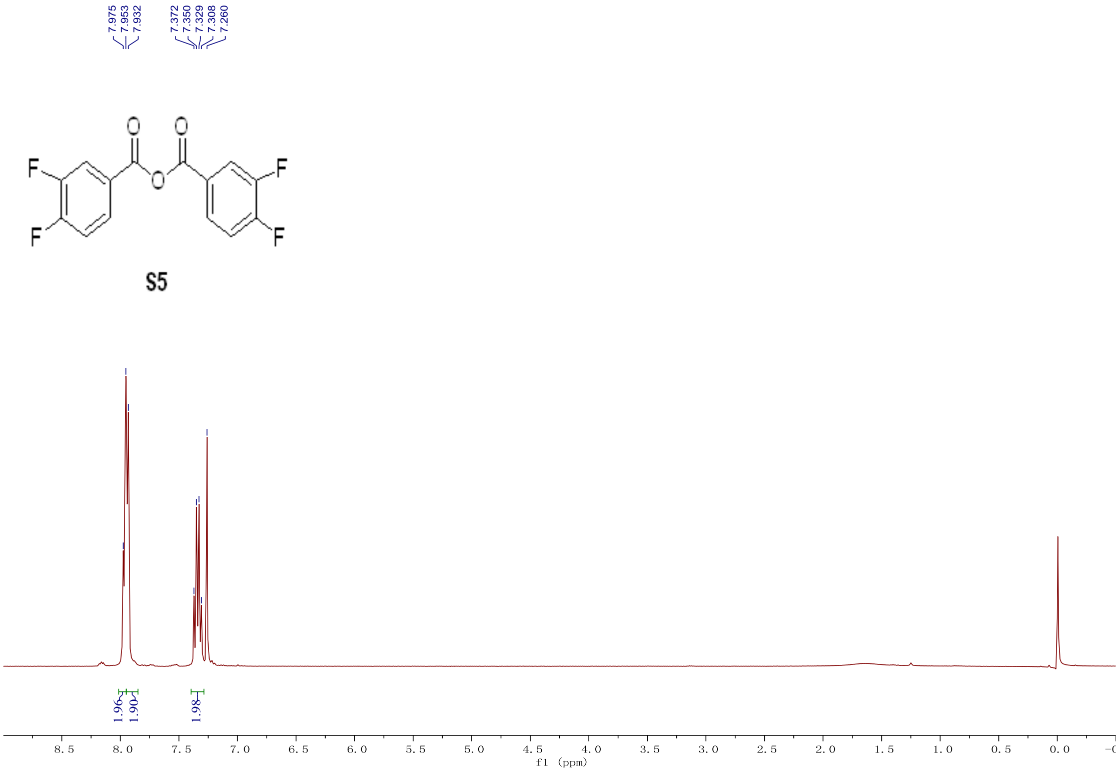

Supplementary Figure 29. ^{1}**H NMR** spectroscopy of **S5**

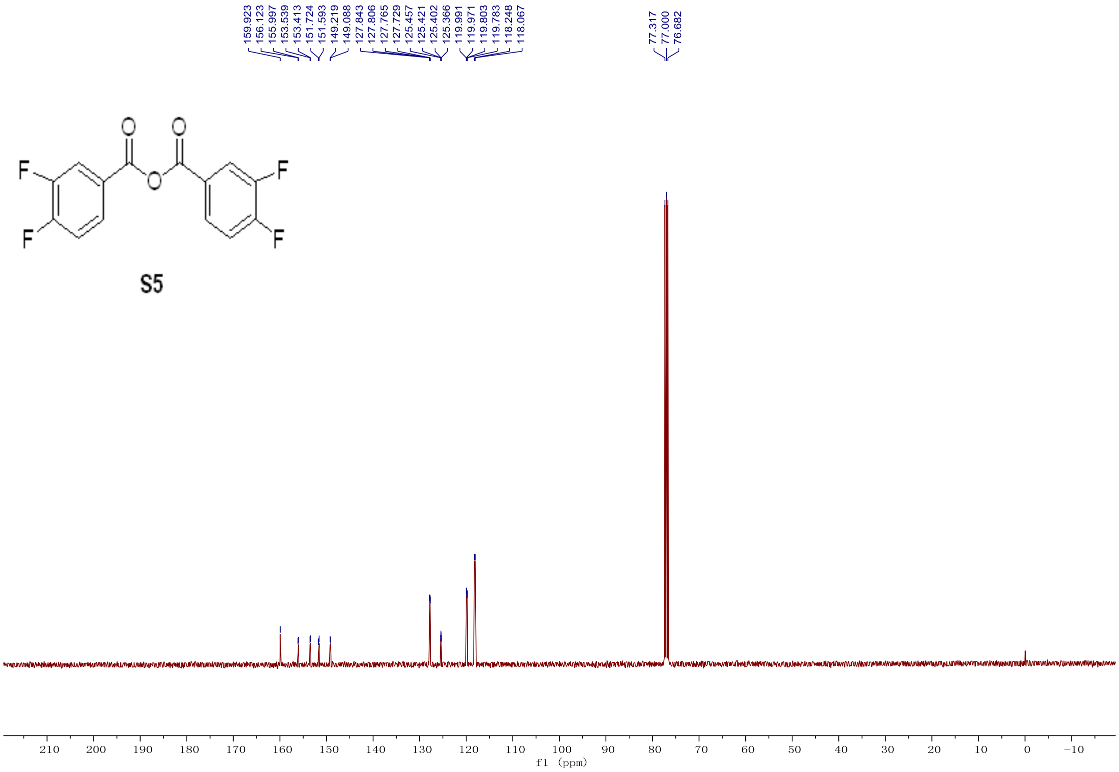

Supplementary Figure 30. ^{13}**C NMR** spectroscopy of **S5**

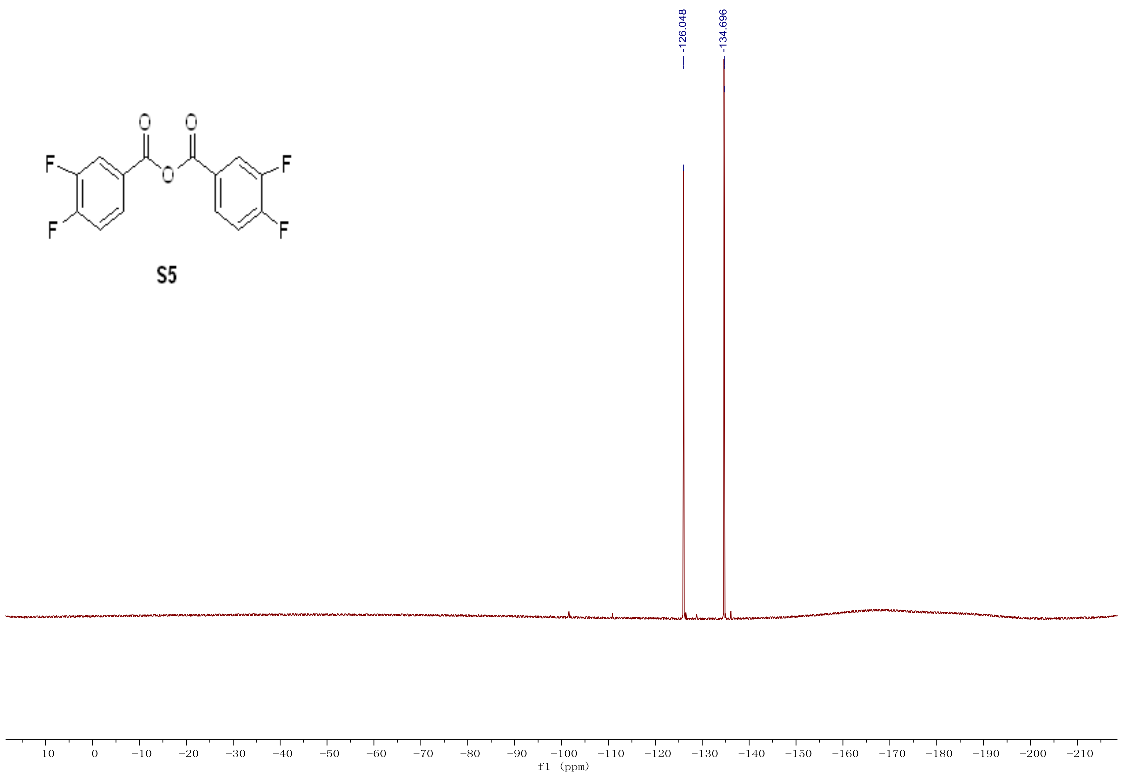

Supplementary Figure 31. ^{19}**F NMR** spectroscopy of **S5**

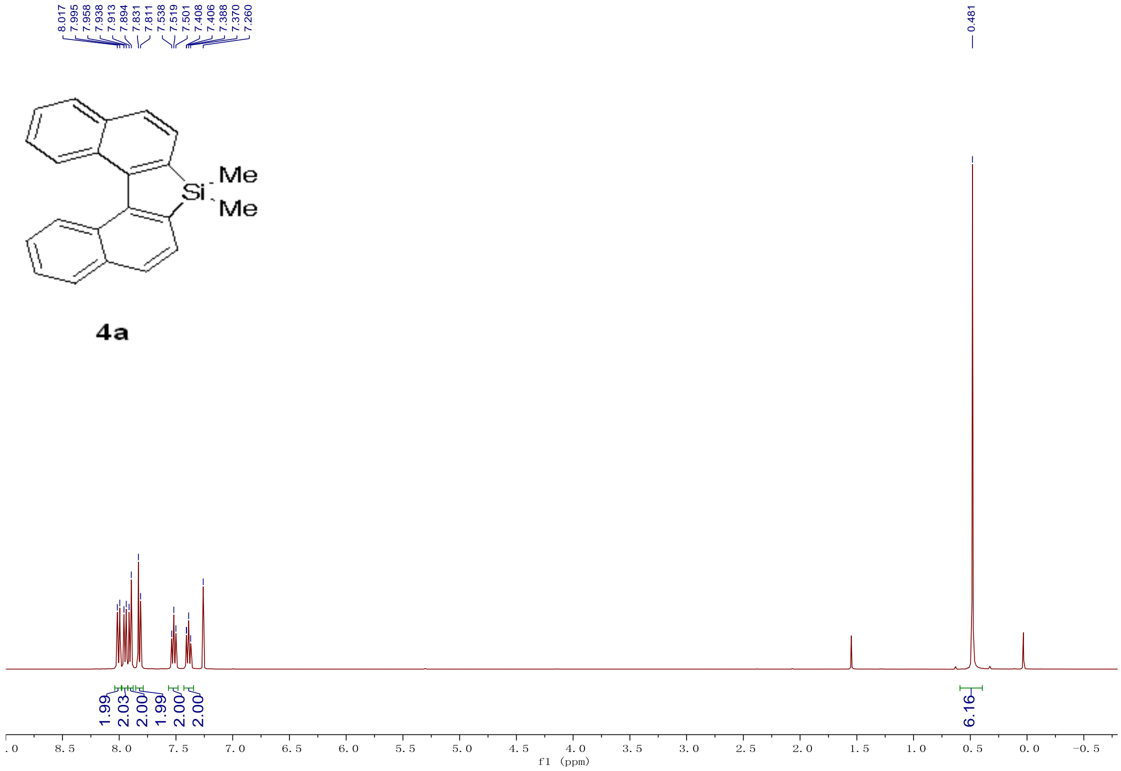

Supplementary Figure 32. ^{1}**H NMR** spectroscopy of **4a**

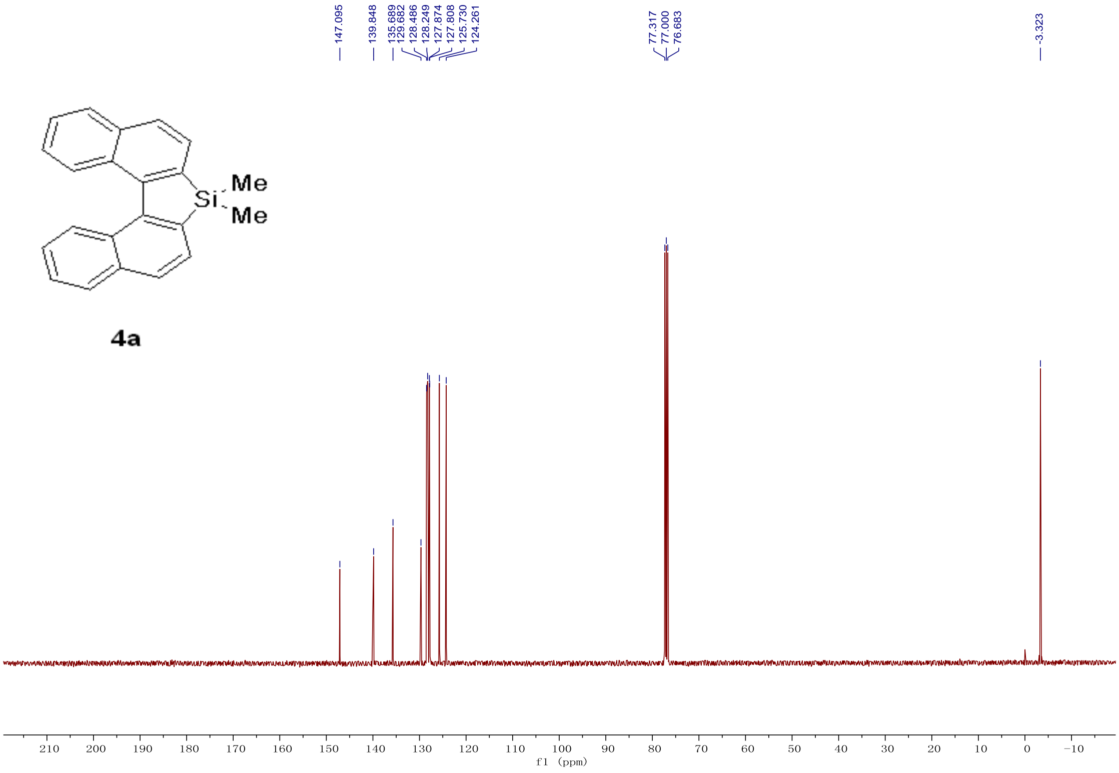

Supplementary Figure 33. ^{13}**C NMR** spectroscopy of **4a**

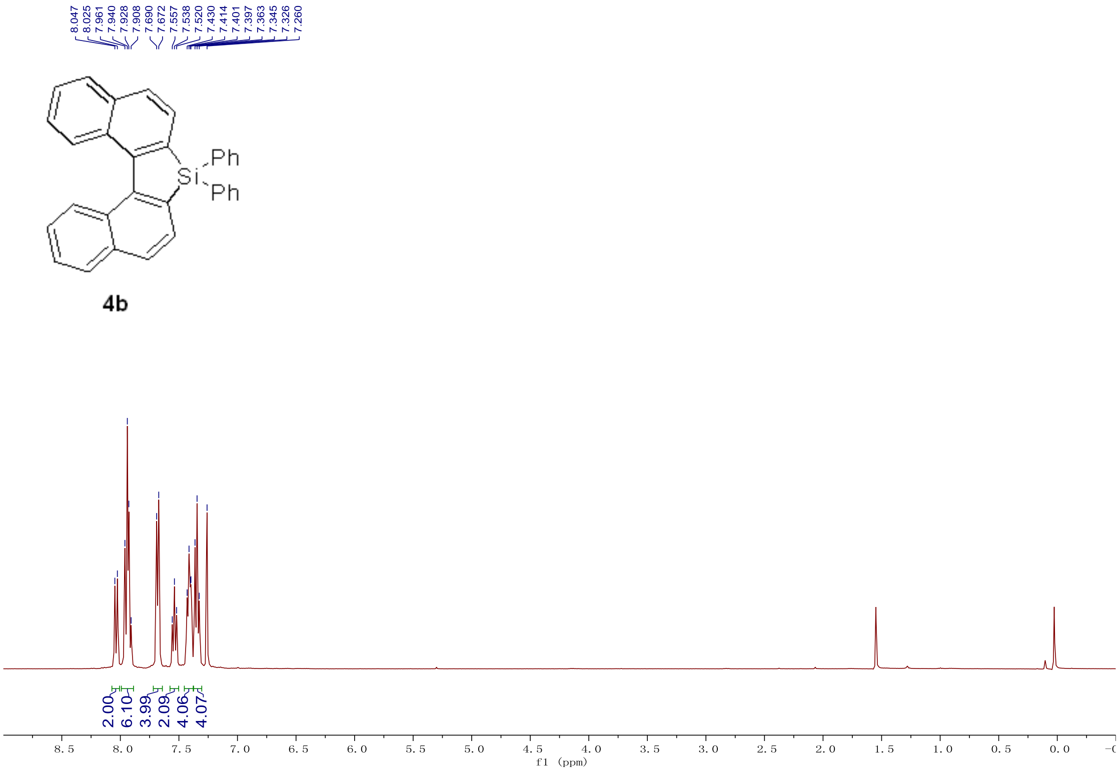

Supplementary Figure 34. ^{1}**H NMR** spectroscopy of **4b**

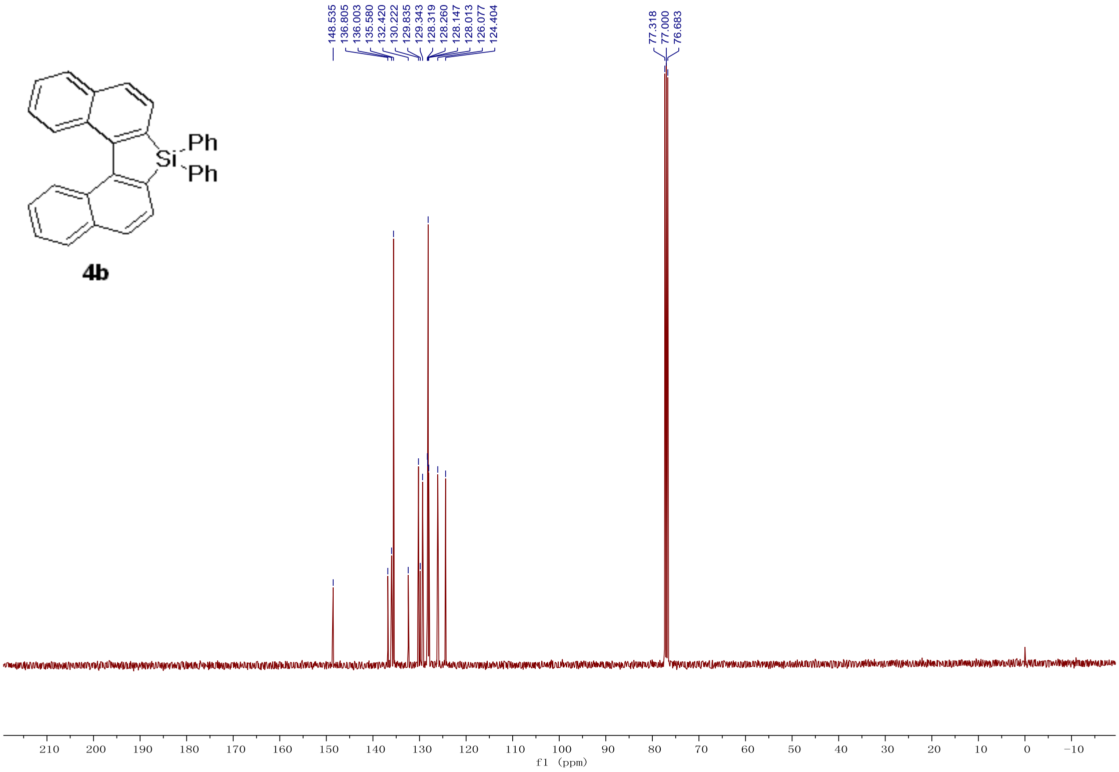

Supplementary Figure 35. ^{13}**C NMR** spectroscopy of **4b**

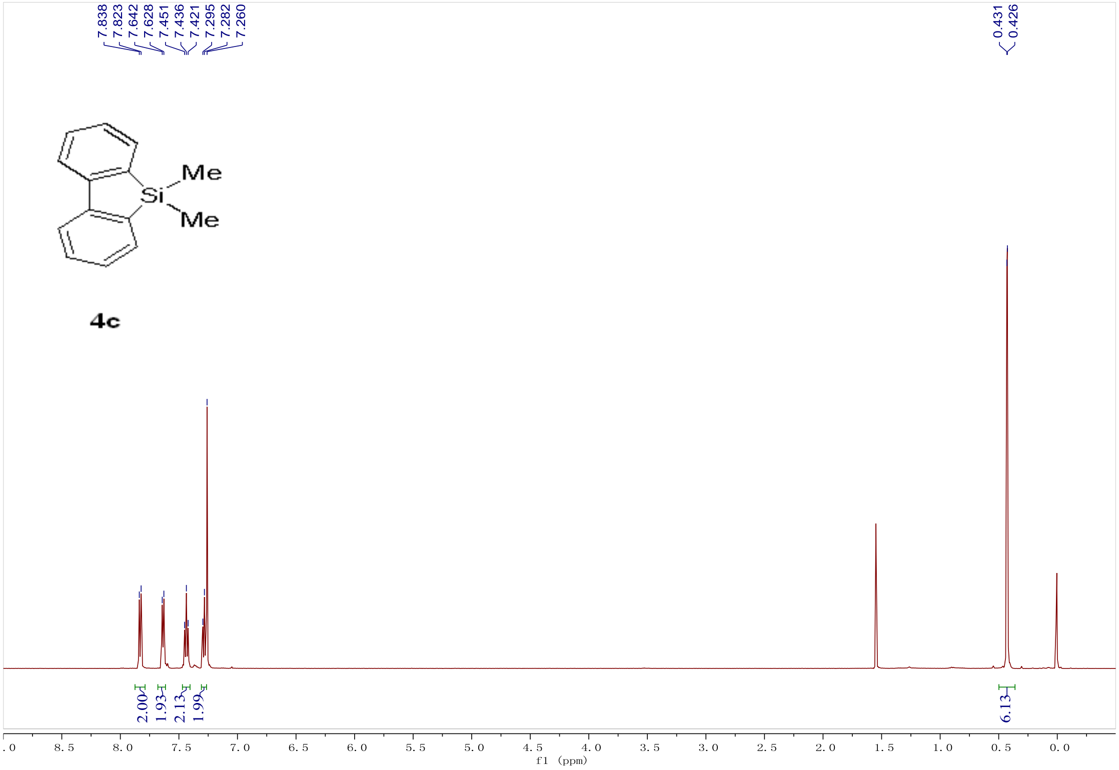

Supplementary Figure 36. ^{1}**H NMR** spectroscopy of **4c**

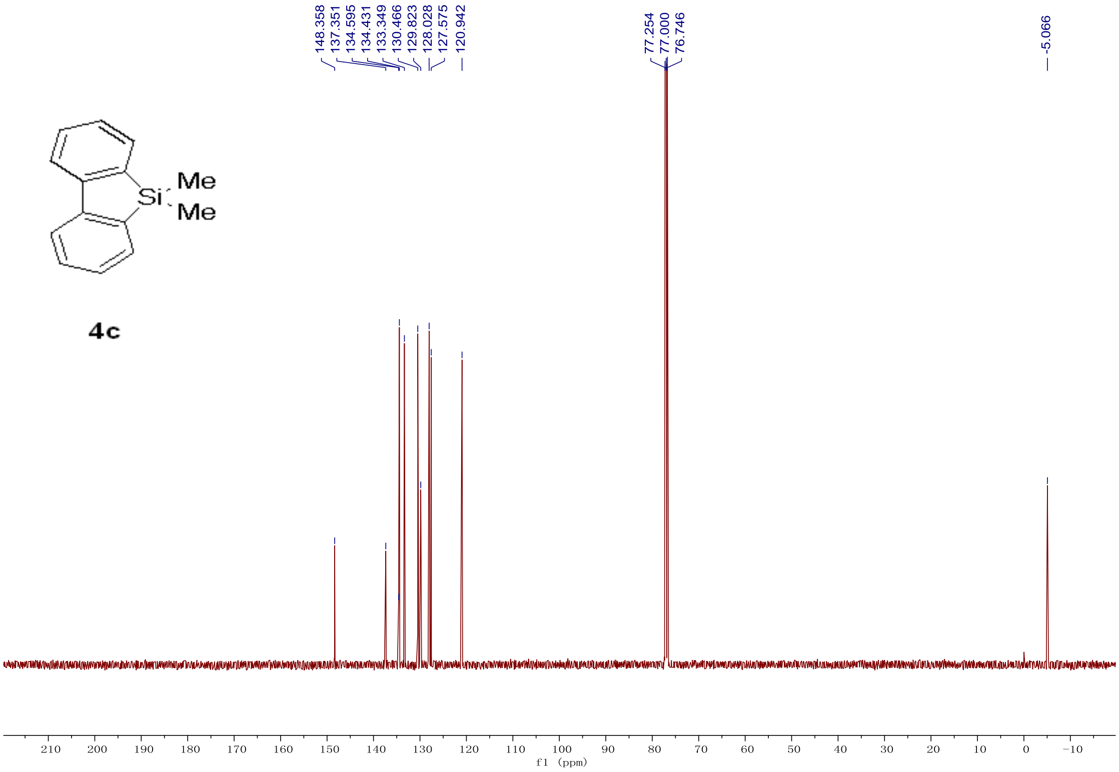

Supplementary Figure 37. ^{13}**C NMR** spectroscopy of **4c**

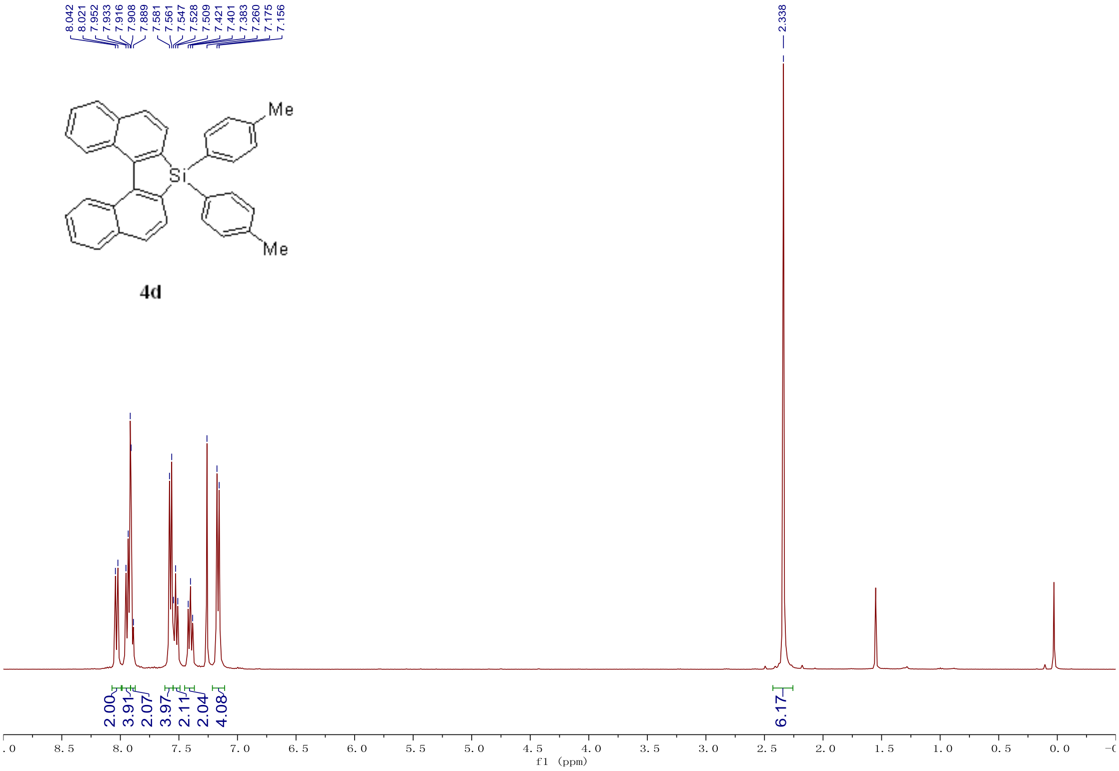

Supplementary Figure 38. ^{1}**H NMR** spectroscopy of **4d**

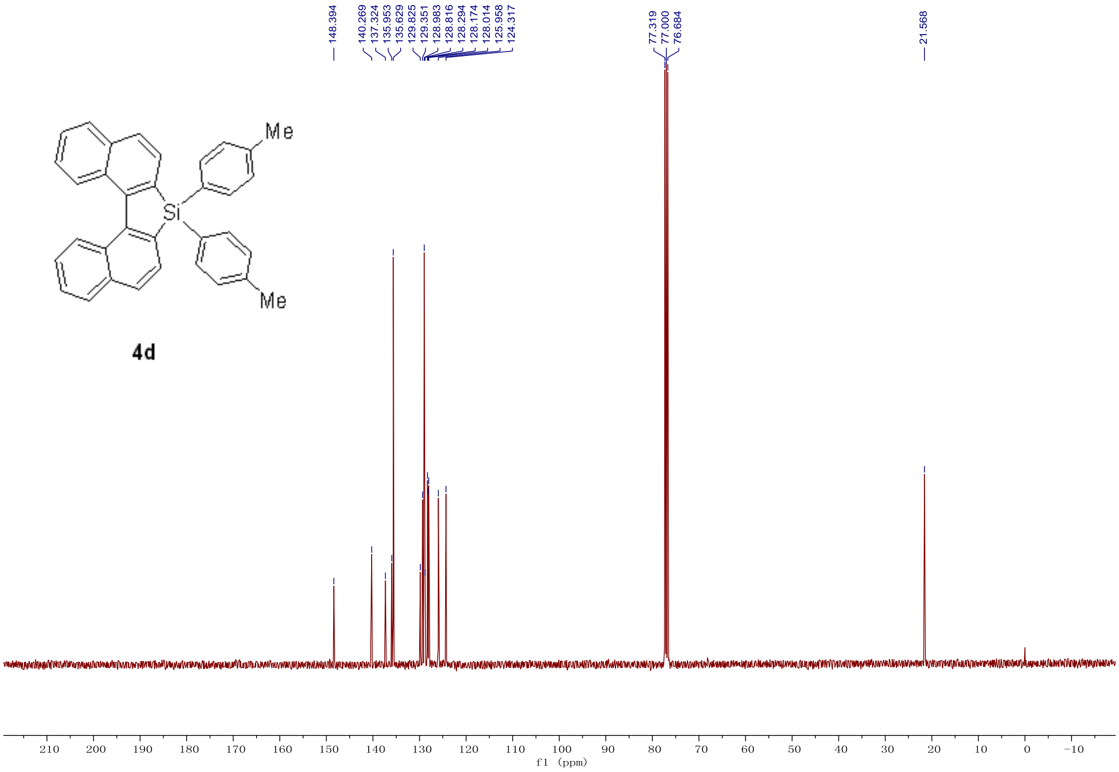

Supplementary Figure 39. ^{13}C NMR spectroscopy of **4d**

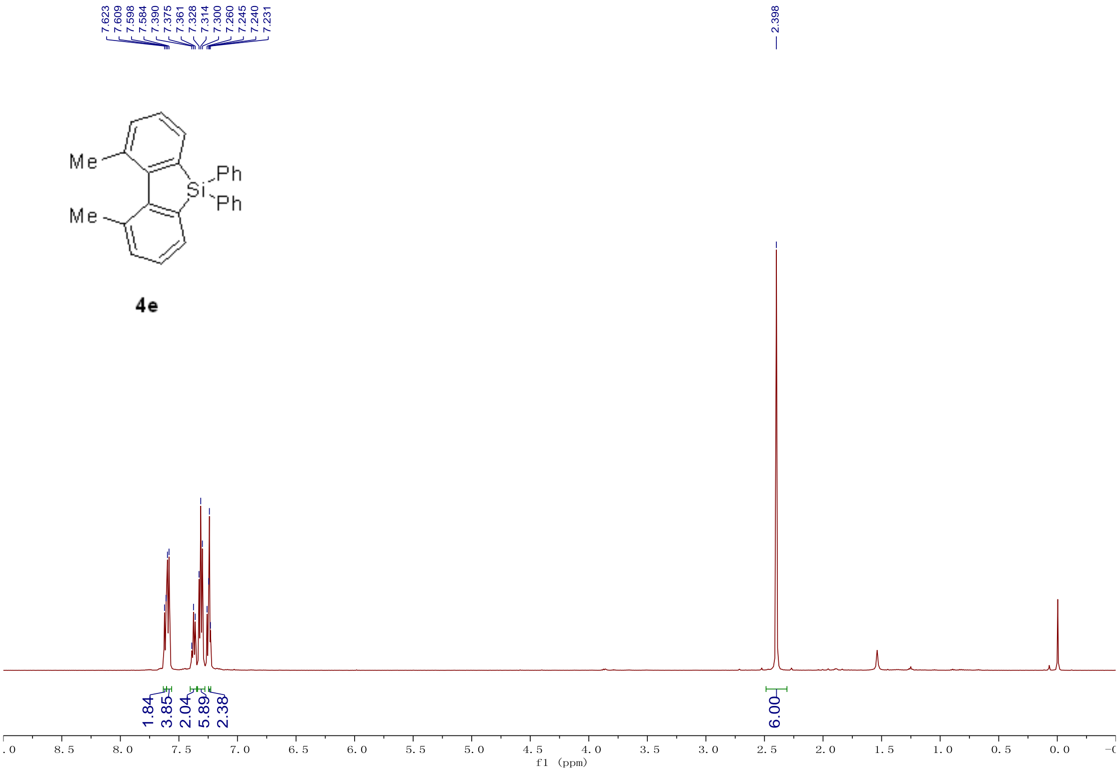

Supplementary Figure 40. ^{1}H NMR spectroscopy of **4e**

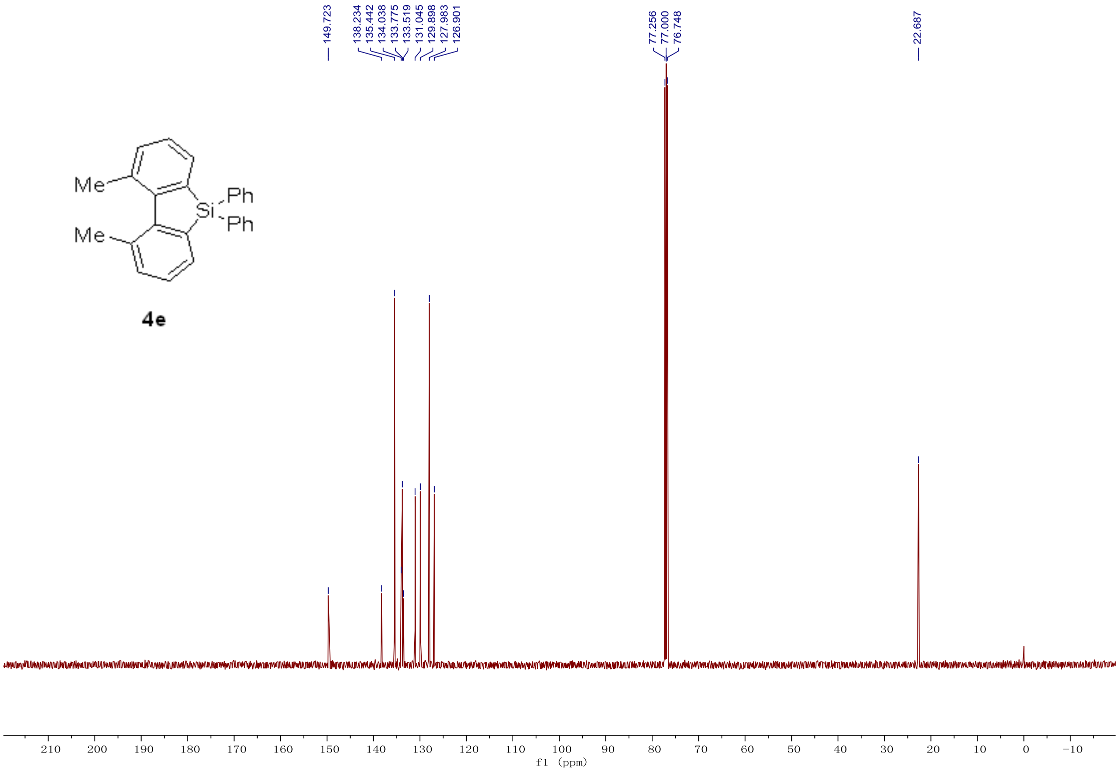

Supplementary Figure 41. ^{13}**C NMR** spectroscopy of **4e**

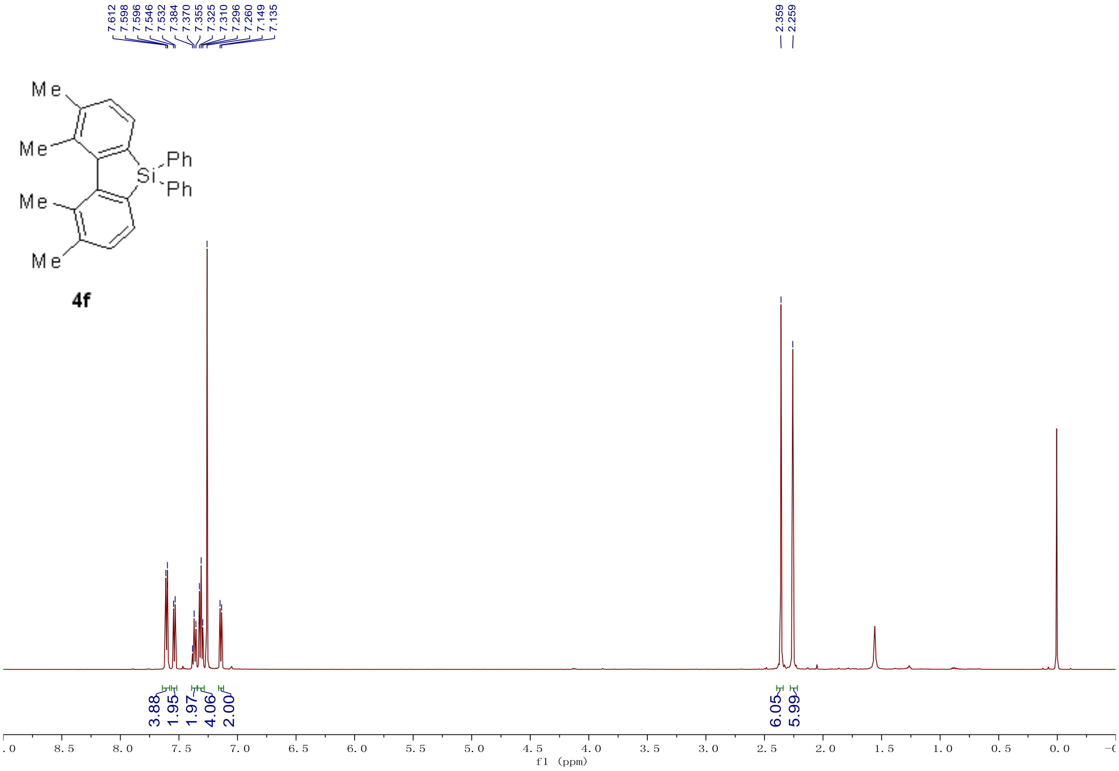

Supplementary Figure 42. ^{1}**H NMR** spectroscopy of **4f**

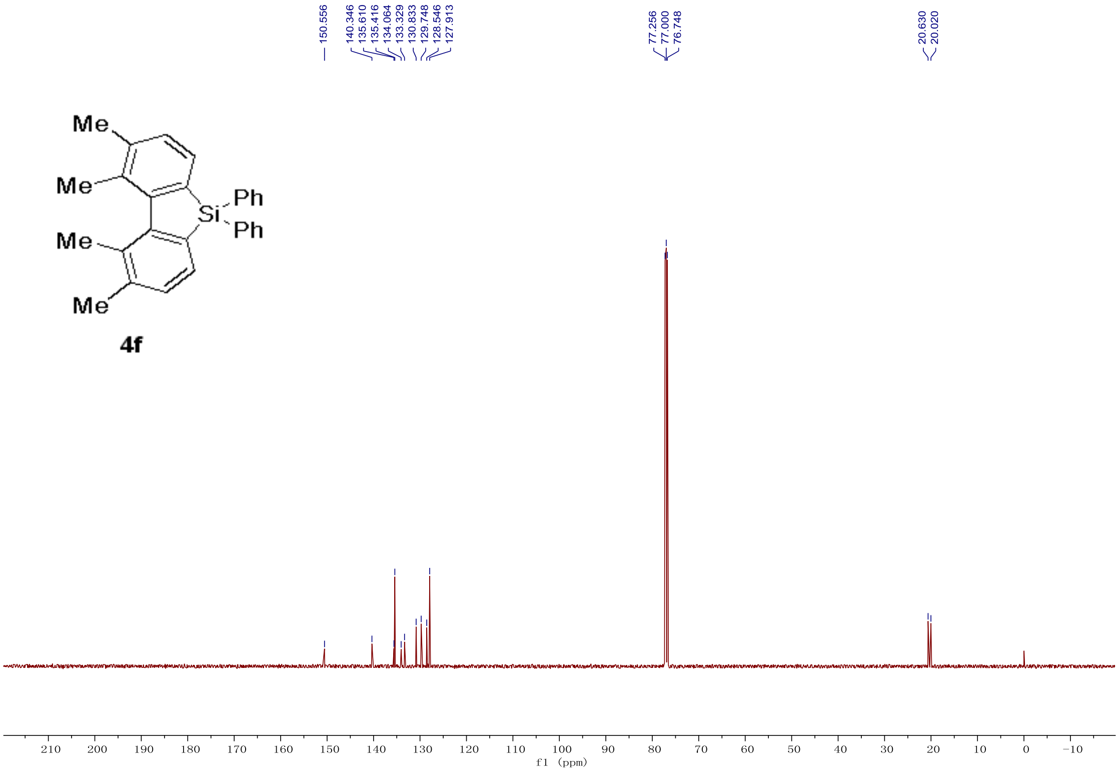

Supplementary Figure 43. ^{13}**C NMR** spectroscopy of **4f**

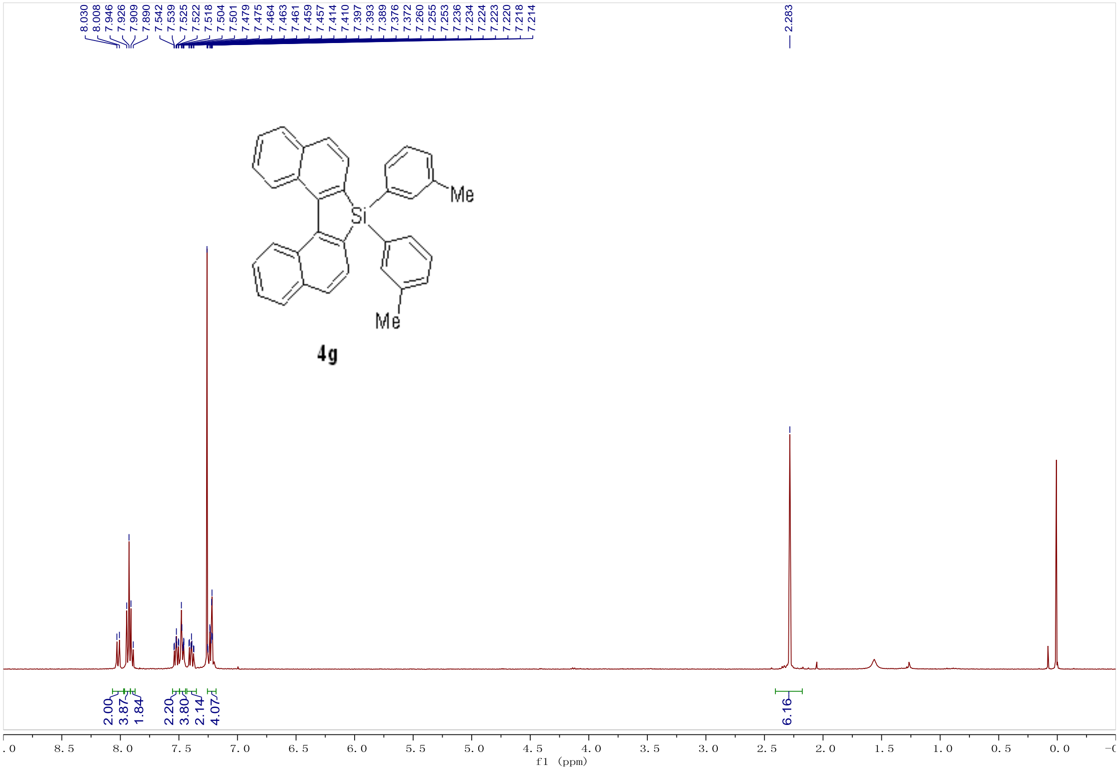

Supplementary Figure 44. ^{1}**H NMR** spectroscopy of **4g**

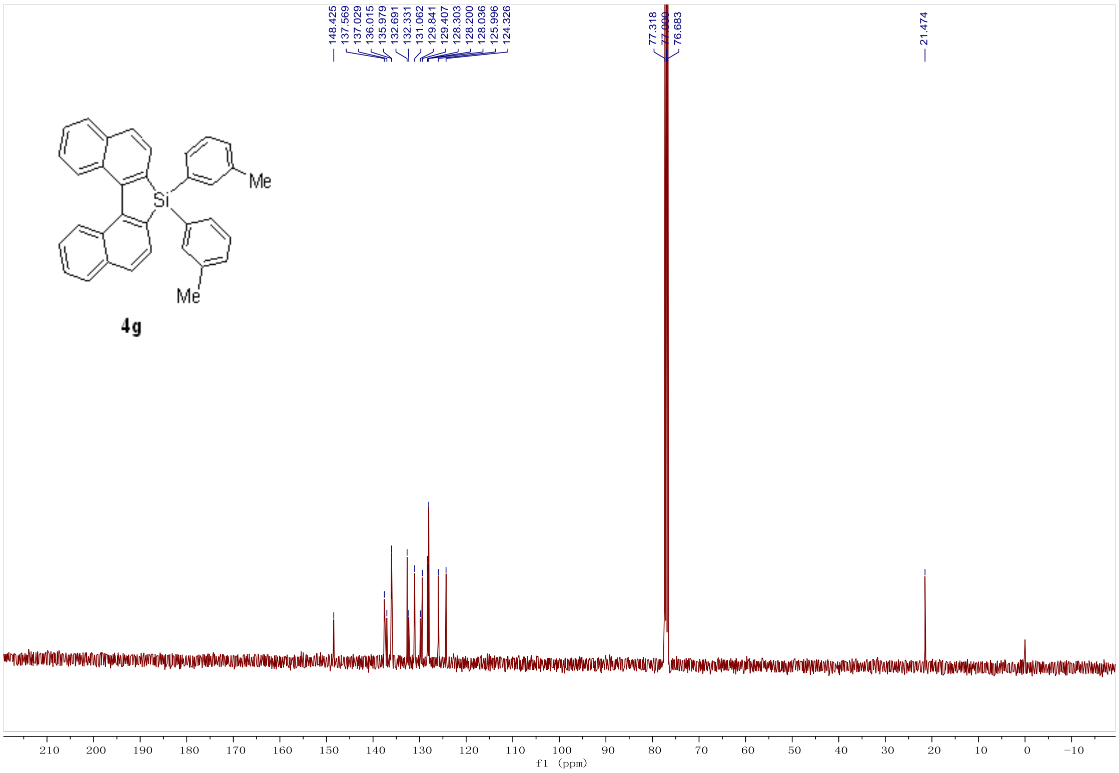

Supplementary Figure 45. ^{13}C NMR spectroscopy of **4g**

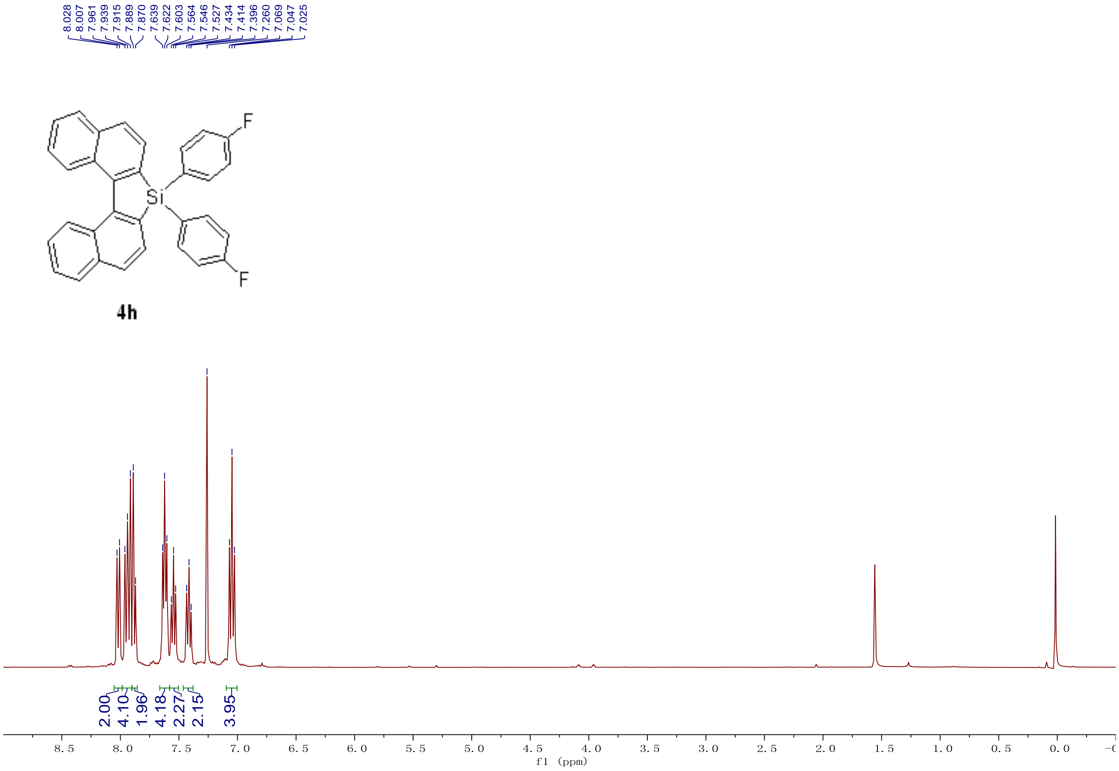

Supplementary Figure 46. ^{1}H NMR spectroscopy of **4h**

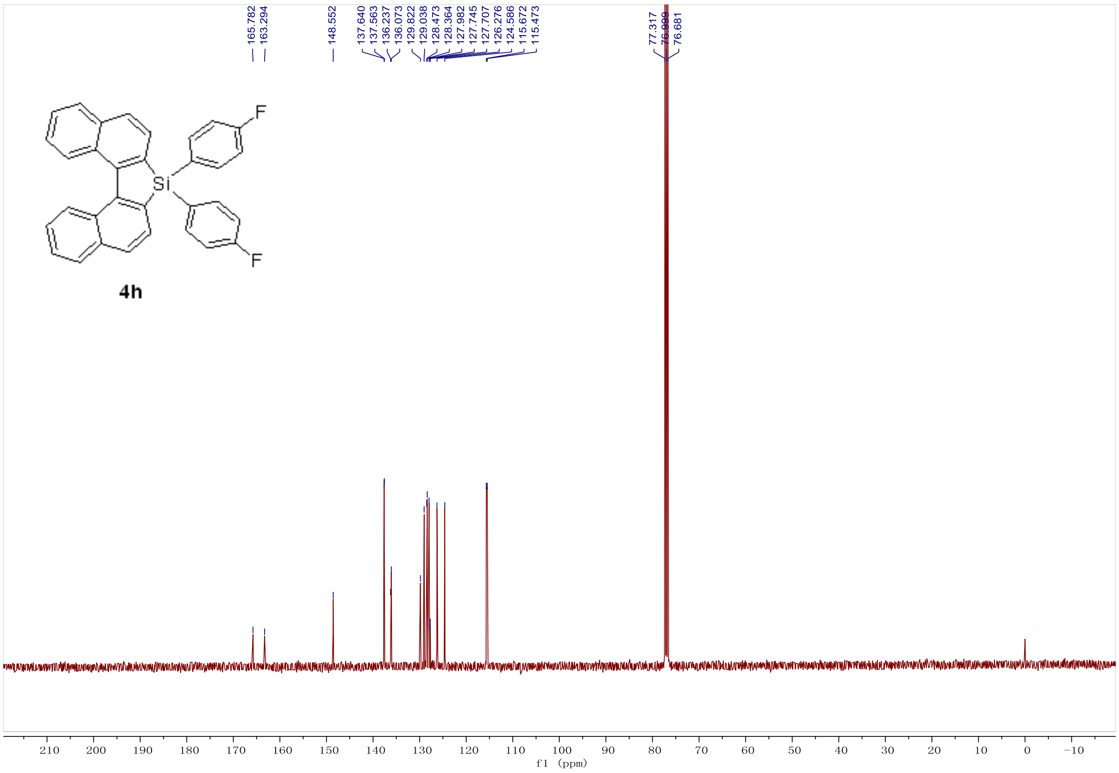

Supplementary Figure 47. ^{13}**C NMR** spectroscopy of **4h**

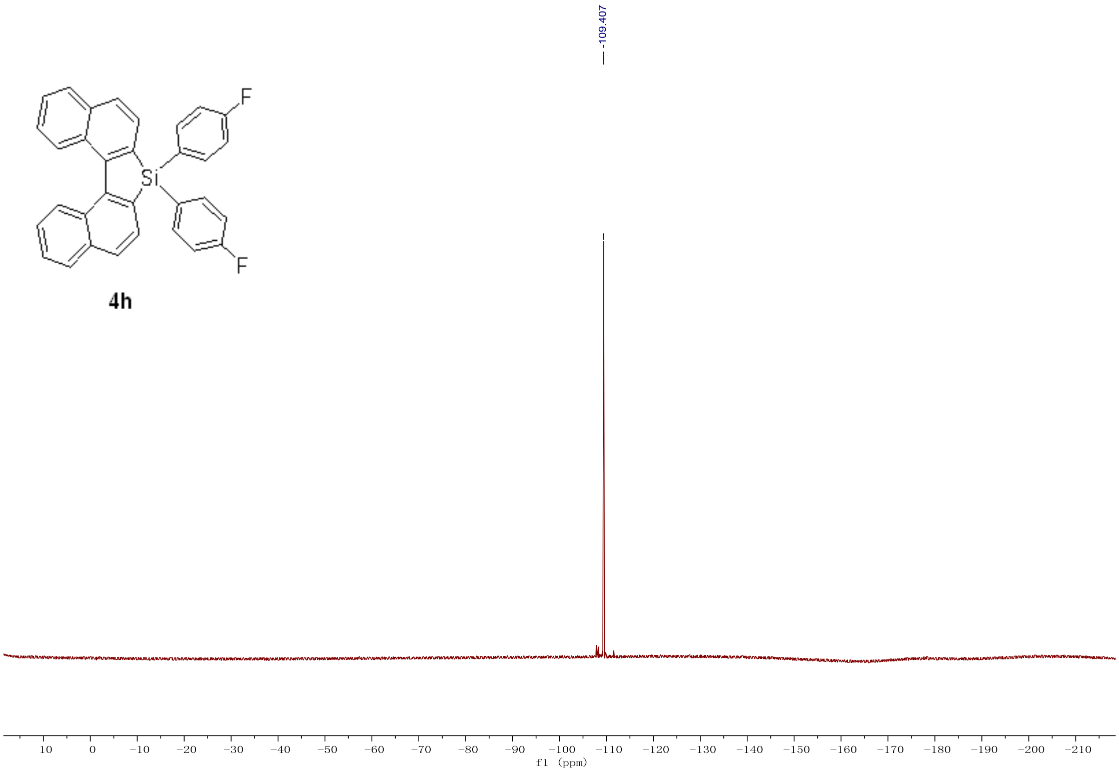

Supplementary Figure 48. ^{19}**F NMR** spectroscopy of **4h**

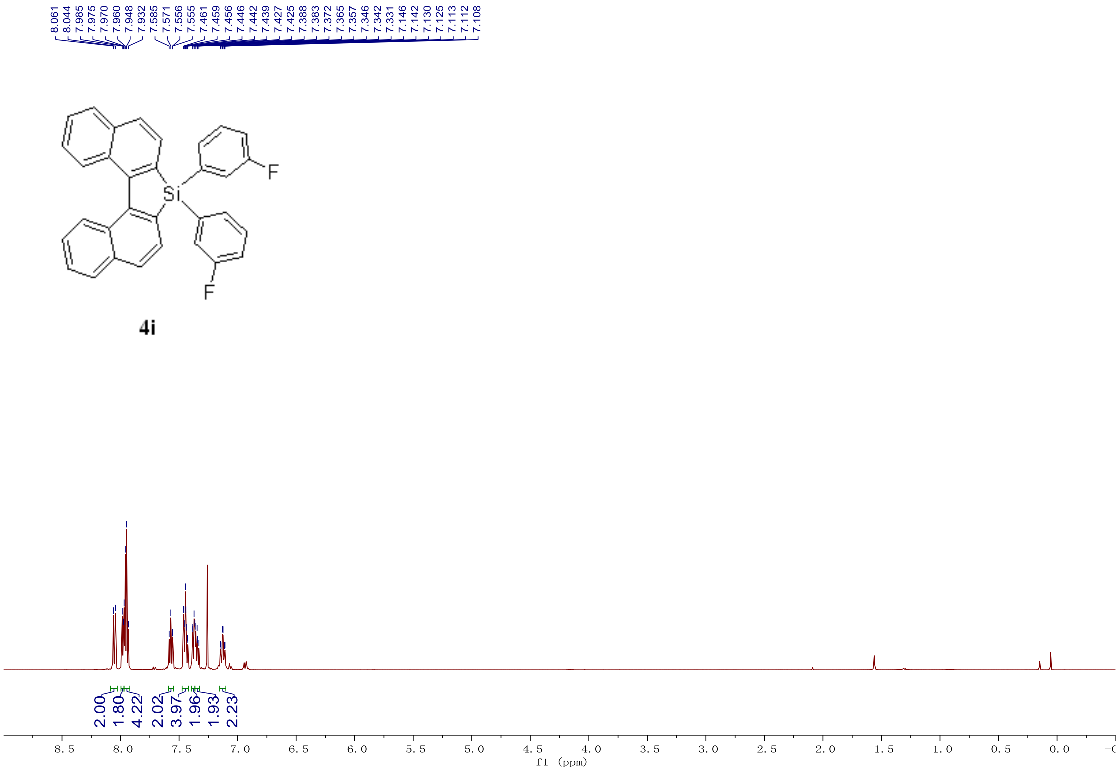

Supplementary Figure 49. ^{1}**H NMR** spectroscopy of **4i**

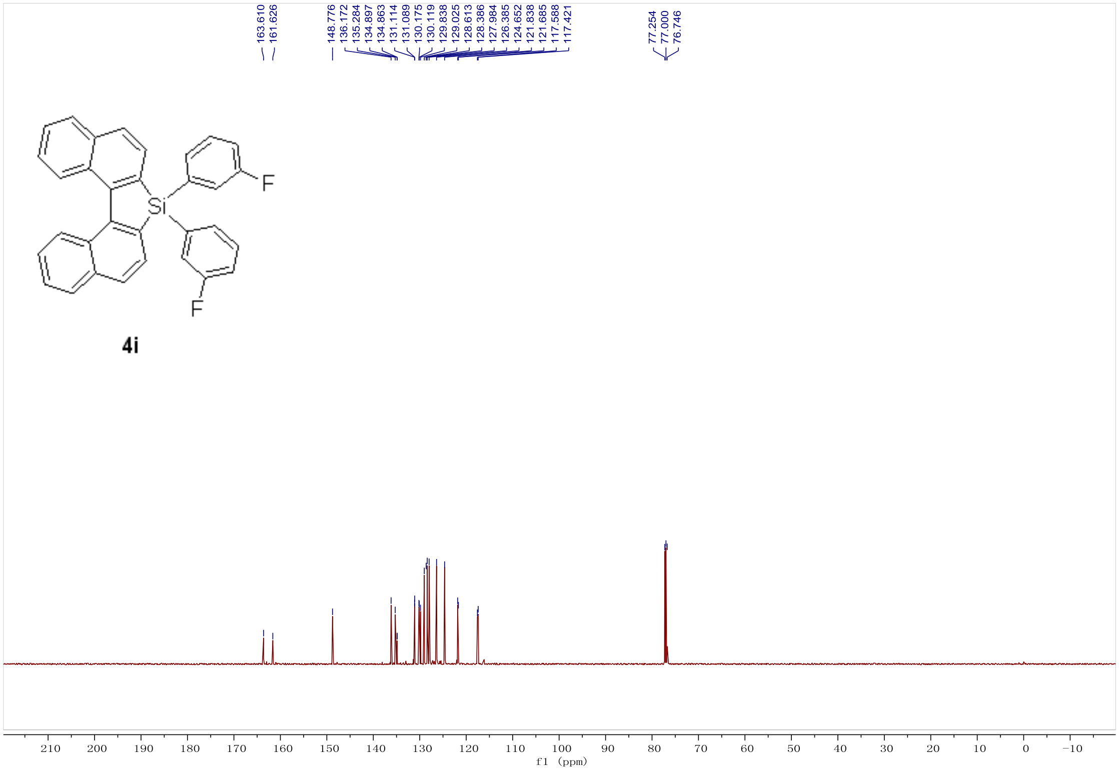

Supplementary Figure 50. ^{13}**C NMR** spectroscopy of **4i**

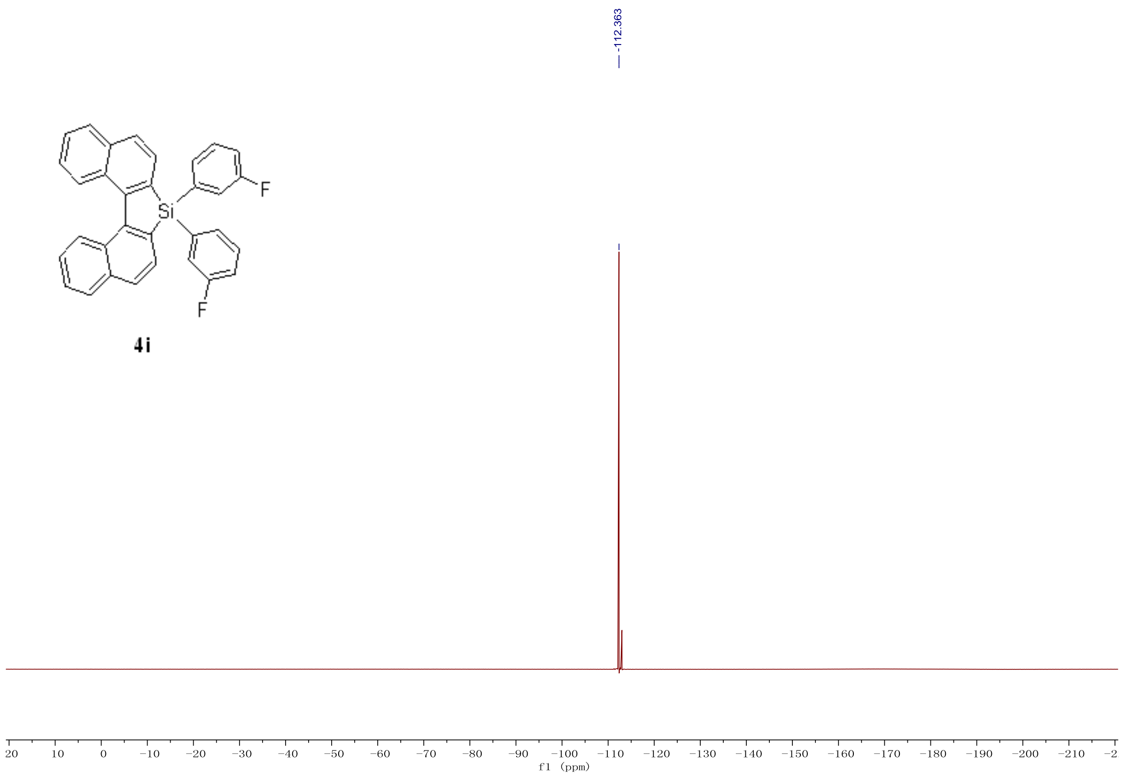

Supplementary Figure 51. ^{19}**F NMR** spectroscopy of **4i**

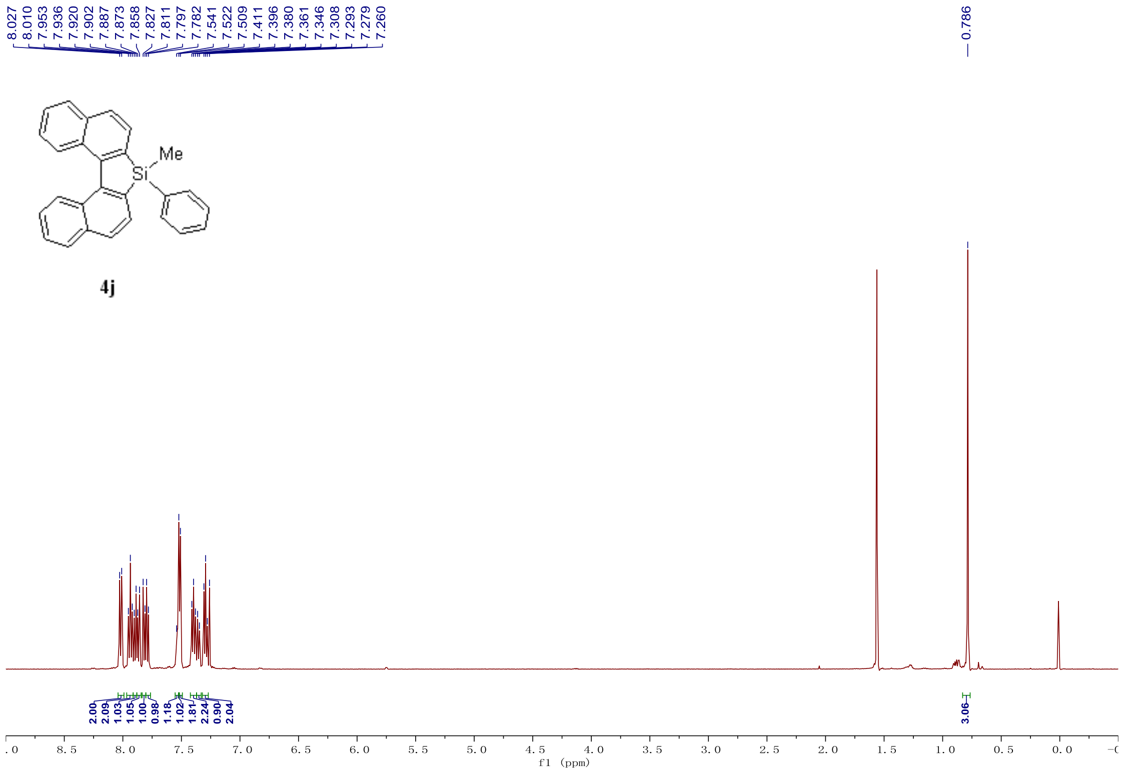

Supplementary Figure 52. ^{1}**H NMR** spectroscopy of **4j**

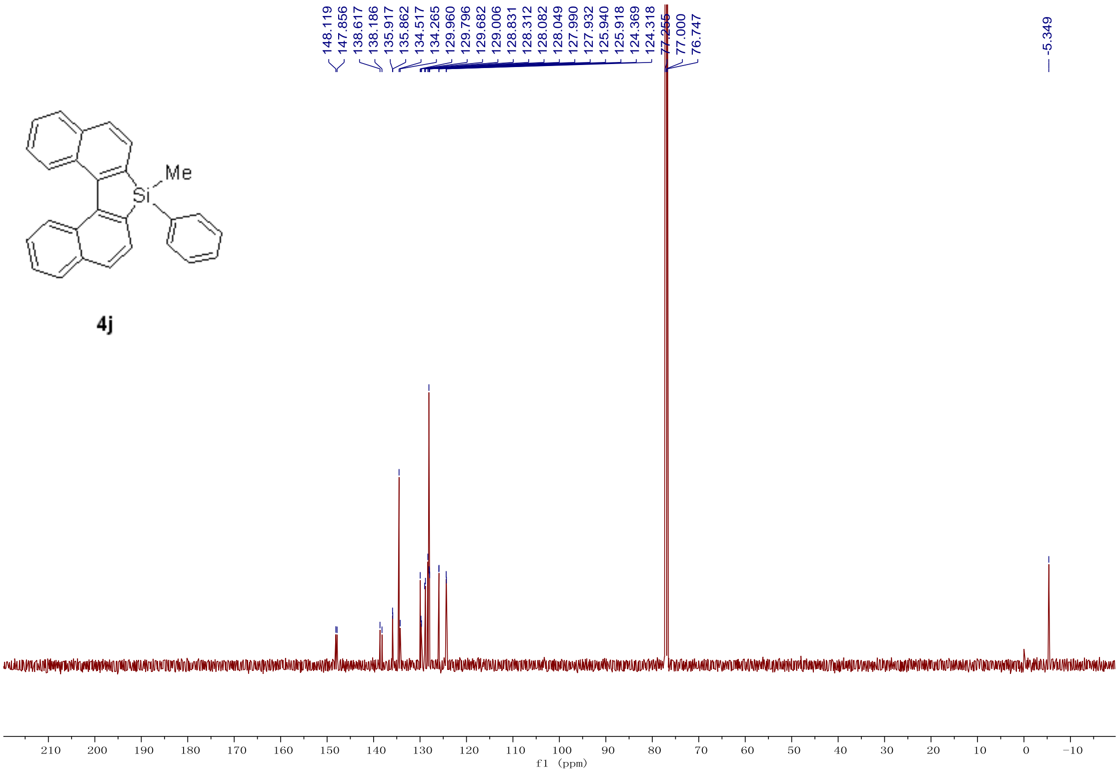

Supplementary Figure 53. ^{13}**C NMR** spectroscopy of **4j**

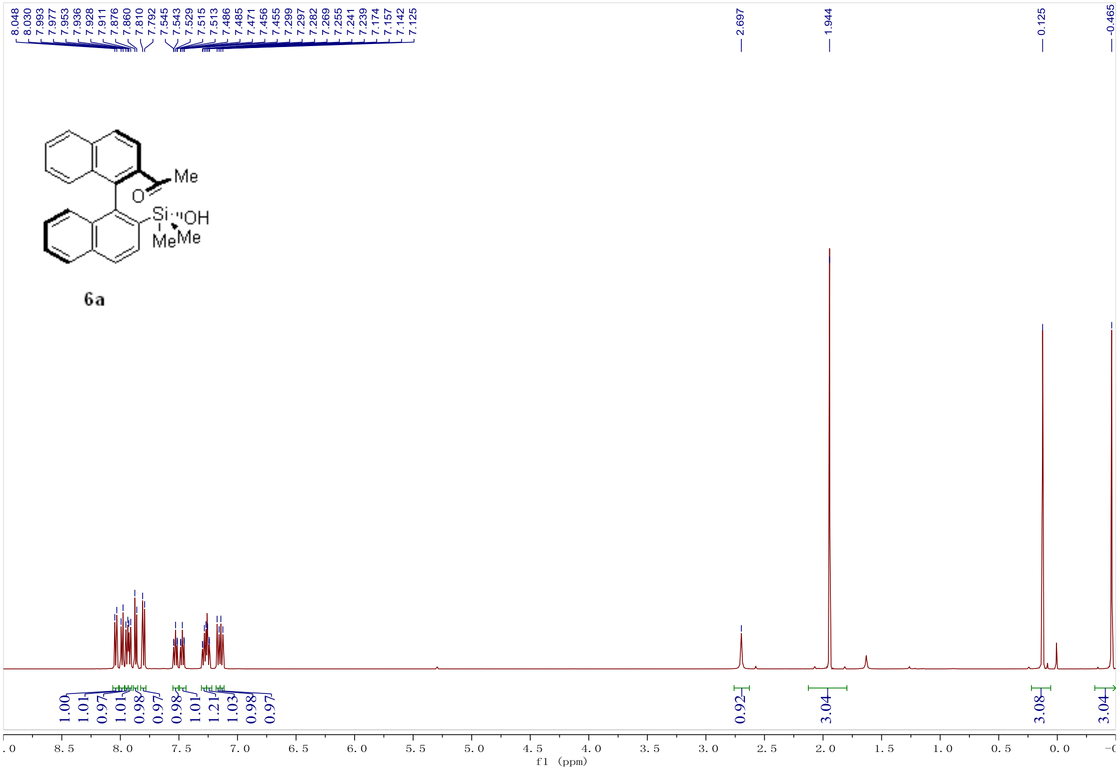

Supplementary Figure 54. ^{1}**H NMR** spectroscopy of **6a**

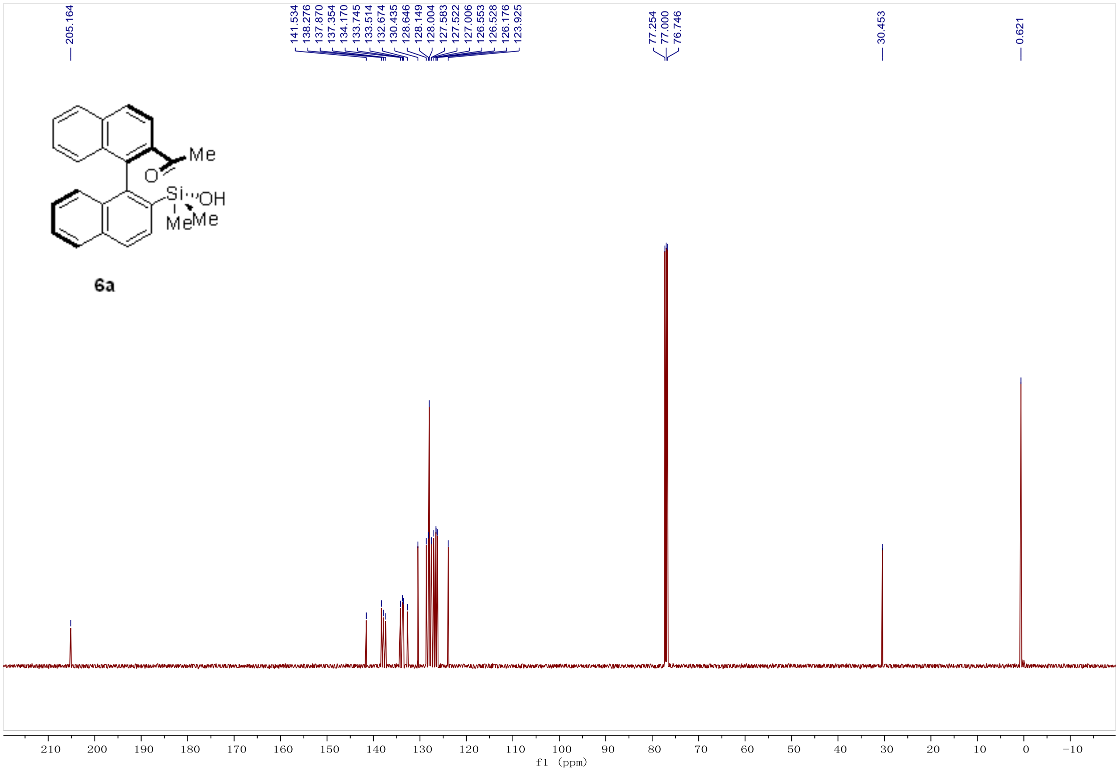

Supplementary Figure 55. ^{13}**C NMR** spectroscopy of **6a**

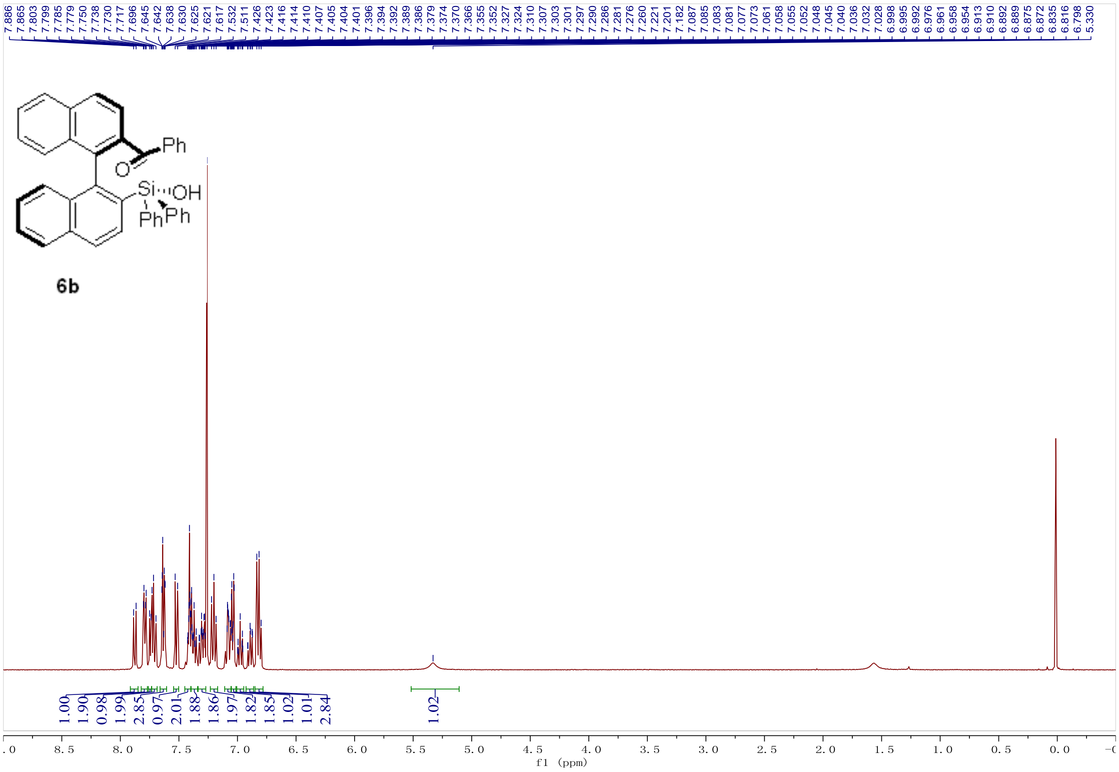

Supplementary Figure 56. ^{1}**H NMR** spectroscopy of **6b**

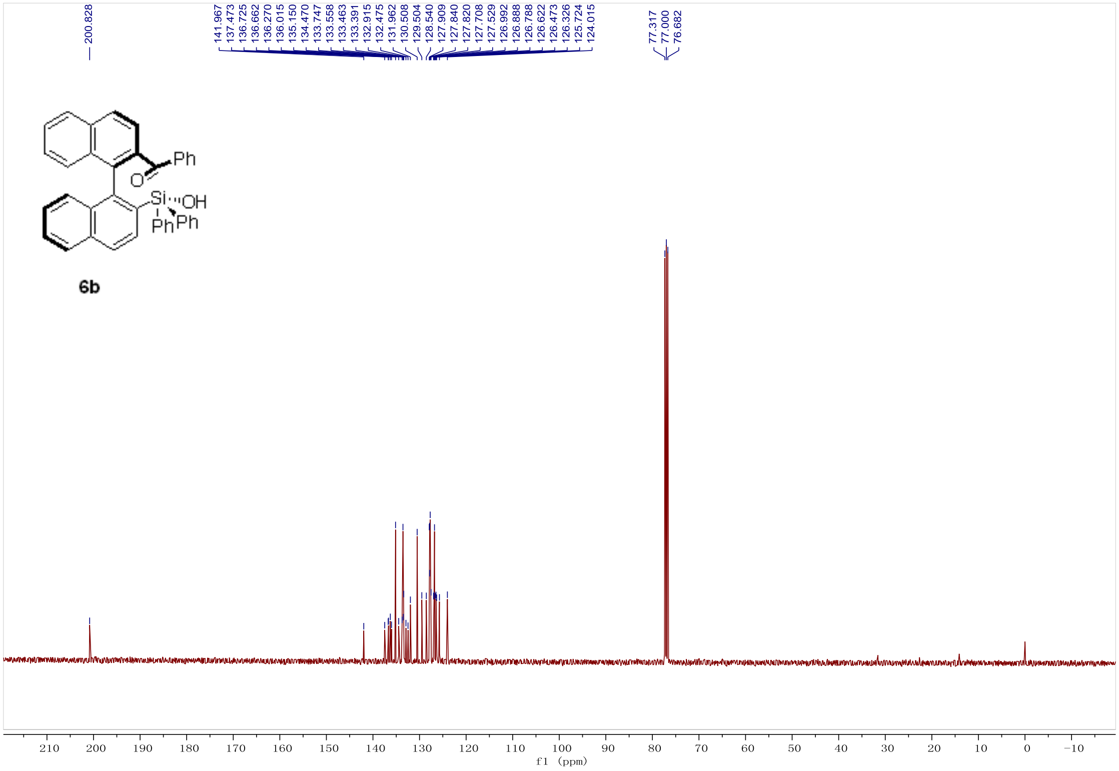

Supplementary Figure 57. ^{13}**C NMR** spectroscopy of **6b**

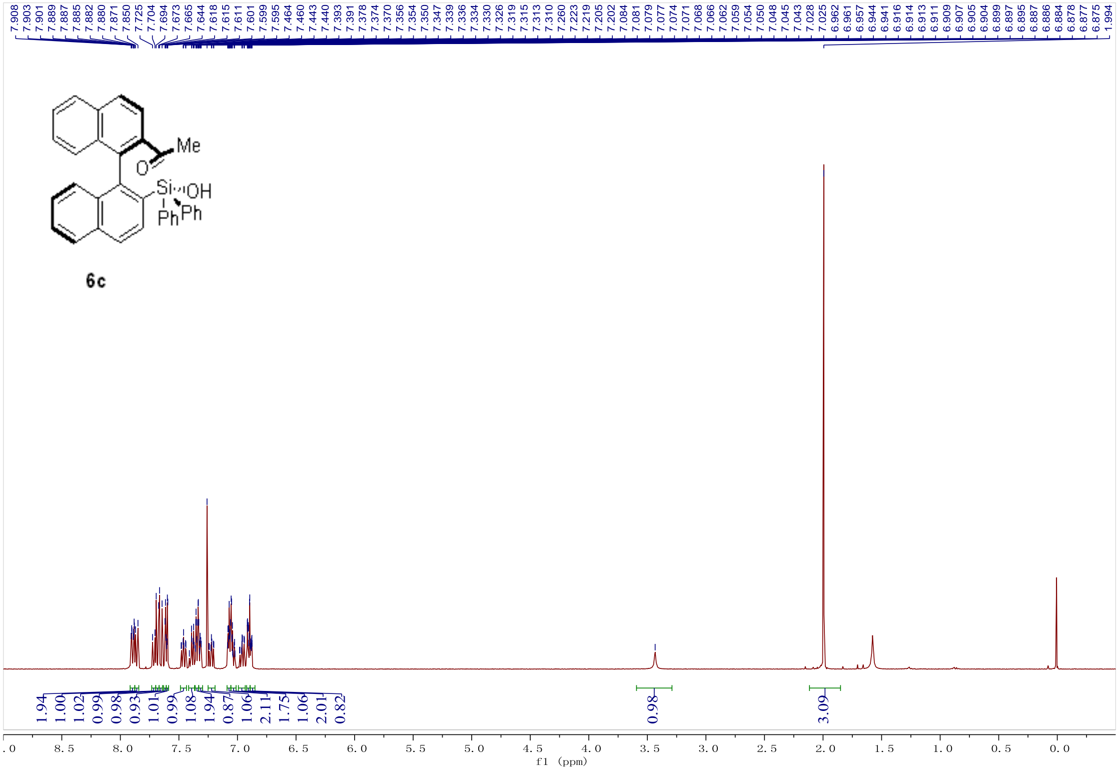

Supplementary Figure 58. ^{1}**H NMR** spectroscopy of **6c**

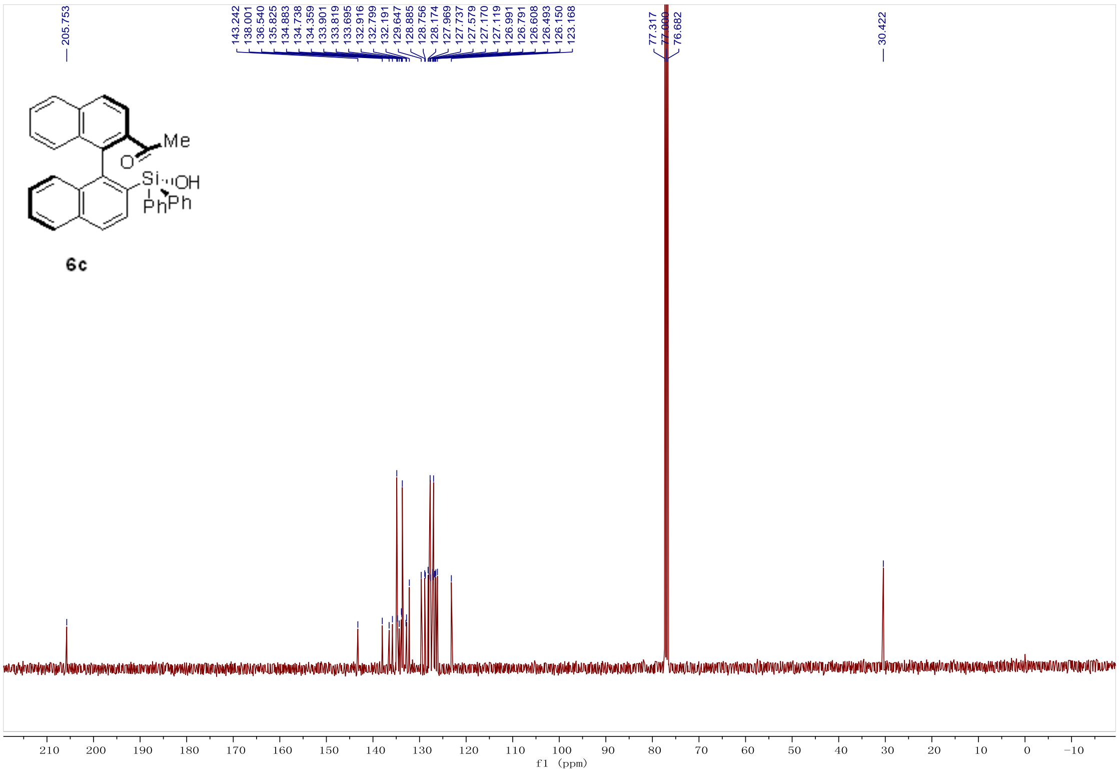

Supplementary Figure 59. ^{13}**C NMR** spectroscopy of **6c**

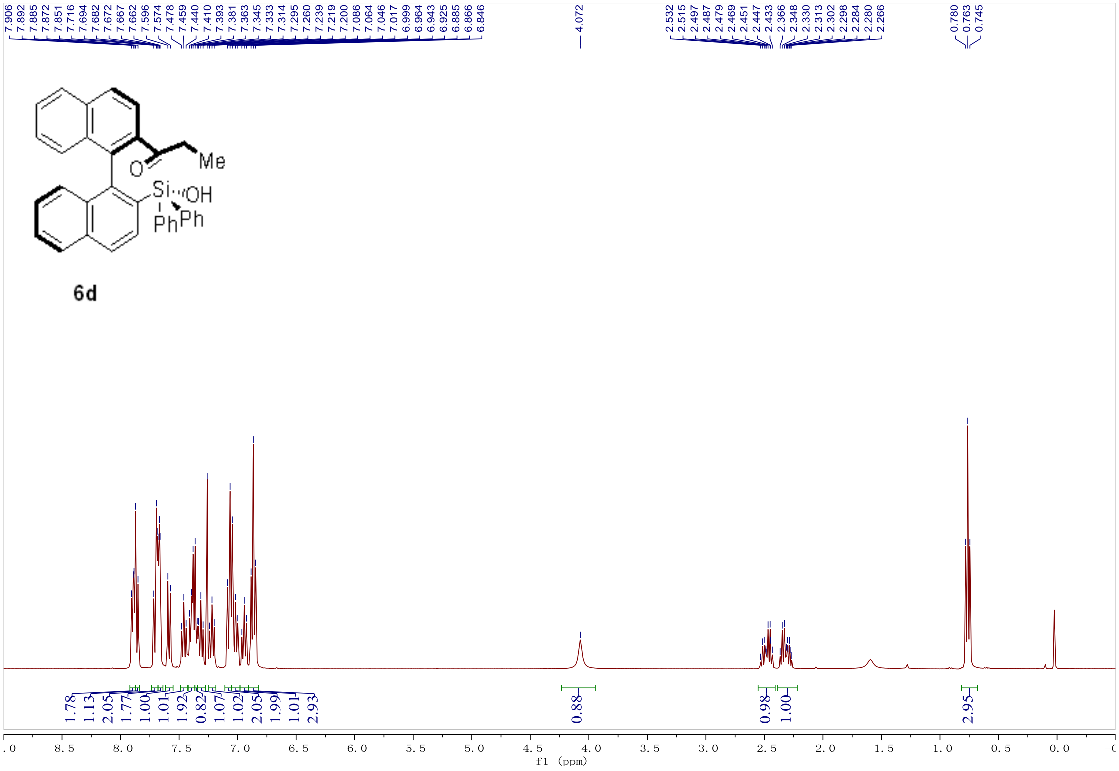

Supplementary Figure 60. ^{1}**H NMR** spectroscopy of **6d**

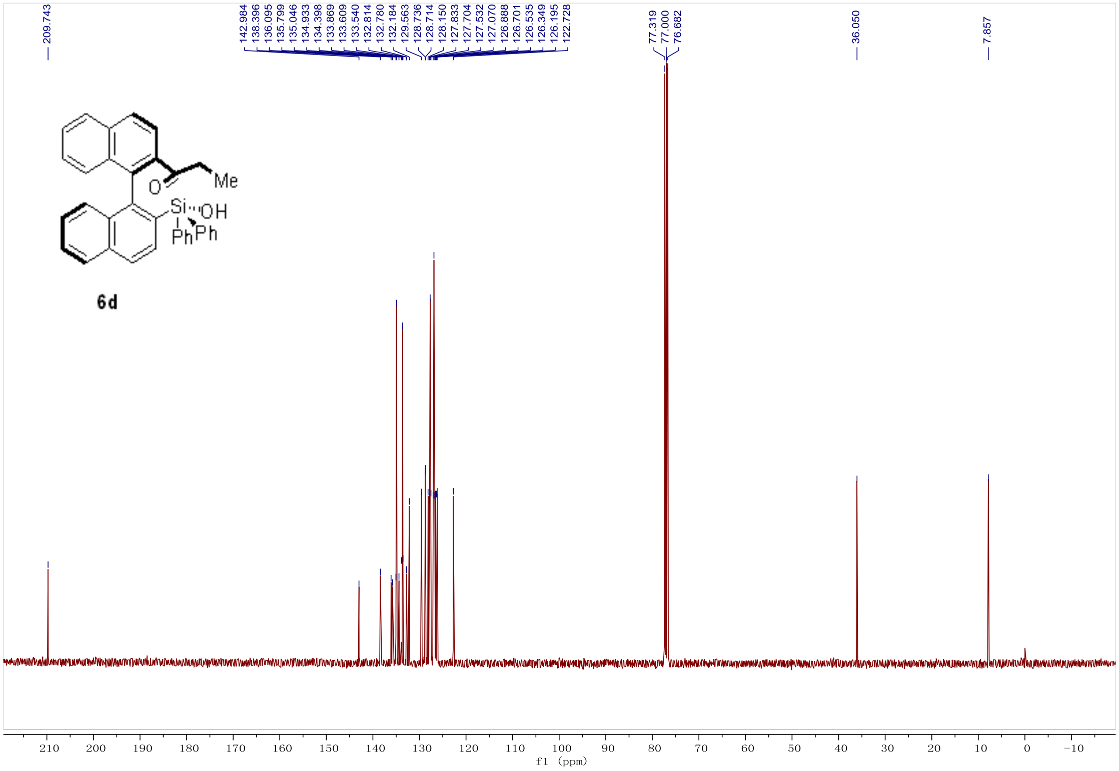

Supplementary Figure 61. ^{13}**C NMR** spectroscopy of **6d**

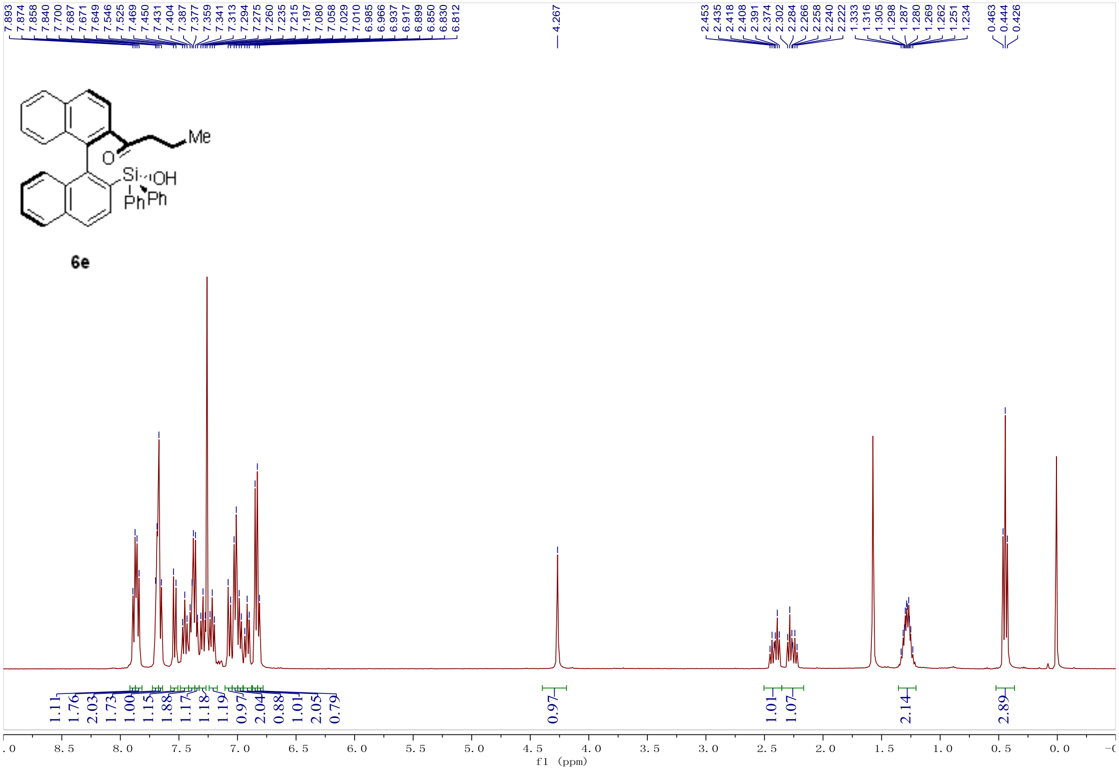

Supplementary Figure 62. ^{1}**H NMR** spectroscopy of **6e**

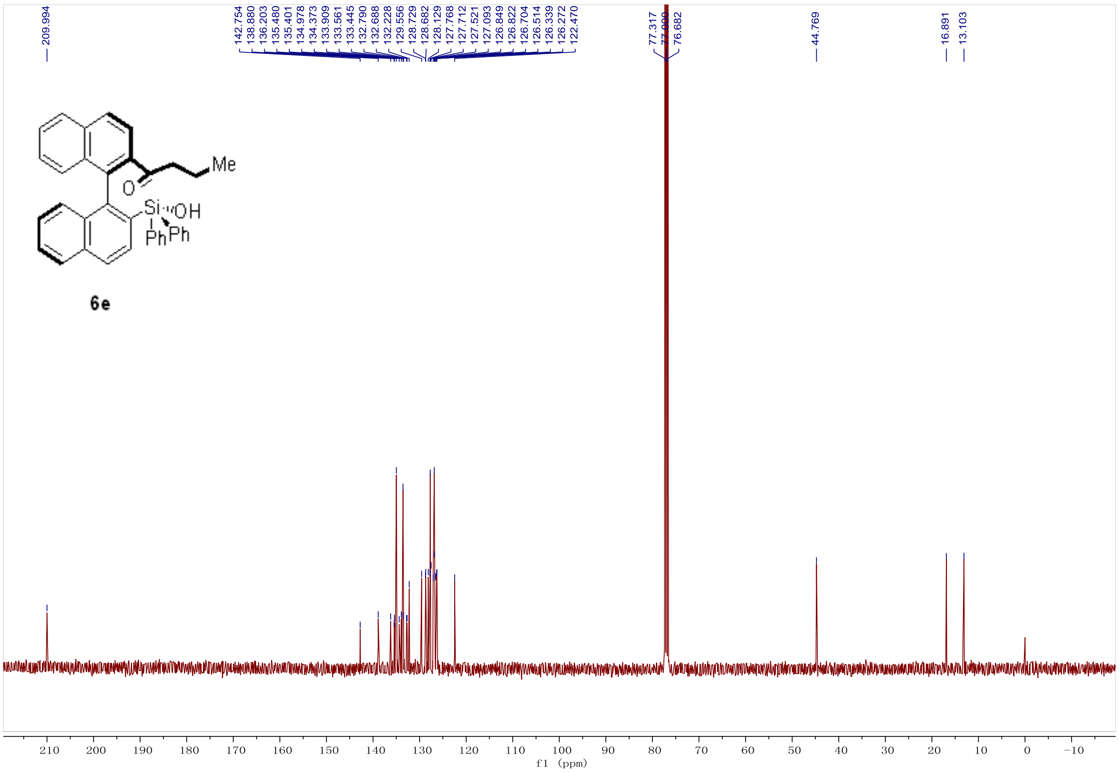

Supplementary Figure 63. ^{13}**C NMR** spectroscopy of **6e**

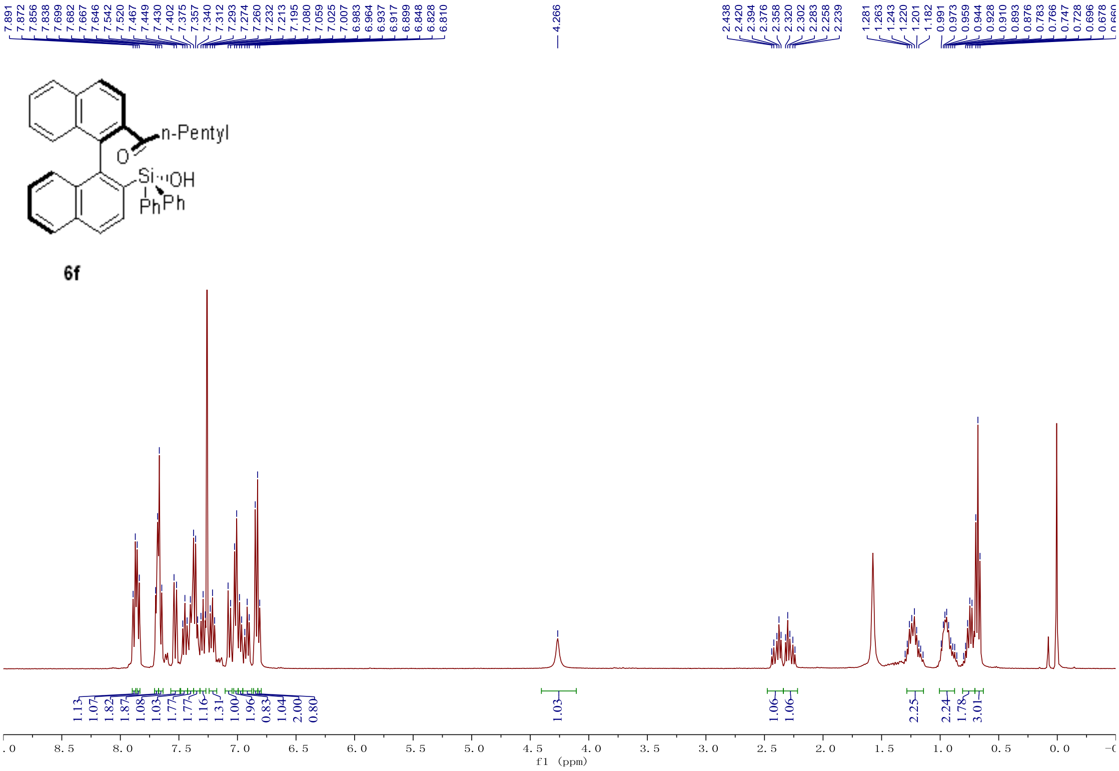

Supplementary Figure 64. ^{1}**H NMR** spectroscopy of **6f**

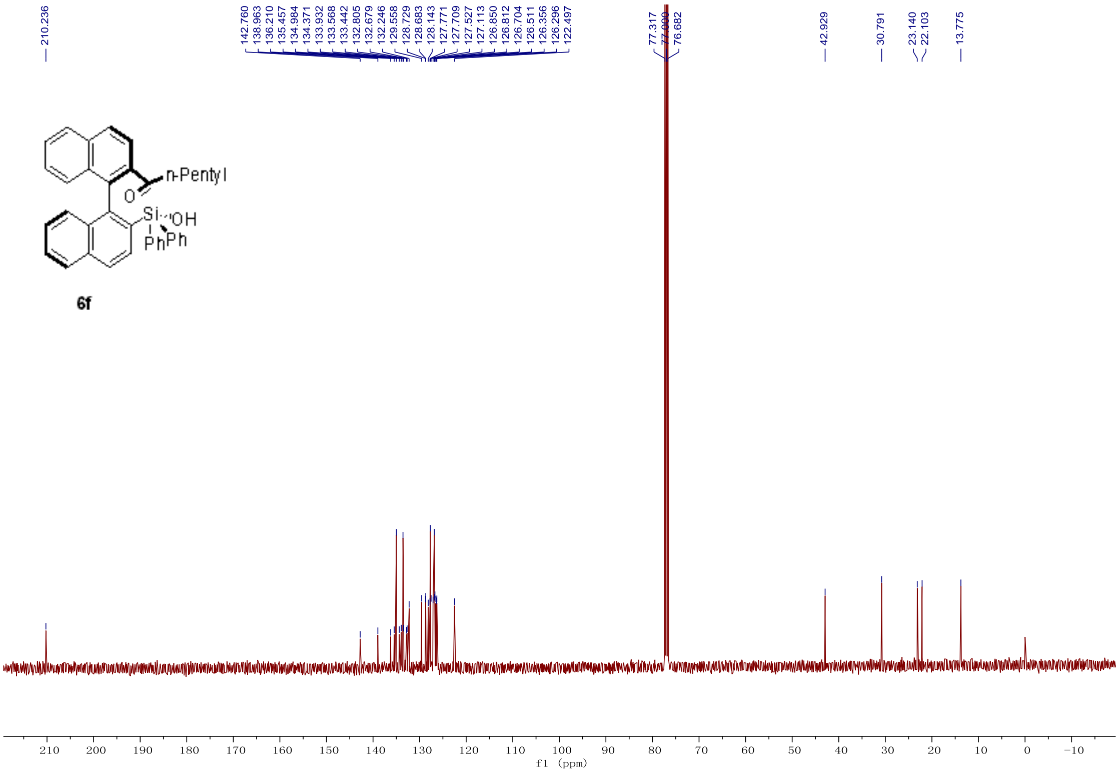

Supplementary Figure 65. ^{13}**C NMR** spectroscopy of **6f**

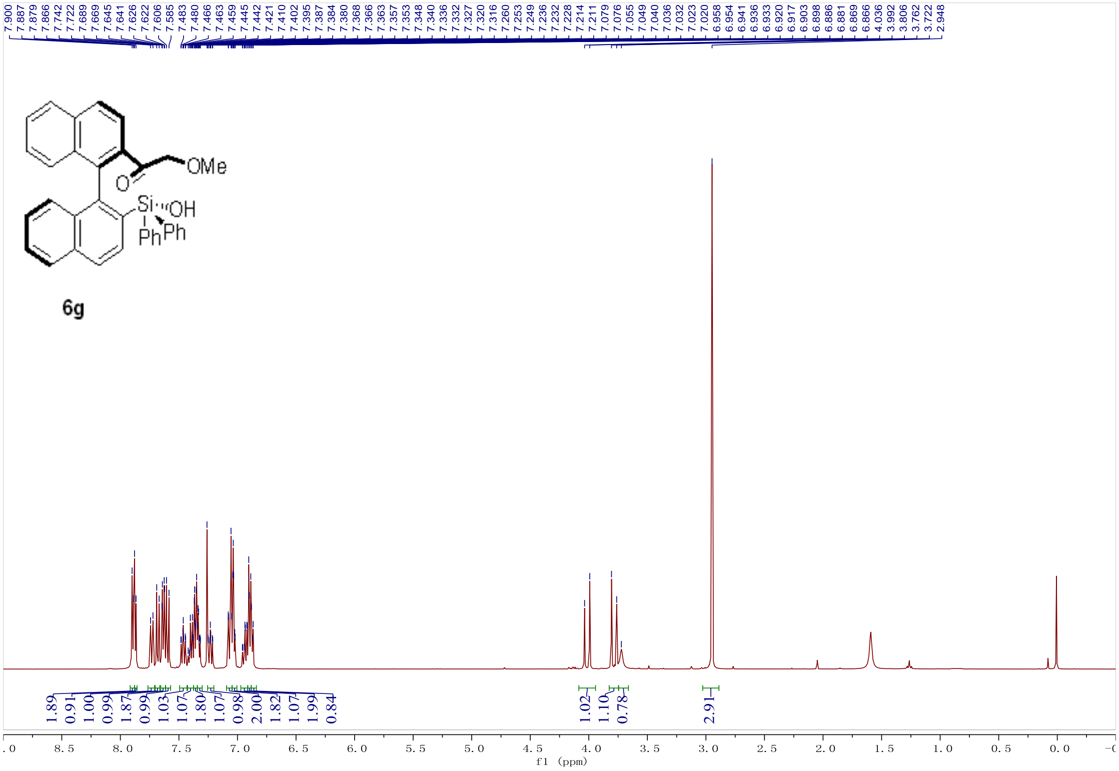

Supplementary Figure 66. ^{1}**H NMR** spectroscopy of **6g**

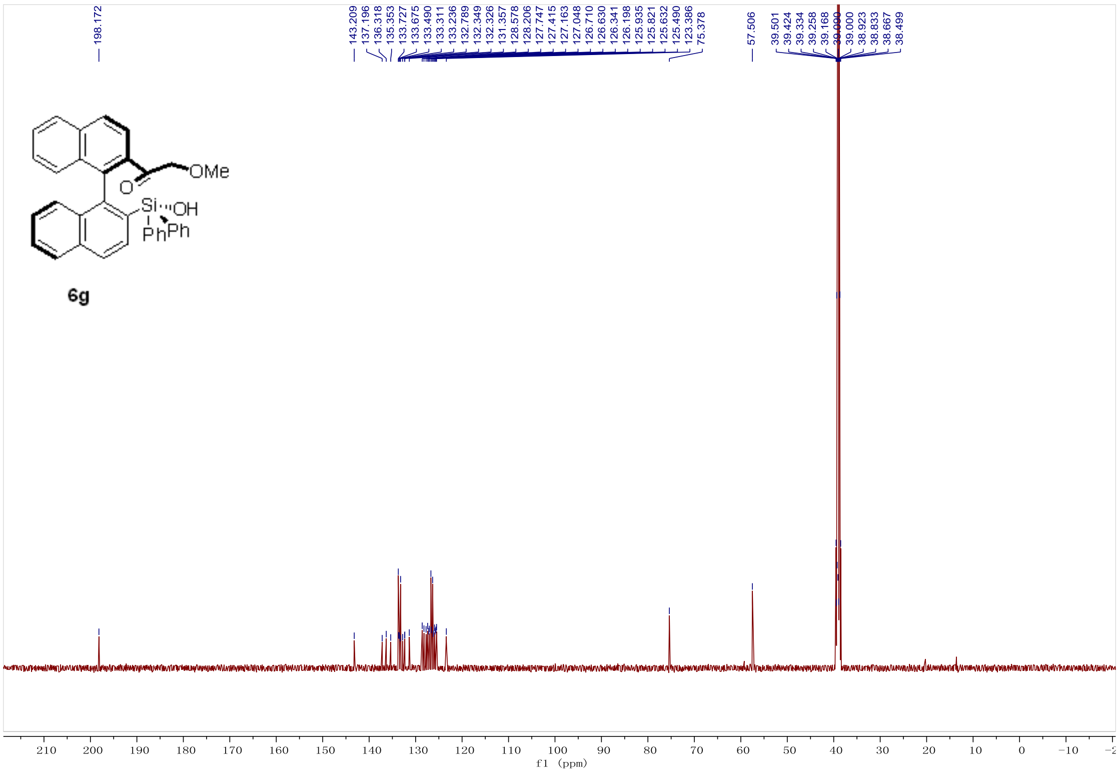

Supplementary Figure 67. ^{13}**C NMR** spectroscopy of **6g**

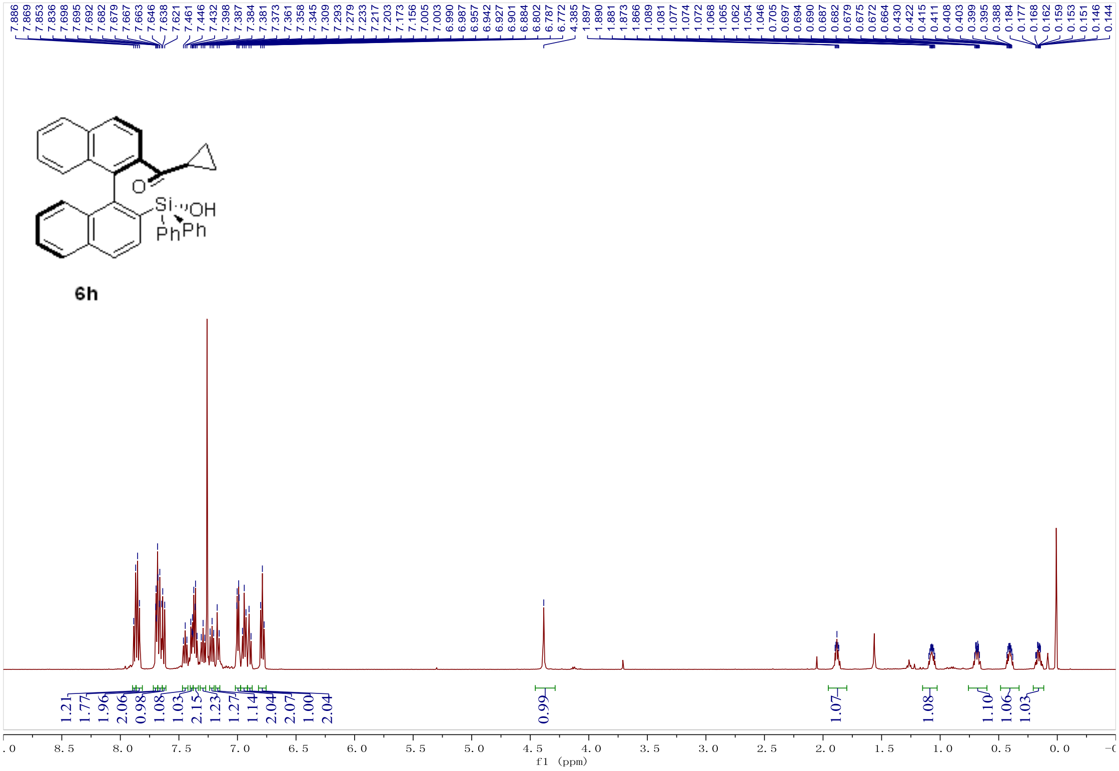

Supplementary Figure 68. ^{1}**H NMR** spectroscopy of **6h**

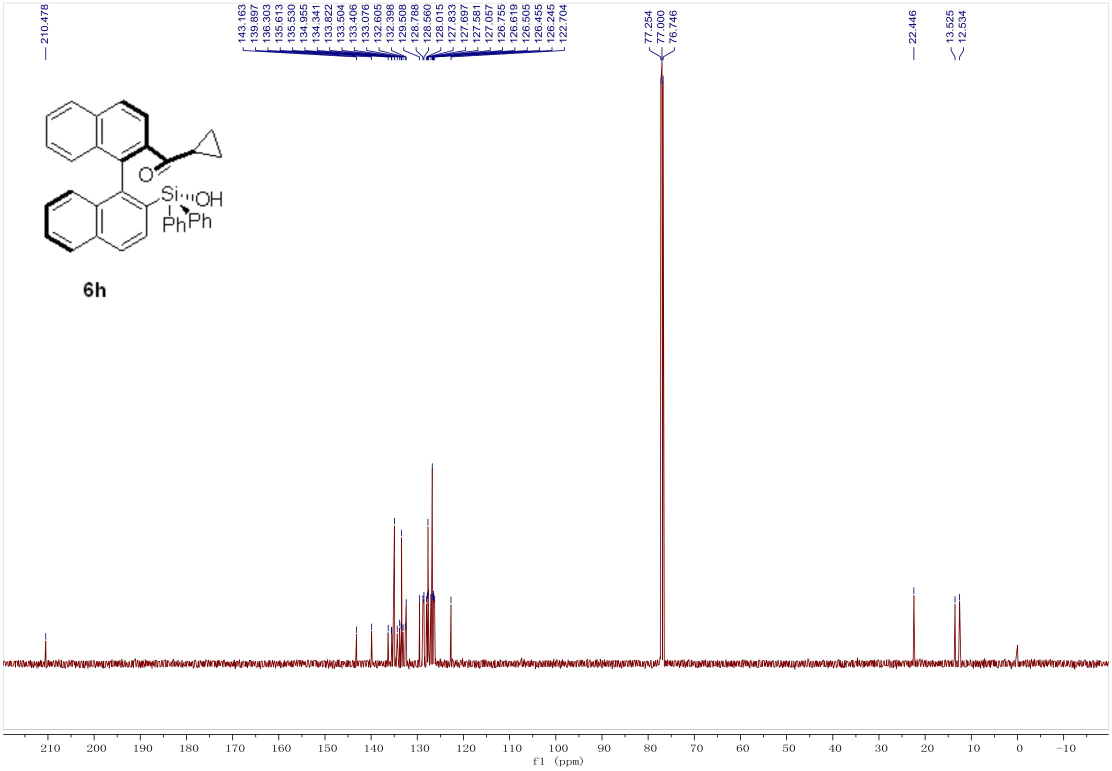

Supplementary Figure 69. ^{13}**C NMR** spectroscopy of **6h**

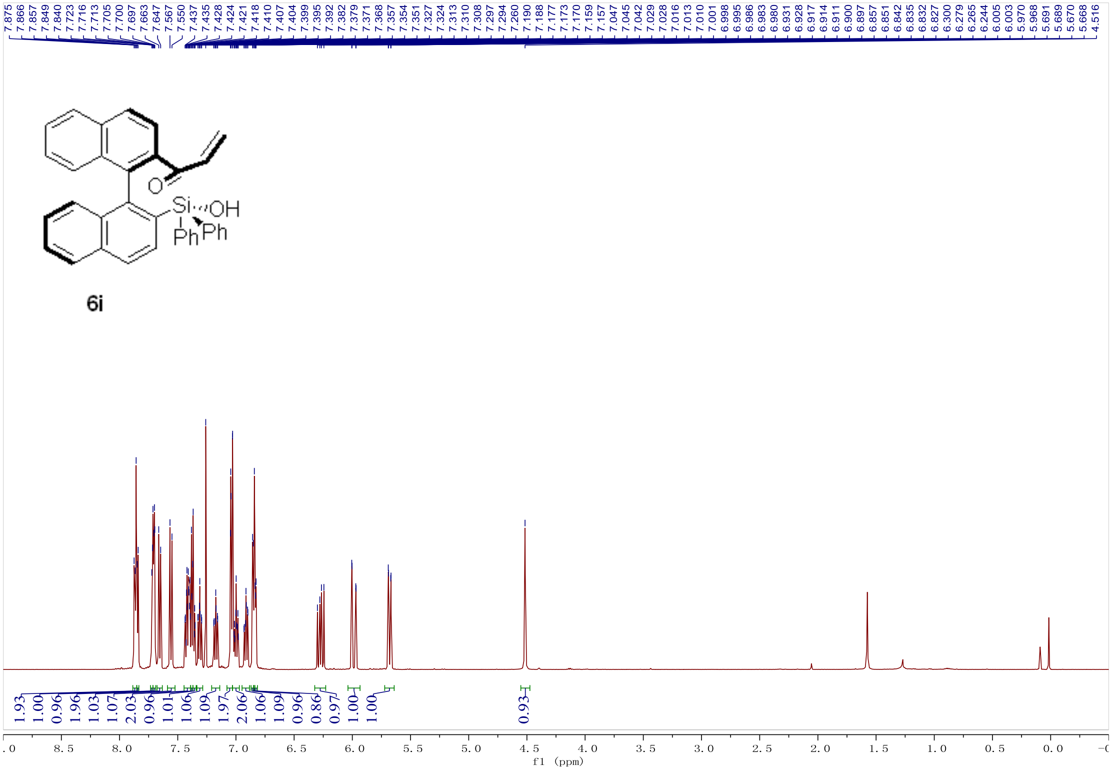

Supplementary Figure 70. ^{1}**H NMR** spectroscopy of **6i**

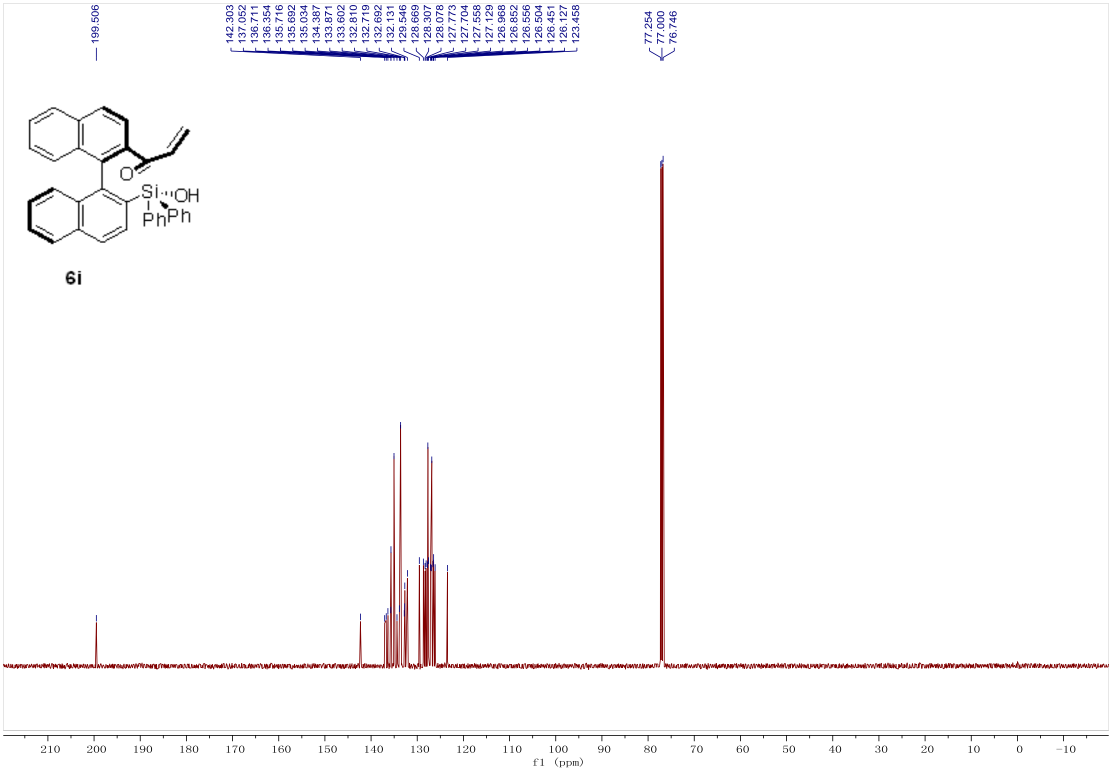

Supplementary Figure 71. ^{13}**C NMR** spectroscopy of **6i**

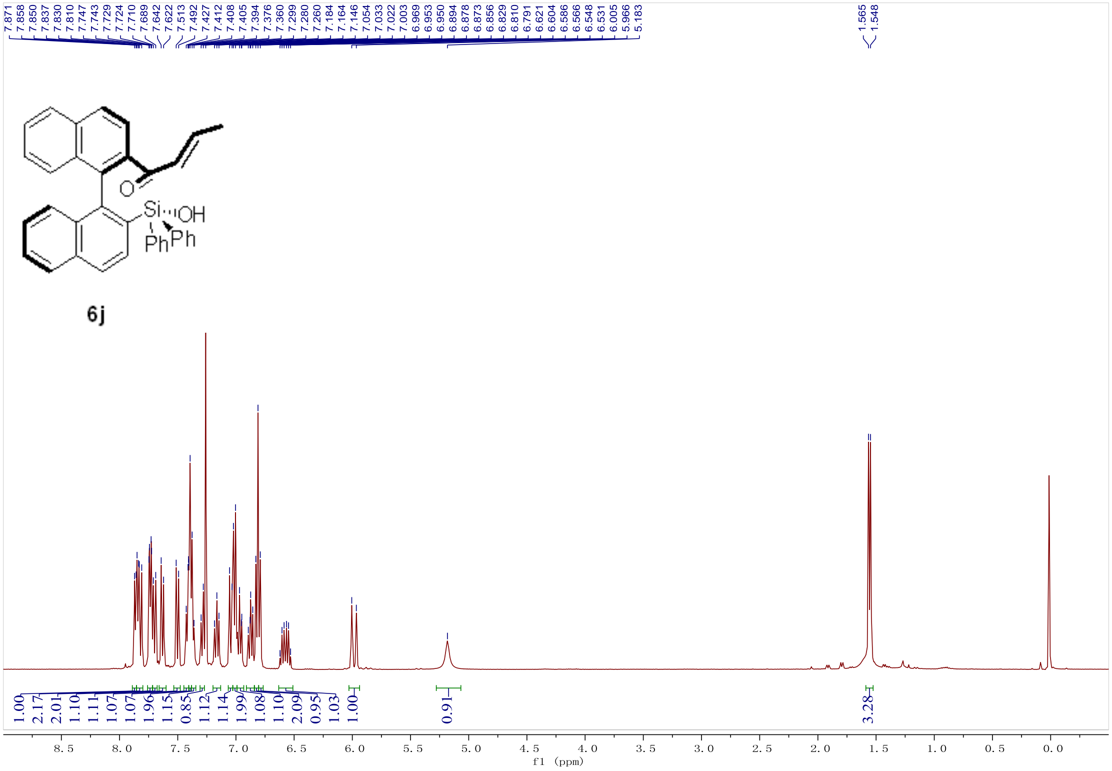

Supplementary Figure 72. ^{1}**H NMR** spectroscopy of **6j**

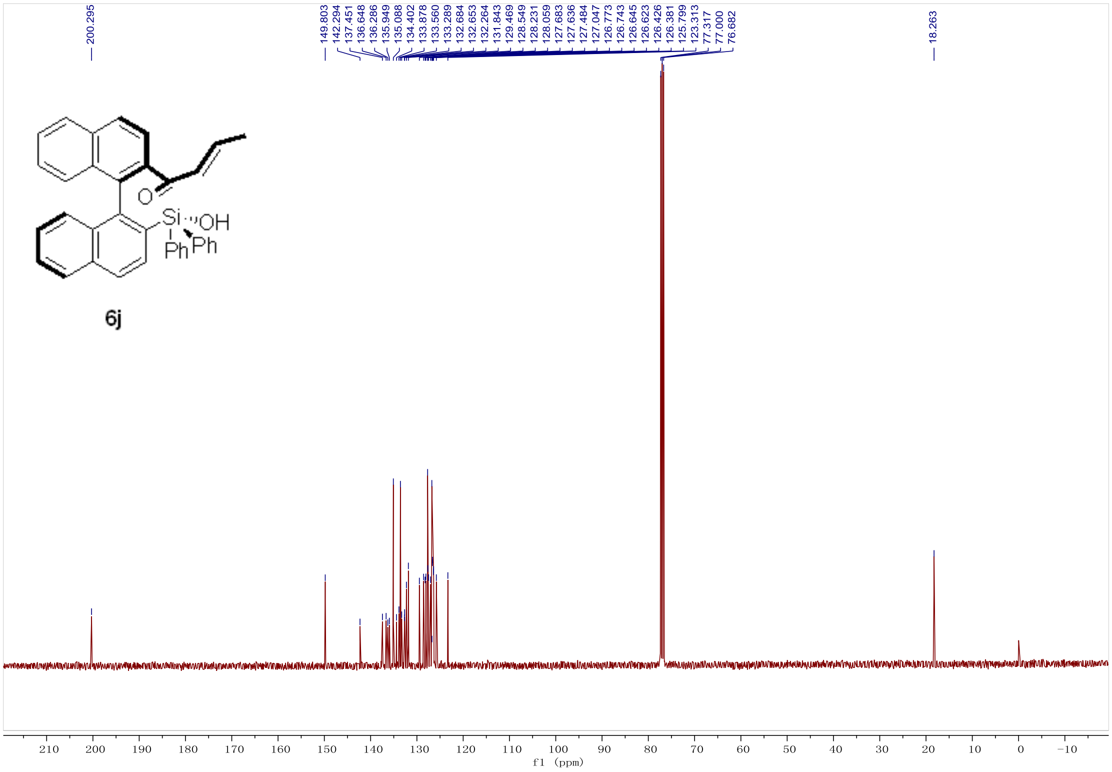

Supplementary Figure 73. ^{13}C NMR spectroscopy of **6j**

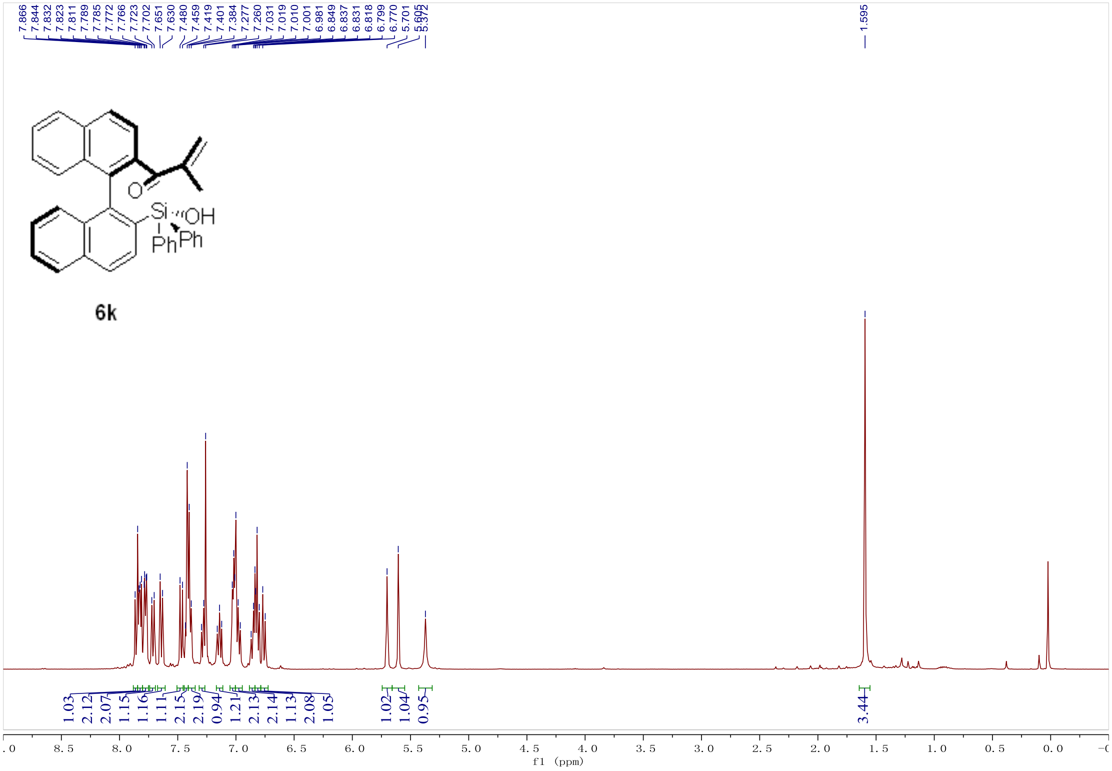

Supplementary Figure 74. ^{1}H NMR spectroscopy of **6k**

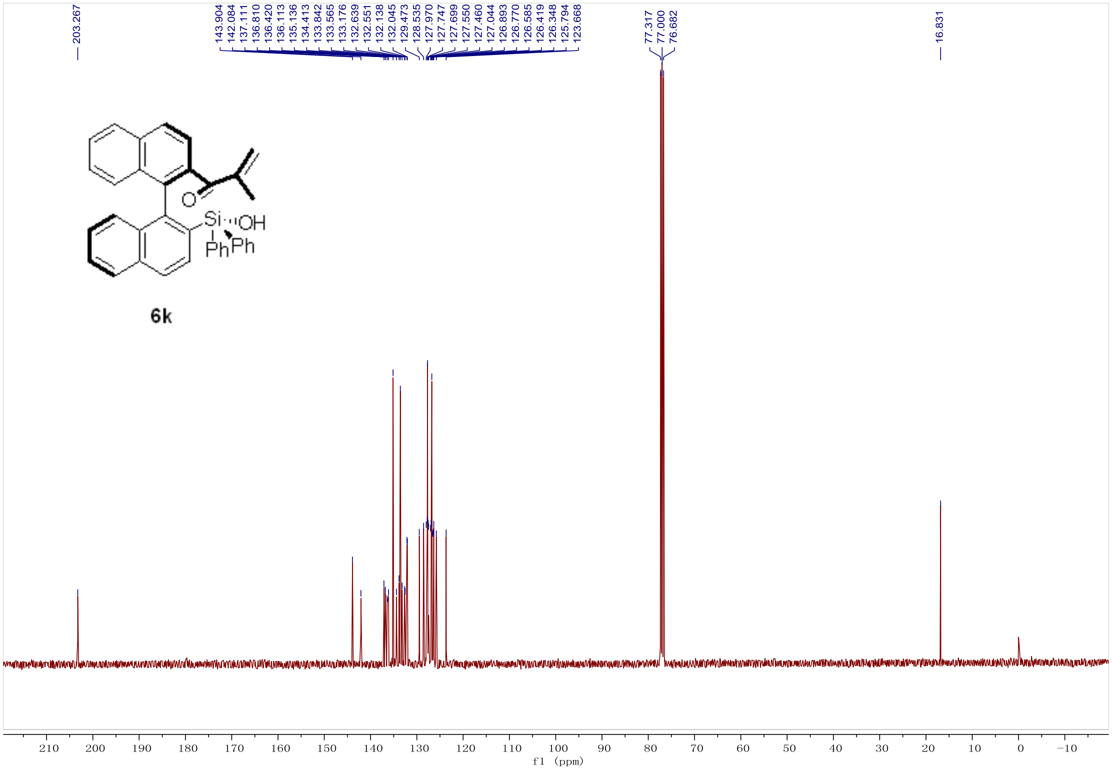

Supplementary Figure 75. ^{13}**C NMR** spectroscopy of **6k**

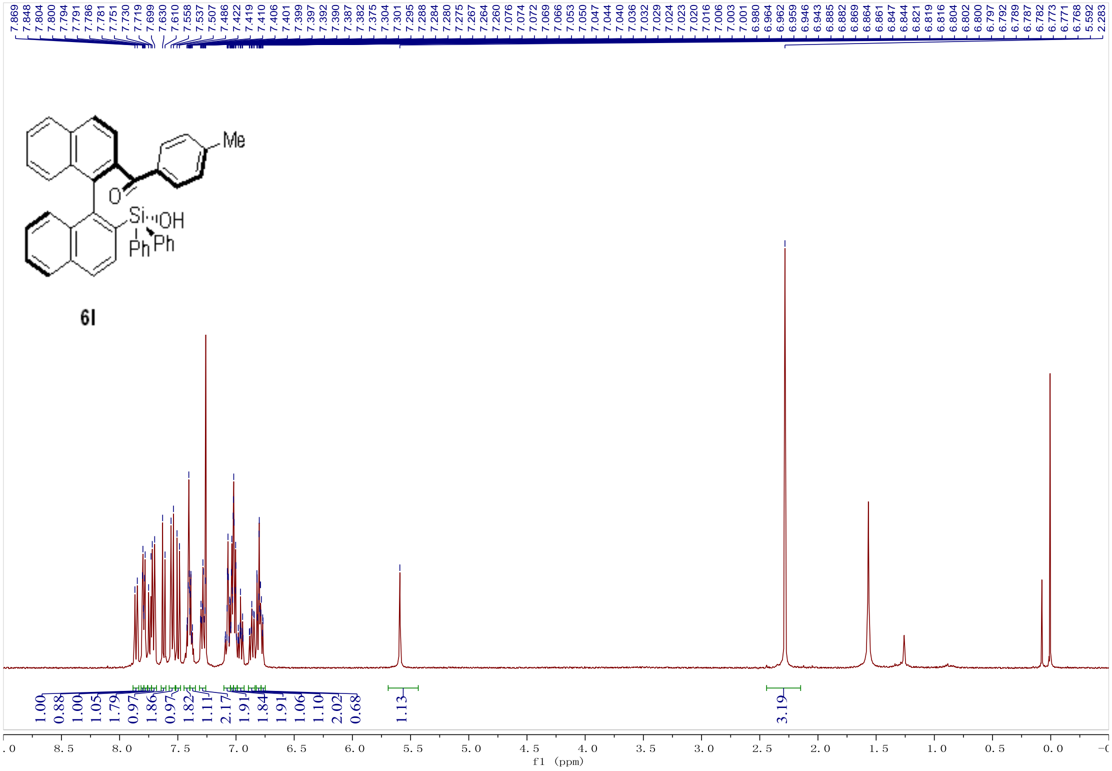

Supplementary Figure 76. ^{1}**H NMR** spectroscopy of **6l**

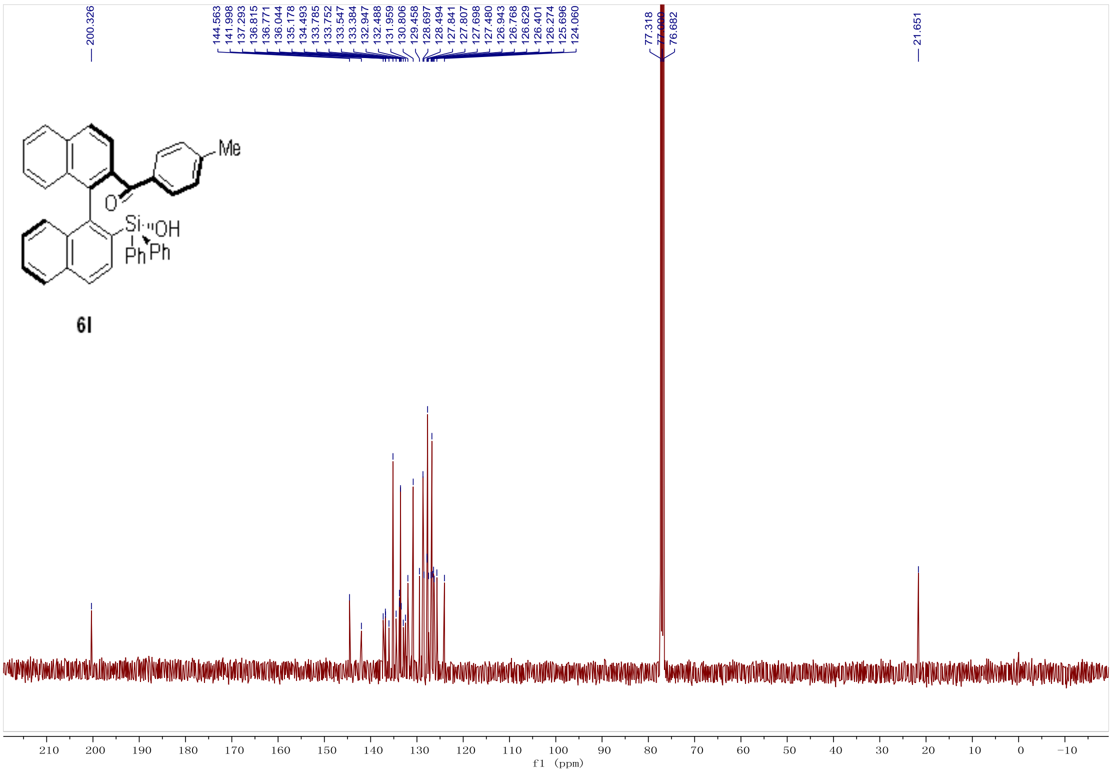

Supplementary Figure 77. ^{13}**C NMR** spectroscopy of **6l**

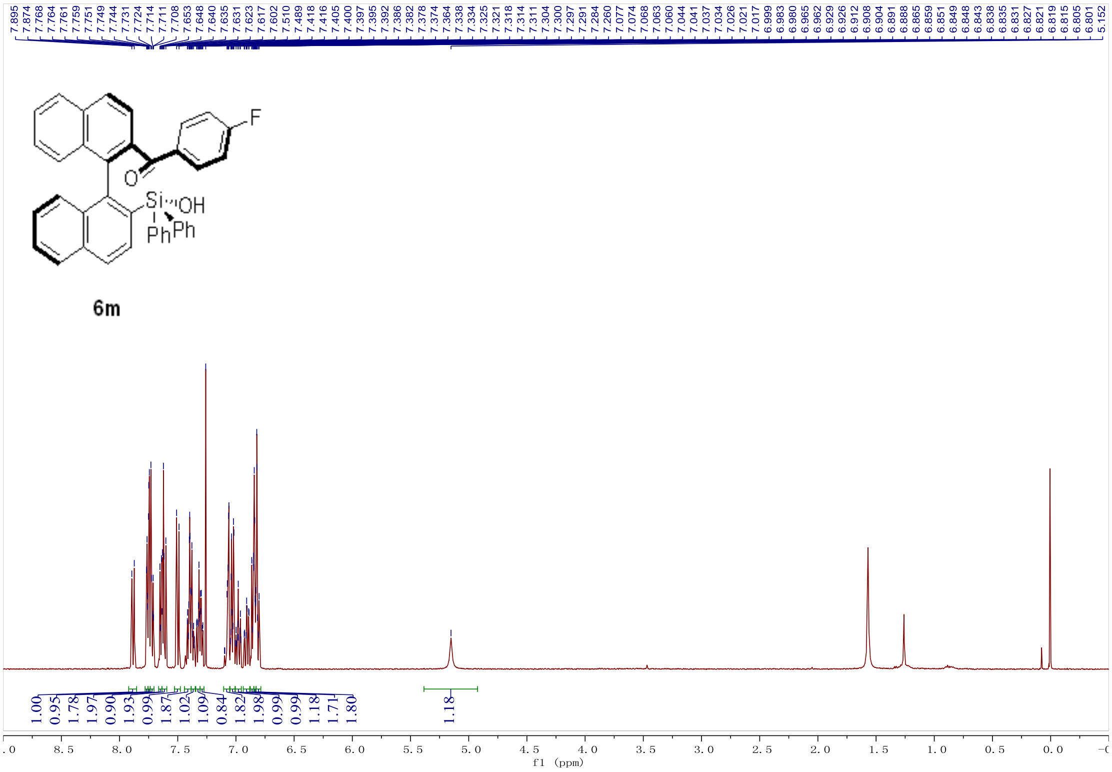

Supplementary Figure 78. ^{1}**H NMR** spectroscopy of **6m**

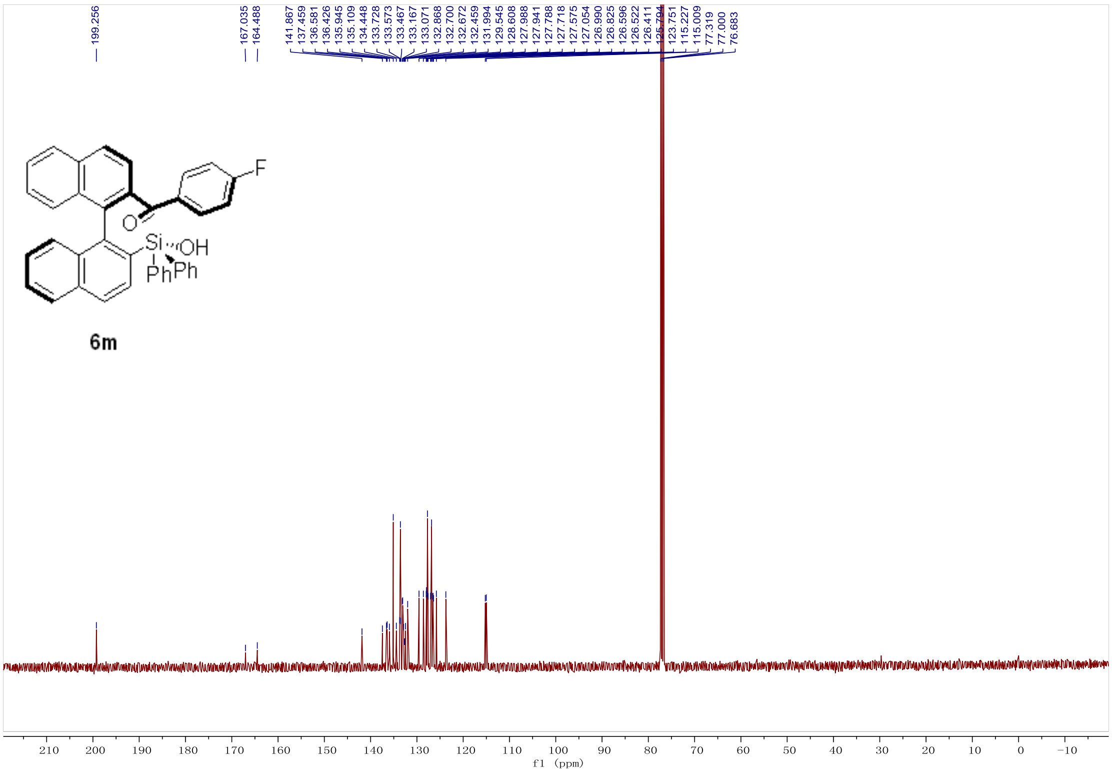

Supplementary Figure 79. ^{13}**C NMR** spectroscopy of **6m**

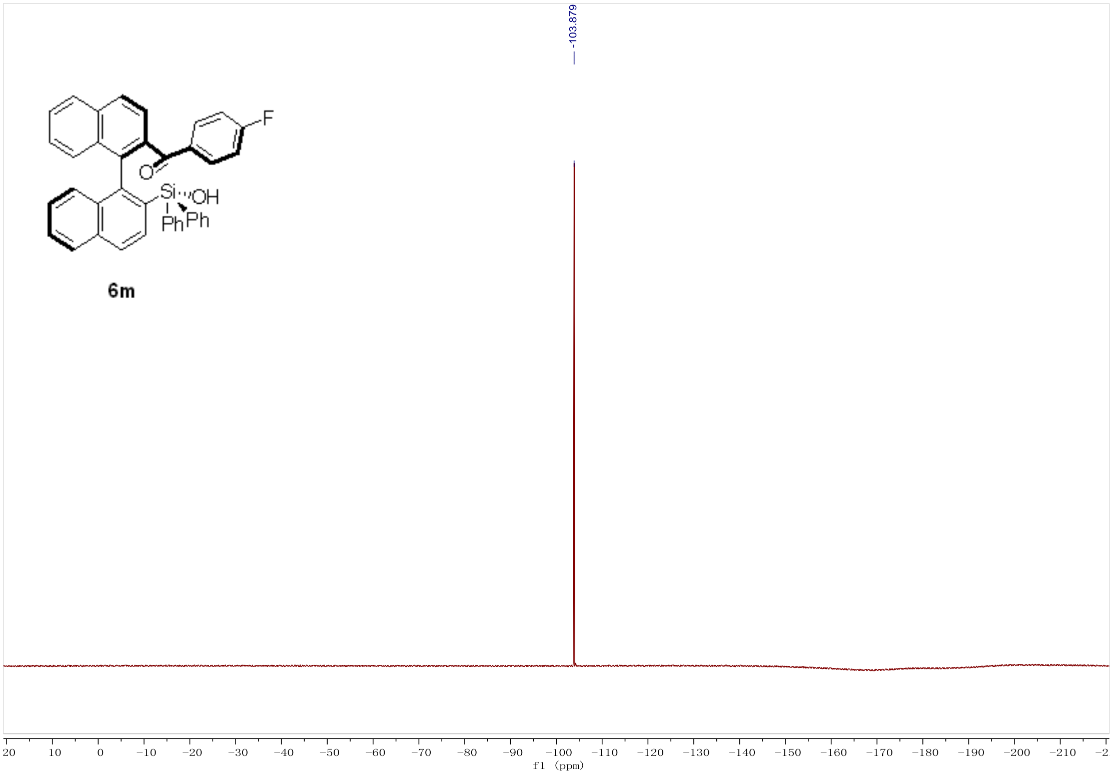

Supplementary Figure 80. ^{19}**F NMR** spectroscopy of **6m**

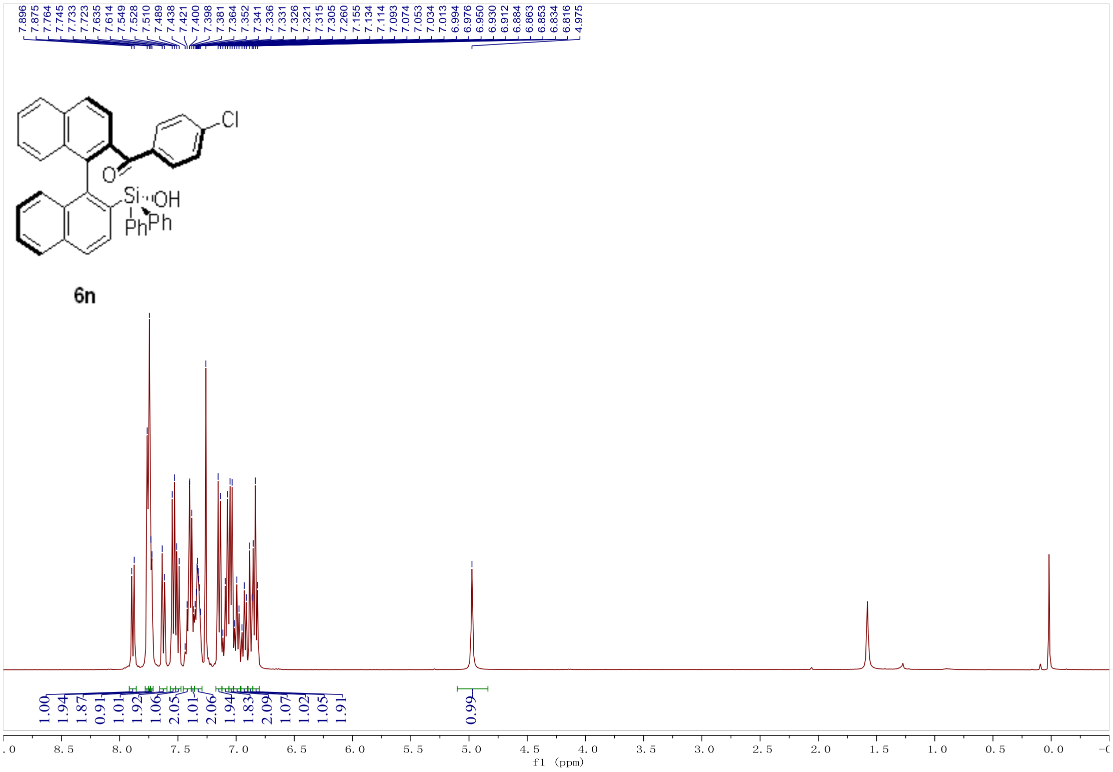

Supplementary Figure 81. ^{1}**H NMR** spectroscopy of **6n**

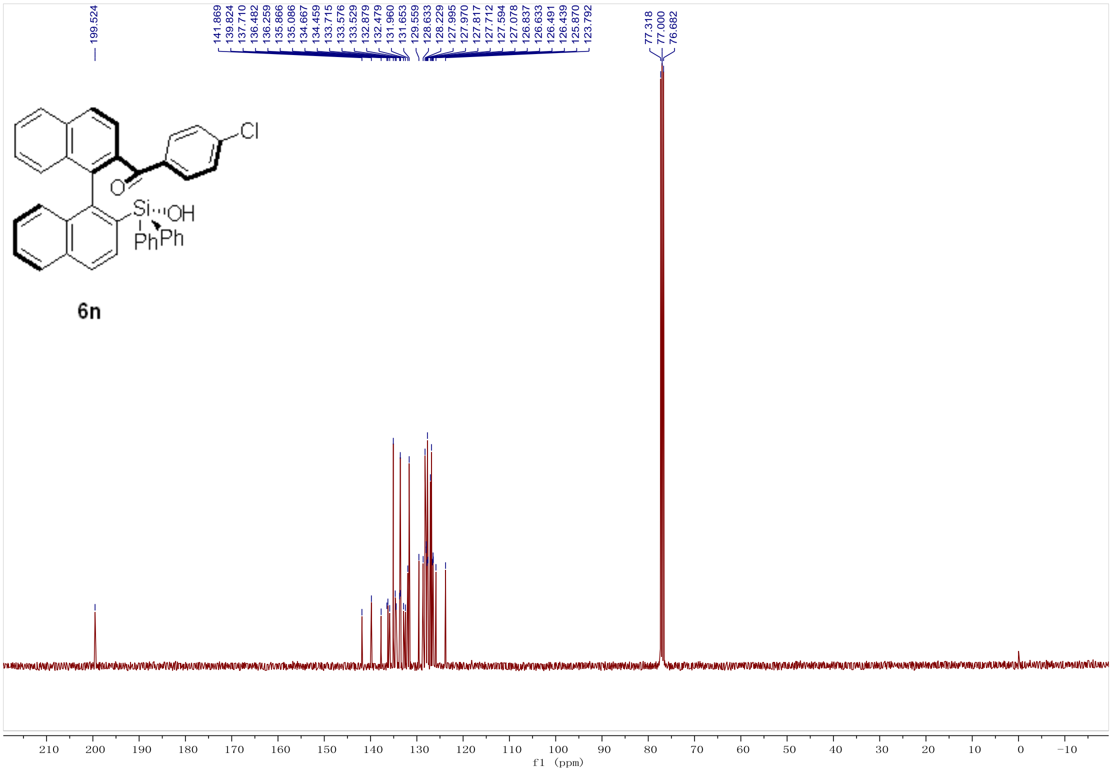

Supplementary Figure 82. ^{13}**C NMR** spectroscopy of **6n**

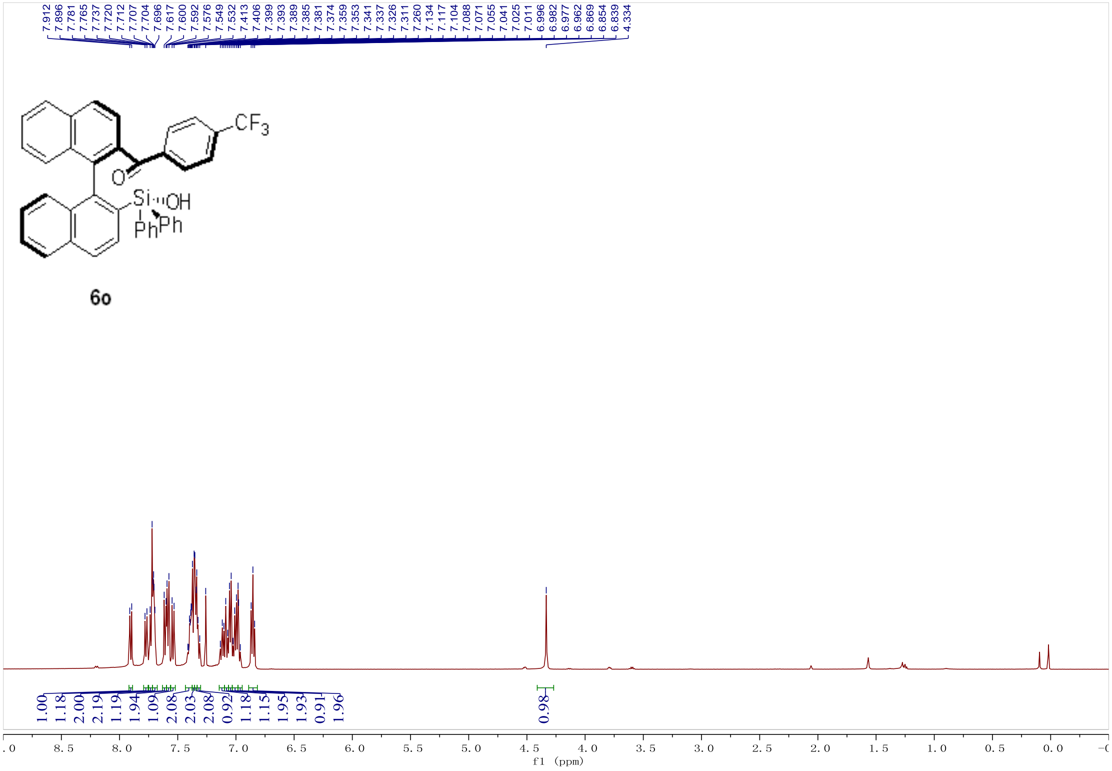

Supplementary Figure 83. **^{1}H NMR** spectroscopy of **6o**

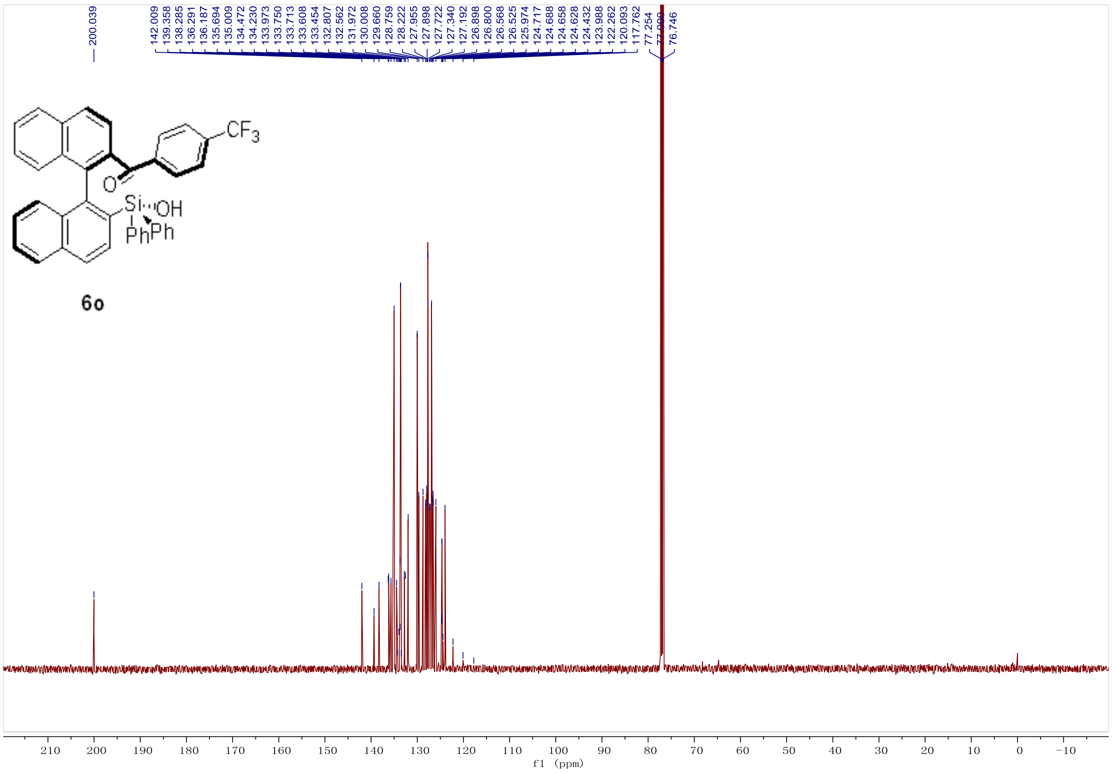

Supplementary Figure 84. **^{13}C NMR** spectroscopy of **6o**

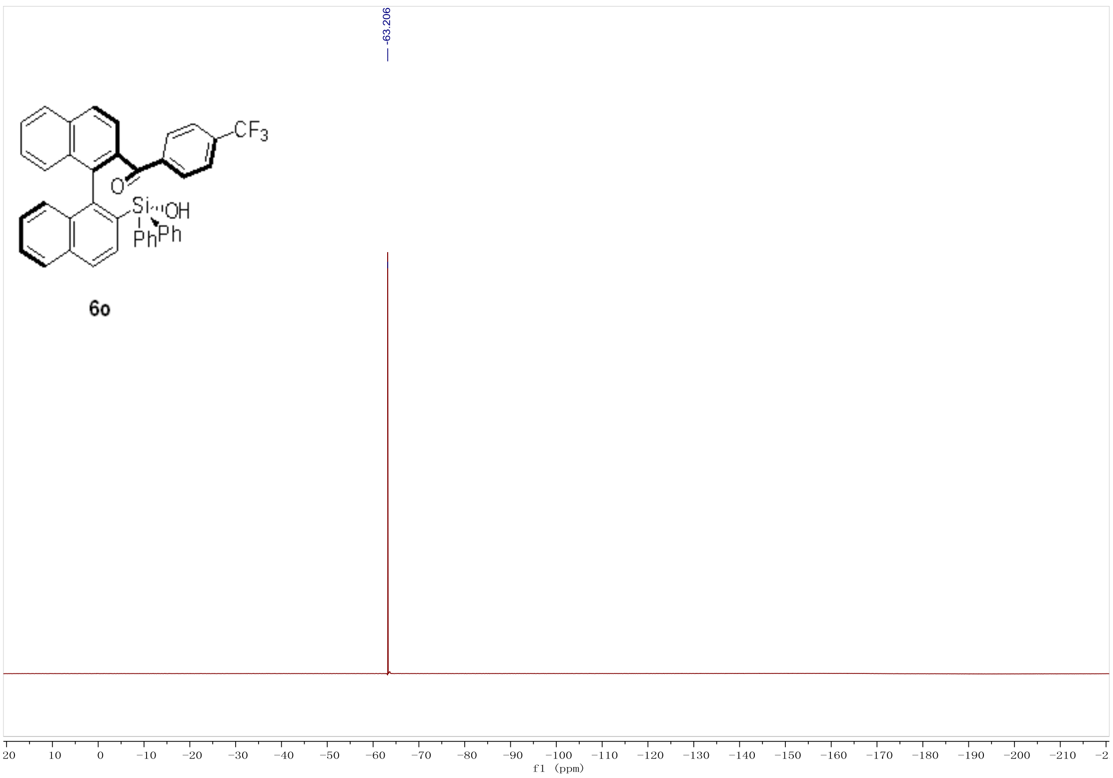

Supplementary Figure 85. ^{19}**F NMR** spectroscopy of **6o**

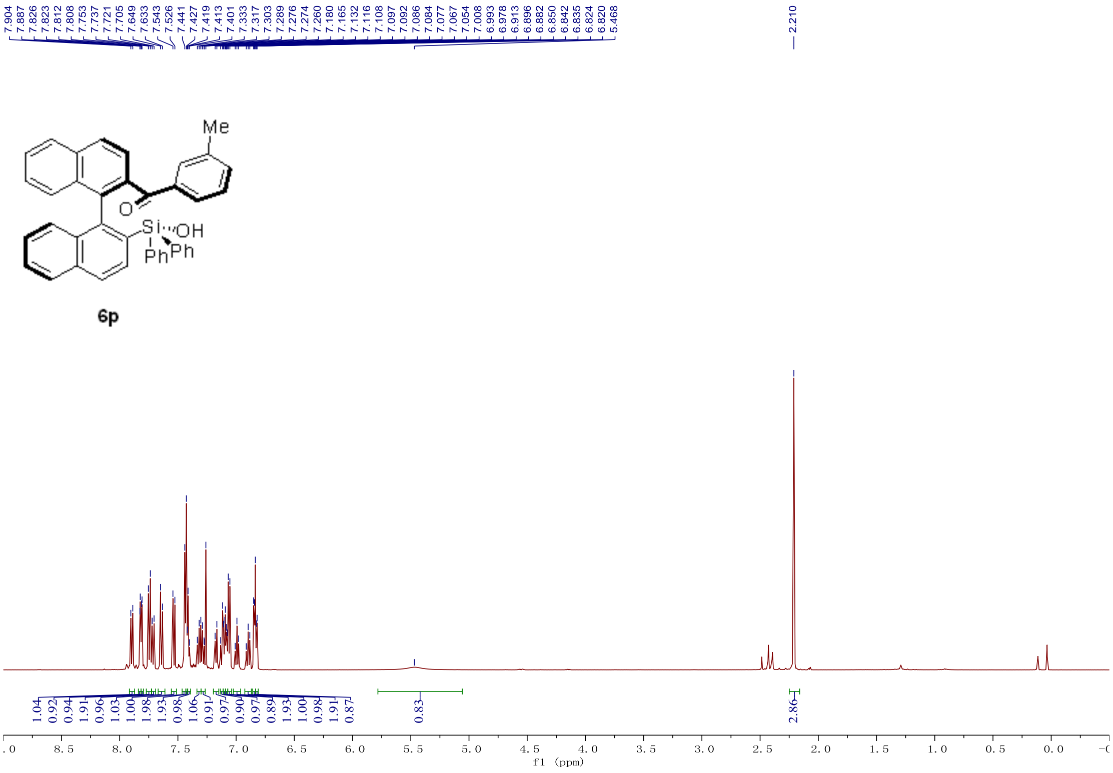

Supplementary Figure 86. ^{1}**H NMR** spectroscopy of **6p**

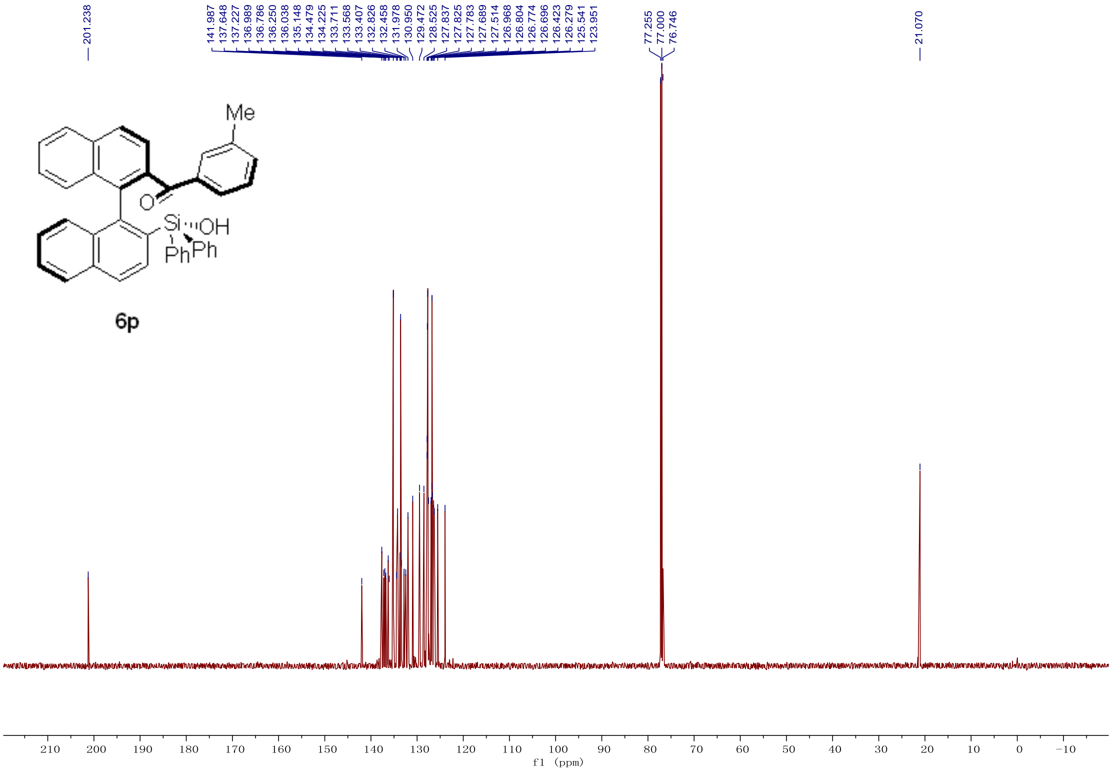

Supplementary Figure 87. ^{13}**C NMR** spectroscopy of **6p**

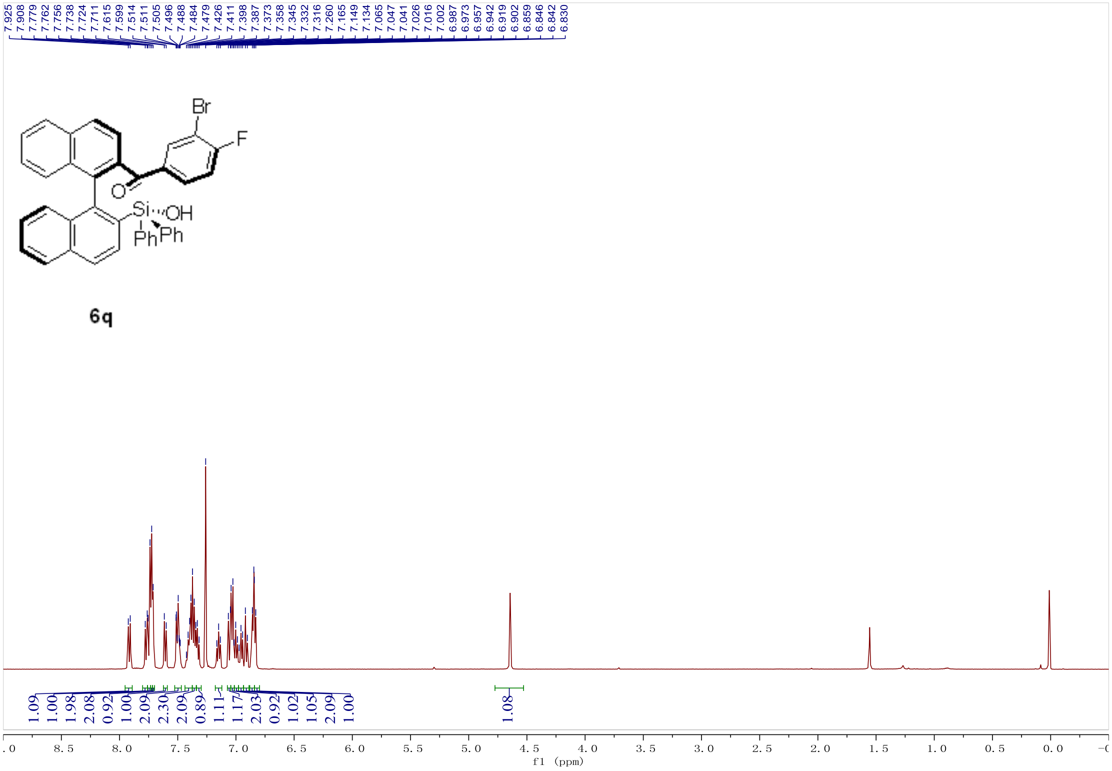

Supplementary Figure 88. ^{1}**H NMR** spectroscopy of **6q**

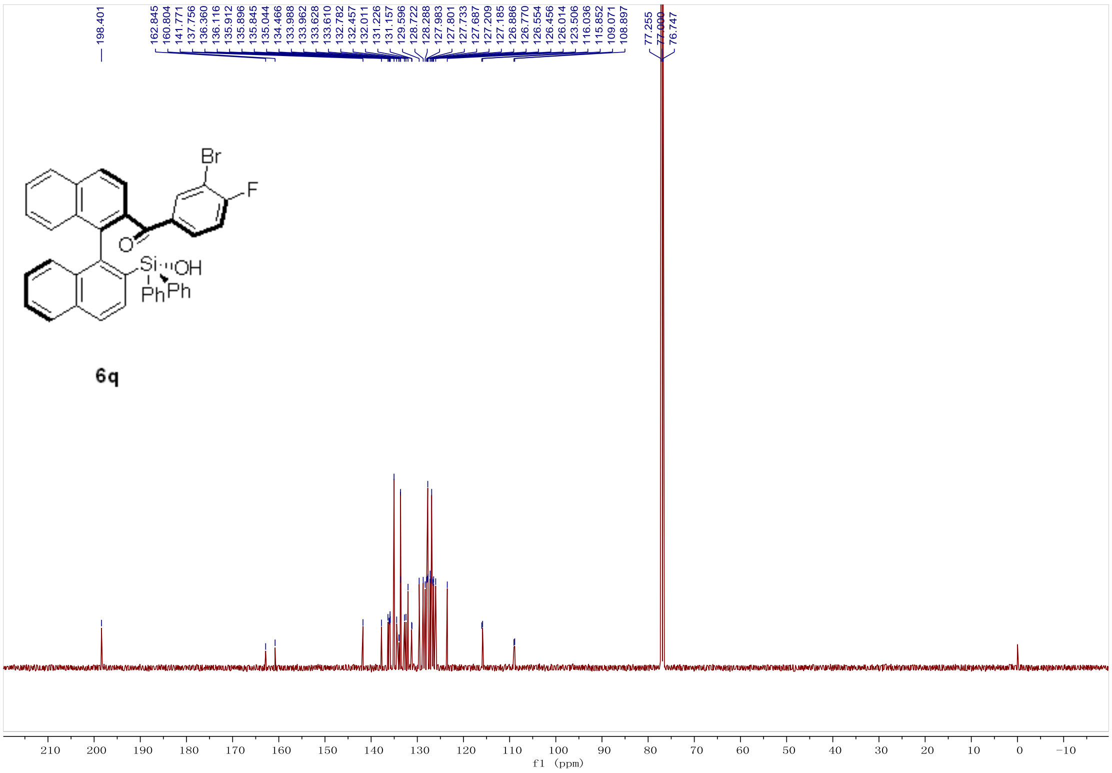

Supplementary Figure 89. ^{13}C NMR spectroscopy of **6q**

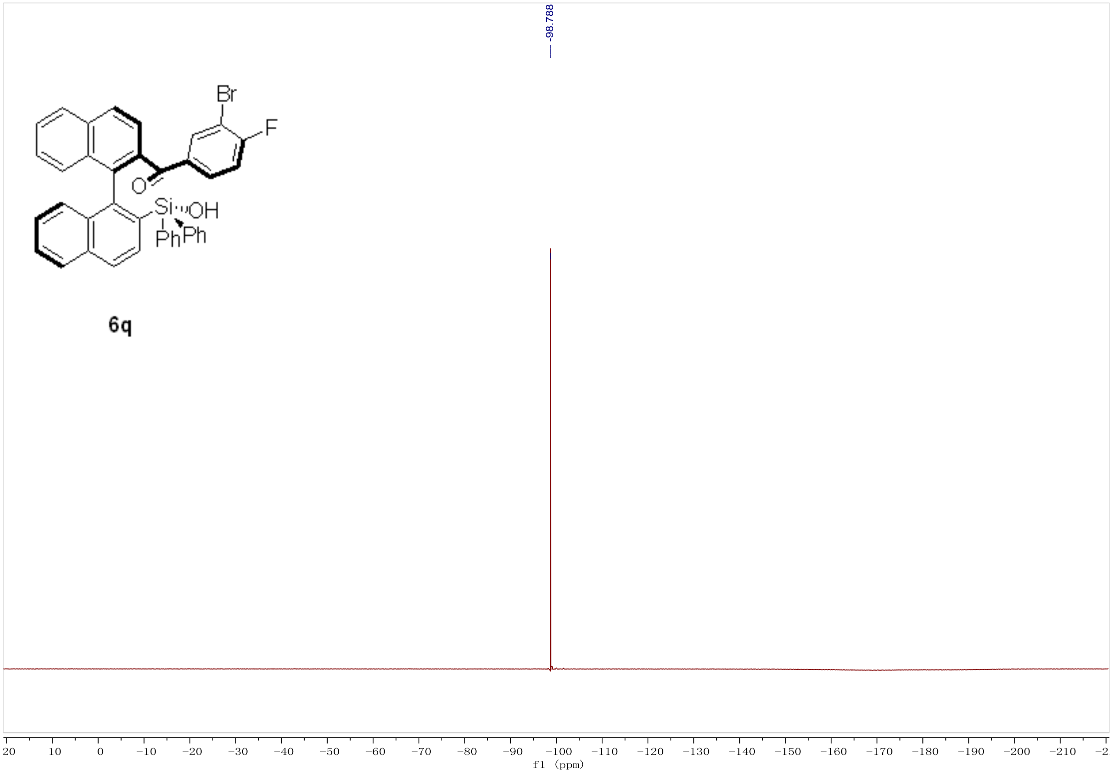

Supplementary Figure 90. ^{19}F NMR spectroscopy of **6q**

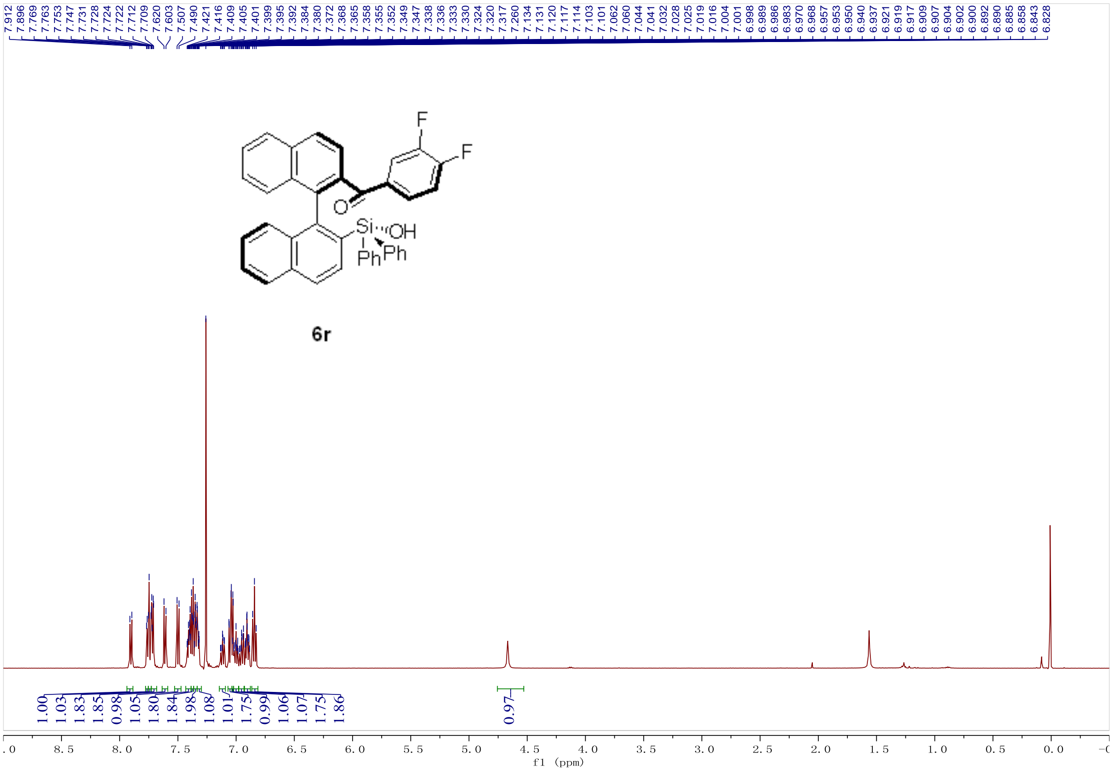

Supplementary Figure 91. **^{1}H NMR** spectroscopy of **6r**

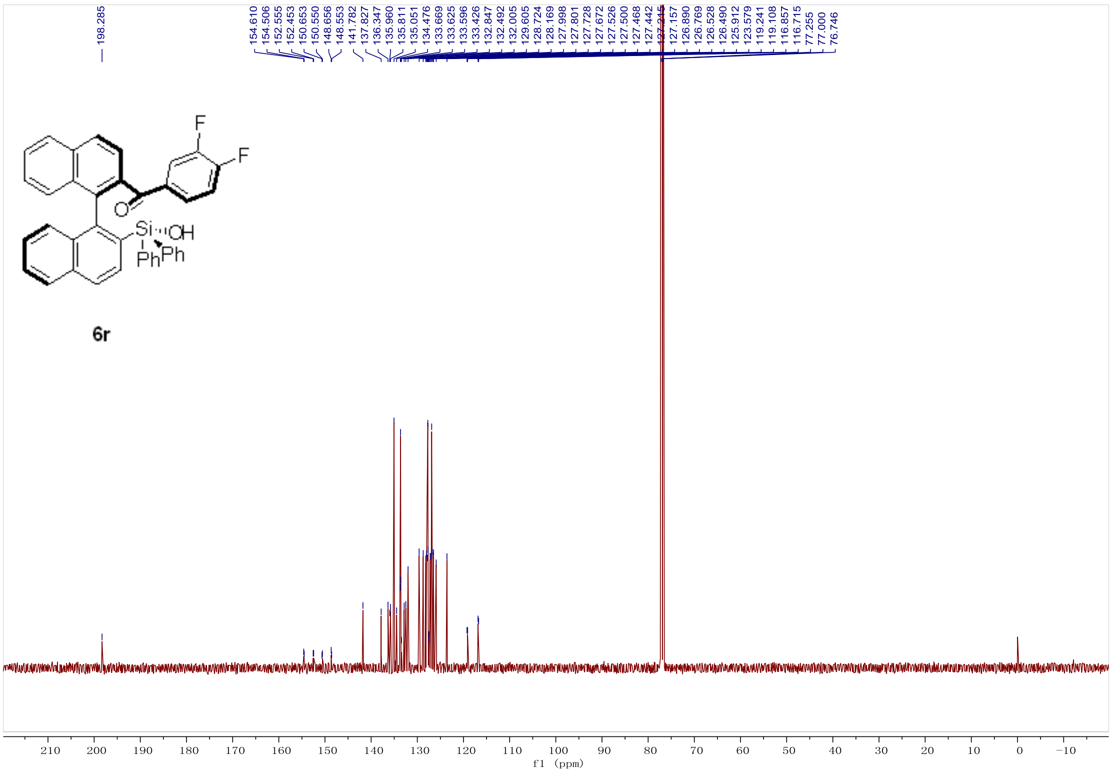

Supplementary Figure 92. **^{13}C NMR** spectroscopy of **6r**

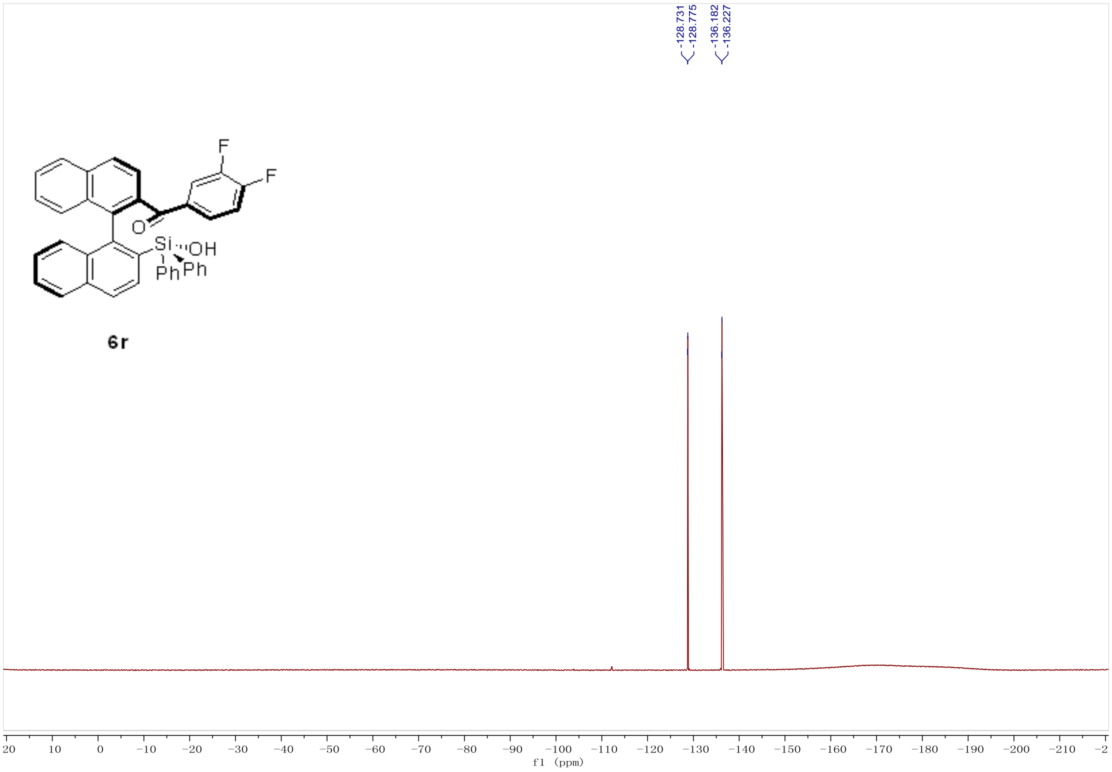

Supplementary Figure 93. ^{19}**F NMR** spectroscopy of **6r**

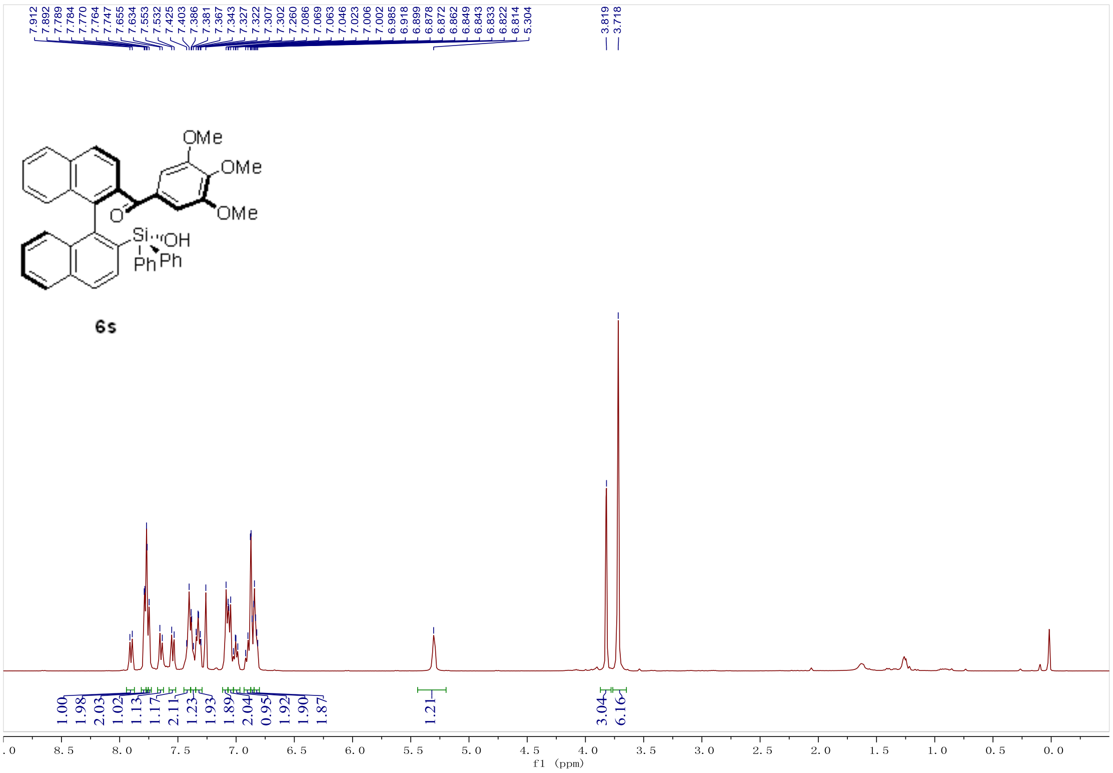

Supplementary Figure 94. ^{1}**H NMR** spectroscopy of **6s**

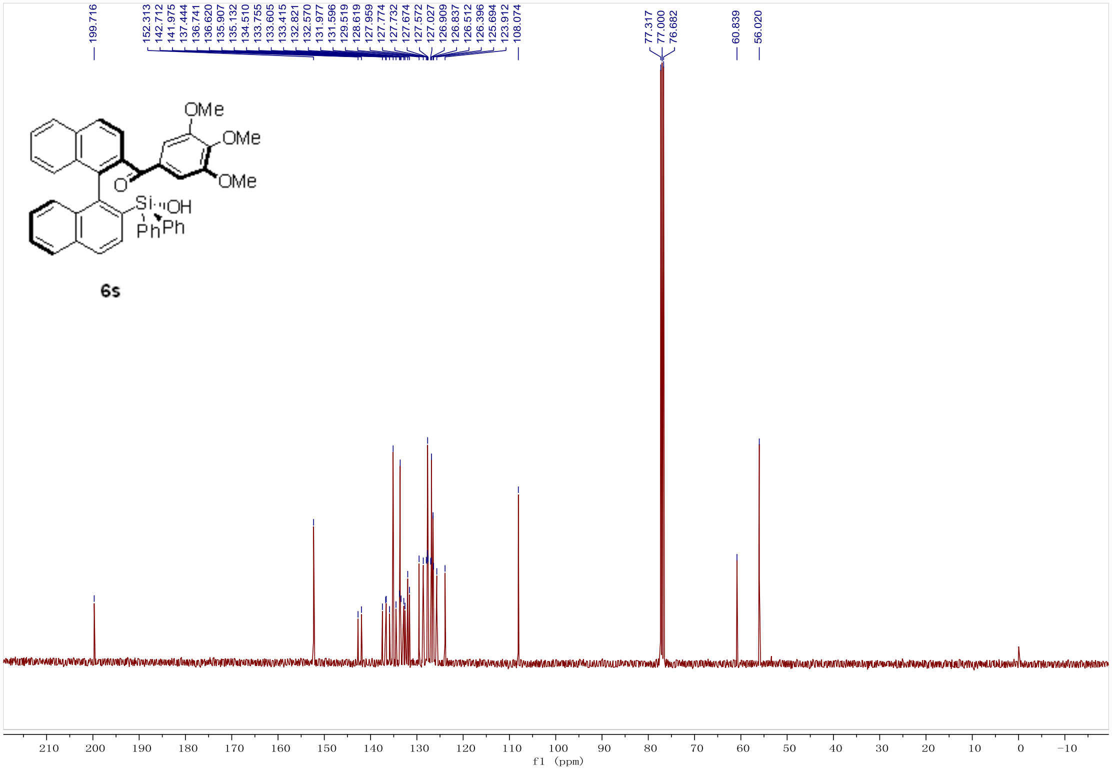

Supplementary Figure 95. ^{13}**C NMR** spectroscopy of **6s**

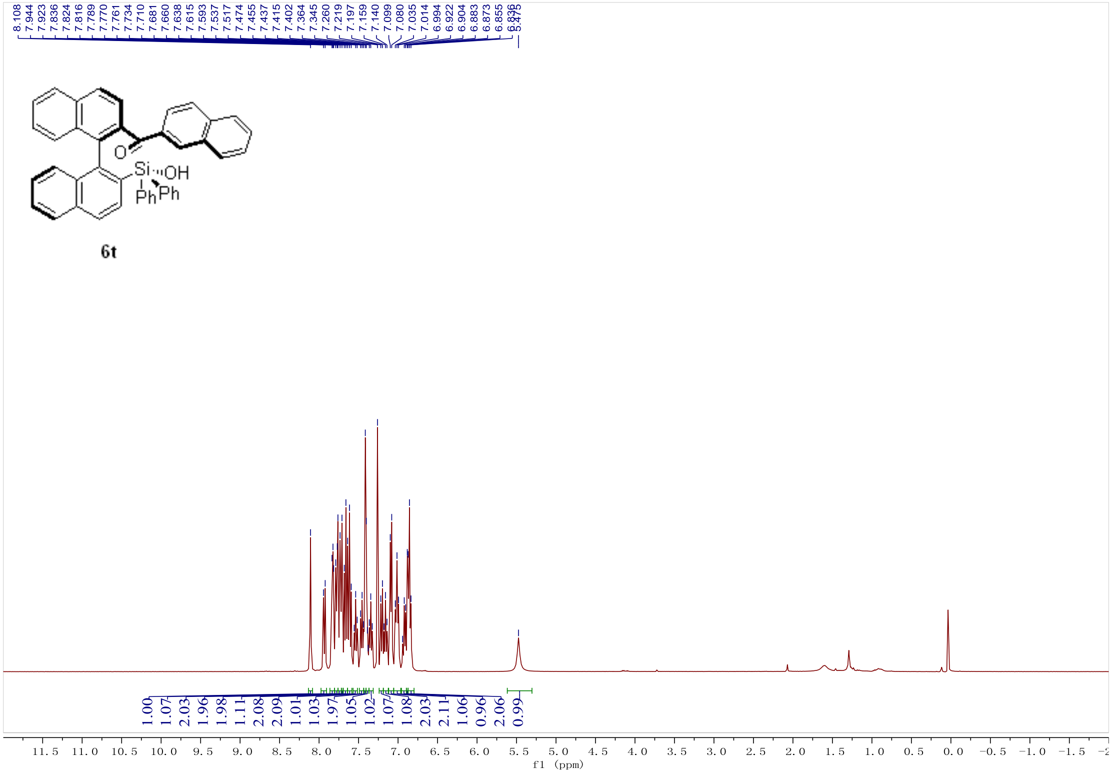

Supplementary Figure 96. ^{1}**H NMR** spectroscopy of **6t**

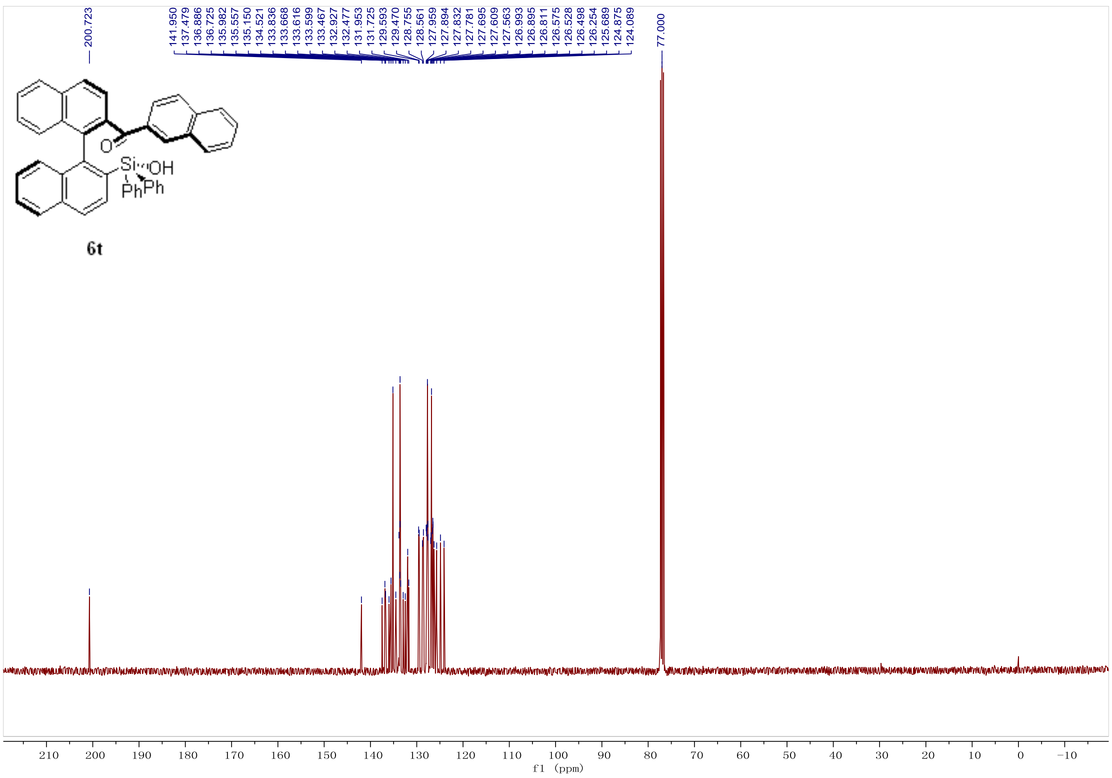

Supplementary Figure 97. ^{13}**C NMR** spectroscopy of **6t**

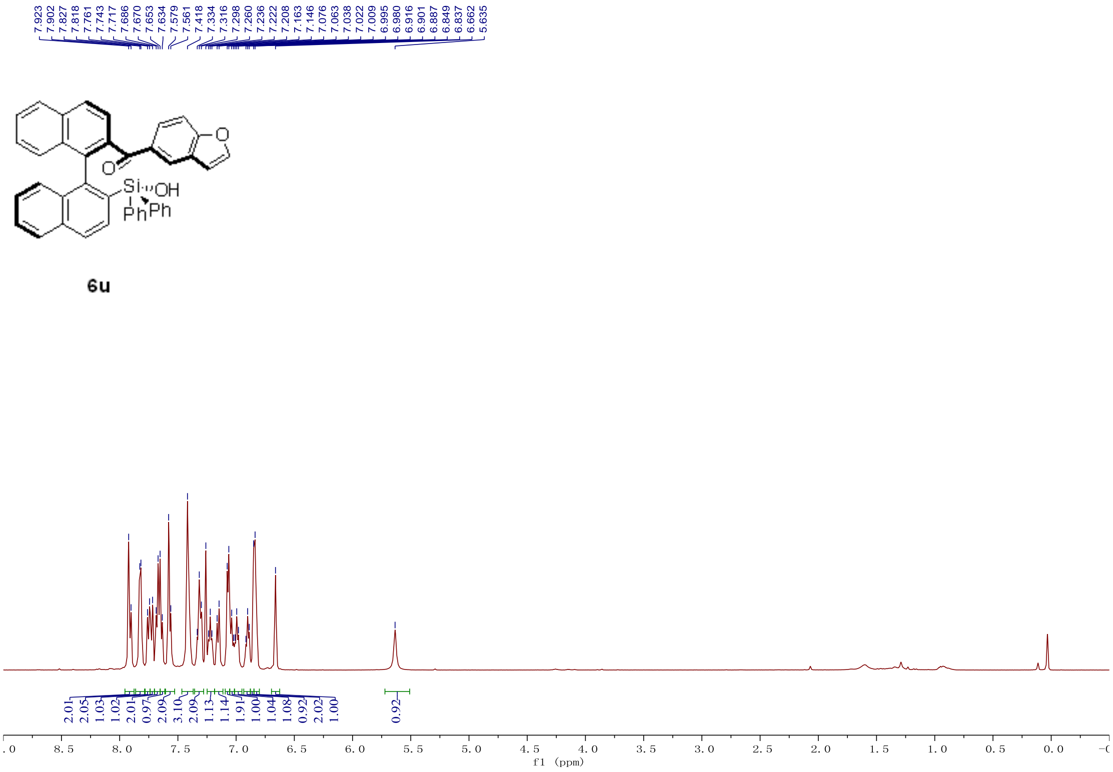

Supplementary Figure 98. ^{1}**H NMR** spectroscopy of **6u**

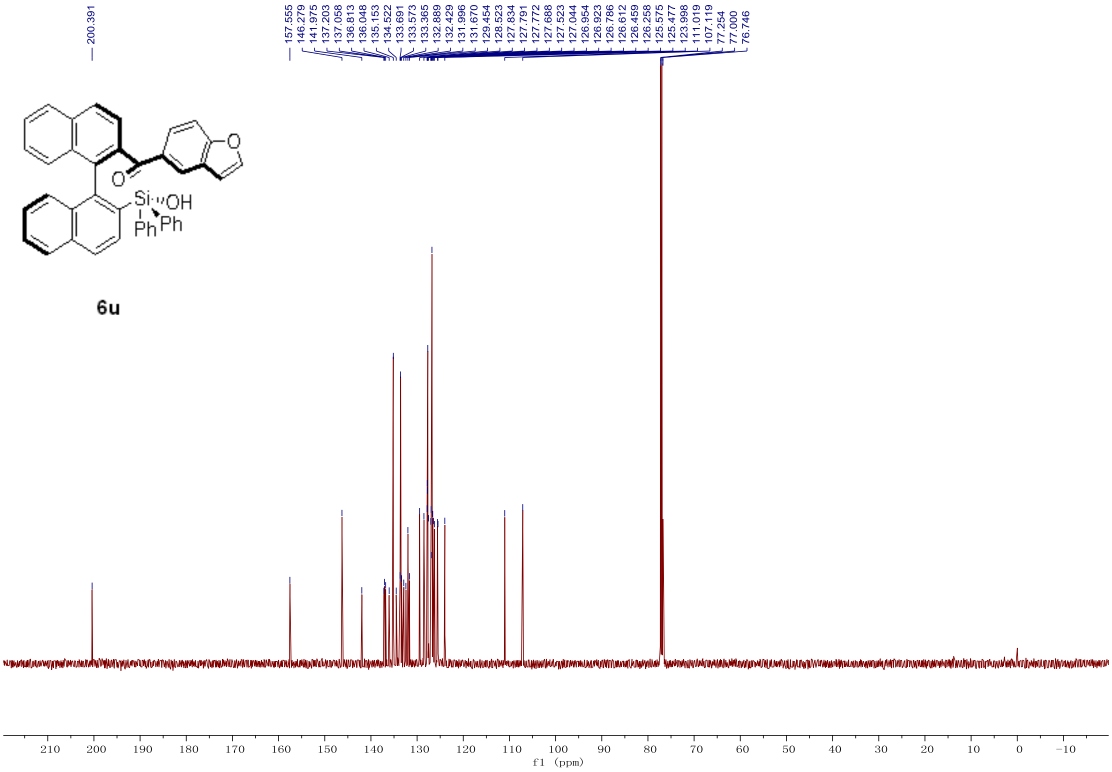

Supplementary Figure 99. ^{13}C NMR spectroscopy of **6u**

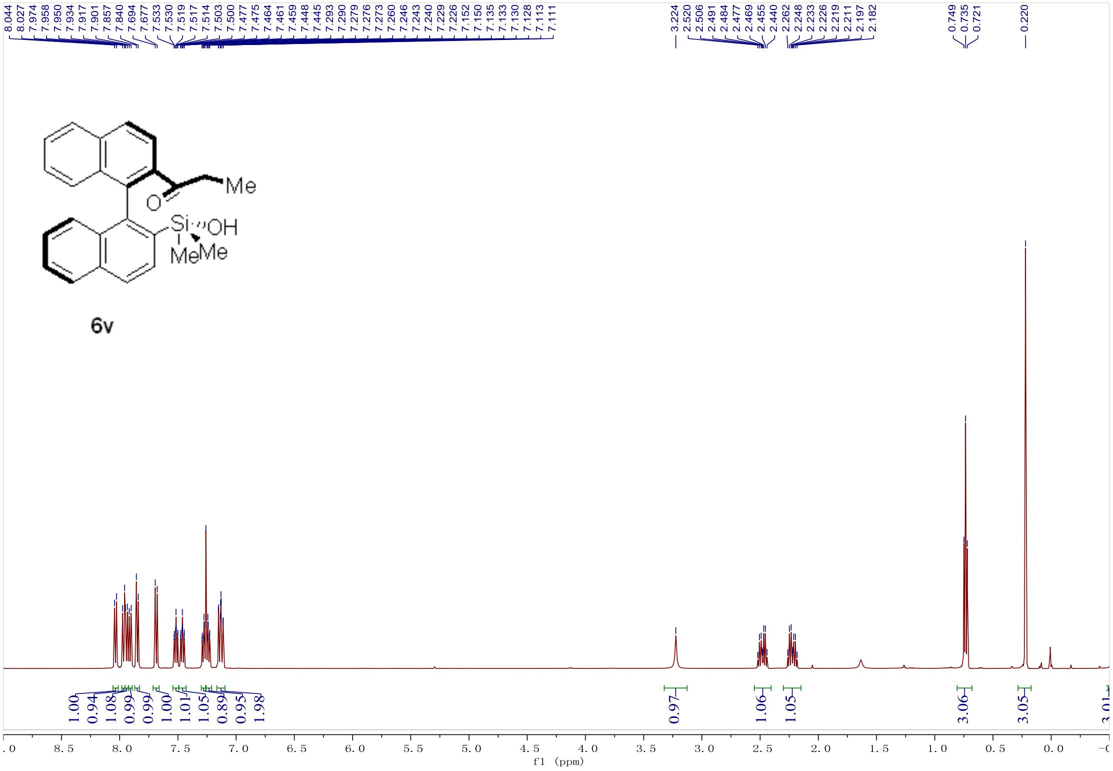

Supplementary Figure 100. ^{1}H NMR spectroscopy of **6v**

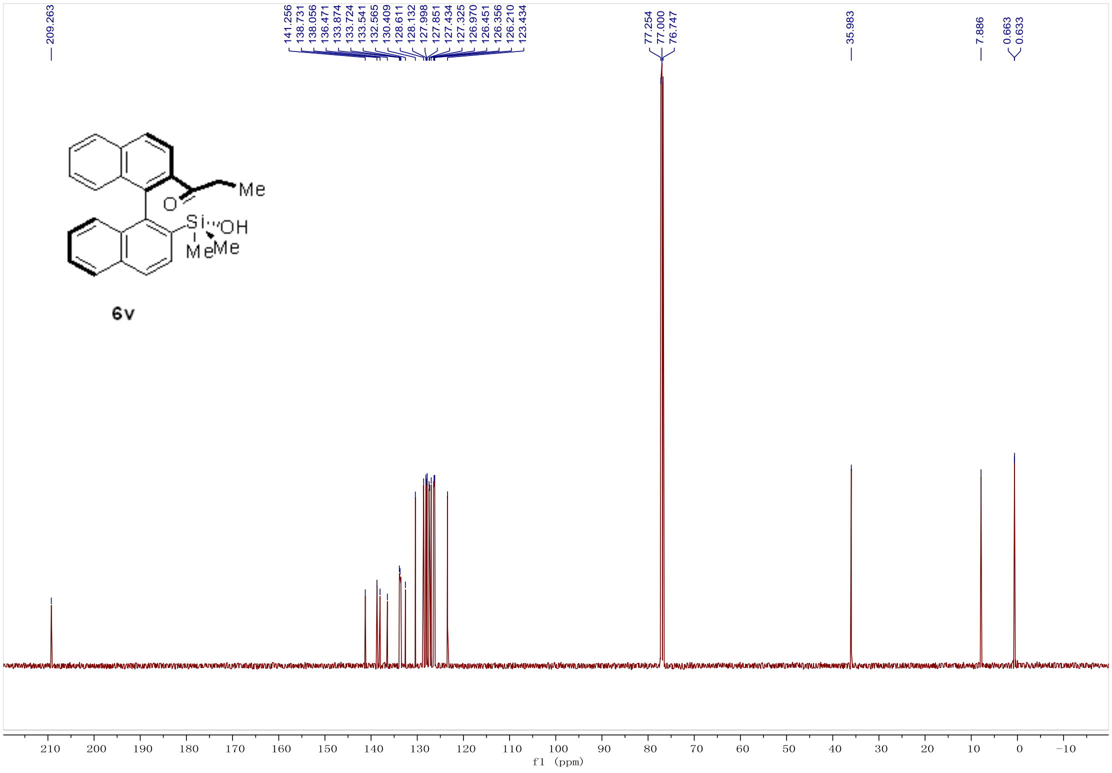

Supplementary Figure 101. ^{13}**C NMR** spectroscopy of **6v**

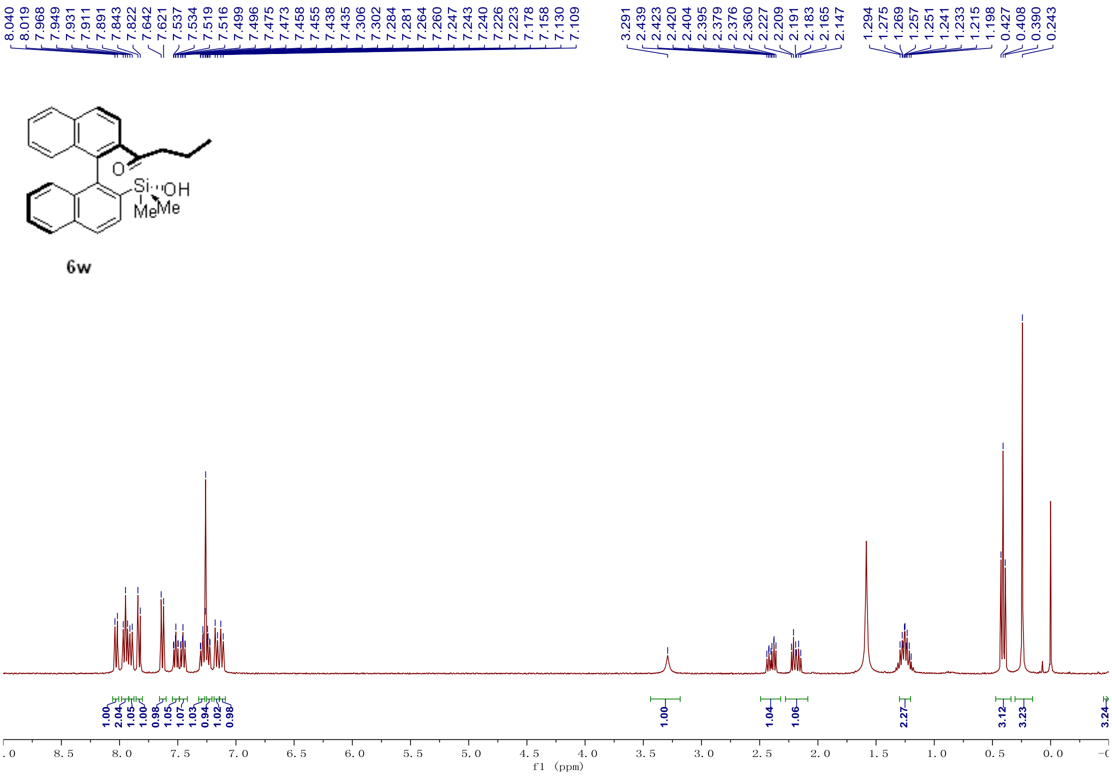

Supplementary Figure 102. ^{1}**H NMR** spectroscopy of **6w**

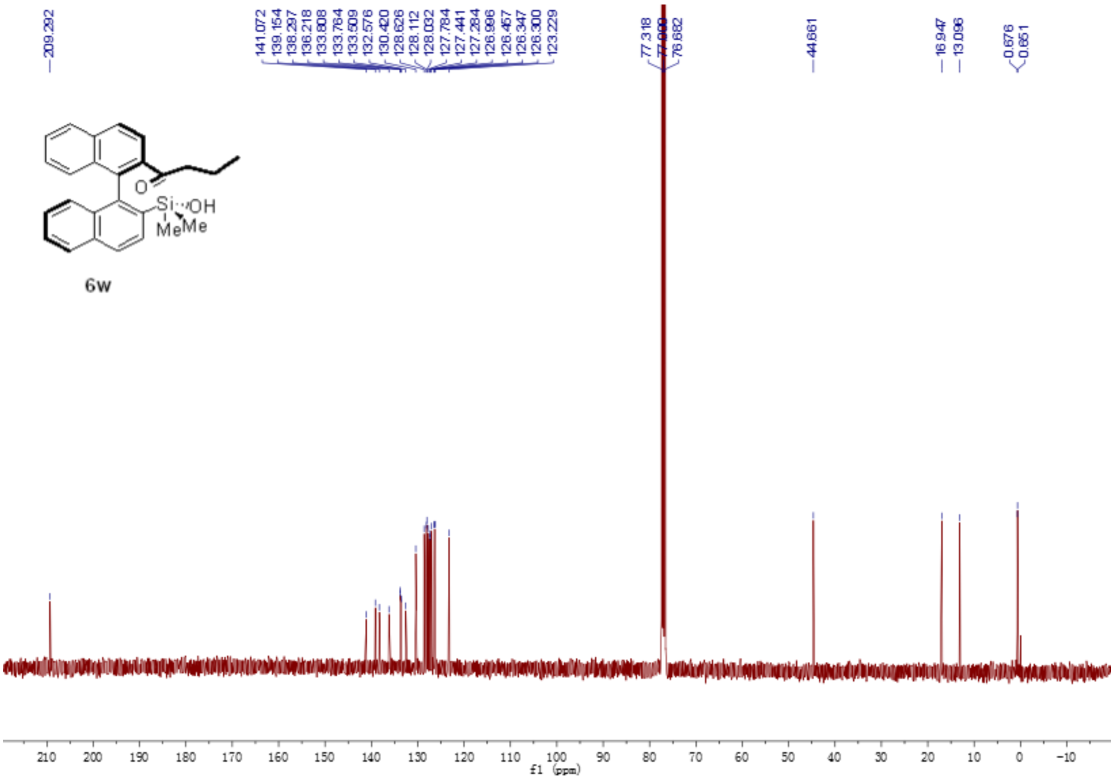

Supplementary Figure 103. ^{13}**C NMR** spectroscopy of **6w**

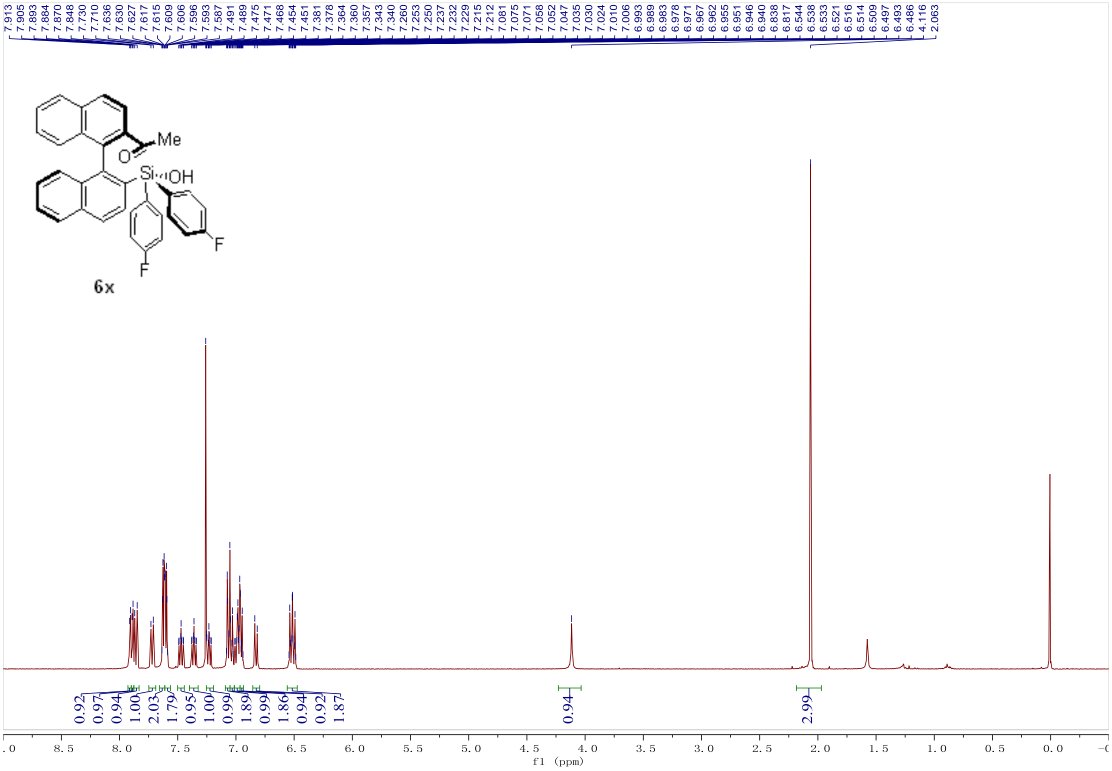

Supplementary Figure 104. ^{1}**H NMR** spectroscopy of **6x**

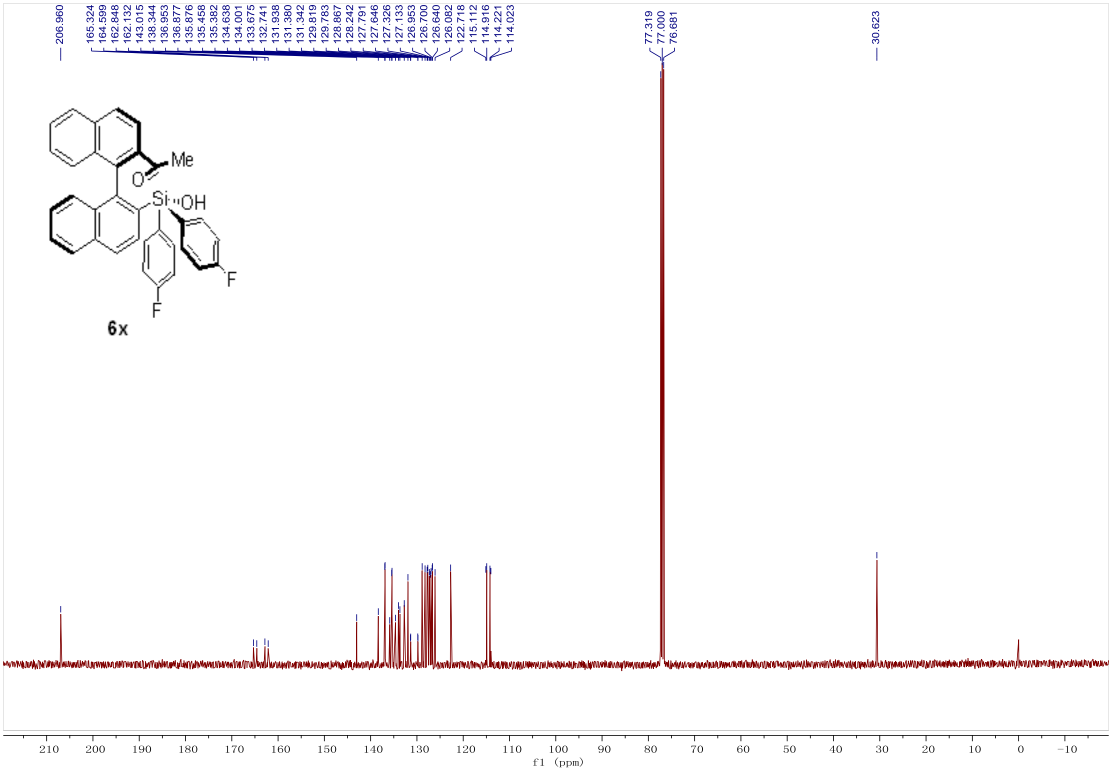

Supplementary Figure 105. ^{13}**C NMR** spectroscopy of **6x**

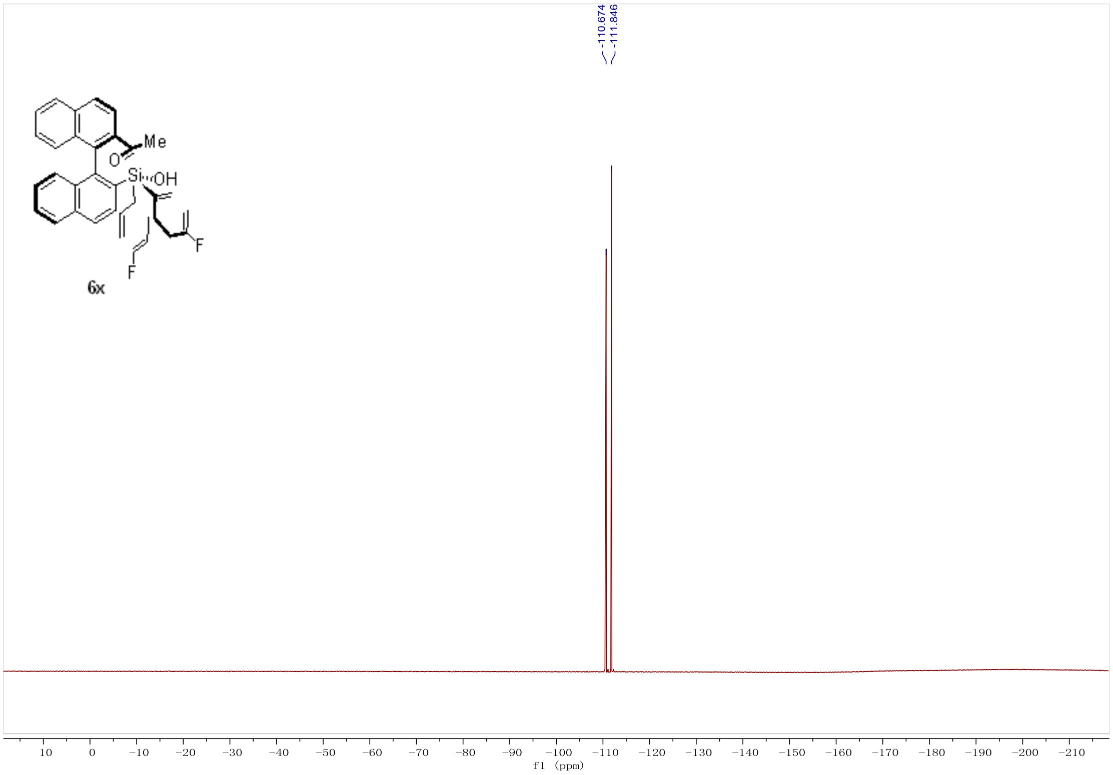

Supplementary Figure 106. ^{19}**F NMR** spectroscopy of **6x**

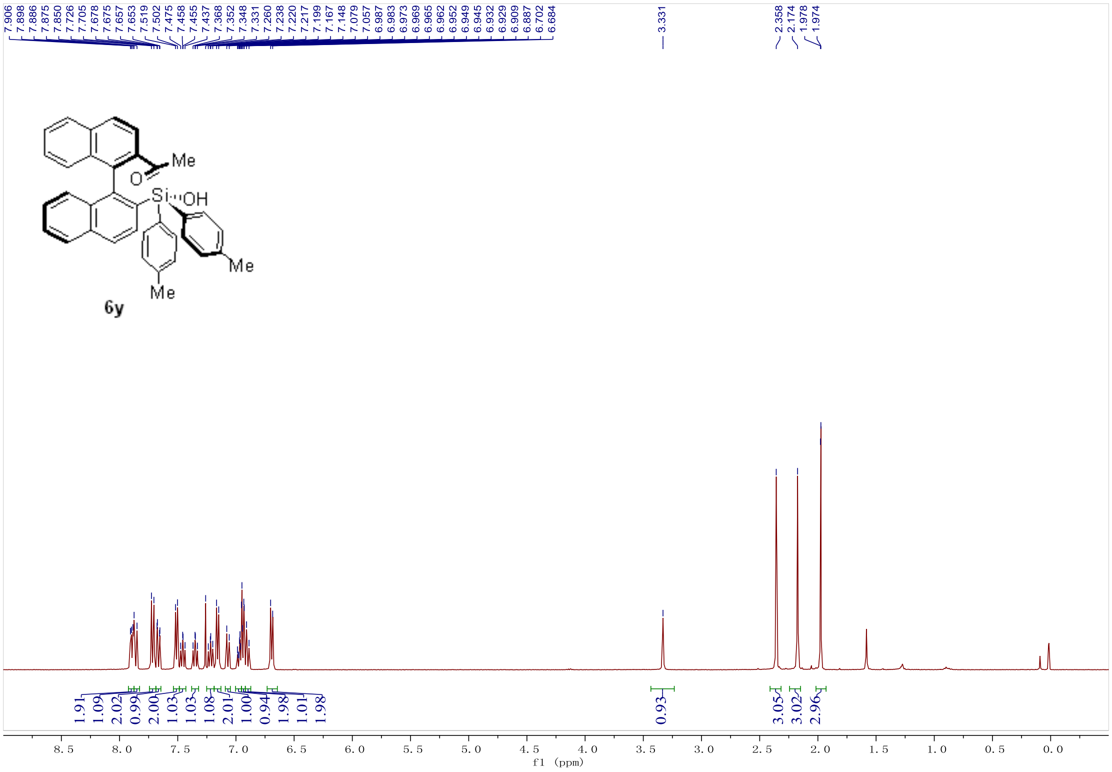

Supplementary Figure 107. ^{1}**H NMR** spectroscopy of **6y**

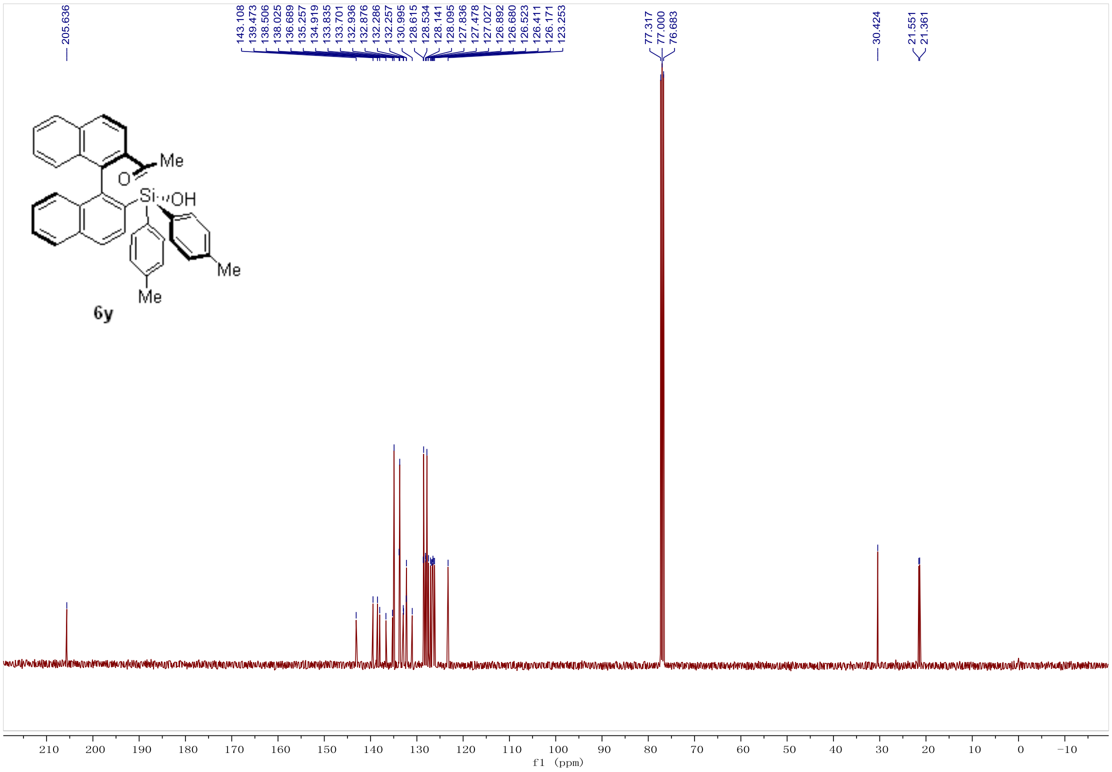

Supplementary Figure 108. ^{13}**C NMR** spectroscopy of **6y**

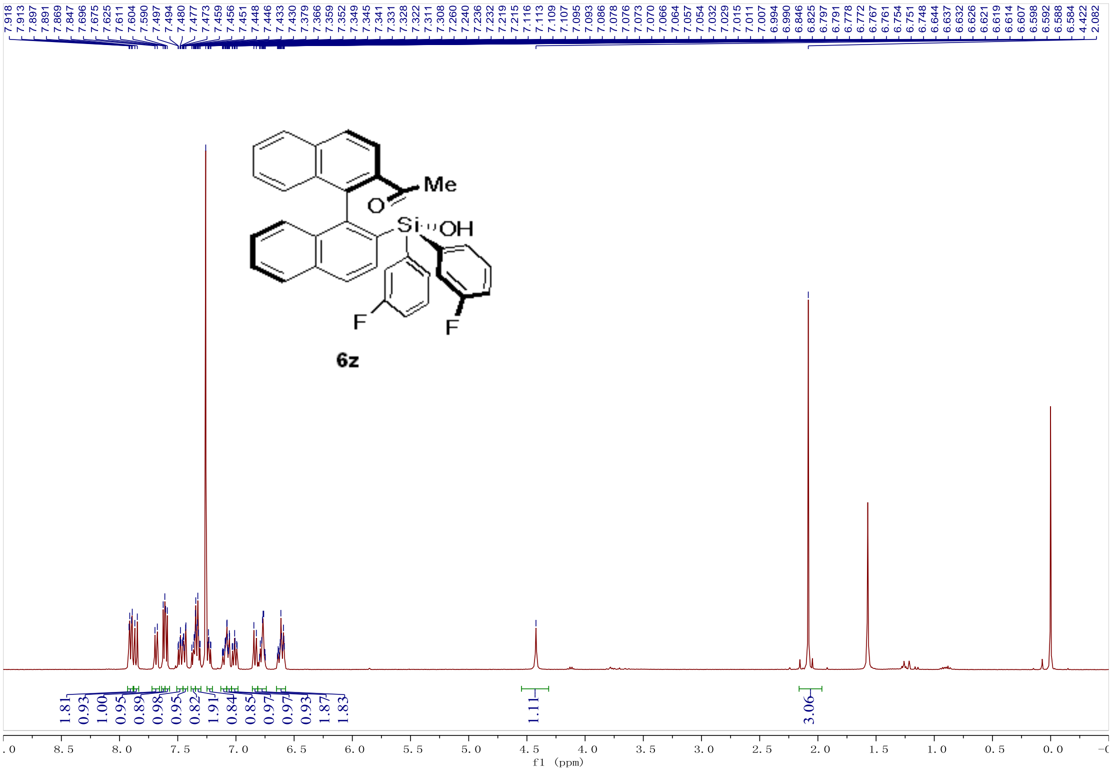

Supplementary Figure 109. ^{1}**H NMR** spectroscopy of **6z**

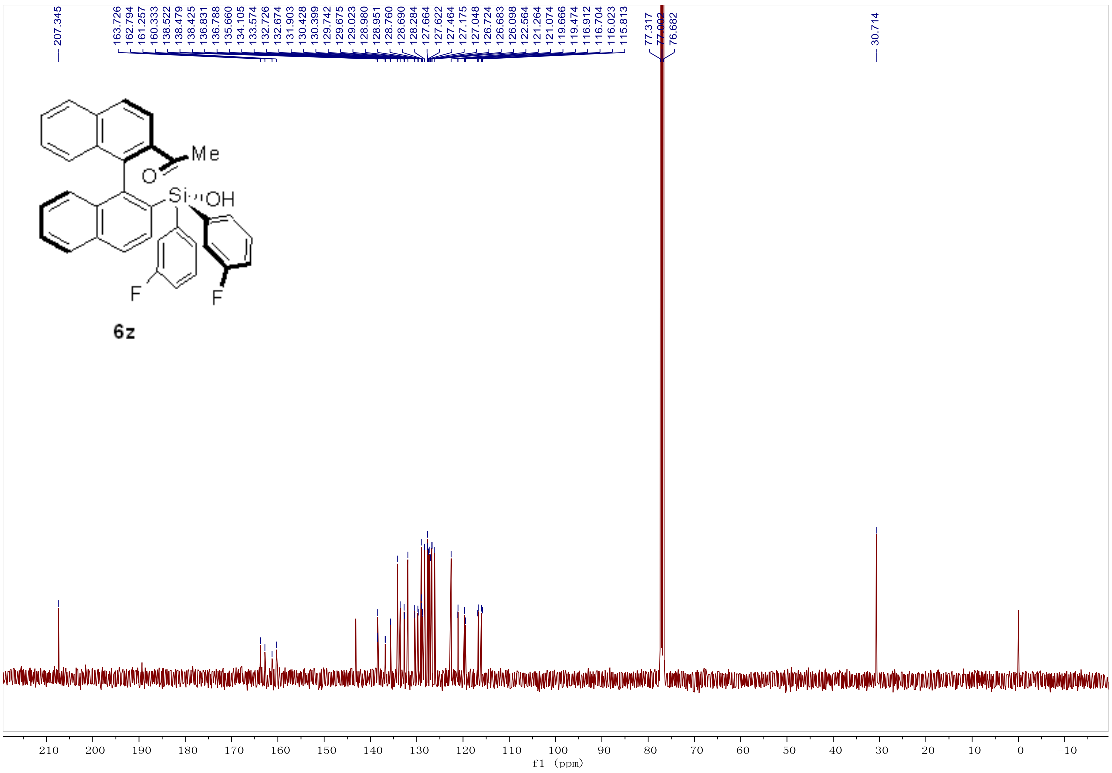

Supplementary Figure 110. ^{13}**C NMR** spectroscopy of **6z**

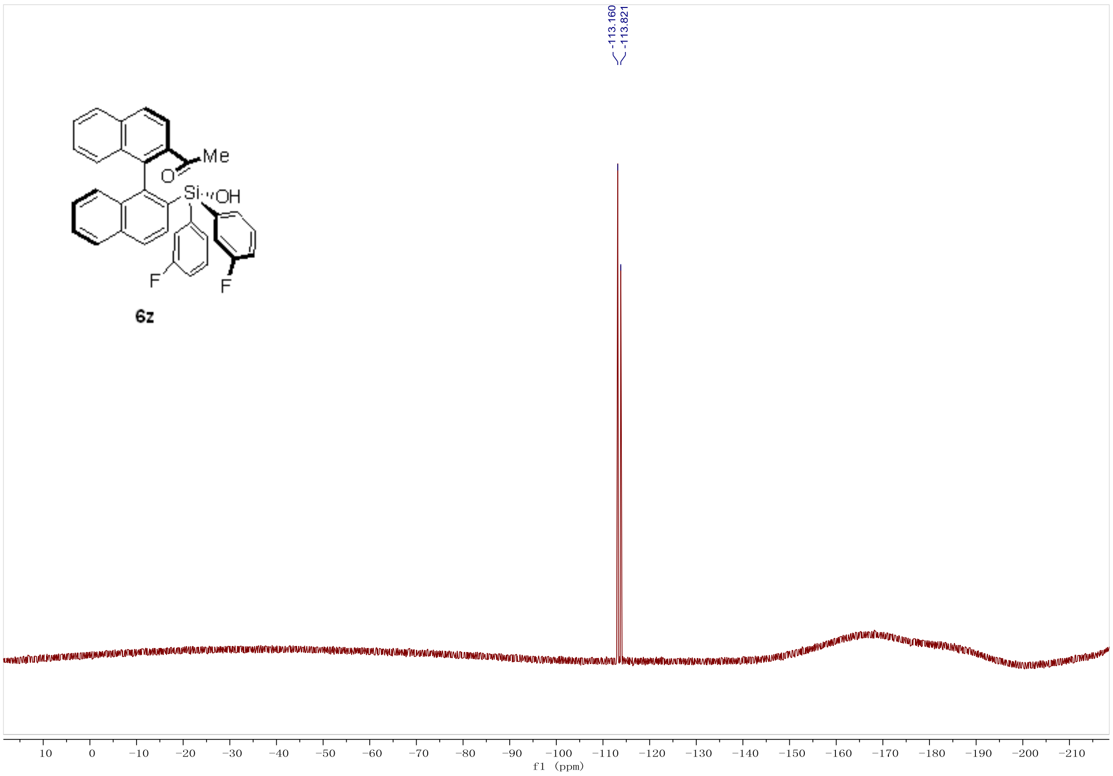

Supplementary Figure 111. ^{19}F NMR spectroscopy of **6z**

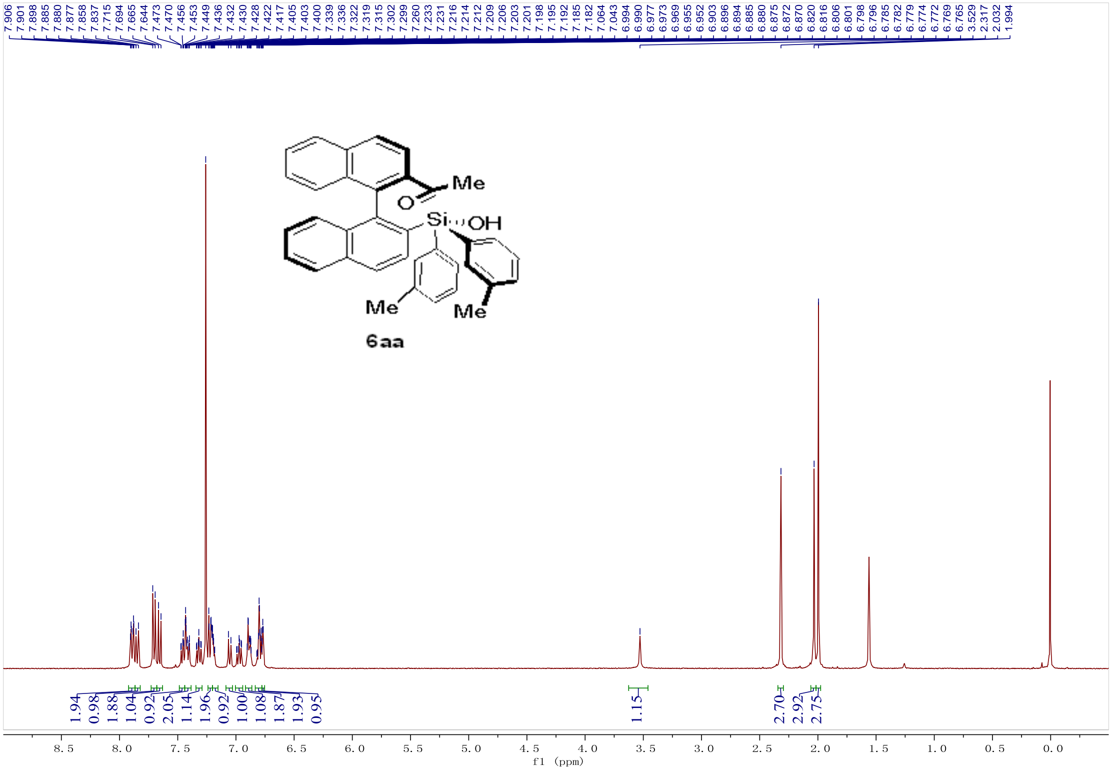

Supplementary Figure 112. ^{1}H NMR spectroscopy of **6aa**

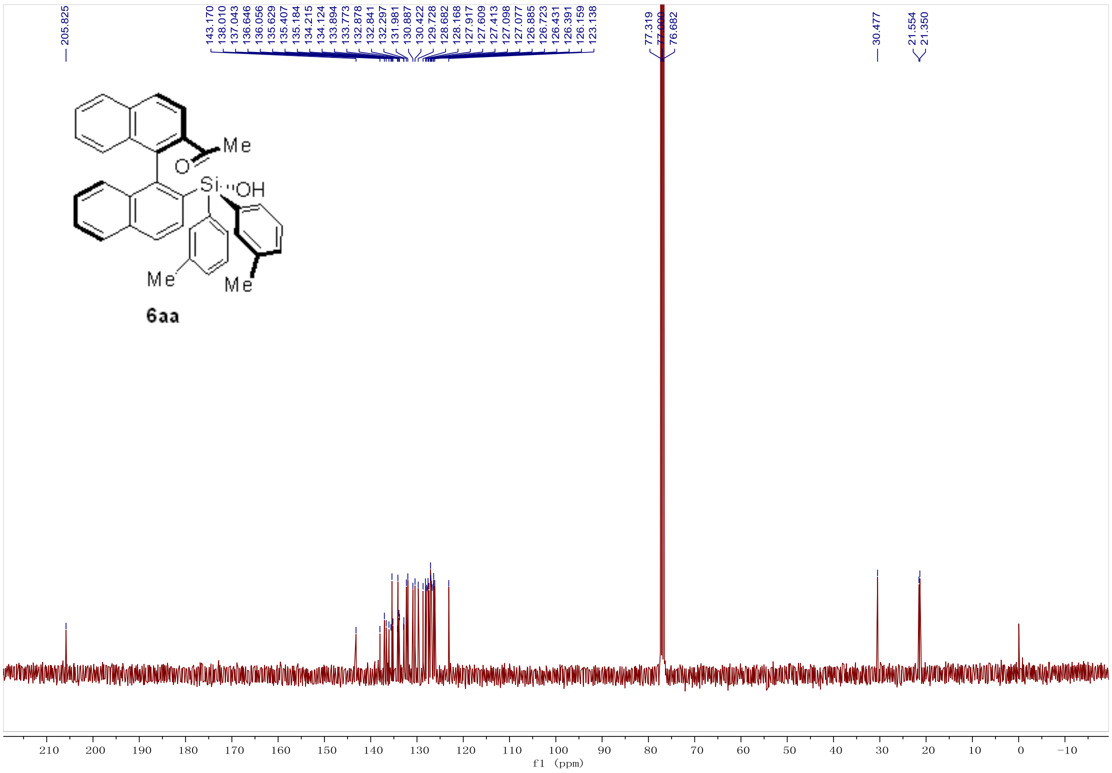

Supplementary Figure 113. ^{13}**C NMR** spectroscopy of **6aa**

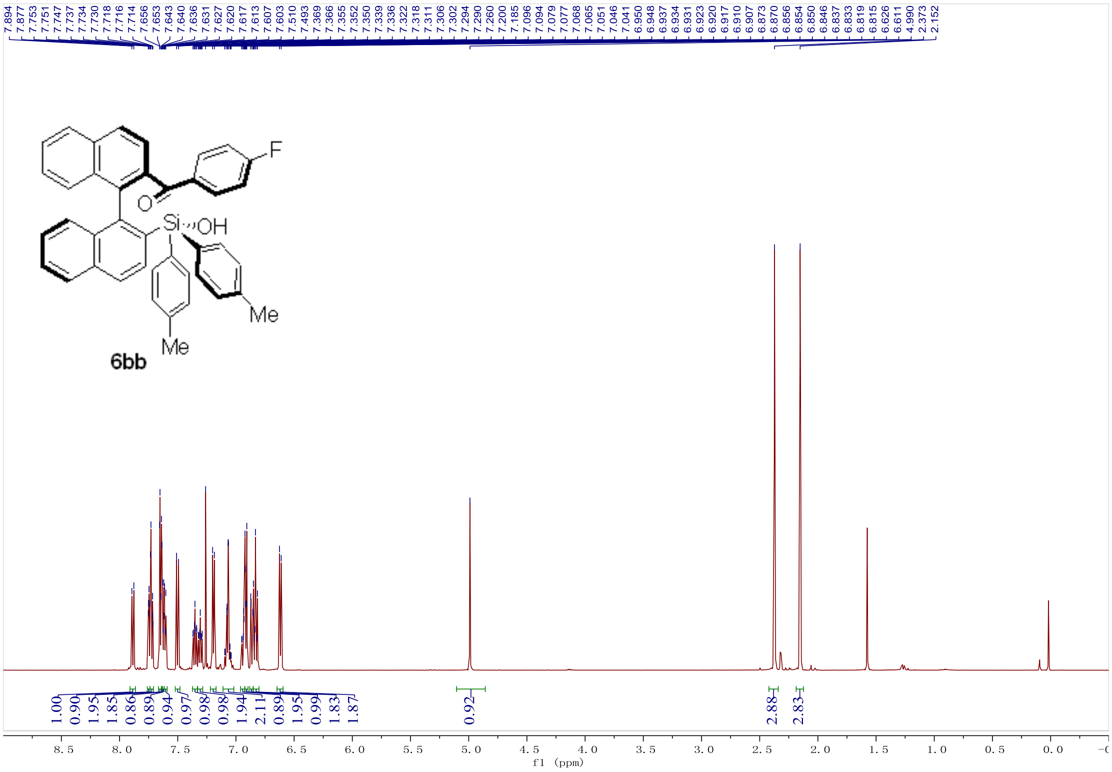

Supplementary Figure 114. ^{1}**H NMR** spectroscopy of **6bb**

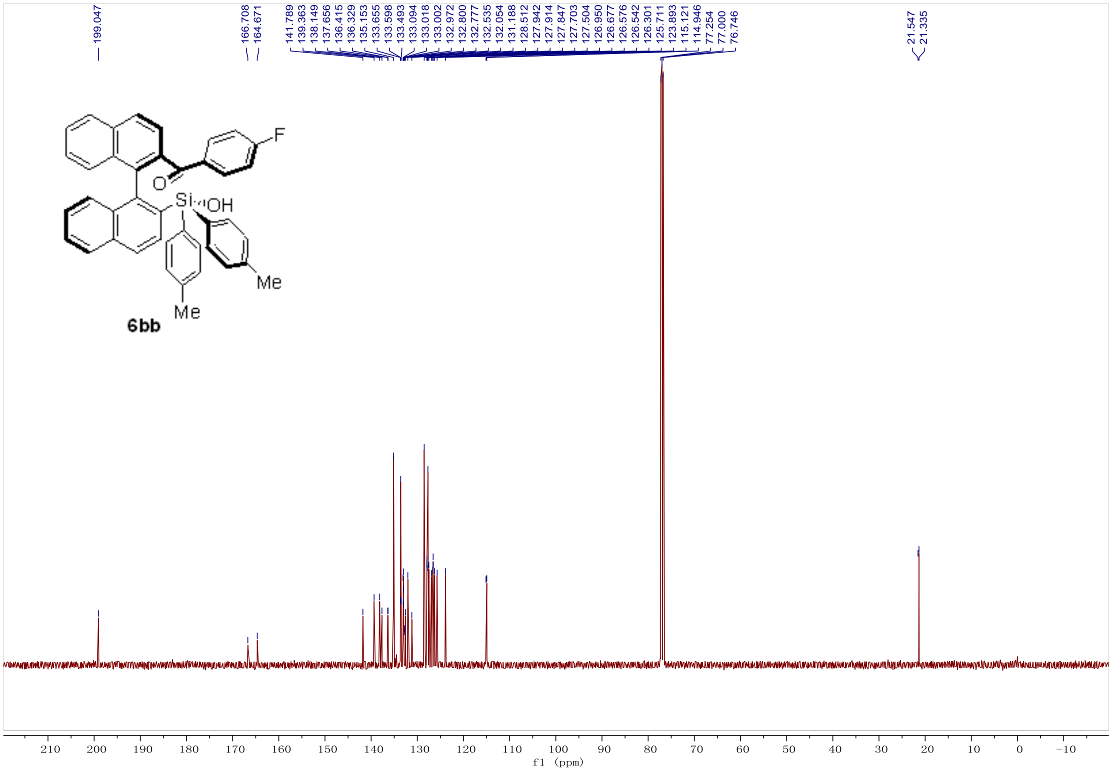

Supplementary Figure 115. ^{13}**C NMR** spectroscopy of **6bb**

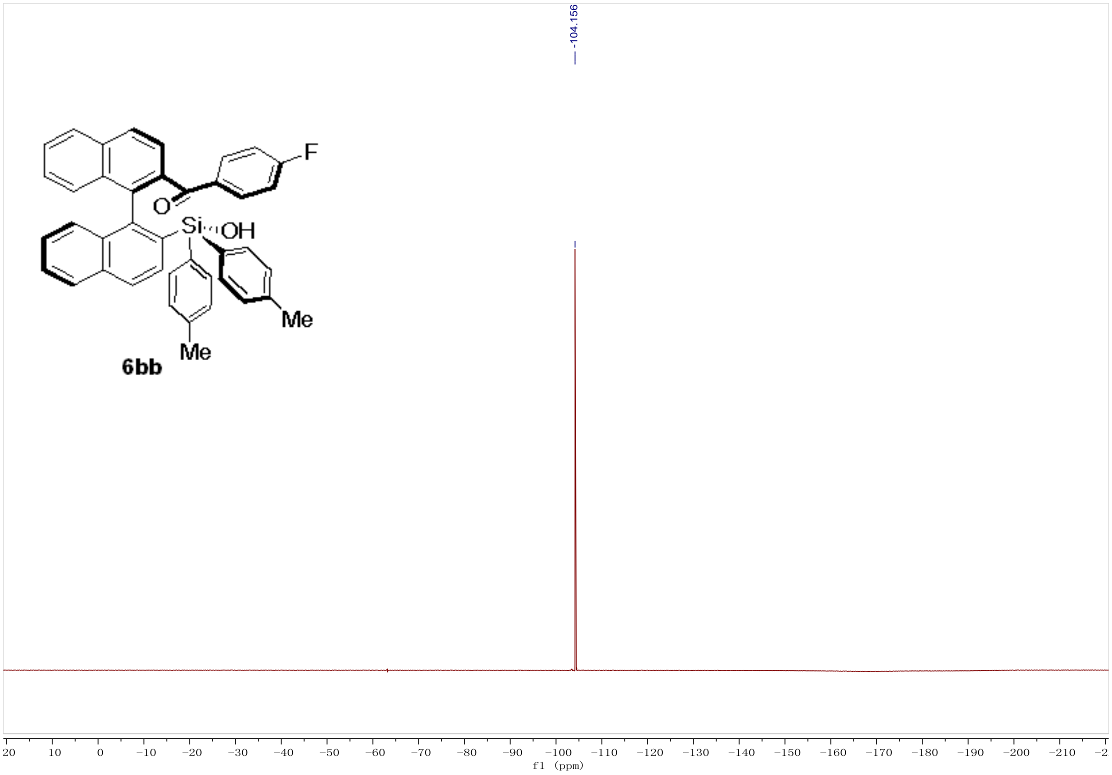

Supplementary Figure 116. ^{19}**F NMR** spectroscopy of **6bb**

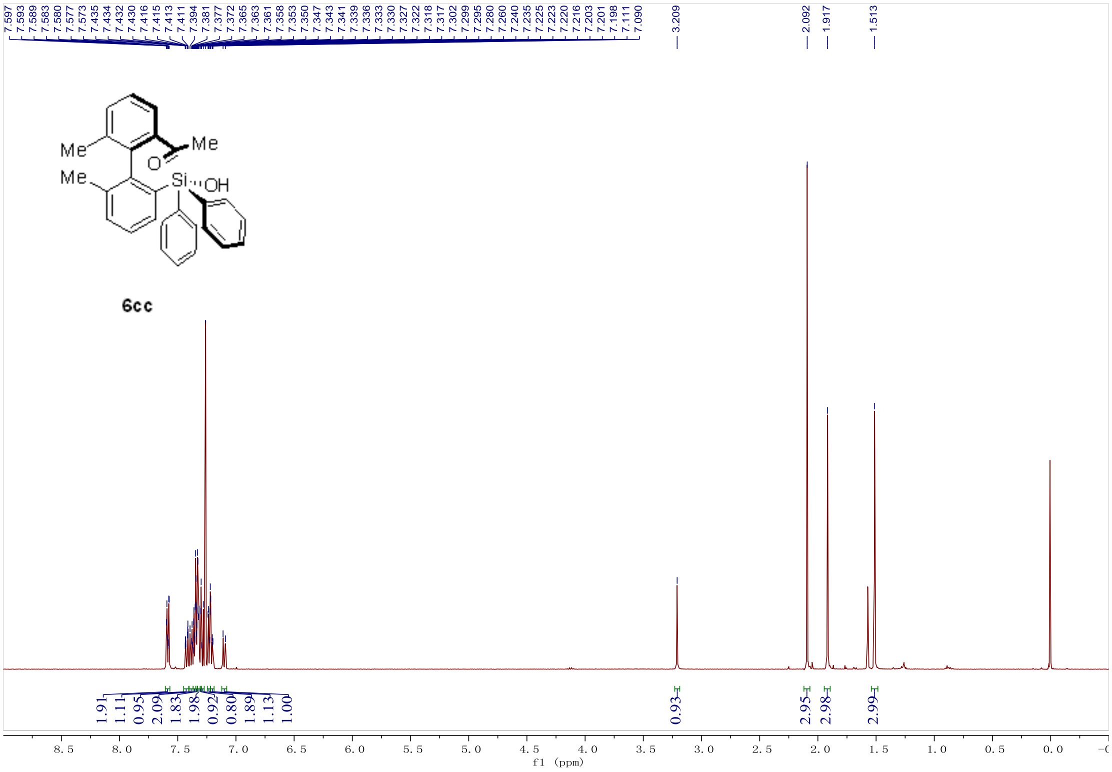

Supplementary Figure 117. ^{1}**H NMR** spectroscopy of **6cc**

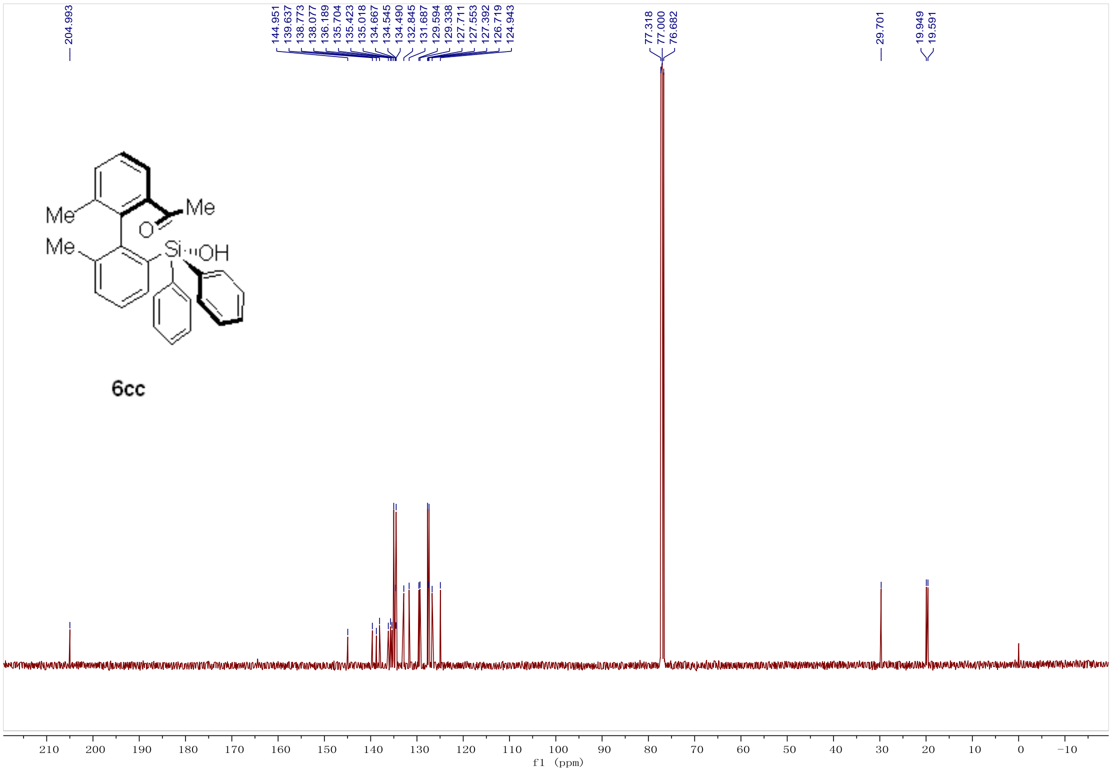

Supplementary Figure 118. ^{13}**C NMR** spectroscopy of **6cc**

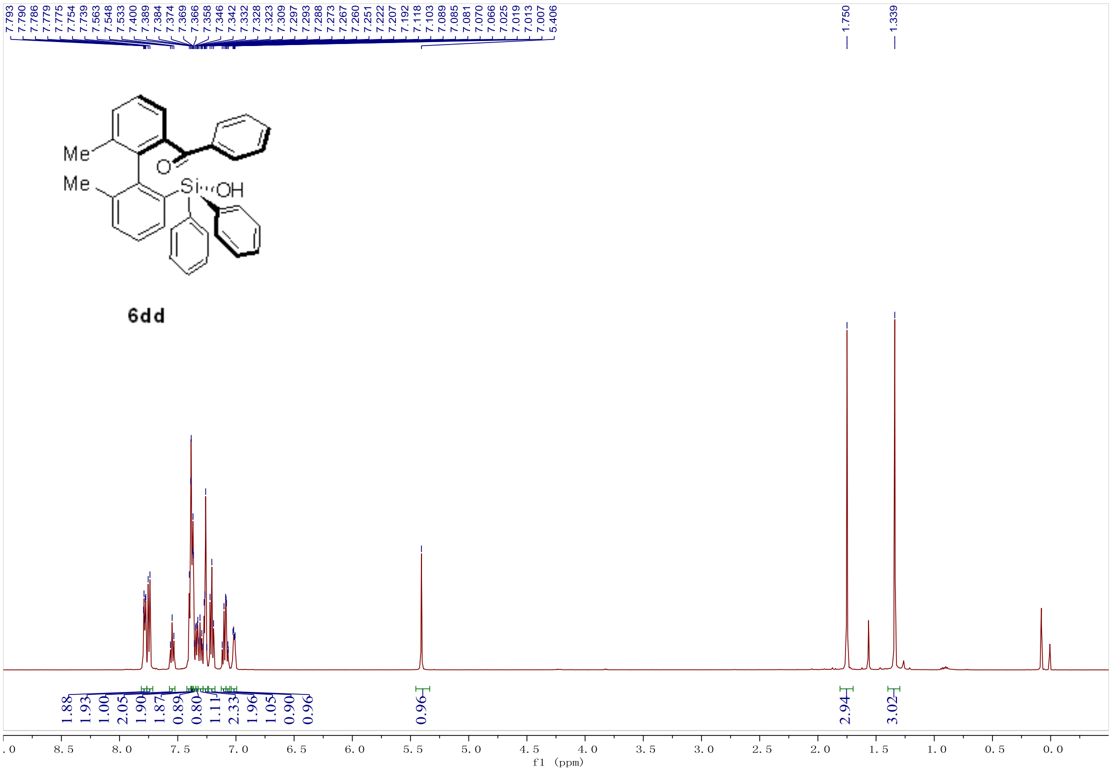

Supplementary Figure 119. ^{1}**H NMR** spectroscopy of **6dd**

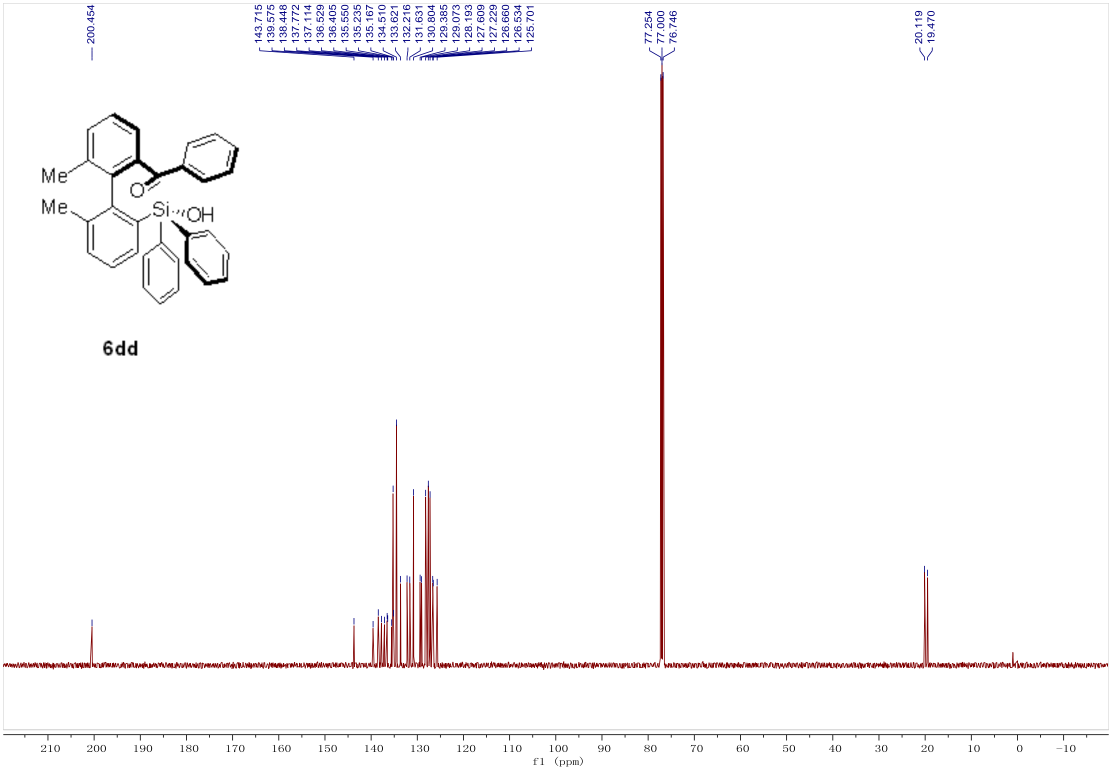

Supplementary Figure 120. ^{13}**C NMR** spectroscopy of **6dd**

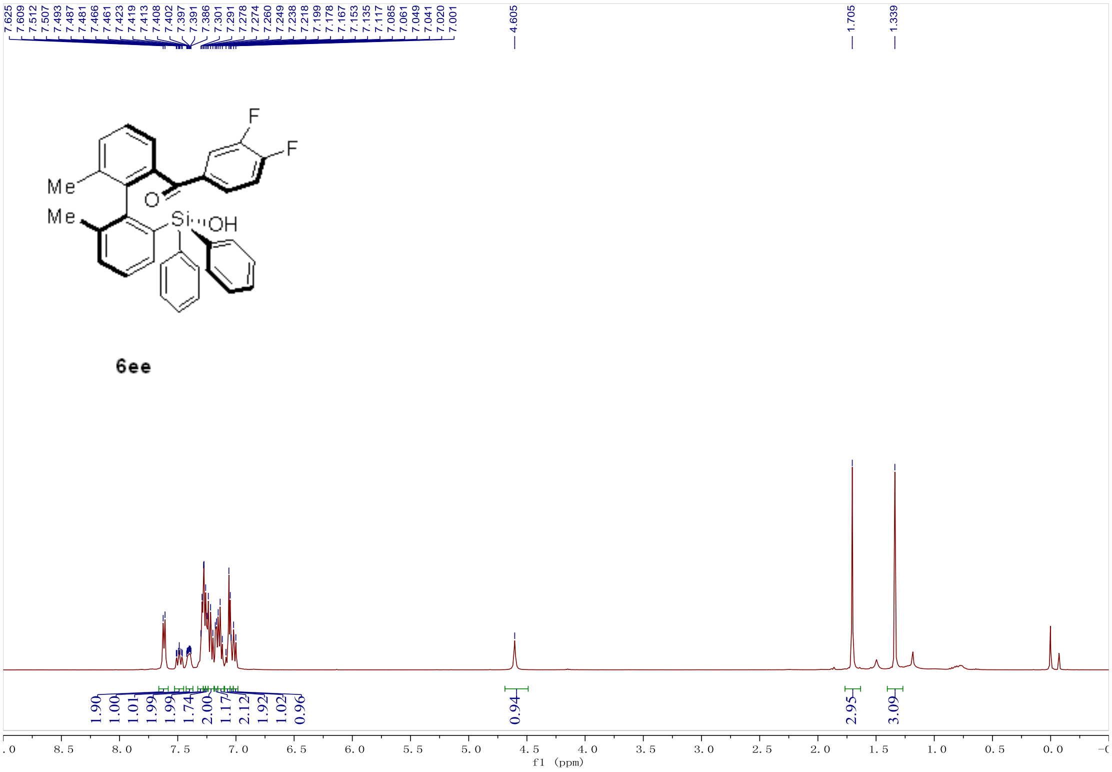

Supplementary Figure 121. ^{1}**H NMR** spectroscopy of **6ee**

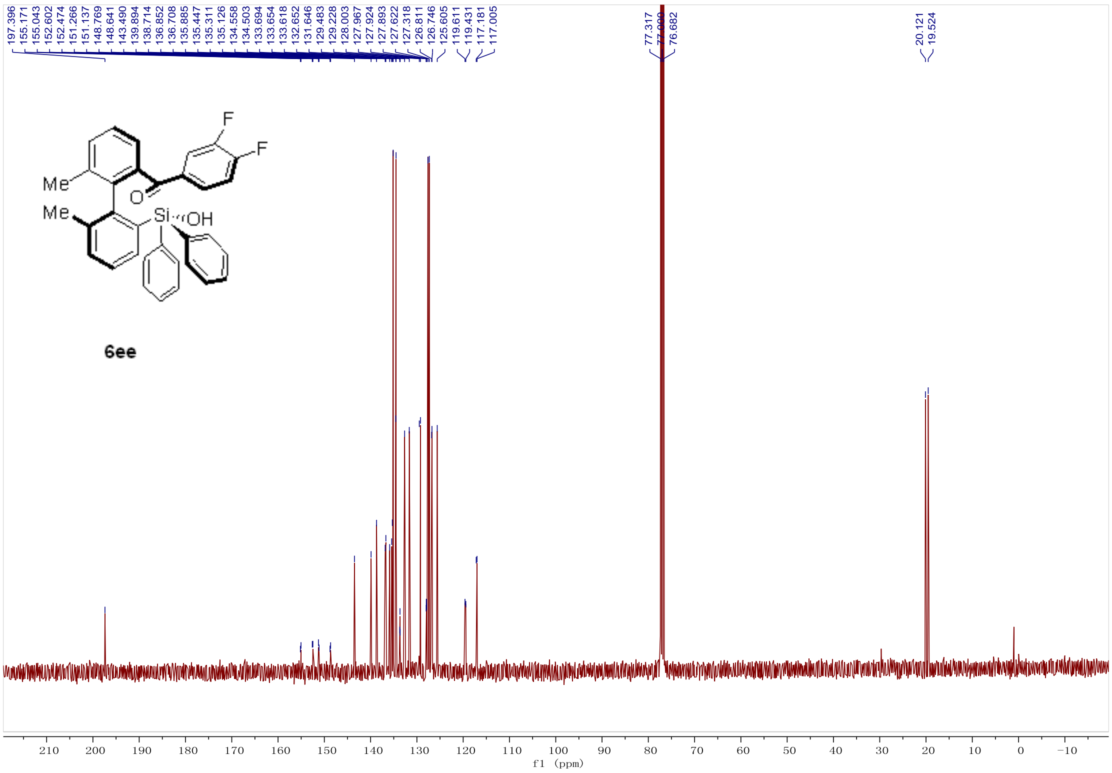

Supplementary Figure 122. ^{13}**C NMR** spectroscopy of **6ee**

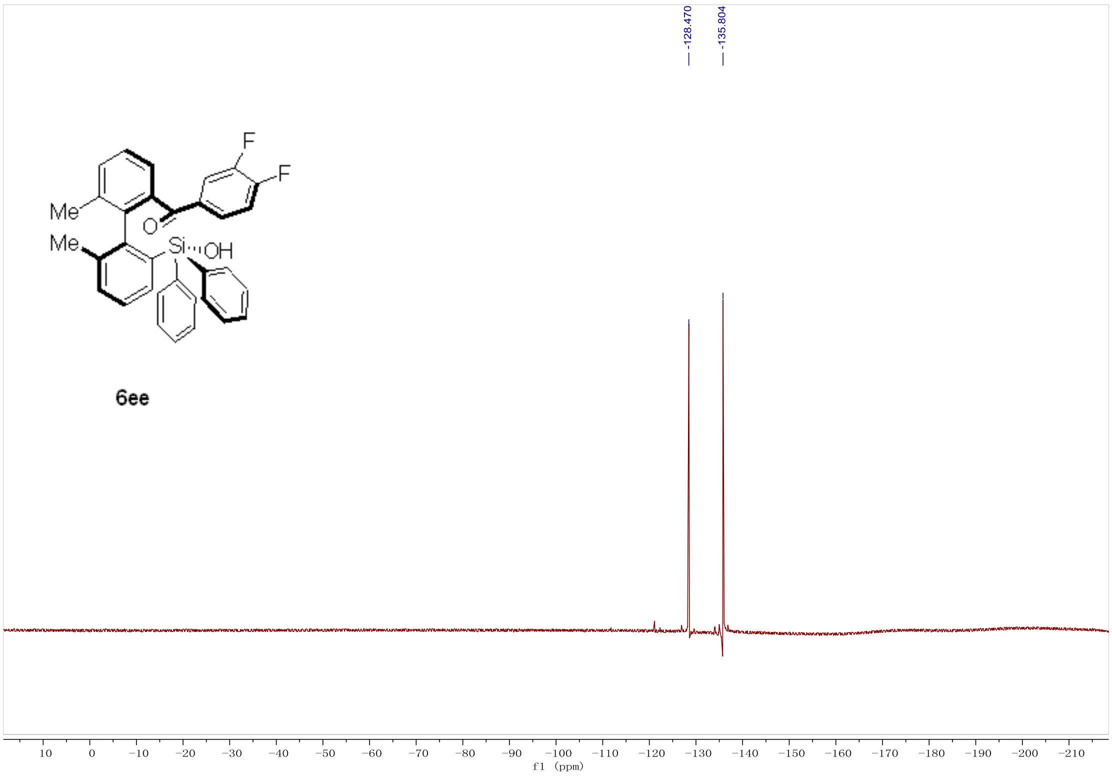

Supplementary Figure 123. ^{19}**F NMR** spectroscopy of **6ee**

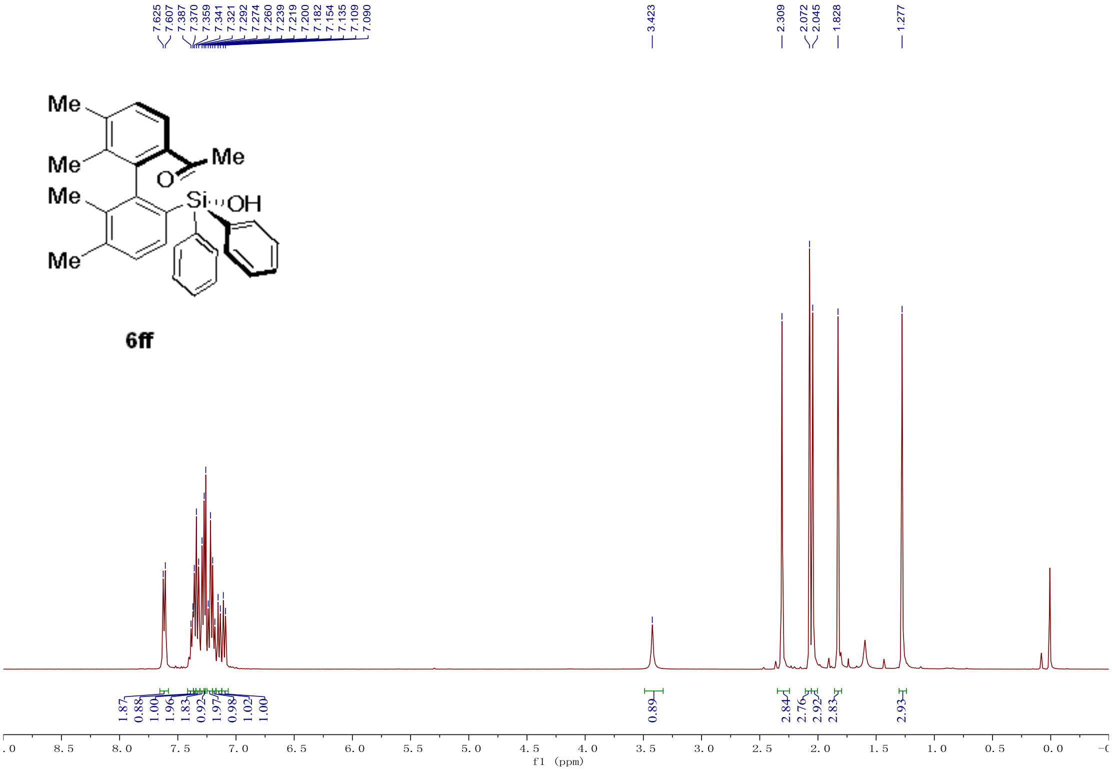

Supplementary Figure 124. ^{1}**H NMR** spectroscopy of **6ff**

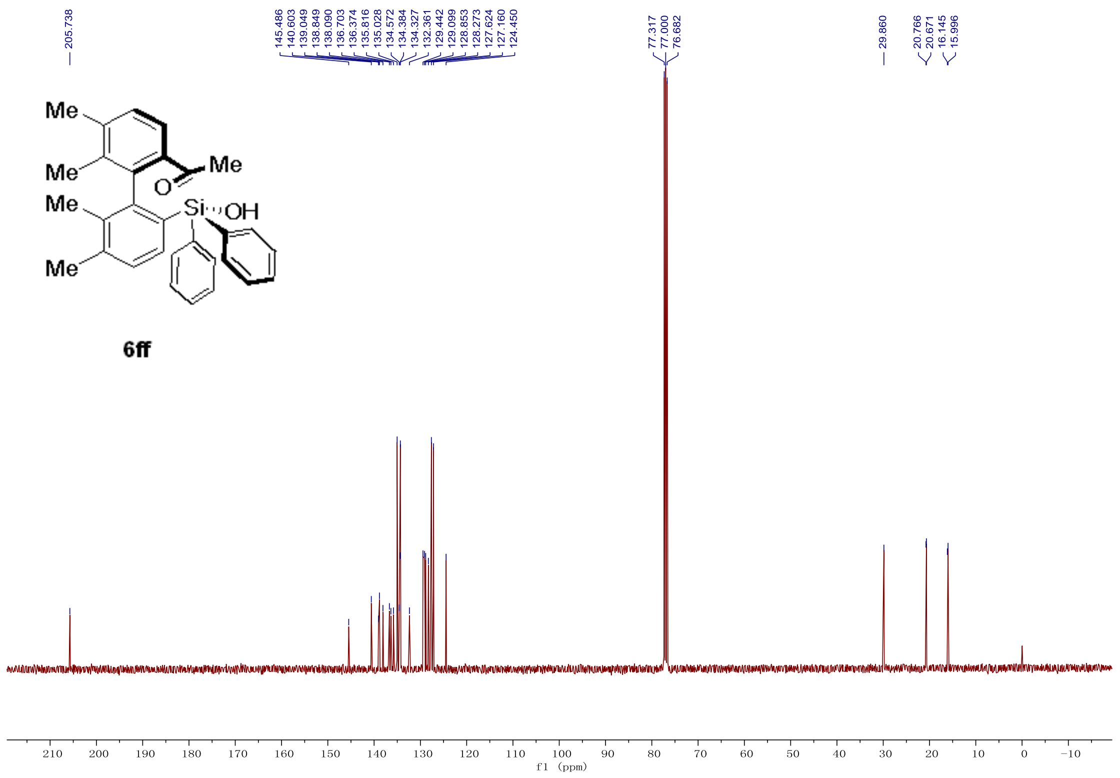

Supplementary Figure 125. ^{13}**C NMR** spectroscopy of **6ff**

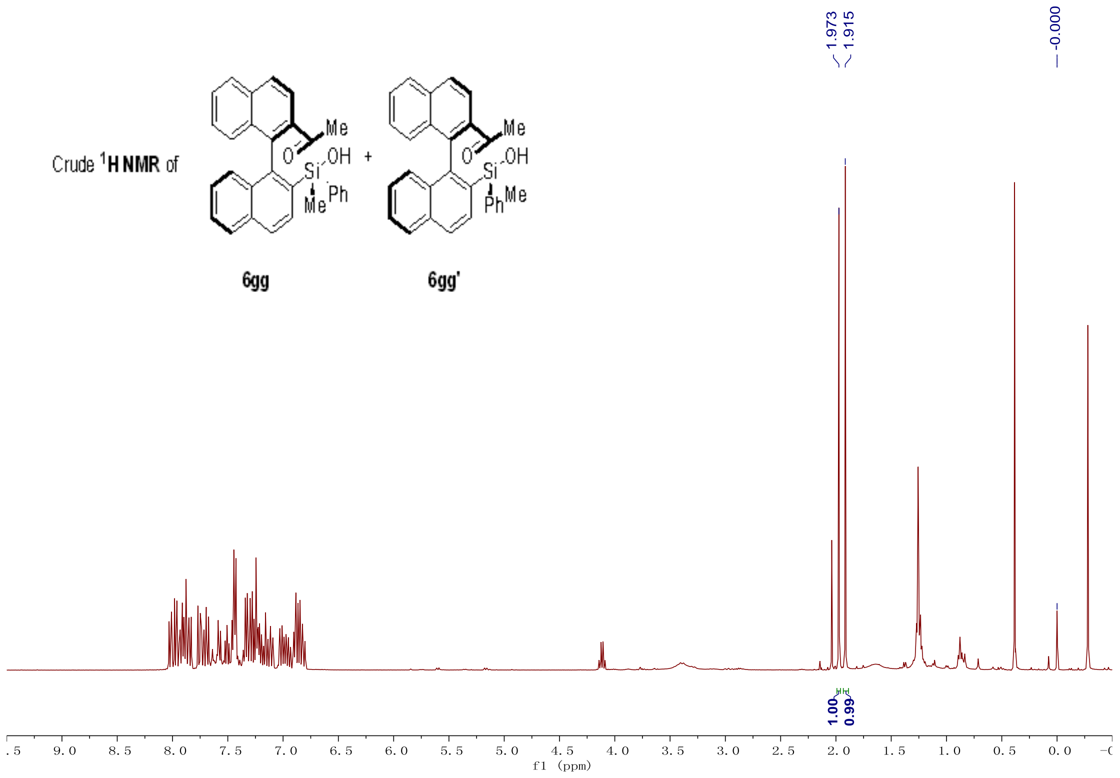

Supplementary Figure 126. Crude ^{1}**H NMR** spectroscopy of **6gg** and **6gg'**

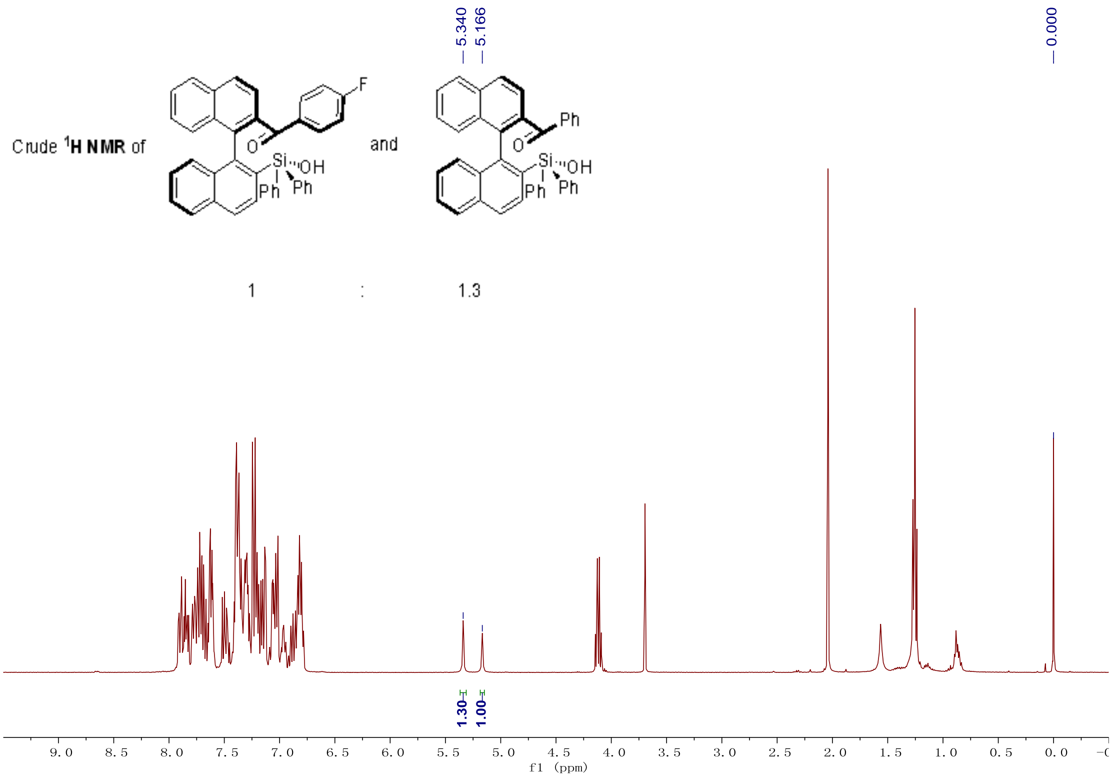

Supplementary Figure 127. Crude ^{1}**H NMR** spectroscopy of **6m** and **6b**

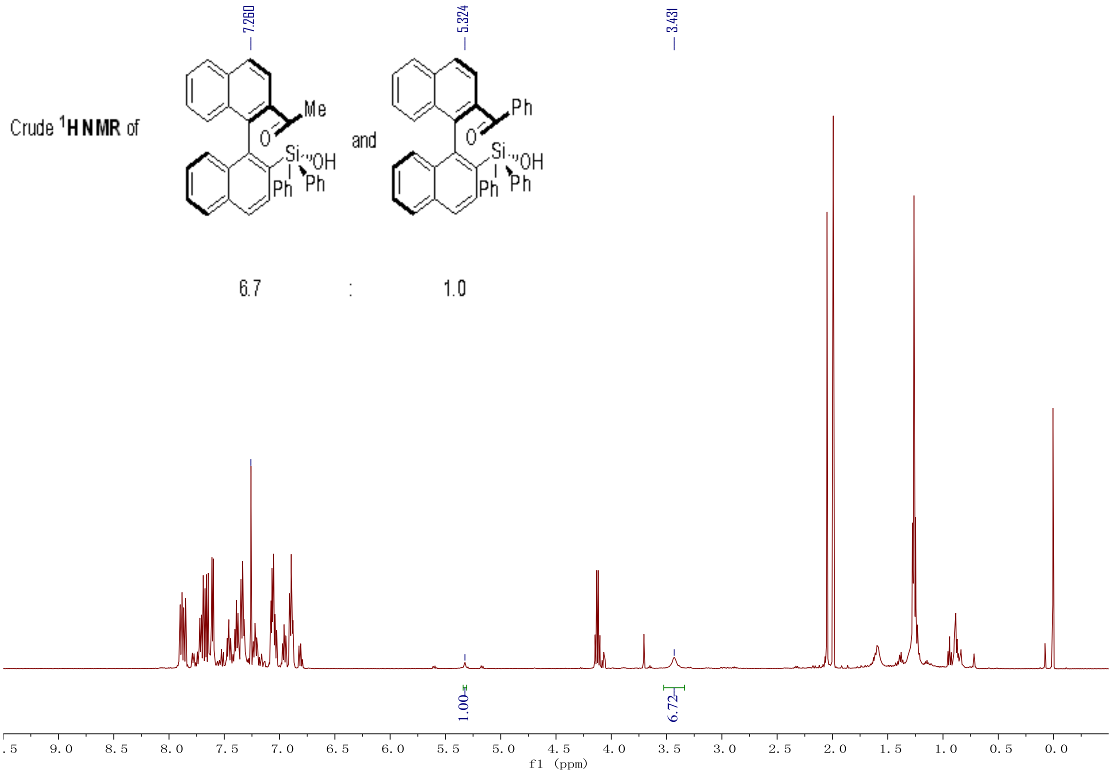

Supplementary Figure 128. Crude ^{1}**H NMR** spectroscopy of **6c** and **6b**

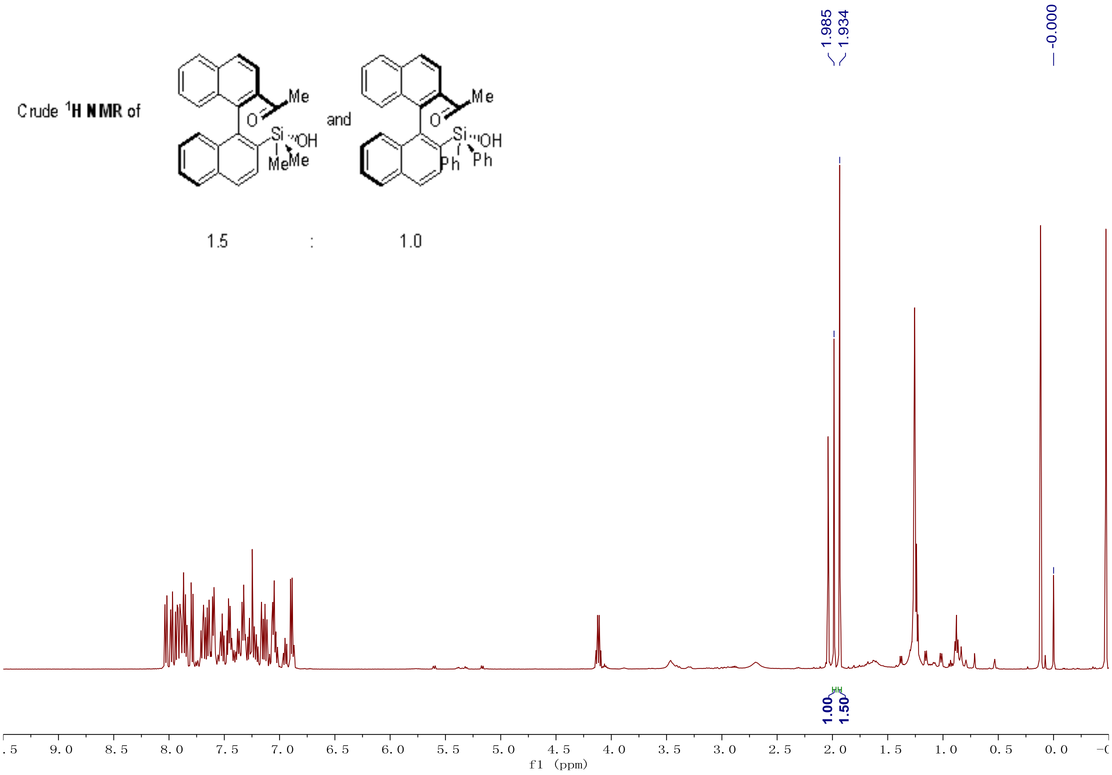

Supplementary Figure 129. Crude ^{1}**H NMR** spectroscopy of **6a** and **6c**

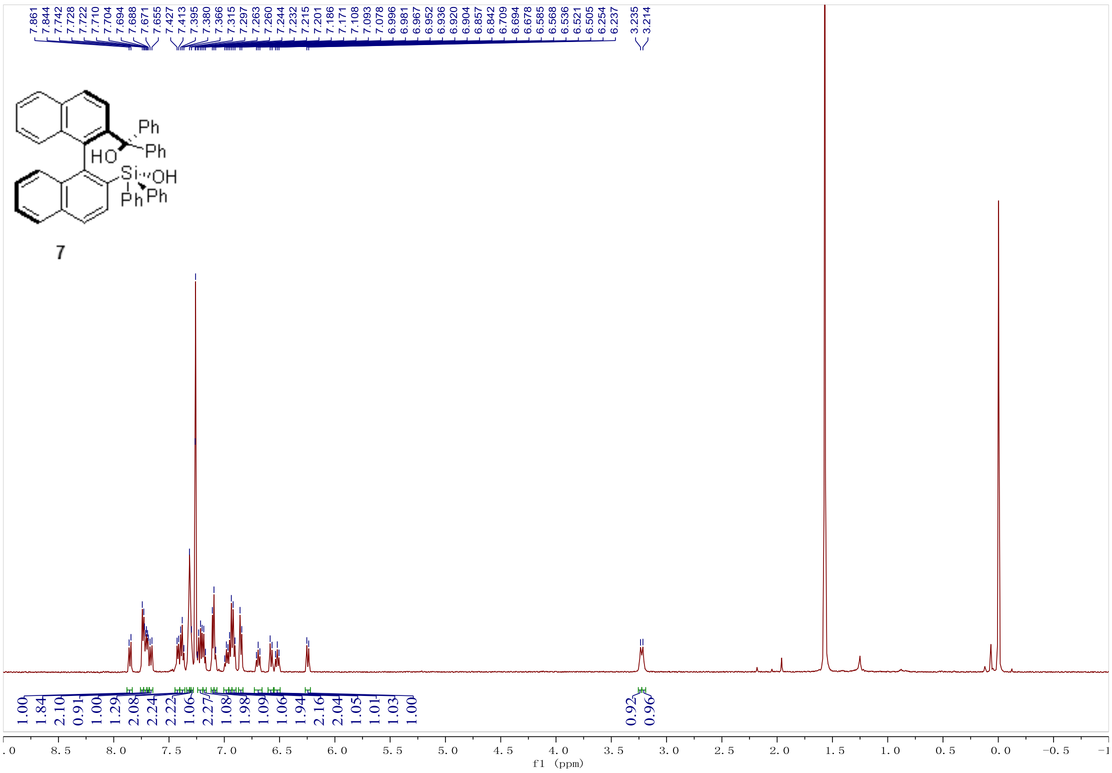

Supplementary Figure 130. ^{1}**H NMR** spectroscopy of **7**

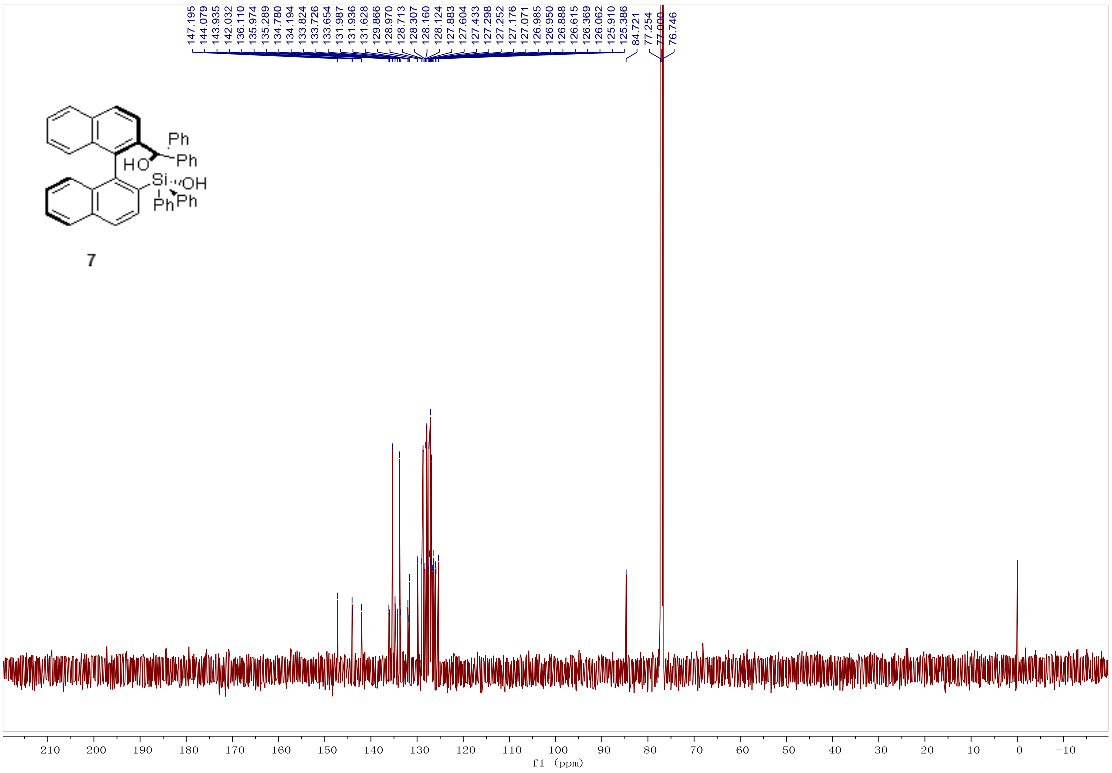

Supplementary Figure 131. ^{13}**C NMR** spectroscopy of **7**

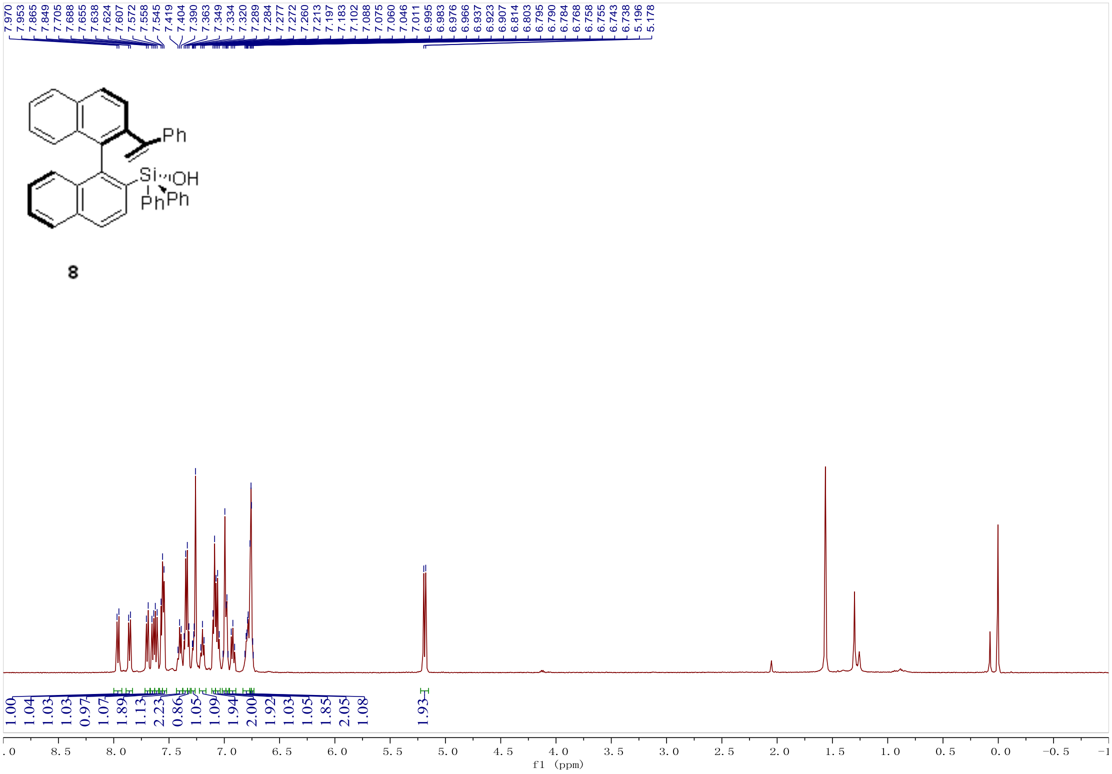

Supplementary Figure 132. ^{1}**H NMR** spectroscopy of **8**

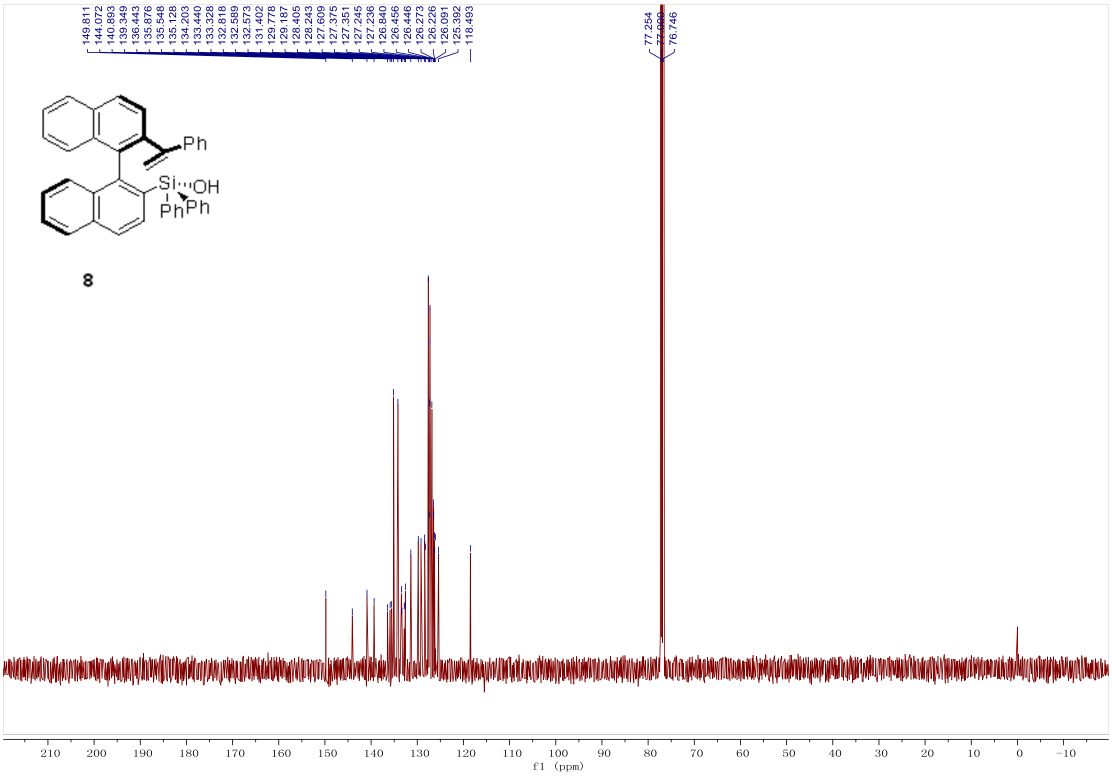

Supplementary Figure 133. ^{13}**C NMR** spectroscopy of **8**

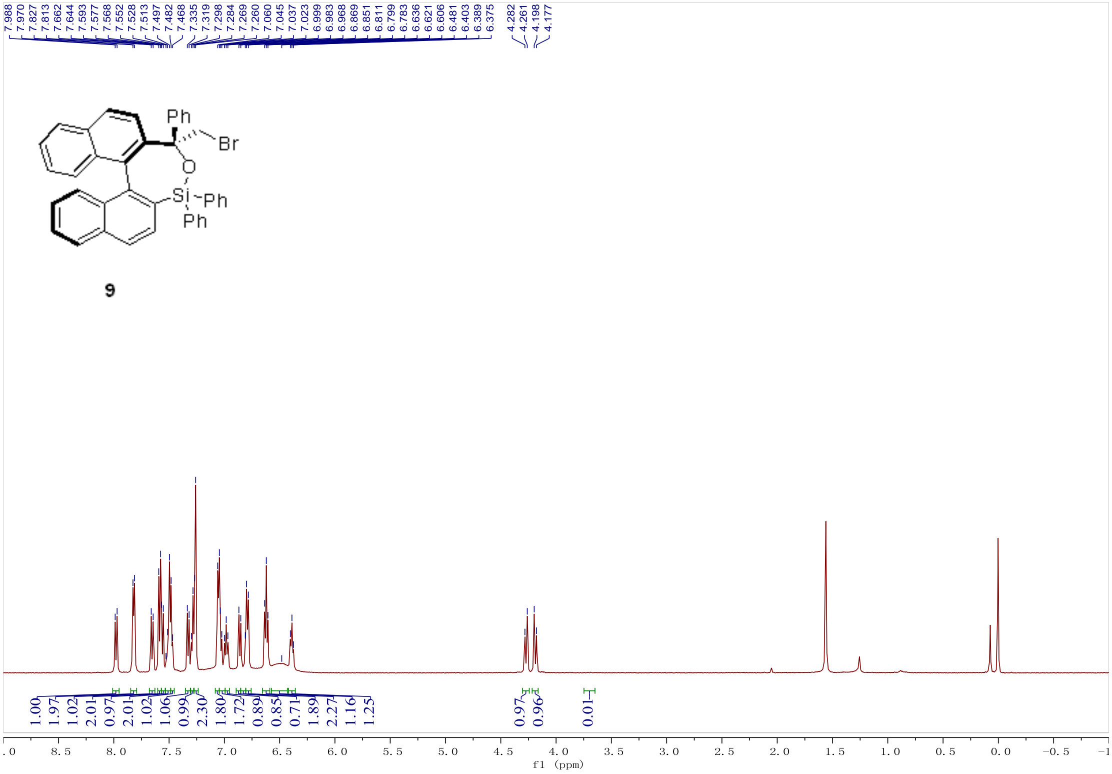

Supplementary Figure 134. ^{1}**H NMR** spectroscopy of **9**

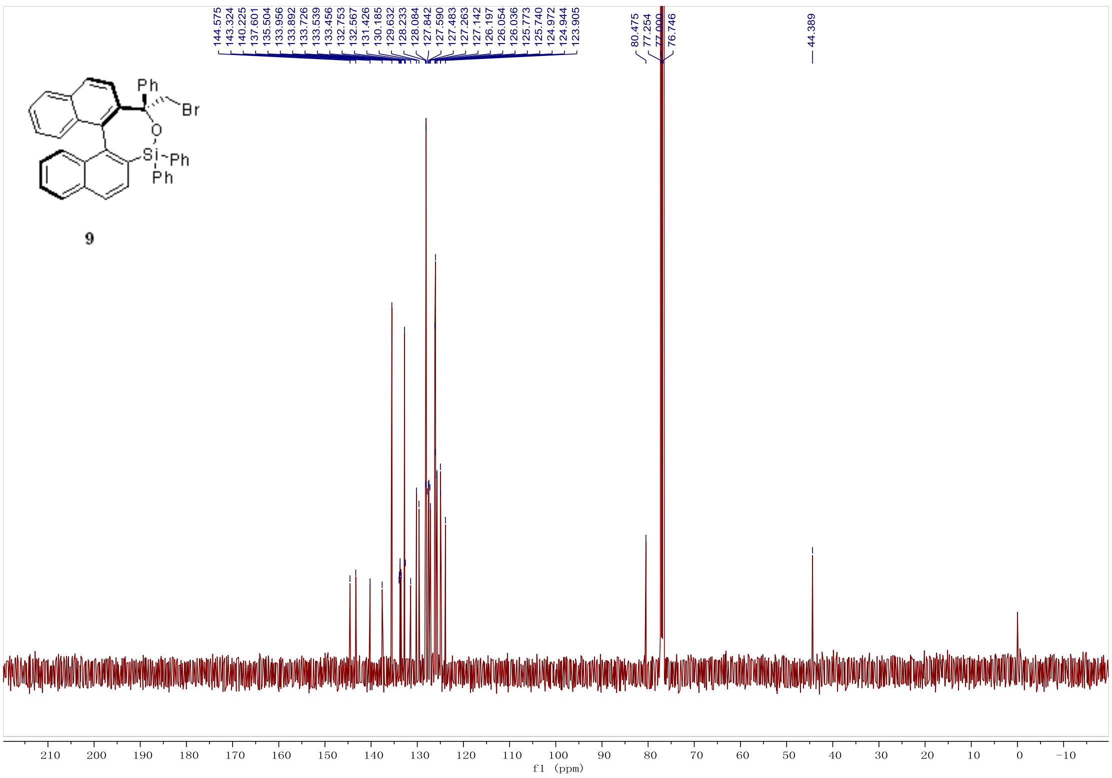

Supplementary Figure 135. ^{13}**C NMR** spectroscopy of **9**

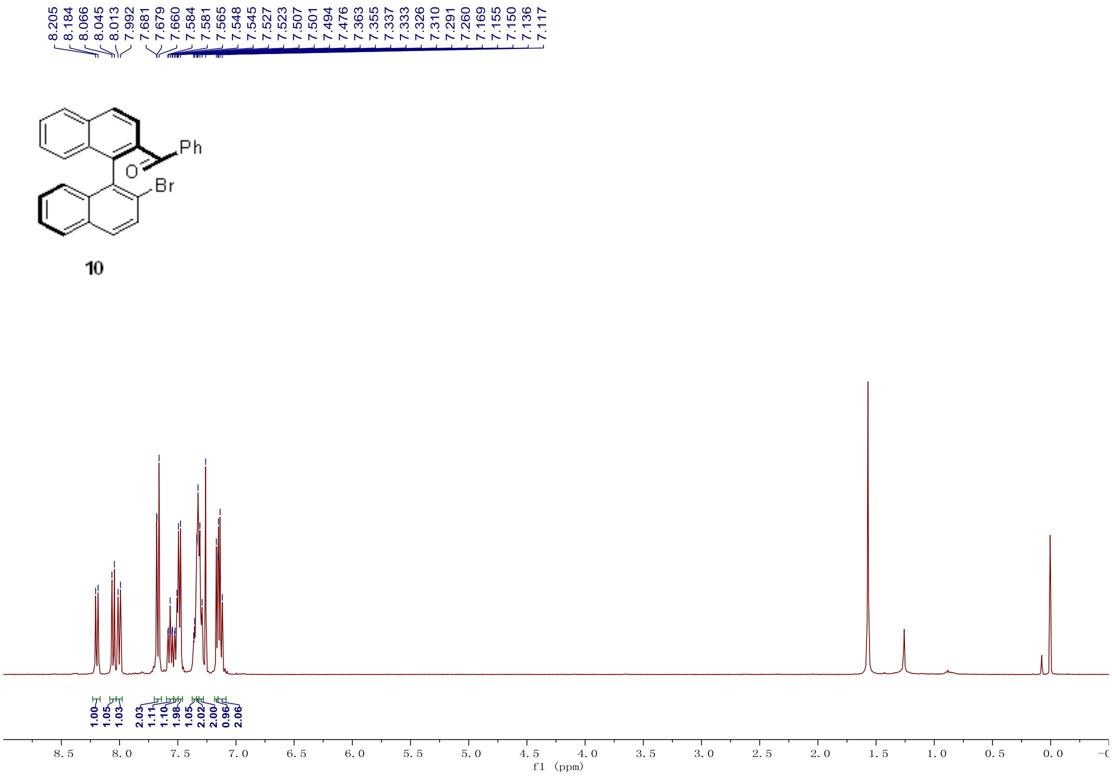

Supplementary Figure 136. ^{1}**H NMR** spectroscopy of **10**

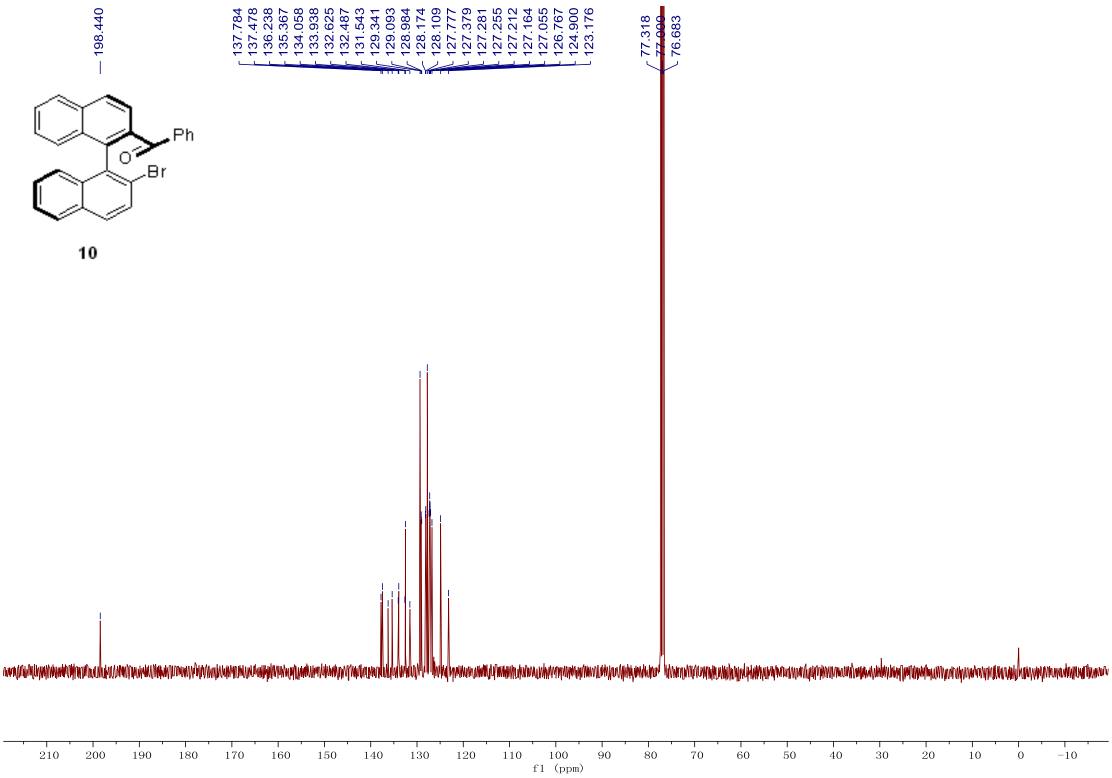

Supplementary Figure 137. ^{13}**C NMR** spectroscopy of **10**

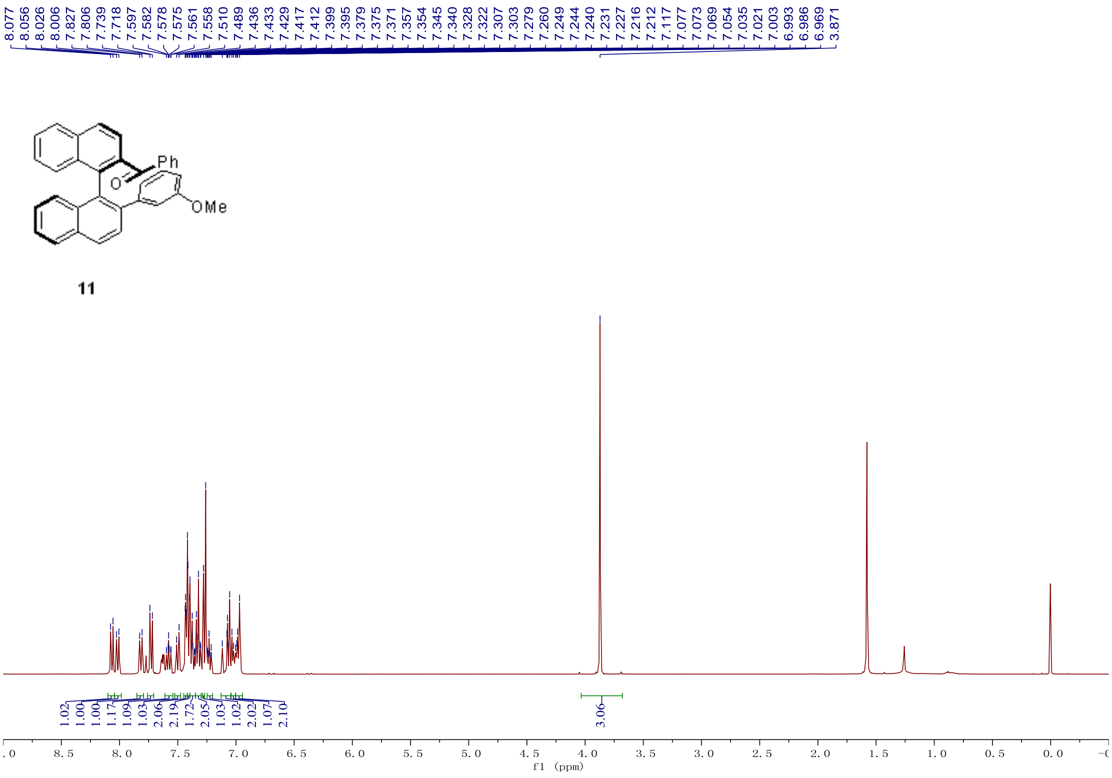

Supplementary Figure 138. ^{1}**H NMR** spectroscopy of **11**

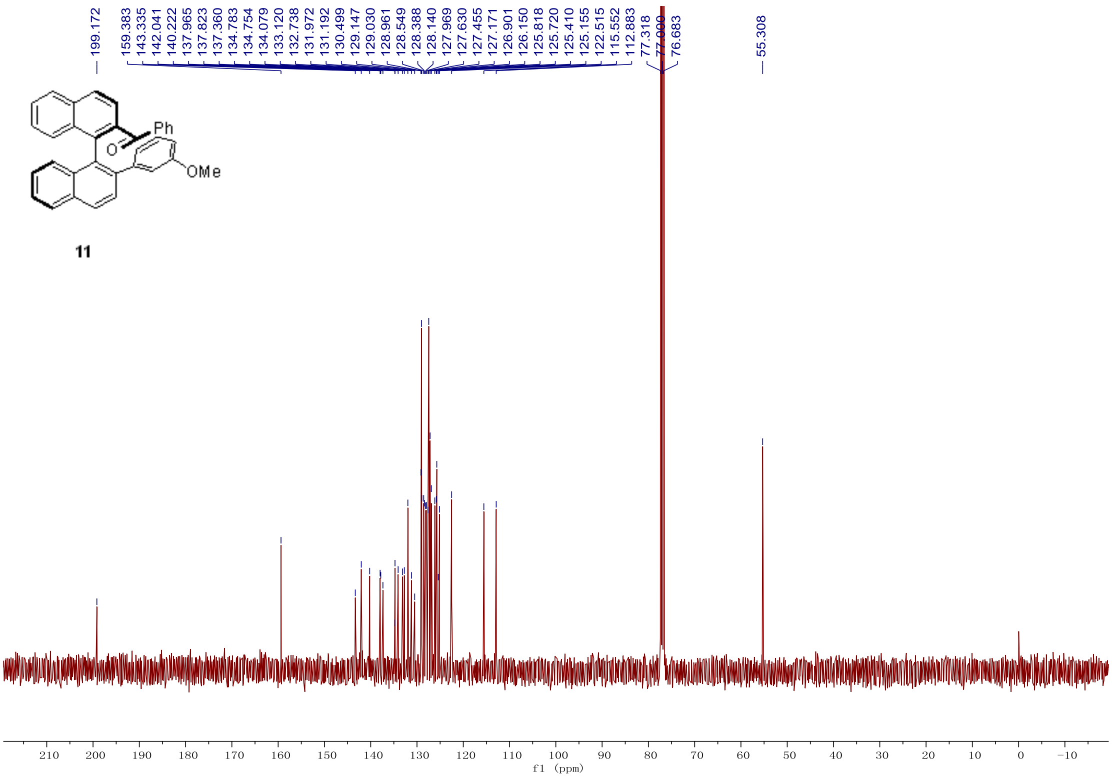

Supplementary Figure 139. ^{13}**C NMR** spectroscopy of **11**

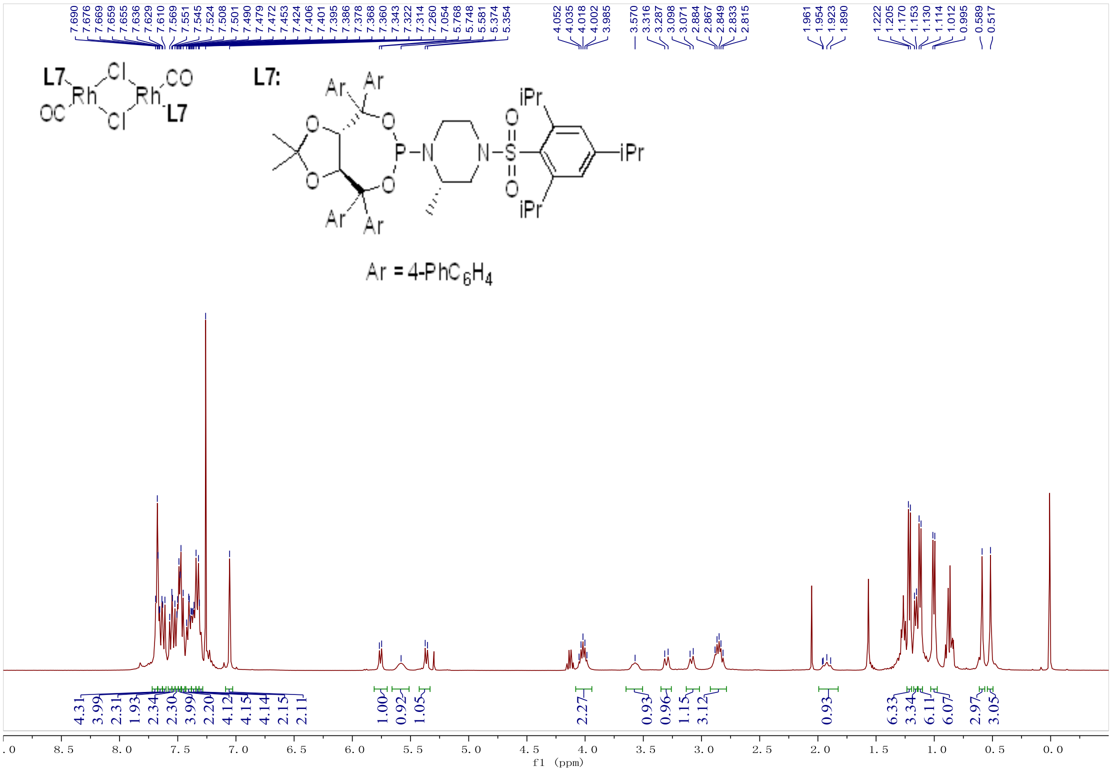

Supplementary Figure 140. ^{1}**H NMR** spectroscopy of **Rh-1**

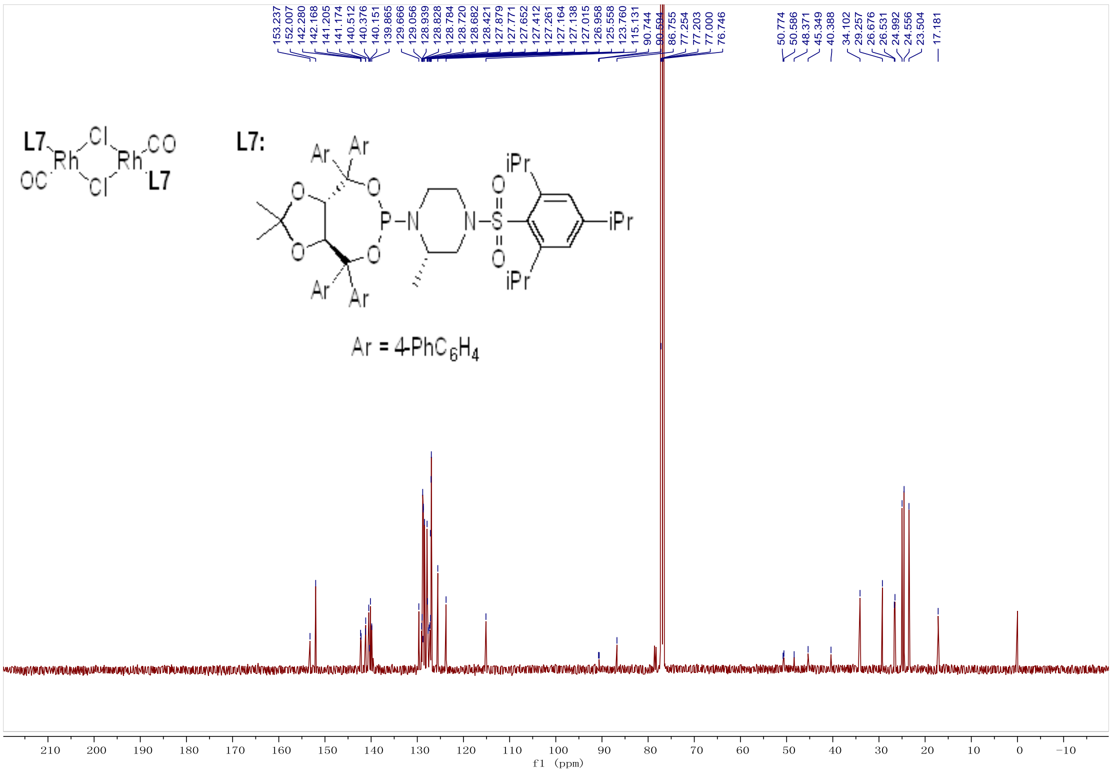

Supplementary Figure 141. ^{13}**C NMR** spectroscopy of **Rh-1**

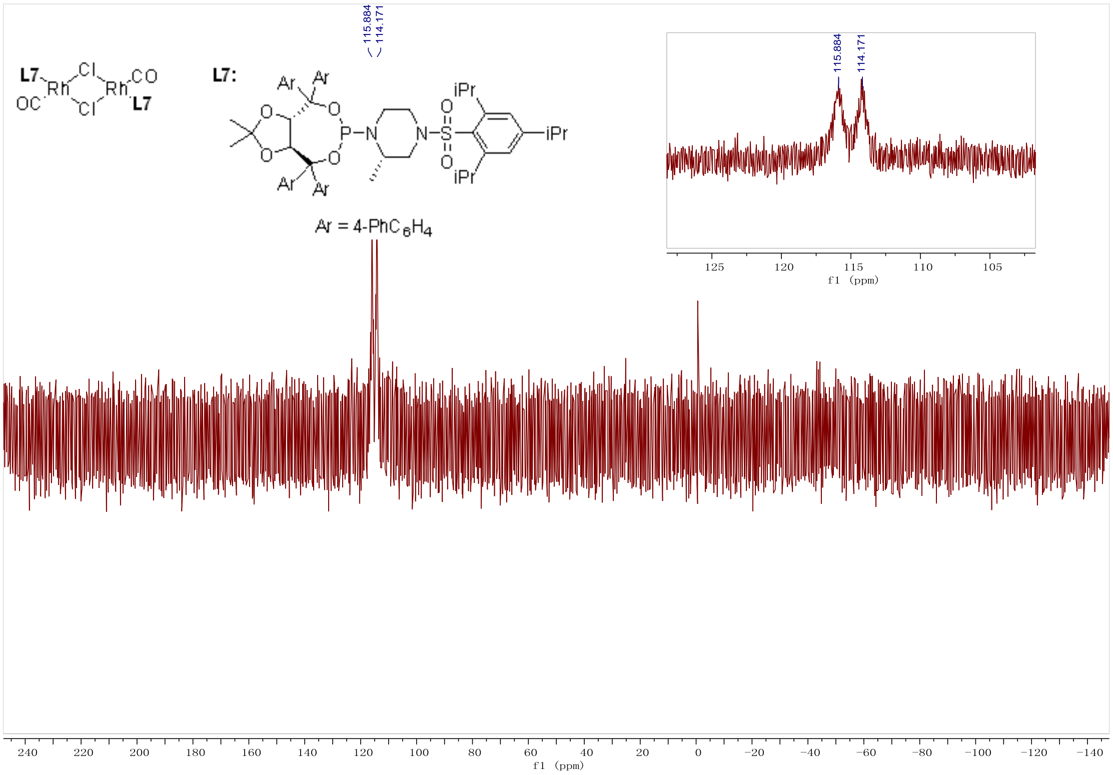

Supplementary Figure 142. ^{31}**P NMR** spectroscopy of **Rh-1**

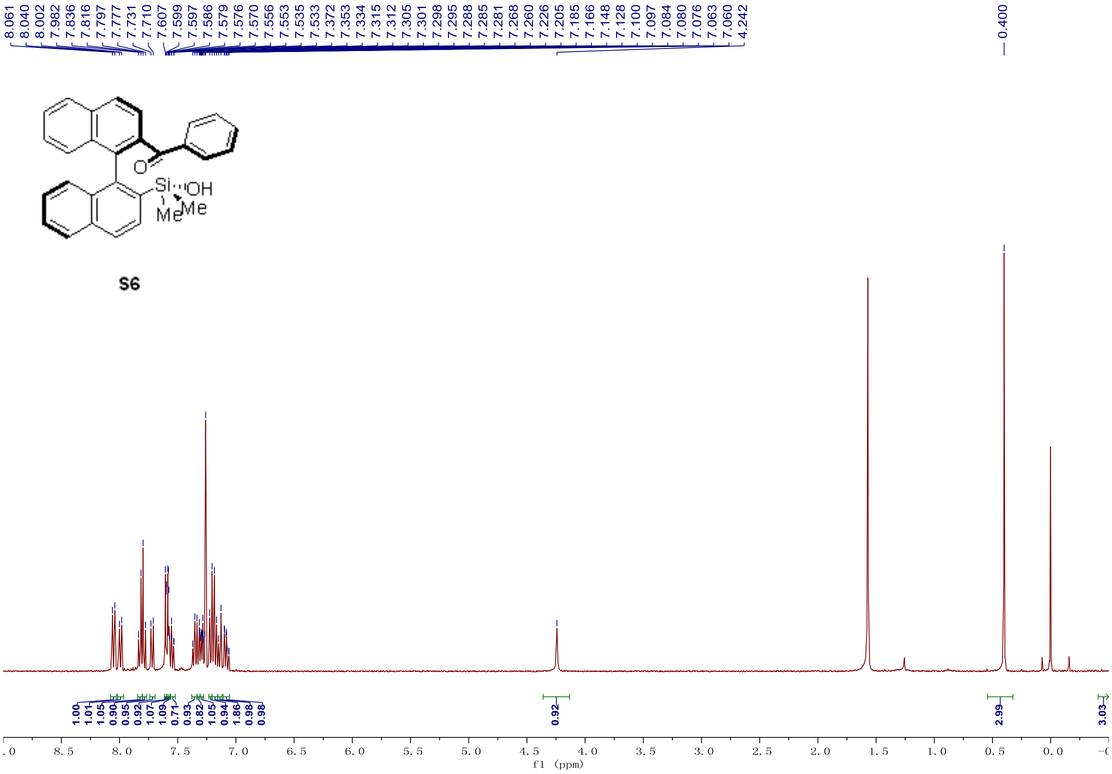

Supplementary Figure 143. ^{1}**H NMR** spectroscopy of **S6**

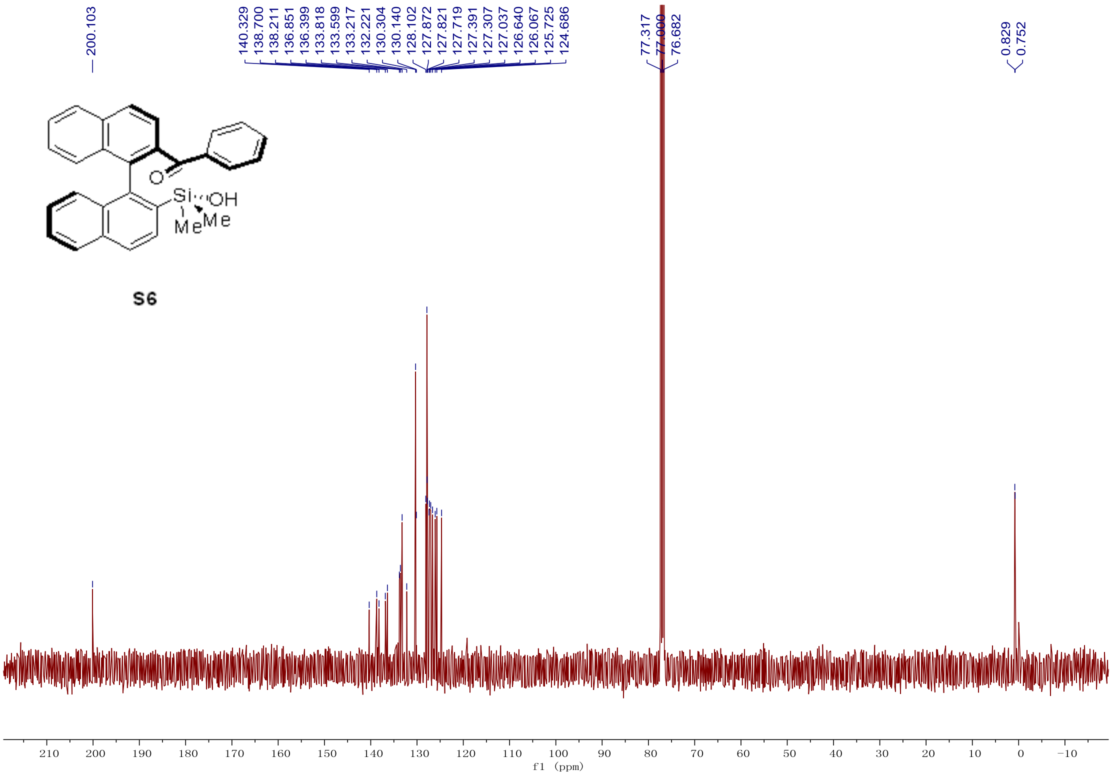

Supplementary Figure 144. ^{13}**C NMR** spectroscopy of **S6**

Copies of HPLC traces

Data File F:\DATA\JFENG\DATA\BXF-2-34-rac(AD-H,10%,1.0).D
Sample Name: BXF-2-34-rac(AD-H,10%,1.0)

```
=====================================================================
Acq. Operator   :
Sample Operator :
Acq. Instrument : LC1260                          Location :   1
Injection Date  : 09/09/2019 21:20:27
                                               Inj Volume : No inj
Acq. Method     : C:\Chem32\1\Methods\DEF_LC.M
Last changed    : 09/09/2019 21:09:32 by
                  (modified after loading)
Analysis Method : F:\METHOD\ZK.M
Last changed    : 14/07/2019 15:34:15 by
Additional Info : Peak(s) manually integrated
```

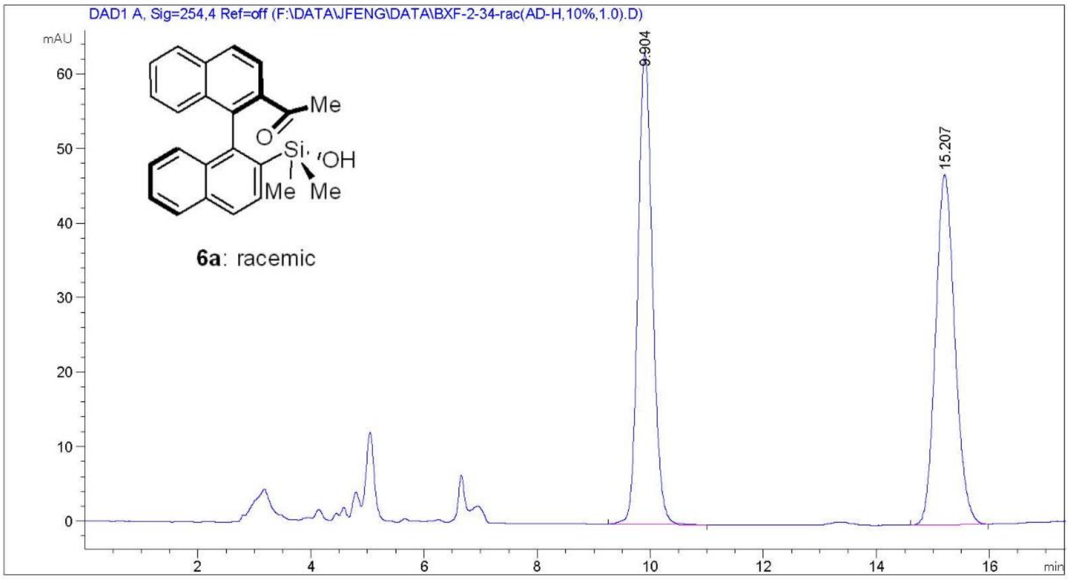


```
=====================================================================
                        Area Percent Report
=====================================================================

Sorted By             :      Signal
Multiplier            :      1.0000
Dilution              :      1.0000
Use Multiplier & Dilution Factor with ISTDs


Signal 1: DAD1 A, Sig=254,4 Ref=off
```

Peak #	RetTime [min]	Type	Width [min]	Area [mAU*s]	Height [mAU]	Area %
1	9.904	BB	0.2761	1127.30164	63.20520	50.3058
2	15.207	BB	0.3646	1113.59607	46.97394	49.6942

Totals : 2240.89771 110.17914

LC1260 30/11/2019 09:43:56 Page 1 of 2

Supplementary Figure 145. HPLC data of **6a**

```
Data File F:\DATA\JFENG\DATA\BXF-2-31-repeat-2(AD,10%,1.0).D
Sample Name: BXF-2-31-repeat-2(AD,10%,1.0)

=====================================================================
Acq. Operator   :
Sample Operator :
Acq. Instrument : LC1260                          Location :   1
Injection Date  : 16/09/2019 21:12:50
                                                Inj Volume : No inj
Acq. Method     : C:\Chem32\1\Methods\DEF_LC.M
Last changed    : 16/09/2019 21:11:18 by
                  (modified after loading)
Analysis Method : F:\METHOD\ZK.M
Last changed    : 14/07/2019 15:34:15 by
Additional Info : Peak(s) manually integrated
```

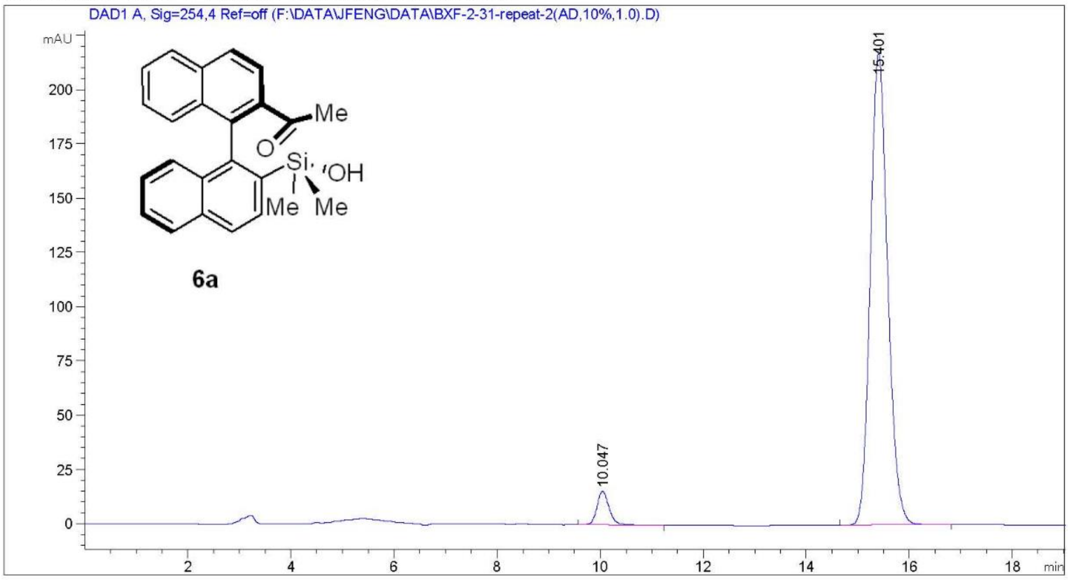


```
=====================================================================
                         Area Percent Report
=====================================================================

Sorted By             :      Signal
Multiplier            :      1.0000
Dilution              :      1.0000
Use Multiplier & Dilution Factor with ISTDs


Signal 1: DAD1 A, Sig=254,4 Ref=off

Peak RetTime Type  Width     Area      Height     Area
  #   [min]        [min]   [mAU*s]     [mAU]       %
----|-------|----|-------|----------|----------|--------|
   1  10.047 BB    0.2489  254.15904   15.53496   4.7712
   2  15.401 BB    0.3628 5072.73682  216.96078  95.2288

Totals :                  5326.89586  232.49575
```


Supplementary Figure 146. HPLC data of **6a**

Data File F:\DATA\JFENG\DATA\LN-1-3(AD,20%,1.0).D
Sample Name: LN-1-3(AD,20%,1.0)

===

Acq. Operator :
Sample Operator :
Acq. Instrument : LC1260 Location : 1
Injection Date : 11/09/2019 09:56:26
Inj Volume : Manually
Acq. Method : F:\METHOD\JFeng.M
Last changed : 11/09/2019 09:48:46 by
(modified after loading)
Analysis Method : F:\METHOD\ZK.M
Last changed : 14/07/2019 15:34:15 by
Additional Info : Peak(s) manually integrated

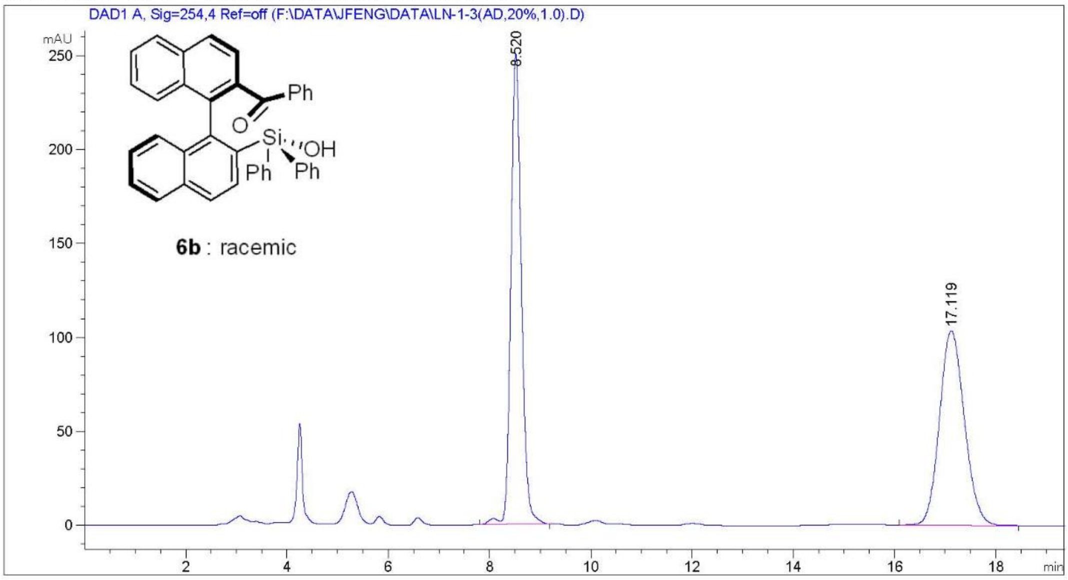

===

Area Percent Report

===

Sorted By : Signal
Multiplier : 1.0000
Dilution : 1.0000
Use Multiplier & Dilution Factor with ISTDs

Signal 1: DAD1 A, Sig=254,4 Ref=off

Peak #	RetTime [min]	Type	Width [min]	Area [mAU*s]	Height [mAU]	Area %
1	8.520	VB R	0.2198	3598.10596	250.34334	50.4370
2	17.119	BB	0.5306	3535.75171	103.48499	49.5630

Totals : 7133.85767 353.82833

LC1260 30/11/2019 10:48:02 Page 1 of 2

Supplementary Figure 147. HPLC data of **6b**

Data File F:\DATA\JFENG\DATA\BXF-2-30-(2)-0911(AD,20%,1.0).D
Sample Name: BXF-2-30-(2)-0911(AD,20%,1.0)

===

Acq. Operator :

Location : 1

Injection Date : 11/09/2019 10:19:20
Acq. Method : JFeng.M
Analysis Method : F:\METHOD\ZK.M
Last changed : 14/07/2019 15:34:15 by
Additional Info : Peak(s) manually integrated

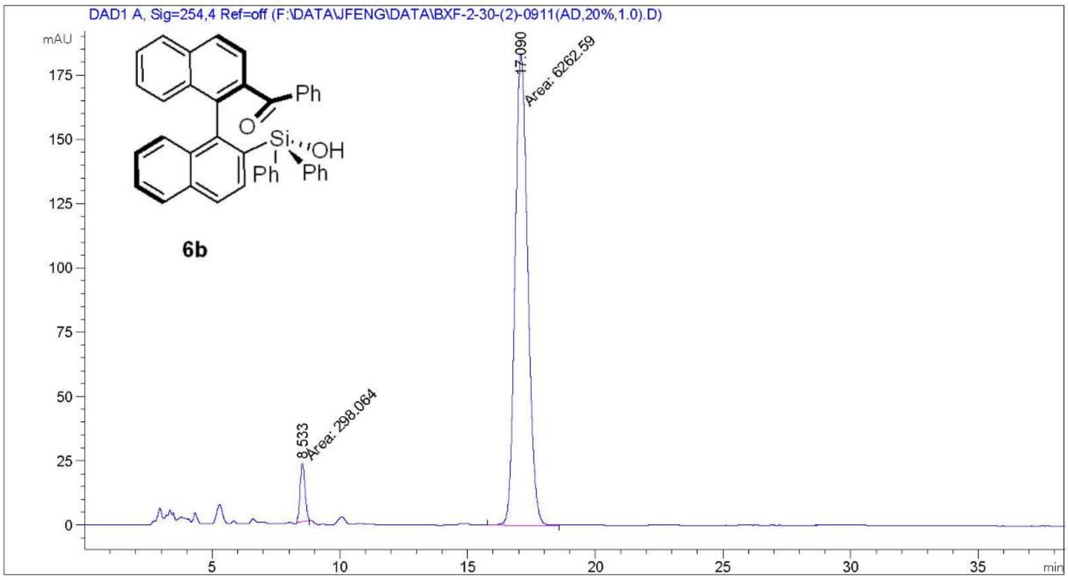

===

Area Percent Report

===

Sorted By : Signal
Multiplier : 1.0000
Dilution : 1.0000
Use Multiplier & Dilution Factor with ISTDs

Signal 1: DAD1 A, Sig=254,4 Ref=off

Peak #	RetTime [min]	Type	Width [min]	Area [mAU*s]	Height [mAU]	Area %
1	8.533	MM	0.2184	298.06430	22.74938	4.5432
2	17.090	MM	0.5704	6262.58691	183.00049	95.4568

Totals : 6560.65121 205.74987

===

*** End of Report ***

LC1260 30/11/2019 10:50:23 Page 1 of 1

Supplementary Figure 148. HPLC data of **6b**

```
Data File F:\DATA\JFENG\DATA\BXF-2-73(AD,15%,1.0).D
Sample Name: BXF-2-73(AD,15%,1.0)

=====================================================================
Acq. Operator   :
Sample Operator :
Acq. Instrument : LC1260                          Location :   1
Injection Date  : 06/10/2019 20:03:26
                                              Inj Volume : No inj
Acq. Method     : C:\Chem32\1\Methods\DEF_LC.M
Last changed    : 06/10/2019 19:53:44 by
                  (modified after loading)
Analysis Method : F:\METHOD\ZK.M
Last changed    : 14/07/2019 15:34:15 by
Additional Info : Peak(s) manually integrated
```

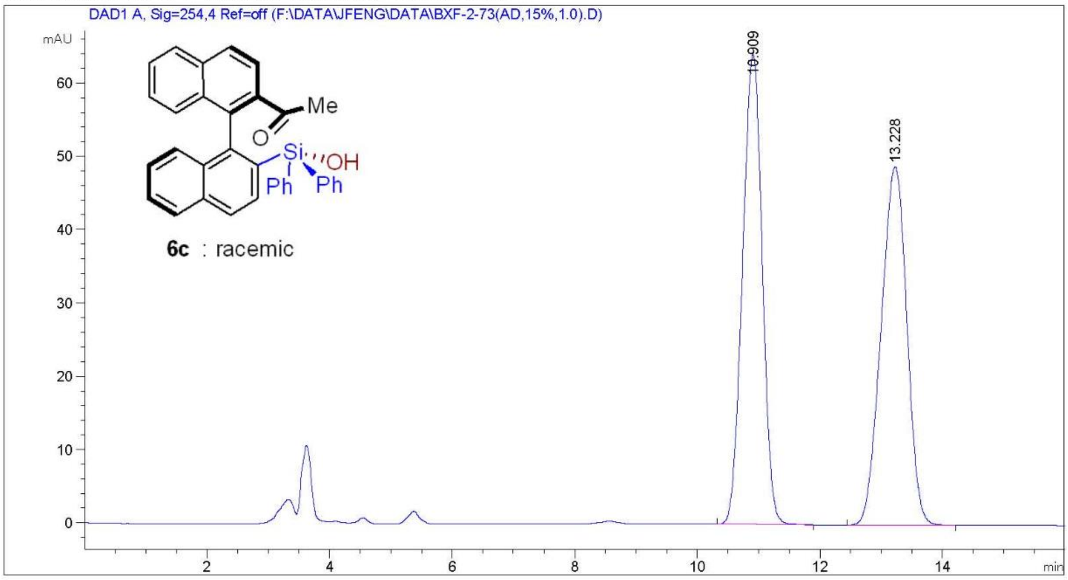


```
=====================================================================
                          Area Percent Report
=====================================================================

Sorted By             :      Signal
Multiplier            :      1.0000
Dilution              :      1.0000
Use Multiplier & Dilution Factor with ISTDs


Signal 1: DAD1 A, Sig=254,4 Ref=off

Peak RetTime Type  Width     Area      Height     Area
  #   [min]        [min]   [mAU*s]     [mAU]        %
----|-------|----|-------|----------|----------|--------|
   1  10.909 BB    0.3515 1426.47388   64.12357  50.0319
   2  13.228 BB    0.4561 1424.65601   48.86647  49.9681

Totals :                  2851.12988  112.99004
```


Supplementary Figure 149. HPLC data of **6c**

Data File F:\DATA\JFENG\DATA\BXF-2-71(AD,15%,1.0).D
Sample Name: BXF-2-71(AD,15%,1.0)

===
Acq. Operator   :
Location :   1
Injection Date : 06/10/2019 20:30:50
Acq. Method   : DEF_LC.M
Analysis Method : F:\METHOD\ZK.M
Last changed   : 14/07/2019 15:34:15 by
Additional Info : Peak(s) manually integrated

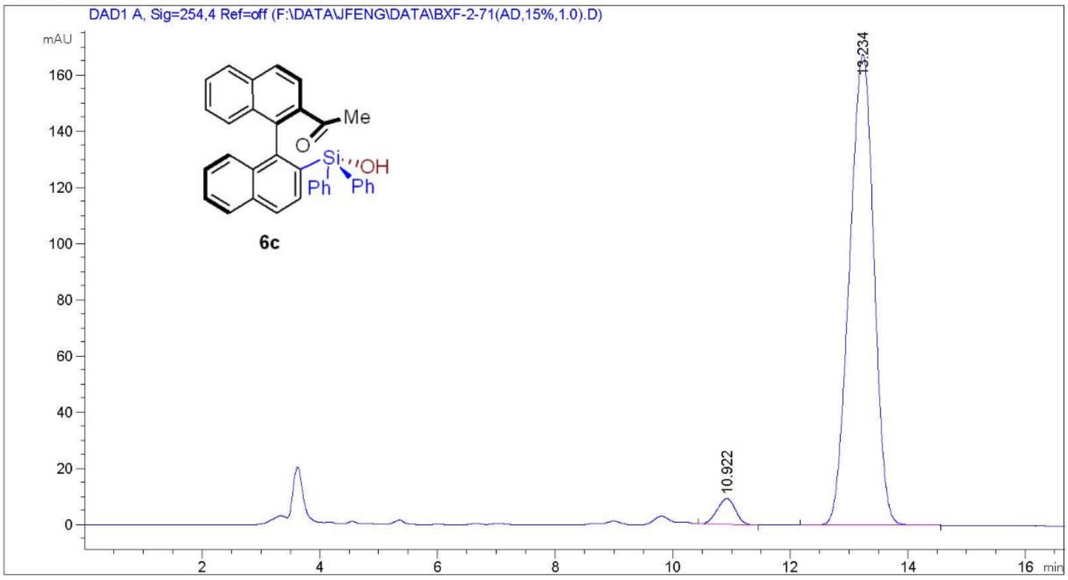

===
Area Percent Report
===

Sorted By : Signal
Multiplier : 1.0000
Dilution : 1.0000
Use Multiplier & Dilution Factor with ISTDs

Signal 1: DAD1 A, Sig=254,4 Ref=off

Peak #	RetTime [min]	Type	Width [min]	Area [mAU*s]	Height [mAU]	Area %
1	10.922	BB	0.3409	199.83362	9.21795	3.9765
2	13.234	BB	0.4523	4825.53223	167.40480	96.0235

Totals : 5025.36584 176.62275

===
*** End of Report ***

LC1260 30/11/2019 10:06:05 Page 1 of 1

Supplementary Figure 150. HPLC data of **6c**

Data File F:\DATA\JFENG\DATA\BXF-2-74-2(AD,5%,1.0).D
Sample Name: BXF-2-74-2(AD,5%,1.0)

===

Acq. Operator :
Sample Operator :
Acq. Instrument : LC1260 Location : 1
Injection Date : 10/10/2019 19:57:58
Inj Volume : No inj
Acq. Method : C:\Chem32\1\Methods\DEF_LC.M
Last changed : 10/10/2019 19:49:52 by
(modified after loading)
Analysis Method : F:\METHOD\ZK.M
Last changed : 14/07/2019 15:34:15 by
Additional Info : Peak(s) manually integrated

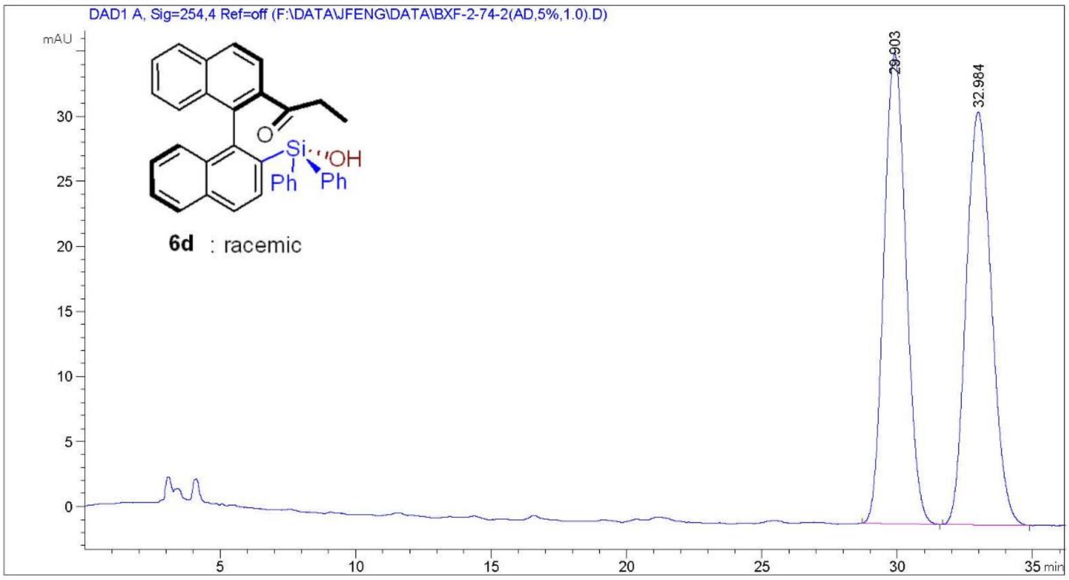

===

Area Percent Report

===

Sorted By : Signal
Multiplier : 1.0000
Dilution : 1.0000
Use Multiplier & Dilution Factor with ISTDs

Signal 1: DAD1 A, Sig=254,4 Ref=off

Peak #	RetTime [min]	Type	Width [min]	Area [mAU*s]	Height [mAU]	Area %
1	29.903	BB	0.8909	2052.03784	36.11281	49.9056
2	32.984	BB	0.9987	2059.80151	31.75861	50.0944

Totals : 4111.83936 67.87142

LC1260 30/11/2019 10:02:21 Page 1 of 2

Supplementary Figure 151. HPLC data of **6d**

Data File F:\DATA\JFENG\DATA\BXF-2-81-1(AD,5%,1.0).D
Sample Name: BXF-2-81-1(AD,5%,1.0)

===

Acq. Operator :

Location : 1

Injection Date : 09/10/2019 16:30:49
Acq. Method : DEF_LC.M
Analysis Method : F:\METHOD\ZK.M
Last changed : 14/07/2019 15:34:15 by
Additional Info : Peak(s) manually integrated

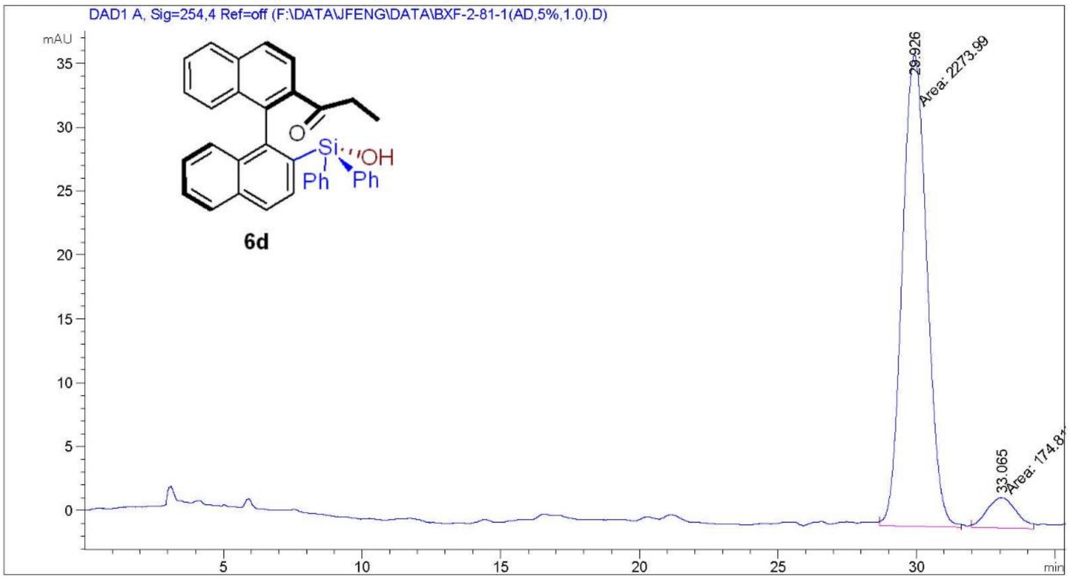

===

Area Percent Report

===

Sorted By : Signal
Multiplier : 1.0000
Dilution : 1.0000
Use Multiplier & Dilution Factor with ISTDs

Signal 1: DAD1 A, Sig=254,4 Ref=off

Peak #	RetTime [min]	Type	Width [min]	Area [mAU*s]	Height [mAU]	Area %
1	29.926	MM	1.0270	2273.98682	36.90383	92.8611
2	33.065	MM	1.2275	174.81848	2.37354	7.1389

Totals : 2448.80530 39.27737

===

*** End of Report ***

LC1260 30/11/2019 10:03:50 Page 1 of 1

Supplementary Figure 152. HPLC data of **6d**

Data File F:\DATA\JFENG\DATA\FJ-11-17-rac(AD,10%,1.0).D
Sample Name: FJ-11-17-rac(AD,10%,1.0)

===

Acq. Operator :
Sample Operator :
Acq. Instrument : LC1260 Location : 1
Injection Date : 11/10/2019 15:51:57
Inj Volume : Manually
Acq. Method : F:\METHOD\JFeng.M
Last changed : 11/10/2019 15:42:49 by
(modified after loading)
Analysis Method : F:\METHOD\ZK.M
Last changed : 14/07/2019 15:34:15 by
Additional Info : Peak(s) manually integrated

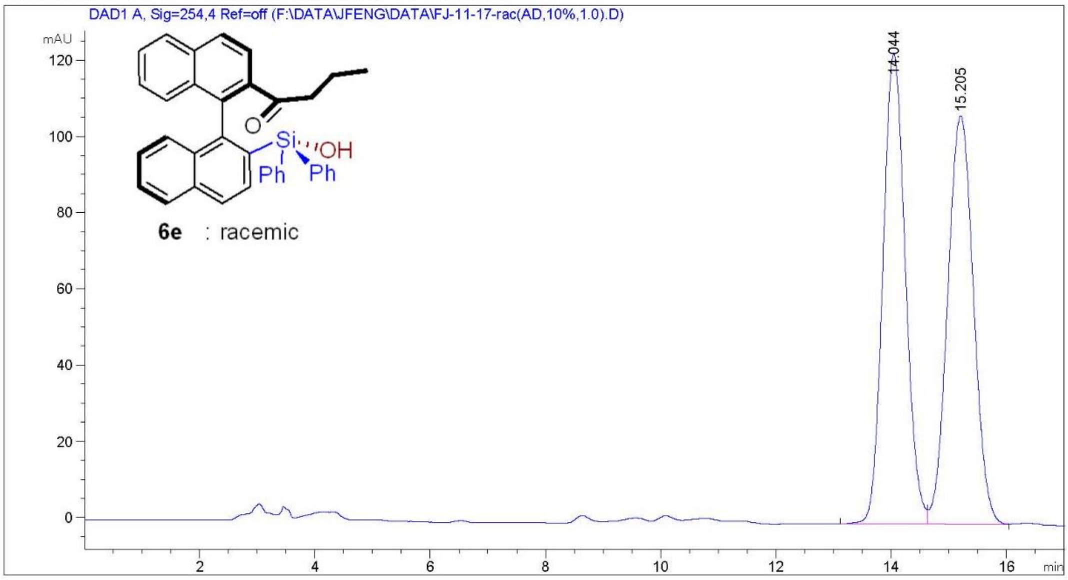

===

Area Percent Report

===

Sorted By : Signal
Multiplier : 1.0000
Dilution : 1.0000
Use Multiplier & Dilution Factor with ISTDs

Signal 1: DAD1 A, Sig=254,4 Ref=off

Peak #	RetTime [min]	Type	Width [min]	Area [mAU*s]	Height [mAU]	Area %
1	14.044	BV	0.4236	3334.27148	123.24934	50.0597
2	15.205	VB	0.4903	3326.31982	107.10140	49.9403

Totals : 6660.59131 230.35074

LC1260 30/11/2019 15:05:07 Page 1 of 2

Supplementary Figure 153. HPLC data of **6e**

Data File F:\DATA\JFENG\DATA\FJ-11-16(AD,10%,1.0).D
Sample Name: FJ-11-16(AD,10%,1.0)

===

Acq. Operator :
Location : 1
Injection Date : 11/10/2019 16:11:50
Acq. Method : JFeng.M
Analysis Method : F:\METHOD\ZK.M
Last changed : 14/07/2019 15:34:15 by
Additional Info : Peak(s) manually integrated

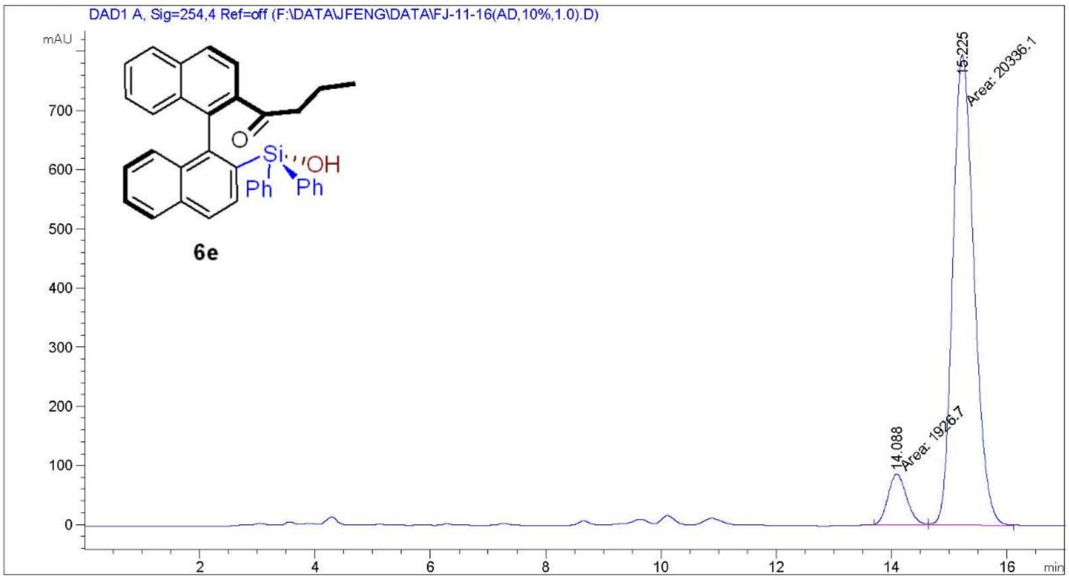

===

Area Percent Report

===

Sorted By : Signal
Multiplier : 1.0000
Dilution : 1.0000
Use Multiplier & Dilution Factor with ISTDs

Signal 1: DAD1 A, Sig=254,4 Ref=off

Peak #	RetTime [min]	Type	Width [min]	Area [mAU*s]	Height [mAU]	Area %
1	14.088	MM	0.3750	1926.70337	85.64039	8.6543
2	15.225	MM	0.4260	2.03361e4	795.60052	91.3457

Totals : 2.22628e4 881.24091

===

*** End of Report ***

LC1260 30/11/2019 15:07:20 Page 1 of 1

Supplementary Figure 154. HPLC data of **6e**

```
Data File F:\DATA\JFENG\DATA\FJ-11-19-rac(AD,10%,1.0).D
Sample Name: FJ-11-19-rac(AD,10%,1.0)

    =====================================================================
    Acq. Operator   :
    Sample Operator :
    Acq. Instrument : LC1260                          Location :   1
    Injection Date  : 11/10/2019 16:31:48
                                                    Inj Volume : Manually
    Acq. Method     : F:\METHOD\JFeng.M
    Last changed    : 11/10/2019 15:42:49 by
                      (modified after loading)
    Analysis Method : F:\METHOD\ZK.M
    Last changed    : 14/07/2019 15:34:15 by
    Additional Info : Peak(s) manually integrated
```

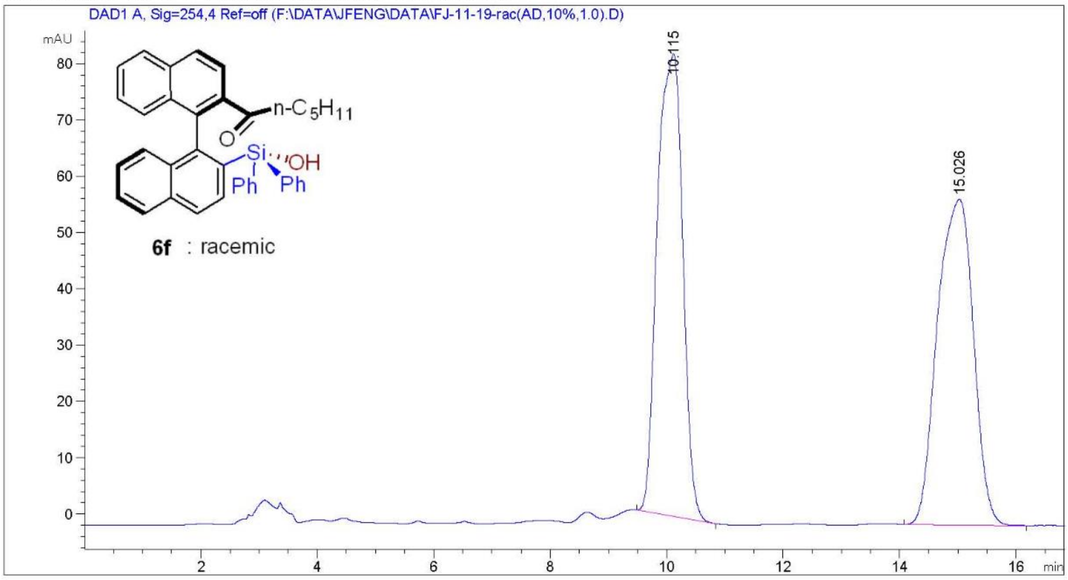


```
    =====================================================================
                         Area Percent Report
    =====================================================================

    Sorted By             :      Signal
    Multiplier            :      1.0000
    Dilution              :      1.0000
    Use Multiplier & Dilution Factor with ISTDs


    Signal 1: DAD1 A, Sig=254,4 Ref=off

    Peak RetTime Type  Width     Area      Height     Area
      #   [min]        [min]   [mAU*s]     [mAU]        %
    ----|-------|----|-------|----------|----------|--------|
       1  10.115 BB    0.4213 2487.20947   82.14089  50.0878
       2  15.026 BB    0.7055 2478.48828   57.89181  49.9122

    Totals :                  4965.69775  140.03270


LC1260 30/11/2019 15:09:11                                        Page    1 of 2
```

Supplementary Figure 155. HPLC data of **6f**

Data File F:\DATA\JFENG\DATA\FJ-11-18(AD,10%,1.0).D
Sample Name: FJ-11-18(AD,10%,1.0)

===

Acq. Operator :
Sample Operator :
Acq. Instrument : LC1260 Location : 1
Injection Date : 11/10/2019 16:52:17
Inj Volume : Manually
Acq. Method : F:\METHOD\JFeng.M
Last changed : 11/10/2019 15:42:49 by
(modified after loading)
Analysis Method : F:\METHOD\ZK.M
Last changed : 14/07/2019 15:34:15 by
Additional Info : Peak(s) manually integrated

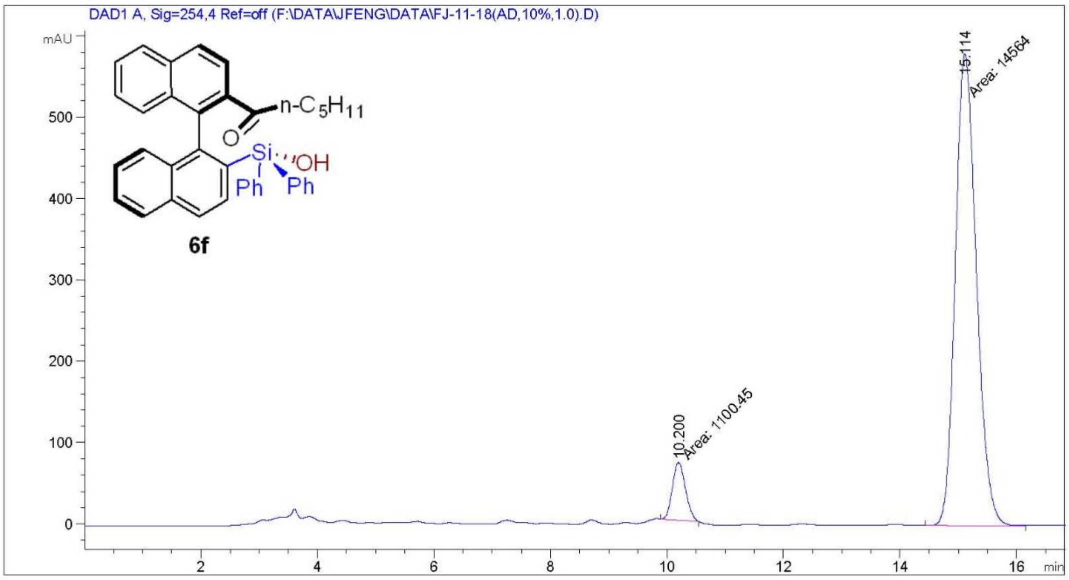

===

Area Percent Report

===

Sorted By : Signal
Multiplier : 1.0000
Dilution : 1.0000
Use Multiplier & Dilution Factor with ISTDs

Signal 1: DAD1 A, Sig=254,4 Ref=off

Peak #	RetTime [min]	Type	Width [min]	Area [mAU*s]	Height [mAU]	Area %
1	10.200	MM	0.2587	1100.44836	70.89850	7.0251
2	15.114	MM	0.4187	1.45640e4	579.76477	92.9749

Totals : 1.56645e4 650.66327

LC1260 30/11/2019 15:12:24 Page 1 of 2

Supplementary Figure 156. HPLC data of **6f**

Data File F:\DATA\JFENG\DATA\LN-1-9(AD,20%,1.0).D
Sample Name: LN-1-9(AD,20%,1.0)

===

Acq. Operator :
Sample Operator :
Acq. Instrument : LC1260 Location : 1
Injection Date : 14/10/2019 16:15:31
Inj Volume : No inj
Acq. Method : C:\Chem32\1\Methods\DEF_LC.M
Last changed : 14/10/2019 15:32:00 by
(modified after loading)
Analysis Method : F:\METHOD\ZK.M
Last changed : 14/07/2019 15:34:15 by
Additional Info : Peak(s) manually integrated

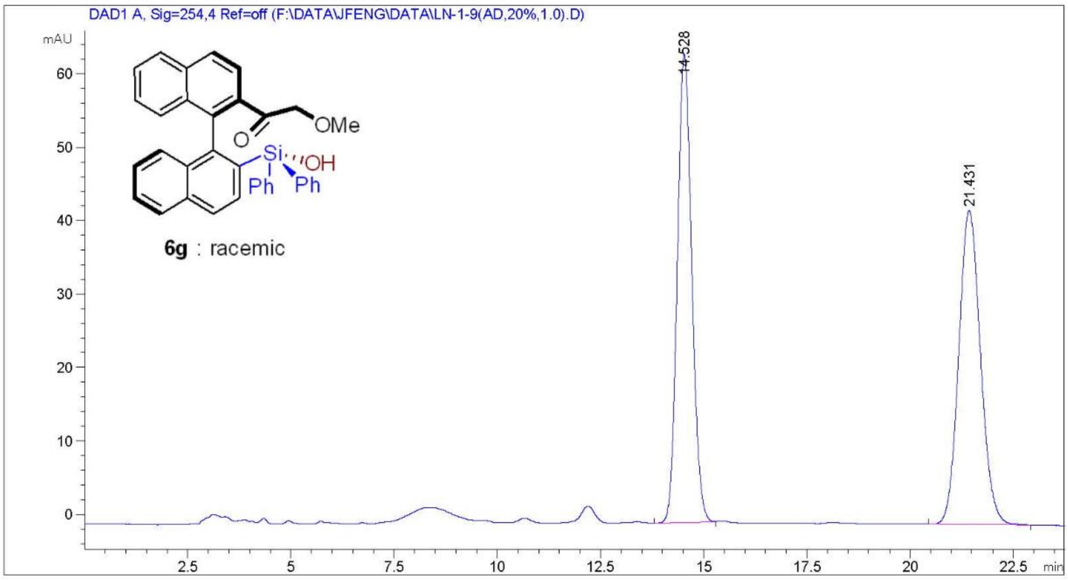

===

Area Percent Report

===

Sorted By : Signal
Multiplier : 1.0000
Dilution : 1.0000
Use Multiplier & Dilution Factor with ISTDs

Signal 1: DAD1 A, Sig=254,4 Ref=off

Peak #	RetTime [min]	Type	Width [min]	Area [mAU*s]	Height [mAU]	Area %
1	14.528	BB	0.3775	1549.51868	63.77249	49.8408
2	21.431	BB	0.5662	1559.41711	42.68543	50.1592

Totals : 3108.93579 106.45792

LC1260 30/11/2019 09:59:52 Page 1 of 2

Supplementary Figure 157. HPLC data of **6g**

Data File F:\DATA\JFENG\DATA\BXF-2-85(AD,80%,1.0).D
Sample Name: BXF-2-85(AD,80%,1.0)

===

Acq. Operator :
Sample Operator :
Acq. Instrument : LC1260 Location : 1
Injection Date : 16/10/2019 19:40:12
Inj Volume : No inj
Acq. Method : F:\METHOD\ZK.M
Last changed : 16/10/2019 19:31:05 by
(modified after loading)
Analysis Method : F:\METHOD\ZK.M
Last changed : 14/07/2019 15:34:15 by
Additional Info : Peak(s) manually integrated

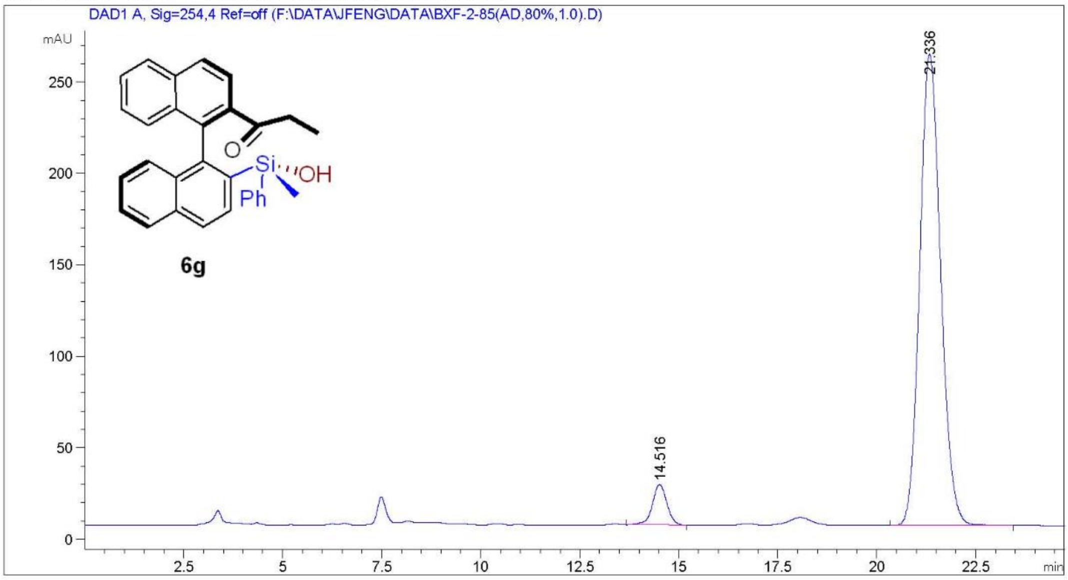

===

Area Percent Report

===

Sorted By : Signal
Multiplier : 1.0000
Dilution : 1.0000
Use Multiplier & Dilution Factor with ISTDs

Signal 1: DAD1 A, Sig=254,4 Ref=off

Peak #	RetTime [min]	Type	Width [min]	Area [mAU*s]	Height [mAU]	Area %
1	14.516	BB	0.3906	561.67645	22.09038	5.6016
2	21.336	BB	0.5726	9465.40332	257.65445	94.3984

Totals : 1.00271e4 279.74483

Supplementary Figure 158. HPLC data of **6g**

Data File F:\DATA\JFENG\DATA\FJ-11-80(AD,20%,1.0).D
Sample Name: FJ-11-80(AD,20%,1.0)

===

Acq. Operator :

Location : 1

Injection Date : 14/11/2019 20:51:16
Acq. Method : DEF_LC.M
Analysis Method : F:\METHOD\ZK.M
Last changed : 14/07/2019 15:34:15 by
Additional Info : Peak(s) manually integrated

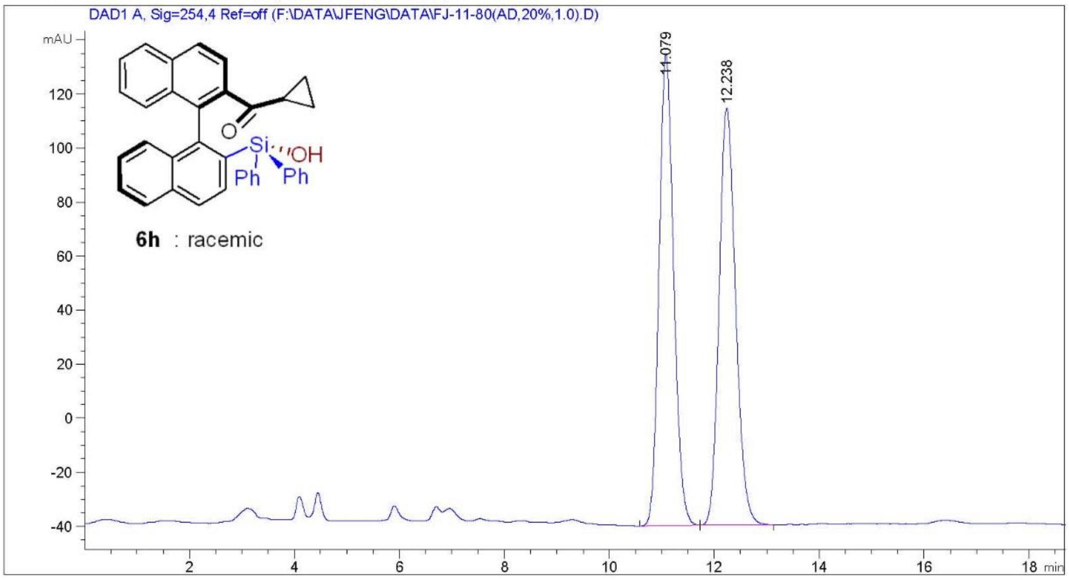

===

Area Percent Report

===

Sorted By : Signal
Multiplier : 1.0000
Dilution : 1.0000
Use Multiplier & Dilution Factor with ISTDs

Signal 1: DAD1 A, Sig=254,4 Ref=off

Peak #	RetTime [min]	Type	Width [min]	Area [mAU*s]	Height [mAU]	Area %
1	11.079	BB	0.2995	3369.19971	174.28862	49.9996
2	12.238	BB	0.3367	3369.25903	154.30183	50.0004

Totals : 6738.45874 328.59045

===

*** End of Report ***

Supplementary Figure 159. HPLC data of **6h**

Data File F:\DATA\JFENG\DATA\FJ-11-79-re(AD,20%,1.0).D
Sample Name: FJ-11-79-re(AD,20%,1.0)

===

Acq. Operator   :
Sample Operator :
Acq. Instrument : LC1260   Location :   1
Injection Date : 17/11/2019 20:32:33
                                                 Inj Volume : Manually
Acq. Method   : F:\METHOD\JFeng.M
Last changed   : 17/11/2019 20:02:36 by
                  (modified after loading)
Analysis Method : F:\METHOD\ZK.M
Last changed   : 14/07/2019 15:34:15 by
Additional Info : Peak(s) manually integrated

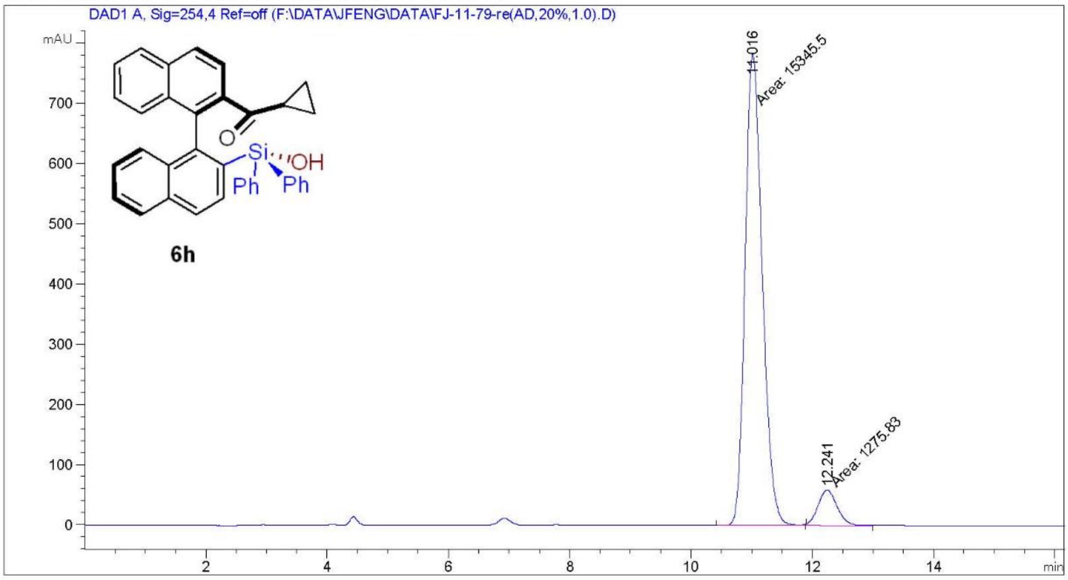

===

Area Percent Report

===

Sorted By : Signal
Multiplier : 1.0000
Dilution : 1.0000
Use Multiplier & Dilution Factor with ISTDs

Signal 1: DAD1 A, Sig=254,4 Ref=off

Peak #	RetTime [min]	Type	Width [min]	Area [mAU*s]	Height [mAU]	Area %
1	11.016	MM	0.3266	1.53455e4	783.07855	92.3242
2	12.241	MM	0.3632	1275.82849	58.54312	7.6758

Totals : 1.66214e4 841.62167

LC1260 30/11/2019 15:53:11 Page 1 of 2

Supplementary Figure 160. HPLC data of **6h**

```
Data File F:\DATA\JFENG\DATA\BXF-2-28(AD-H,15%,1.0).D
Sample Name: BXF-2-28(AD-H,15%,1.0)

=====================================================================
Acq. Operator   :
                                                 Location :   1
Injection Date  : 09/09/2019 20:22:35
Acq. Method     : DEF_LC.M
Analysis Method : F:\METHOD\ZK.M
Last changed    : 14/07/2019 15:34:15 by
Additional Info : Peak(s) manually integrated
```

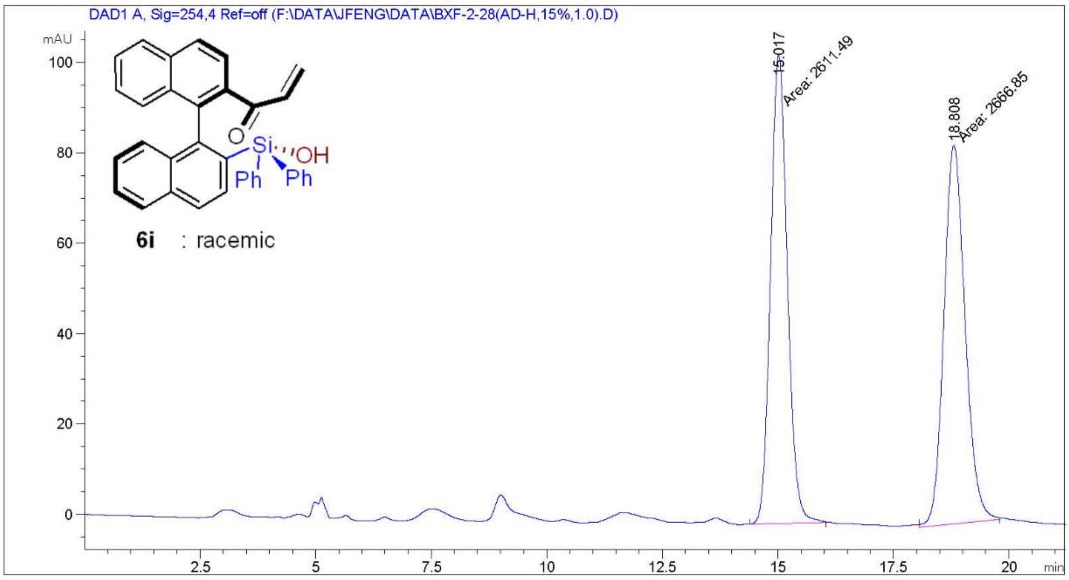


```
=====================================================================
                        Area Percent Report
=====================================================================

Sorted By             :      Signal
Multiplier            :      1.0000
Dilution              :      1.0000
Use Multiplier & Dilution Factor with ISTDs


Signal 1: DAD1 A, Sig=254,4 Ref=off

Peak RetTime Type  Width     Area      Height     Area
  #   [min]        [min]   [mAU*s]     [mAU]        %
----|-------|----|-------|----------|----------|--------|
   1  15.017 MM    0.4193 2611.48633  103.79387  49.4755
   2  18.808 MM    0.5307 2666.85132   83.75375  50.5245

Totals :                  5278.33765  187.54762


=====================================================================
                         *** End of Report ***


LC1260 30/11/2019 09:36:17                                      Page    1 of 1
```

Supplementary Figure 161. HPLC data of **6i**

Data File F:\DATA\JFENG\DATA\BXF-2-33-repeat(AD,15%,1.0).D
Sample Name: BXF-2-33-repeat(AD,15%,1.0)

===

Acq. Operator :

Location : 1

Injection Date : 16/09/2019 21:56:43
Acq. Method : DEF_LC.M
Analysis Method : F:\METHOD\ZK.M
Last changed : 14/07/2019 15:34:15 by
Additional Info : Peak(s) manually integrated

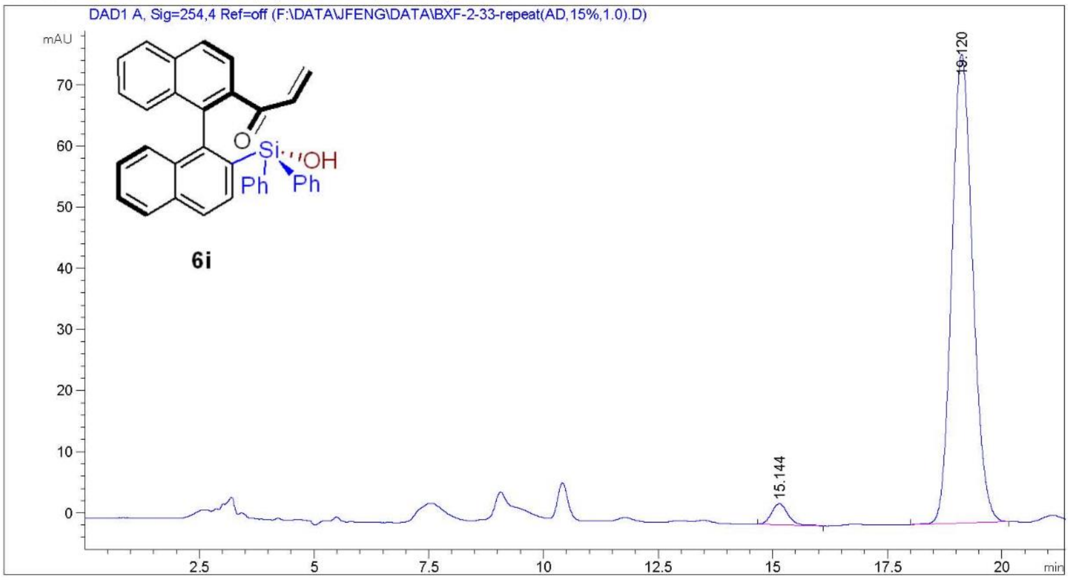

===

Area Percent Report

===

Sorted By : Signal
Multiplier : 1.0000
Dilution : 1.0000
Use Multiplier & Dilution Factor with ISTDs

Signal 1: DAD1 A, Sig=254,4 Ref=off

Peak #	RetTime [min]	Type	Width [min]	Area [mAU*s]	Height [mAU]	Area %
1	15.144	BB	0.3823	83.10104	3.43480	3.2995
2	19.120	BB	0.4930	2435.51465	76.59618	96.7005

Totals : 2518.61569 80.03099

===

*** End of Report ***

LC1260 30/11/2019 09:39:10 Page 1 of 1

Supplementary Figure 162. HPLC data of **6i**

Data File F:\DATA\JFENG\DATA\FJ-11-23-rac(AD,10%,1.0).D
Sample Name: FJ-11-23-rac(AD,10%,1.0)

===

Acq. Operator :
Sample Operator :
Acq. Instrument : LC1260 Location : 1
Injection Date : 04/12/2019 19:37:19
Inj Volume : Manually
Acq. Method : F:\METHOD\JFeng.M
Last changed : 04/12/2019 19:29:04 by
(modified after loading)
Analysis Method : C:\Chem32\1\Methods\DEF_LC.M
Last changed : 13/02/2014 23:27:44 by SYSTEM
Additional Info : Peak(s) manually integrated

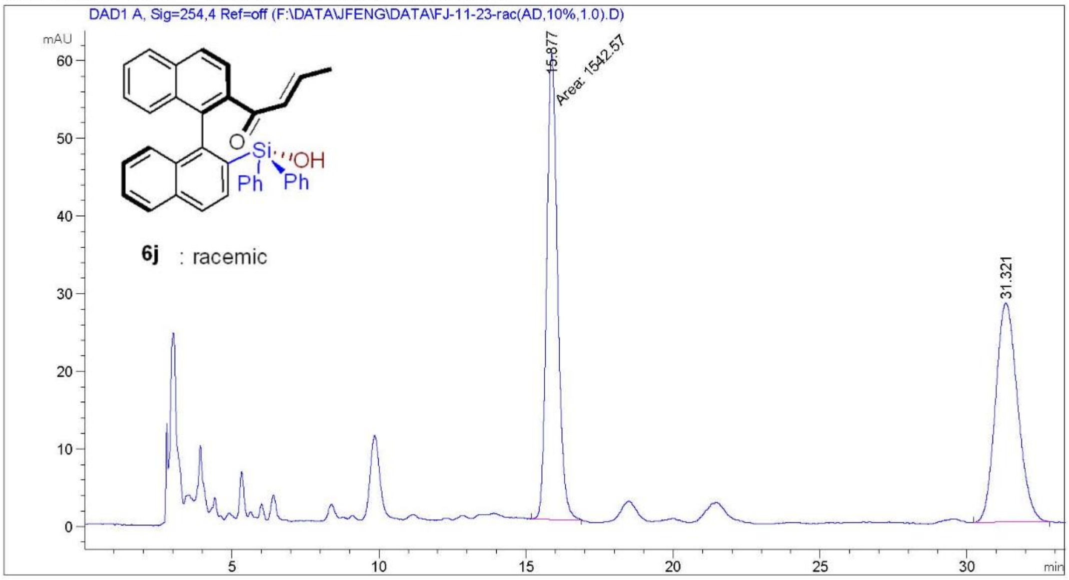

===
Area Percent Report
===

Sorted By : Signal
Multiplier : 1.0000
Dilution : 1.0000
Use Multiplier & Dilution Factor with ISTDs

Signal 1: DAD1 A, Sig=254,4 Ref=off

Peak #	RetTime [min]	Type	Width [min]	Area [mAU*s]	Height [mAU]	Area %
1	15.877	MM	0.4289	1542.57129	59.94010	51.0402
2	31.321	BB	0.7572	1479.69543	28.24319	48.9598

Totals : 3022.26672 88.18329

LC1260 04/12/2019 20:13:56 Page 1 of 2

Supplementary Figure 163. HPLC data of **6j**

Data File F:\DATA\JFENG\DATA\FJ-10-119(AD,10%,1.0).D
Sample Name: FJ-10-119(AD,10%,1.0)

===

Acq. Operator :
Sample Operator :
Acq. Instrument : LC1260 Location : 1
Injection Date : 10/07/2019 10:26:22
Inj Volume : No inj
Acq. Method : C:\Chem32\1\Methods\DEF_LC.M
Last changed : 10/07/2019 09:37:25 by
(modified after loading)
Analysis Method : C:\Chem32\1\Methods\DEF_LC.M
Last changed : 13/02/2014 23:27:44 by SYSTEM
Additional Info : Peak(s) manually integrated

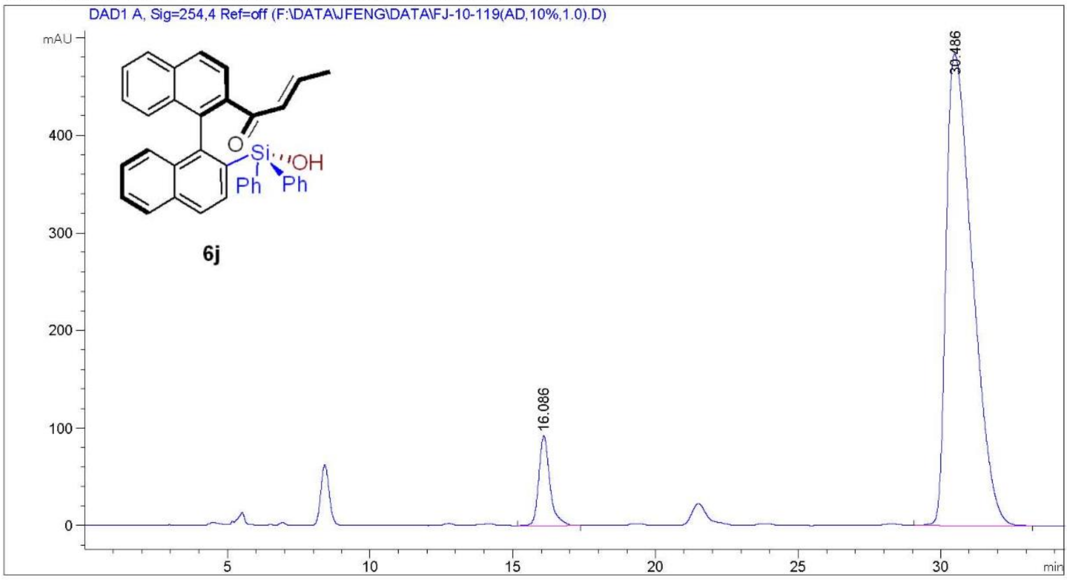

===

Area Percent Report

===

Sorted By : Signal
Multiplier : 1.0000
Dilution : 1.0000
Use Multiplier & Dilution Factor with ISTDs

Signal 1: DAD1 A, Sig=254,4 Ref=off

Peak #	RetTime [min]	Type	Width [min]	Area [mAU*s]	Height [mAU]	Area %
1	16.086	BB	0.4097	2490.03491	92.01404	7.4696
2	30.486	BB	0.9607	3.08457e4	483.23419	92.5304

Totals : 3.33357e4 575.24823

LC1260 04/12/2019 19:08:20 Page 1 of 2

Supplementary Figure 164. HPLC data of **6j**

Data File F:\DATA\JFENG\DATA\FJ-10-168(AD,20%,1.0).D
Sample Name: FJ-10-168(AD,20%,1.0)

===
Acq. Operator :
Location : 1
Injection Date : 06/09/2019 10:47:06
Acq. Method : JFeng.M
Analysis Method : F:\METHOD\ZK.M
Last changed : 14/07/2019 15:34:15 by
Additional Info : Peak(s) manually integrated

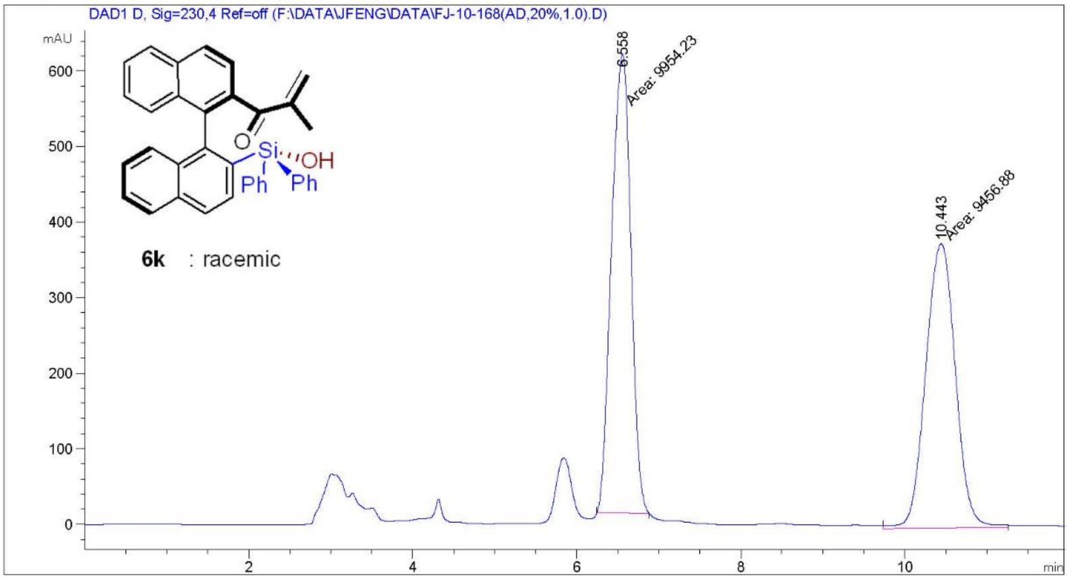

===
Area Percent Report
===

Sorted By : Signal
Multiplier : 1.0000
Dilution : 1.0000
Use Multiplier & Dilution Factor with ISTDs

Signal 1: DAD1 D, Sig=230,4 Ref=off

Peak #	RetTime [min]	Type	Width [min]	Area [mAU*s]	Height [mAU]	Area %
1	6.558	MM	0.2729	9954.22949	608.02948	51.2811
2	10.443	MM	0.4187	9456.88184	376.44547	48.7189

Totals : 1.94111e4 984.47495

===
*** End of Report ***

LC1260 30/11/2019 16:07:52 Page 1 of 1

Supplementary Figure 165. HPLC data of **6k**

Data File F:\DATA\JFENG\DATA\FJ-10-166(AD,20%,1.0).D
Sample Name: FJ-10-166(AD,20%,1.0)

===

Acq. Operator :
Sample Operator :
Acq. Instrument : LC1260 Location : 1
Injection Date : 06/09/2019 11:02:35
Inj Volume : Manually
Acq. Method : F:\METHOD\JFeng.M
Last changed : 06/09/2019 10:15:53 by
(modified after loading)
Analysis Method : F:\METHOD\ZK.M
Last changed : 14/07/2019 15:34:15 by
Additional Info : Peak(s) manually integrated

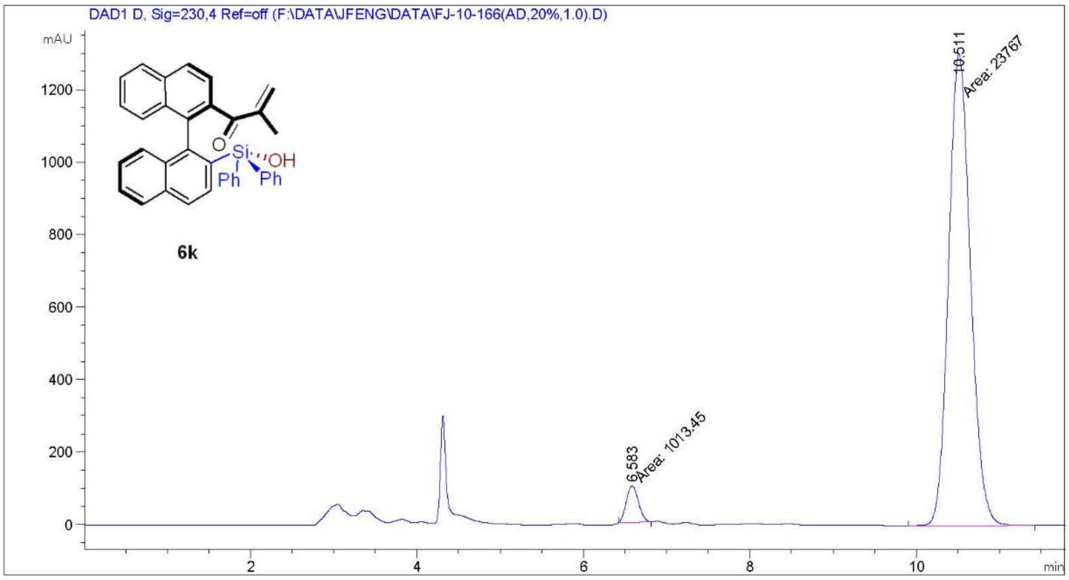

===

Area Percent Report

===

Sorted By : Signal
Multiplier : 1.0000
Dilution : 1.0000
Use Multiplier & Dilution Factor with ISTDs

Signal 1: DAD1 D, Sig=230,4 Ref=off

Peak #	RetTime [min]	Type	Width [min]	Area [mAU*s]	Height [mAU]	Area %
1	6.583	MM	0.1672	1013.44720	101.00916	4.0897
2	10.511	MM	0.3043	2.37670e4	1301.72522	95.9103
Totals :				2.47804e4	1402.73438	

LC1260 30/11/2019 16:02:01 Page 1 of 2

Supplementary Figure 166. HPLC data of **6k**

Data File F:\DATA\JFENG\DATA\LN-1-6-2(AD,20%,1.0).D
Sample Name: LN-1-6-2(AD,20%,1.0)

===

Acq. Operator :

Location : 1

Injection Date : 25/09/2019 20:35:22
Acq. Method : DEF_LC.M
Analysis Method : F:\METHOD\ZK.M
Last changed : 14/07/2019 15:34:15 by
Additional Info : Peak(s) manually integrated

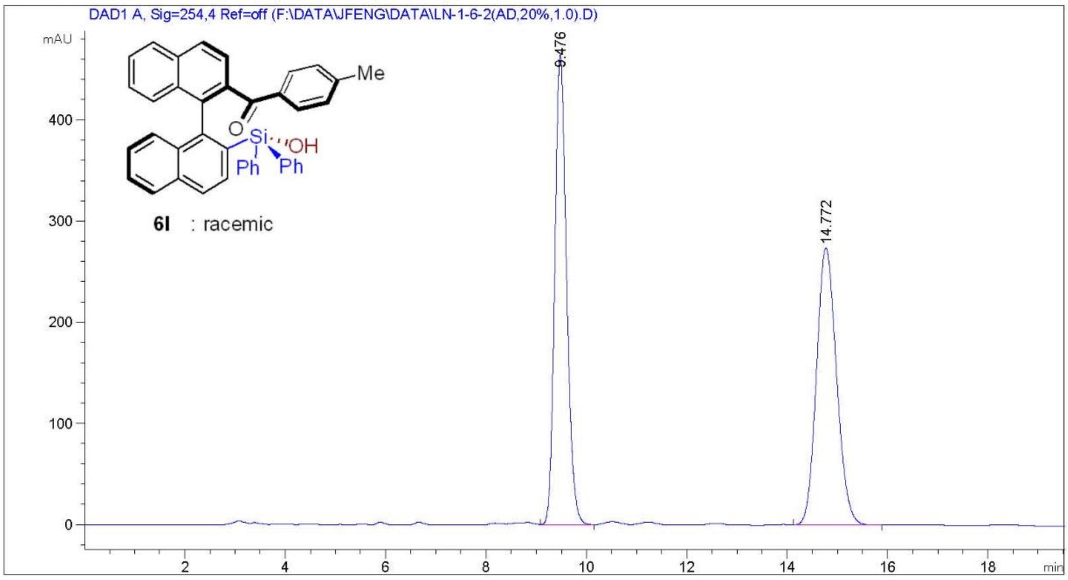

===

Area Percent Report

===

Sorted By : Signal
Multiplier : 1.0000
Dilution : 1.0000
Use Multiplier & Dilution Factor with ISTDs

Signal 1: DAD1 A, Sig=254,4 Ref=off

Peak #	RetTime [min]	Type	Width [min]	Area [mAU*s]	Height [mAU]	Area %
1	9.476	BB	0.2499	7564.23047	464.71857	49.9657
2	14.772	BB	0.4291	7574.60352	273.54688	50.0343

Totals : 1.51388e4 738.26544

===

*** End of Report ***

LC1260 30/11/2019 09:50:47 Page 1 of 1

Supplementary Figure 167. HPLC data of **6l**

Data File F:\DATA\JFENG\DATA\BXF-2-57(AD,20%,1.0).D
Sample Name: BXF-2-57(AD,20%,1.0)

===

Acq. Operator :
Sample Operator :
Acq. Instrument : LC1260 Location : 1
Injection Date : 23/09/2019 22:03:11
Inj Volume : No inj
Acq. Method : C:\Chem32\1\Methods\DEF_LC.M
Last changed : 23/09/2019 20:34:31 by
(modified after loading)
Analysis Method : F:\METHOD\ZK.M
Last changed : 14/07/2019 15:34:15 by
Additional Info : Peak(s) manually integrated

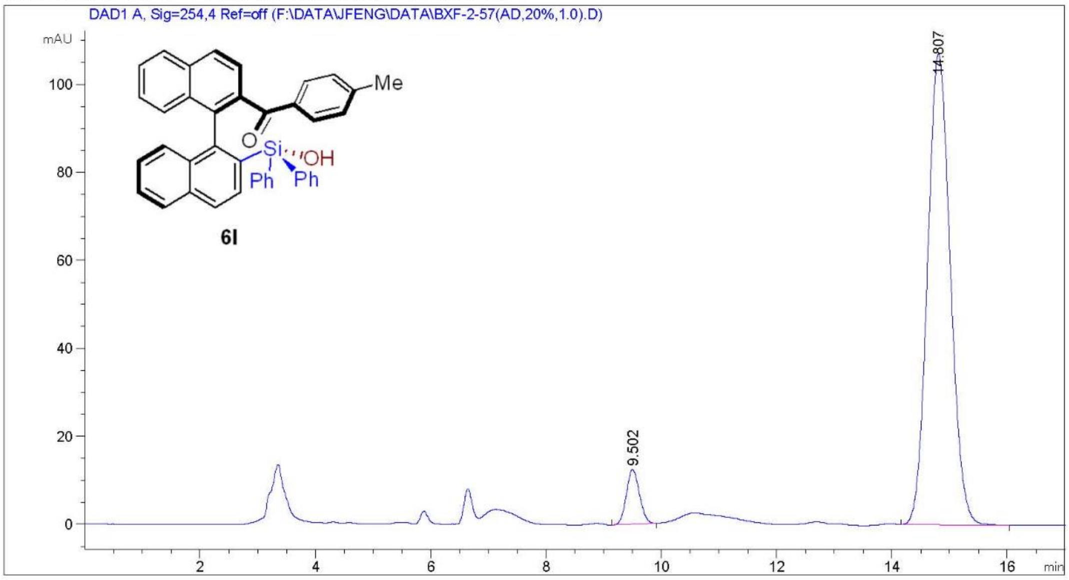

===
Area Percent Report
===

Sorted By : Signal
Multiplier : 1.0000
Dilution : 1.0000
Use Multiplier & Dilution Factor with ISTDs

Signal 1: DAD1 A, Sig=254,4 Ref=off

Peak #	RetTime [min]	Type	Width [min]	Area [mAU*s]	Height [mAU]	Area %
1	9.502	BB	0.2424	194.62357	12.44700	6.2040
2	14.807	BB	0.4286	2942.44897	107.06157	93.7960

Totals : 3137.07254 119.50857

LC1260 30/11/2019 09:52:11 Page 1 of 2

Supplementary Figure 168. HPLC data of **6l**

Data File F:\DATA\JFENG\DATA\LN-1-7(AD,20%,1.0).D
Sample Name: LN-1-7(AD,20%,1.0)

===

Acq. Operator :
Sample Operator :
Acq. Instrument : LC1260 Location : 1
Injection Date : 20/09/2019 20:40:05
Inj Volume : No inj
Acq. Method : C:\Chem32\1\Methods\DEF_LC.M
Last changed : 20/09/2019 20:00:32 by
(modified after loading)
Analysis Method : F:\METHOD\ZK.M
Last changed : 14/07/2019 15:34:15 by
Additional Info : Peak(s) manually integrated

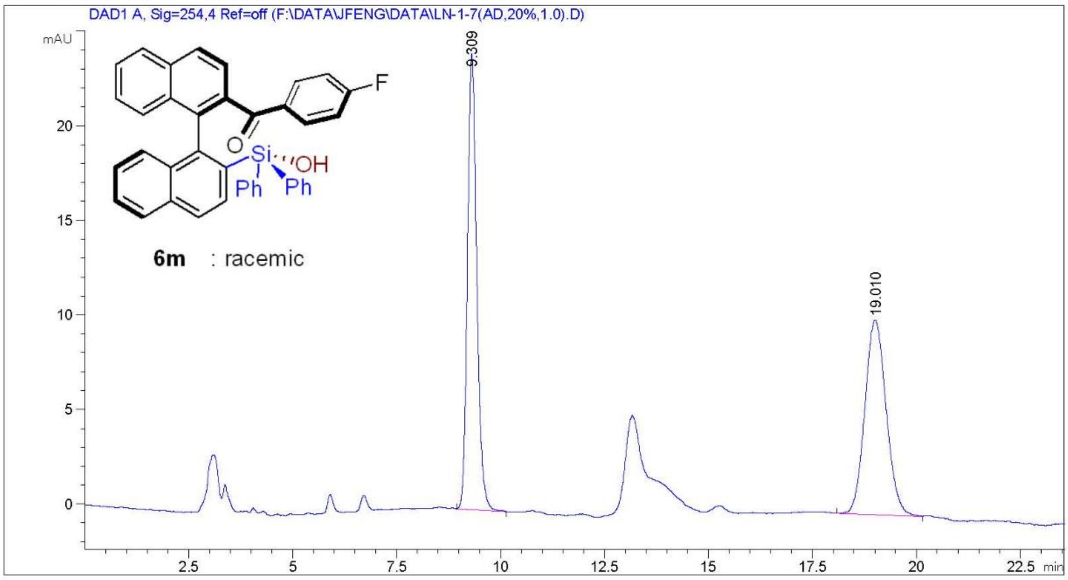

===

Area Percent Report

===

Sorted By : Signal
Multiplier : 1.0000
Dilution : 1.0000
Use Multiplier & Dilution Factor with ISTDs

Signal 1: DAD1 A, Sig=254,4 Ref=off

Peak #	RetTime [min]	Type	Width [min]	Area [mAU*s]	Height [mAU]	Area %
1	9.309	BB	0.2391	373.89233	24.08443	50.5058
2	19.010	BB	0.5490	366.40375	10.30097	49.4942

Totals : 740.29608 34.38540

LC1260 30/11/2019 09:57:19 Page 1 of 2

Supplementary Figure 169. HPLC data of **6m**

Data File F:\DATA\JFENG\DATA\BXF-2-56-1(AD,20%,1.0).D
Sample Name: BXF-2-56-1(AD,20%,1.0)

===

Acq. Operator :
Sample Operator :
Acq. Instrument : LC1260 Location : 1
Injection Date : 27/09/2019 21:12:03
Inj Volume : No inj
Acq. Method : C:\Chem32\1\Methods\DEF_LC.M
Last changed : 27/09/2019 19:56:02 by
(modified after loading)
Analysis Method : F:\METHOD\ZK.M
Last changed : 14/07/2019 15:34:15 by
Additional Info : Peak(s) manually integrated

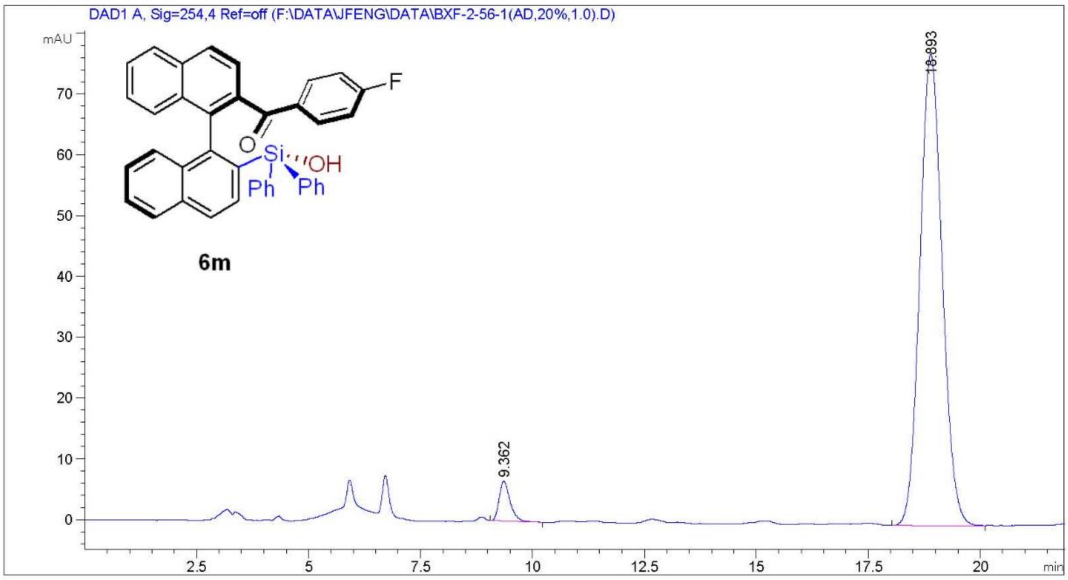

===
Area Percent Report
===

Sorted By : Signal
Multiplier : 1.0000
Dilution : 1.0000
Use Multiplier & Dilution Factor with ISTDs

Signal 1: DAD1 A, Sig=254,4 Ref=off

Peak #	RetTime [min]	Type	Width [min]	Area [mAU*s]	Height [mAU]	Area %
1	9.362	BB	0.2590	110.51694	6.54432	3.9392
2	18.893	BB	0.5395	2695.06079	77.54342	96.0608

Totals : 2805.57773 84.08773

LC1260 30/11/2019 09:58:00 Page 1 of 2

Supplementary Figure 170. HPLC data of **6m**

Data File F:\DATA\JFENG\DATA\BXF-2-106(AD,20%,1.0).D
Sample Name: BXF-2-106(AD,20%,1.0)

===

Acq. Operator :
Sample Operator :
Acq. Instrument : LC1260 Location : 1
Injection Date : 23/10/2019 20:58:57
Inj Volume : No inj
Acq. Method : C:\Chem32\1\Methods\DEF_LC.M
Last changed : 23/10/2019 20:50:34 by
(modified after loading)
Analysis Method : F:\METHOD\ZK.M
Last changed : 14/07/2019 15:34:15 by
Additional Info : Peak(s) manually integrated

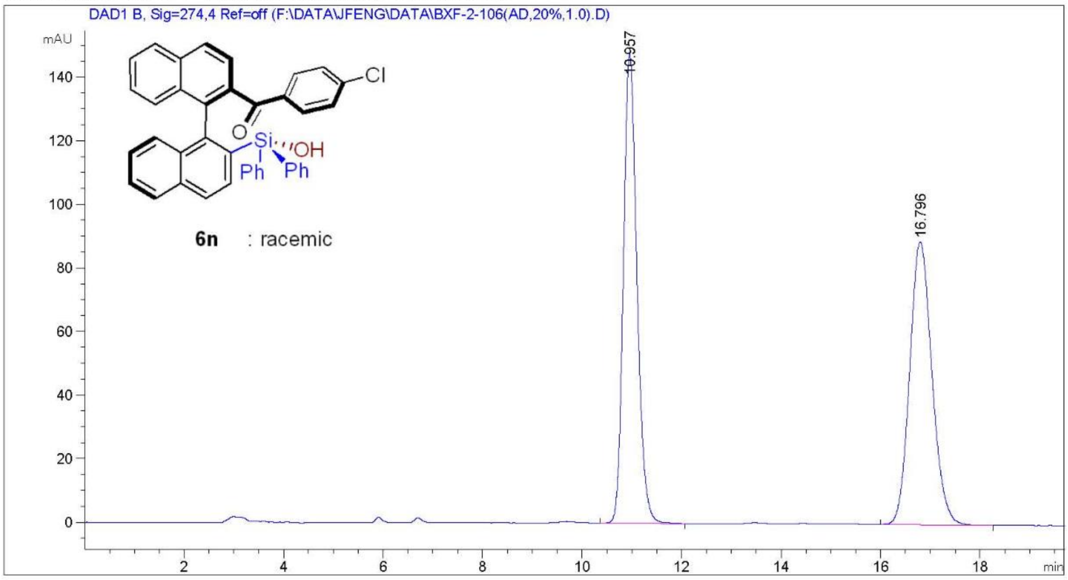

===

Area Percent Report

===

Sorted By : Signal
Multiplier : 1.0000
Dilution : 1.0000
Use Multiplier & Dilution Factor with ISTDs

Signal 1: DAD1 B, Sig=274,4 Ref=off

Peak #	RetTime [min]	Type	Width [min]	Area [mAU*s]	Height [mAU]	Area %
1	10.957	BB	0.2869	2769.13013	147.61443	50.0165
2	16.796	BB	0.4811	2767.30737	88.92298	49.9835

Totals : 5536.43750 236.53741

LC1260 30/11/2019 10:11:53 Page 1 of 2

Supplementary Figure 171. HPLC data of **6n**

Data File F:\DATA\JFENG\DATA\BXF-2-101(AD,20%,1.0).D
Sample Name: BXF-2-101(AD,20%,1.0)

===
Acq. Operator :
Location : 1
Injection Date : 23/10/2019 21:27:47
Acq. Method : DEF_LC.M
Analysis Method : F:\METHOD\ZK.M
Last changed : 14/07/2019 15:34:15 by
Additional Info : Peak(s) manually integrated

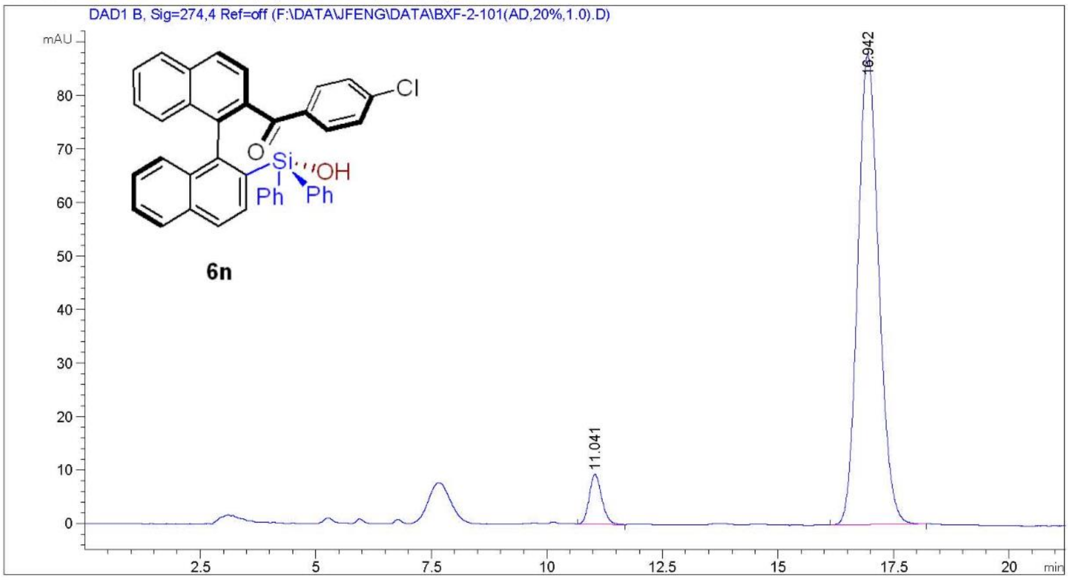

===
Area Percent Report
===

Sorted By : Signal
Multiplier : 1.0000
Dilution : 1.0000
Use Multiplier & Dilution Factor with ISTDs

Signal 1: DAD1 B, Sig=274,4 Ref=off

Peak #	RetTime [min]	Type	Width [min]	Area [mAU*s]	Height [mAU]	Area %
1	11.041	BB	0.2906	174.70988	9.32589	5.9194
2	16.942	BB	0.4908	2776.75806	87.85071	94.0806

Totals : 2951.46794 97.17660

===
*** End of Report ***

LC1260 30/11/2019 10:11:23 Page 1 of 1

Supplementary Figure 172. HPLC data of **6n**

Data File F:\DATA\JFENG\DATA\FJ-10-124-2(AD,20%,1.0).D
Sample Name: FJ-10-124-2(AD,20%,1.0)

===

Acq. Operator :

Location : 1

Injection Date : 12/07/2019 17:51:20
Acq. Method : DEF_LC.M
Analysis Method : F:\METHOD\ZK.M
Last changed : 14/07/2019 15:34:15 by
Additional Info : Peak(s) manually integrated

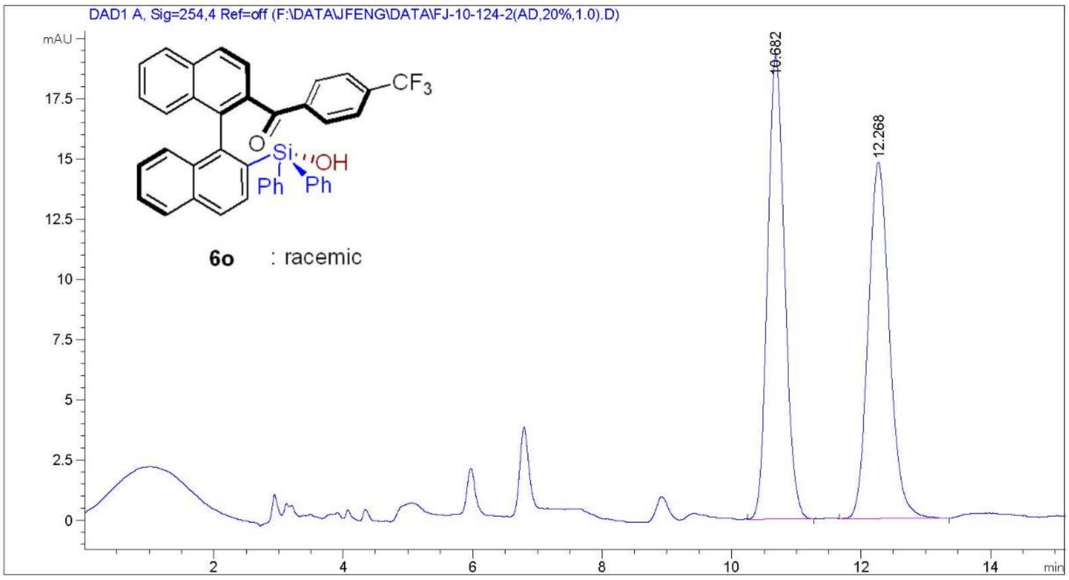

===

Area Percent Report

===

Sorted By : Signal
Multiplier : 1.0000
Dilution : 1.0000
Use Multiplier & Dilution Factor with ISTDs

Signal 1: DAD1 A, Sig=254,4 Ref=off

Peak #	RetTime [min]	Type	Width [min]	Area [mAU*s]	Height [mAU]	Area %
1	10.682	BB	0.2819	349.90295	19.26788	50.9608
2	12.268	BB	0.3499	336.70950	14.77239	49.0392

Totals : 686.61246 34.04027

===

*** End of Report ***

LC1260 30/11/2019 10:21:45 Page 1 of 1

Supplementary Figure 173. HPLC data of **6o**

```
Data File F:\DATA\JFENG\DATA\FJ-10-123-20190716(AD,20%,1.0).D
Sample Name: FJ-10-123-20190716(AD,20%,1.0)

=====================================================================
Acq. Operator   :
Sample Operator :
Acq. Instrument : LC1260                          Location :   1
Injection Date  : 16/07/2019 12:19:17
                                                Inj Volume : No inj
Acq. Method     : C:\Chem32\1\Methods\DEF_LC.M
Last changed    : 16/07/2019 11:46:04 by
                  (modified after loading)
Analysis Method : F:\METHOD\ZK.M
Last changed    : 14/07/2019 15:34:15 by
Additional Info : Peak(s) manually integrated
```

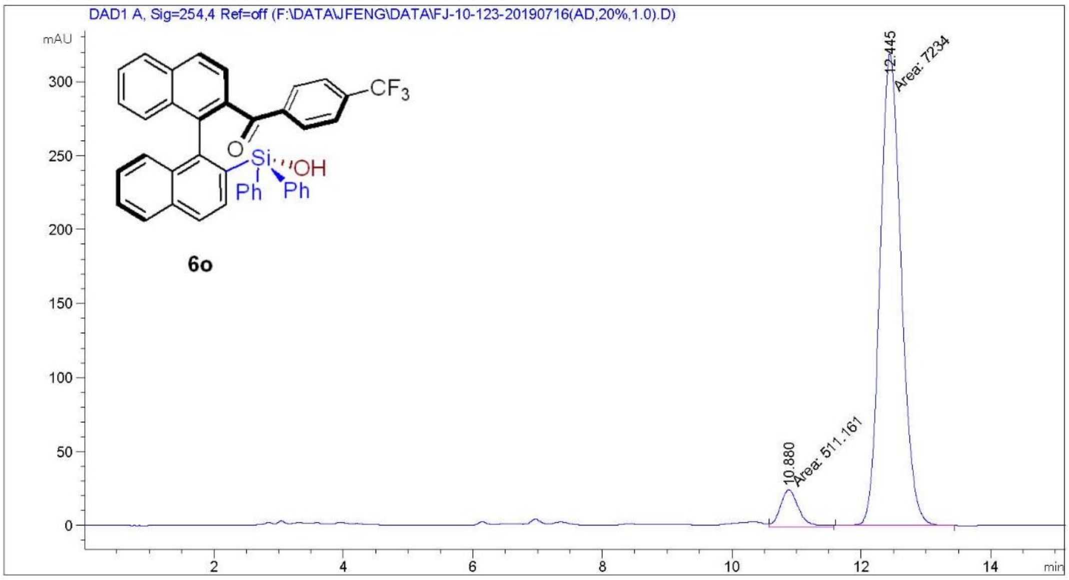


```
=====================================================================
                         Area Percent Report
=====================================================================

Sorted By             :      Signal
Multiplier            :      1.0000
Dilution              :      1.0000
Use Multiplier & Dilution Factor with ISTDs


Signal 1: DAD1 A, Sig=254,4 Ref=off

Peak RetTime Type  Width     Area      Height     Area
  #   [min]        [min]   [mAU*s]     [mAU]        %
----|-------|----|-------|----------|----------|--------|
   1  10.880 MM    0.3366  511.16074   25.30775   6.5997
   2  12.445 MM    0.3784 7234.00195  318.59116  93.4003

Totals :                  7745.16269  343.89891
```

LC1260 30/11/2019 10:23:55   Page   1 of 2

Supplementary Figure 174. HPLC data of **6o**

```
Data File F:\DATA\JFENG\DATA\BXF-2-91(AD,80%,1.0).D
Sample Name: BXF-2-91(AD,80%,1.0)

=====================================================================
Acq. Operator   :
Sample Operator :
Acq. Instrument : LC1260                          Location :   1
Injection Date  : 16/10/2019 20:12:06
                                                Inj Volume : No inj
Acq. Method     : F:\METHOD\ZK.M
Last changed    : 16/10/2019 19:31:05 by
                  (modified after loading)
Analysis Method : F:\METHOD\ZK.M
Last changed    : 14/07/2019 15:34:15 by
Additional Info : Peak(s) manually integrated
```

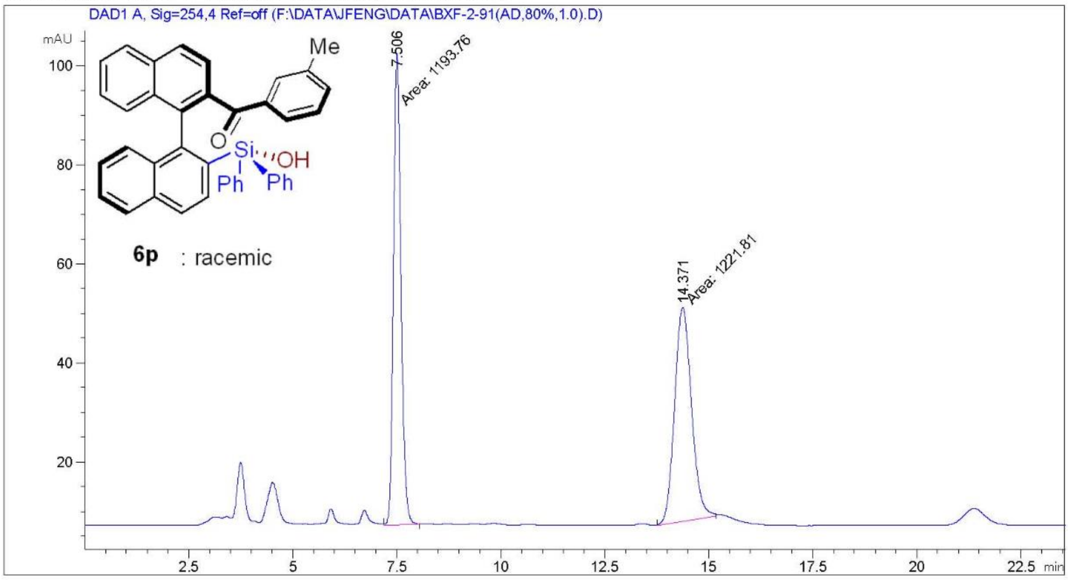


```
=====================================================================
                        Area Percent Report
=====================================================================

Sorted By             :      Signal
Multiplier            :      1.0000
Dilution              :      1.0000
Use Multiplier & Dilution Factor with ISTDs


Signal 1: DAD1 A, Sig=254,4 Ref=off
```

Peak #	RetTime [min]	Type	Width [min]	Area [mAU*s]	Height [mAU]	Area %
1	7.506	MM	0.2091	1193.75562	95.14936	49.4194
2	14.371	MM	0.4715	1221.80725	43.18851	50.5806

Totals : 2415.56287 138.33787

LC1260 30/11/2019 10:19:41   Page 1 of 2

Supplementary Figure 175. HPLC data of **6p**

Data File F:\DATA\JFENG\DATA\BXF-2-90(AD,80%,1.0).D
Sample Name: BXF-2-90(AD,80%,1.0)

===

Acq. Operator :
Sample Operator :
Acq. Instrument : LC1260 Location : 1
Injection Date : 16/10/2019 20:42:01
Inj Volume : No inj
Acq. Method : F:\METHOD\ZK.M
Last changed : 16/10/2019 19:31:05 by
(modified after loading)
Analysis Method : F:\METHOD\ZK.M
Last changed : 14/07/2019 15:34:15 by
Additional Info : Peak(s) manually integrated

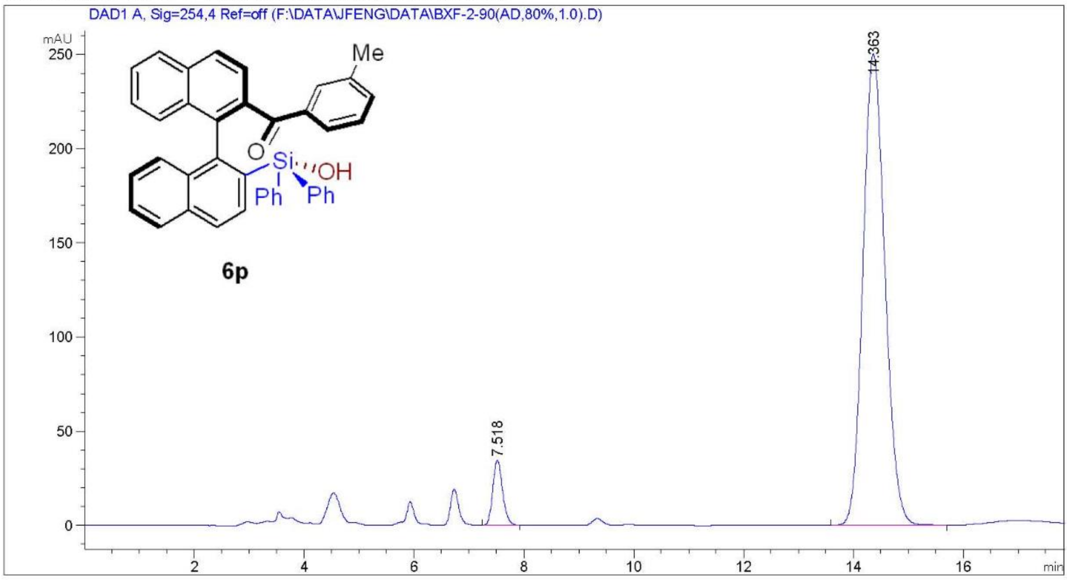

===
Area Percent Report
===

Sorted By : Signal
Multiplier : 1.0000
Dilution : 1.0000
Use Multiplier & Dilution Factor with ISTDs

Signal 1: DAD1 A, Sig=254,4 Ref=off

Peak #	RetTime [min]	Type	Width [min]	Area [mAU*s]	Height [mAU]	Area %
1	7.518	BB	0.1887	421.23117	34.44719	5.7475
2	14.363	BB	0.4302	6907.75732	250.14508	94.2525

Totals : 7328.98849 284.59227

LC1260 30/11/2019 10:18:50 Page 1 of 2

Supplementary Figure 176. HPLC data of **6p**

Data File F:\DATA\JFENG\DATA\FJ-11-89-rac(AD,20%,1.0).D
Sample Name: FJ-11-89-rac(AD,20%,1.0)

===

Acq. Operator :
Sample Operator :
Acq. Instrument : LC1260 Location : 1
Injection Date : 19/11/2019 12:46:43
Inj Volume : Manually
Acq. Method : F:\METHOD\JFeng.M
Last changed : 19/11/2019 12:39:30 by
(modified after loading)
Analysis Method : F:\METHOD\ZK.M
Last changed : 14/07/2019 15:34:15 by
Additional Info : Peak(s) manually integrated

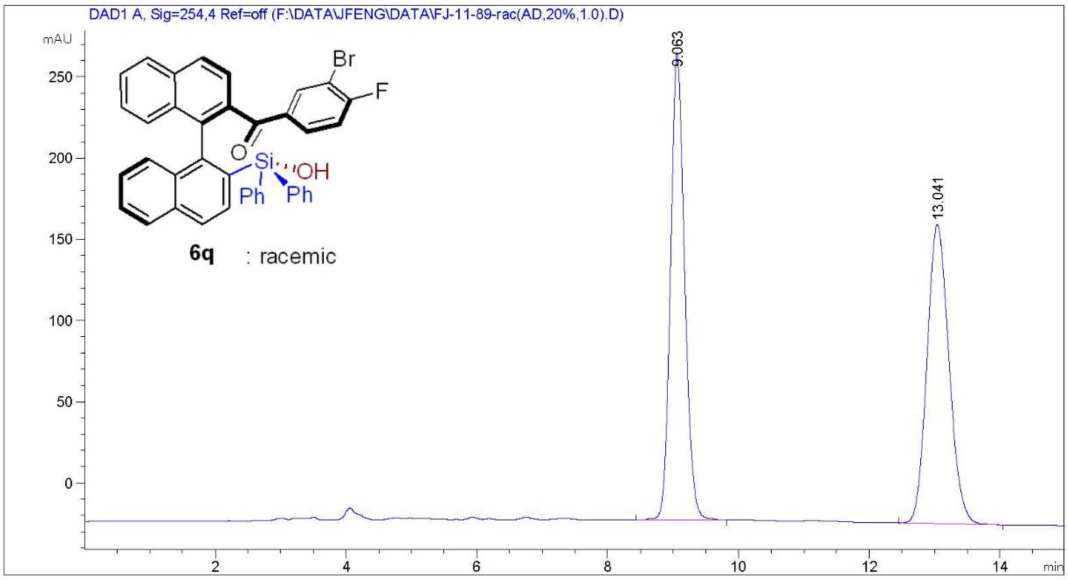

===

Area Percent Report

===

Sorted By : Signal
Multiplier : 1.0000
Dilution : 1.0000
Use Multiplier & Dilution Factor with ISTDs

Signal 1: DAD1 A, Sig=254,4 Ref=off

Peak #	RetTime [min]	Type	Width [min]	Area [mAU*s]	Height [mAU]	Area %
1	9.063	BB	0.2313	4314.06885	287.03632	50.1311
2	13.041	BB	0.3617	4291.50488	184.24858	49.8689

Totals : 8605.57373 471.28490

Supplementary Figure 177. HPLC data of **6q**

Data File F:\DATA\JFENG\DATA\FJ-11-88(AD,20%,1.0).D
Sample Name: FJ-11-88(AD,20%,1.0)

===

Acq. Operator :

Location : 1

Injection Date : 19/11/2019 13:04:23

Acq. Method : JFeng.M

Analysis Method : F:\METHOD\ZK.M

Last changed : 14/07/2019 15:34:15 by

Additional Info : Peak(s) manually integrated

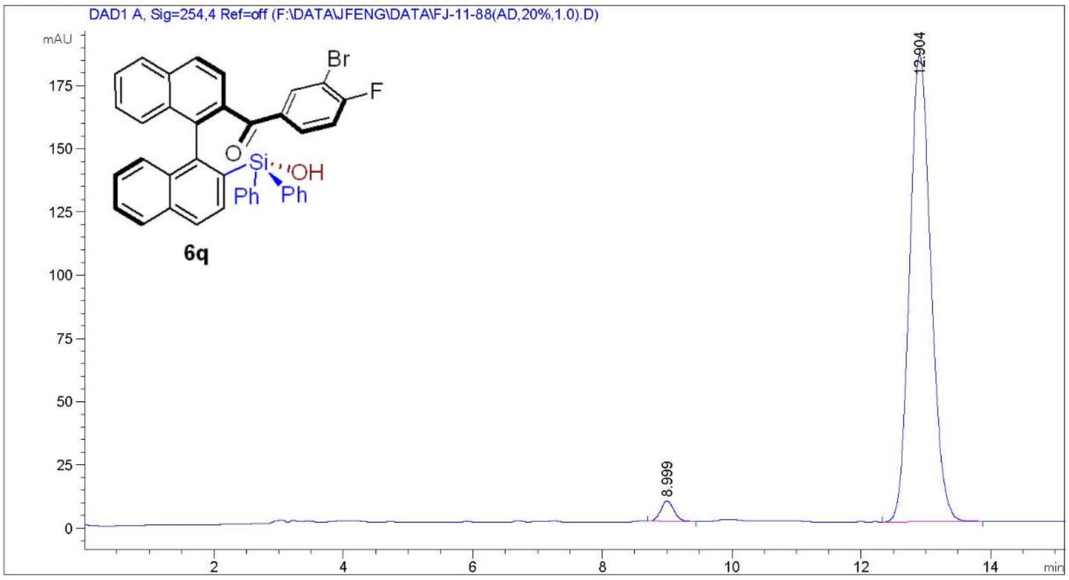

===

Area Percent Report

===

Sorted By : Signal

Multiplier : 1.0000

Dilution : 1.0000

Use Multiplier & Dilution Factor with ISTDs

Signal 1: DAD1 A, Sig=254,4 Ref=off

Peak #	RetTime [min]	Type	Width [min]	Area [mAU*s]	Height [mAU]	Area %
1	8.999	BB	0.2216	114.54284	7.97432	2.6437
2	12.904	BB	0.3544	4218.12646	184.70149	97.3563

Totals : 4332.66930 192.67582

===

*** End of Report ***

LC1260 30/11/2019 15:47:29 Page 1 of 1

Supplementary Figure 178. HPLC data of **6q**

Data File F:\DATA\JFENG\DATA\FJ-11-75(AD,20%,1.0).D
Sample Name: FJ-11-75(AD,20%,1.0)

===

Acq. Operator :
Sample Operator :
Acq. Instrument : LC1260 Location : 1
Injection Date : 14/11/2019 20:11:16
Inj Volume : No inj
Acq. Method : C:\Chem32\1\Methods\DEF_LC.M
Last changed : 14/11/2019 19:23:26 by
(modified after loading)
Analysis Method : F:\METHOD\ZK.M
Last changed : 14/07/2019 15:34:15 by
Additional Info : Peak(s) manually integrated

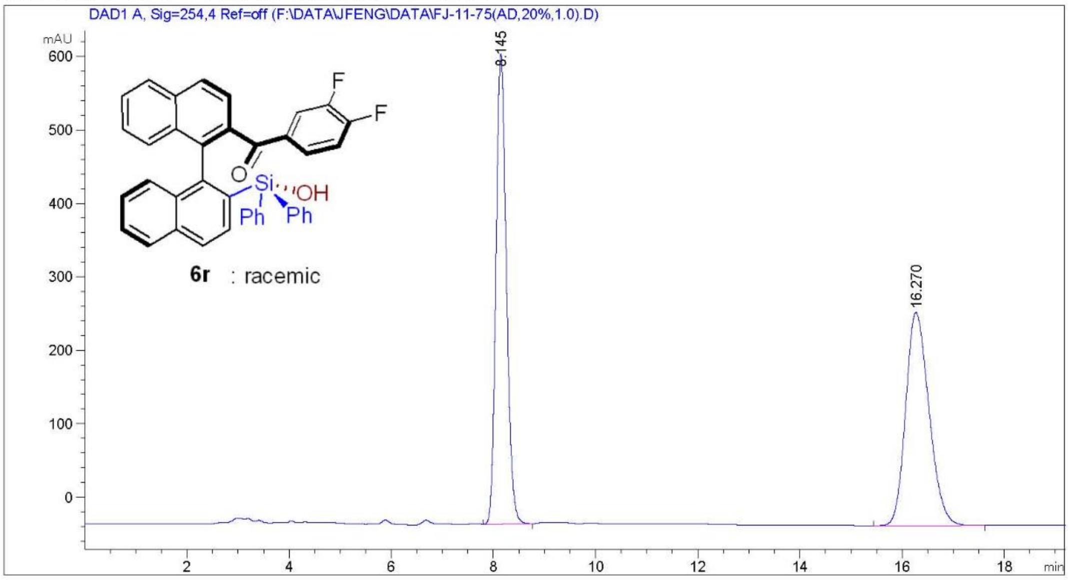

===

Area Percent Report

===

Sorted By : Signal
Multiplier : 1.0000
Dilution : 1.0000
Use Multiplier & Dilution Factor with ISTDs

Signal 1: DAD1 A, Sig=254,4 Ref=off

Peak #	RetTime [min]	Type	Width [min]	Area [mAU*s]	Height [mAU]	Area %
1	8.145	BB	0.2157	8976.04785	639.77362	49.4325
2	16.270	BB	0.4917	9182.15723	289.78668	50.5675

Totals : 1.81582e4 929.56030

LC1260 30/11/2019 15:38:30 Page 1 of 2

Supplementary Figure 179. HPLC data of **6r**

Data File F:\DATA\JFENG\DATA\FJ-11-74(AD,20%,1.0).D
Sample Name: FJ-11-74(AD,20%,1.0)

===

Acq. Operator :
Sample Operator :
Acq. Instrument : LC1260 Location : 1
Injection Date : 14/11/2019 19:50:00
Inj Volume : No inj
Acq. Method : C:\Chem32\1\Methods\DEF_LC.M
Last changed : 14/11/2019 19:23:26 by
(modified after loading)
Analysis Method : F:\METHOD\ZK.M
Last changed : 14/07/2019 15:34:15 by
Additional Info : Peak(s) manually integrated

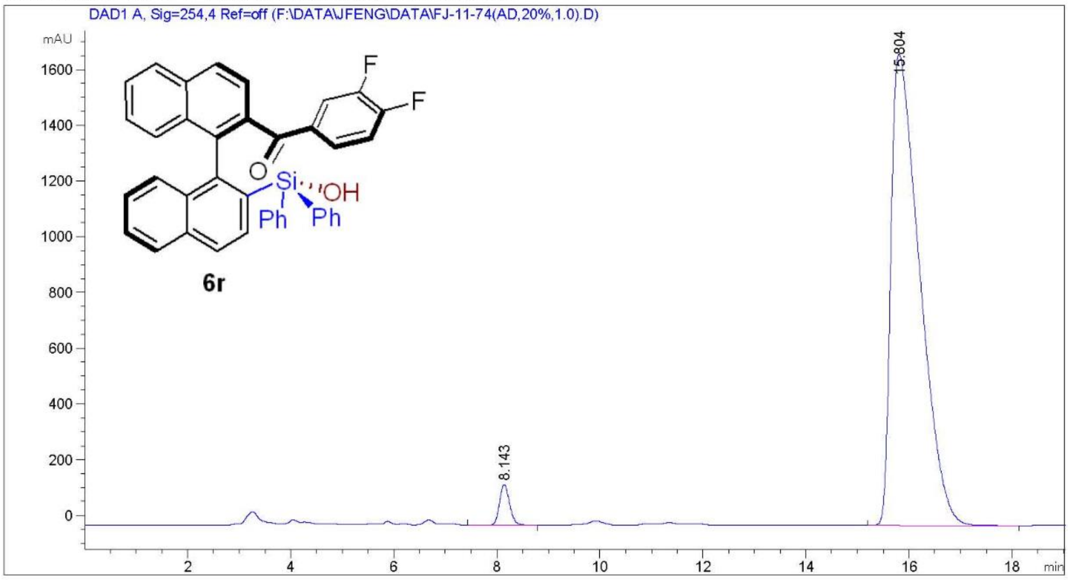

===

Area Percent Report

===

Sorted By : Signal
Multiplier : 1.0000
Dilution : 1.0000
Use Multiplier & Dilution Factor with ISTDs

Signal 1: DAD1 A, Sig=254,4 Ref=off

Peak #	RetTime [min]	Type	Width [min]	Area [mAU*s]	Height [mAU]	Area %
1	8.143	VB R	0.2103	1991.21021	145.64523	2.9667
2	15.804	BB	0.5932	6.51272e4	1691.38562	97.0333

Totals : 6.71184e4 1837.03085

Supplementary Figure 180. HPLC data of **6r**

Data File F:\DATA\JFENG\DATA\FJ-11-34-rac-1(AD,20%,1.0).D
Sample Name: FJ-11-34-rac-1(AD,20%,1.0)

===

Acq. Operator :
Sample Operator :
Acq. Instrument : LC1260 Location : 1
Injection Date : 20/10/2019 11:35:48
Inj Volume : No inj
Acq. Method : C:\Chem32\1\Methods\DEF_LC.M
Last changed : 20/10/2019 11:14:28 by
(modified after loading)
Analysis Method : F:\METHOD\ZK.M
Last changed : 14/07/2019 15:34:15 by
Additional Info : Peak(s) manually integrated

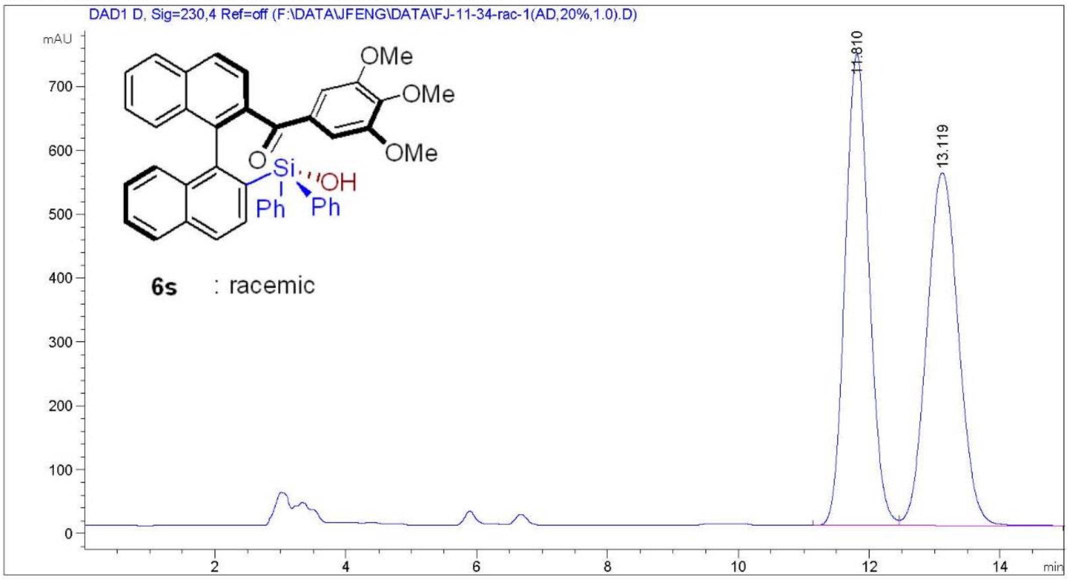

===

Area Percent Report

===

Sorted By : Signal
Multiplier : 1.0000
Dilution : 1.0000
Use Multiplier & Dilution Factor with ISTDs

Signal 1: DAD1 D, Sig=230,4 Ref=off

Peak #	RetTime [min]	Type	Width [min]	Area [mAU*s]	Height [mAU]	Area %
1	11.810	BV	0.3929	1.86599e4	738.26306	50.2036
2	13.119	VBA	0.5265	1.85086e4	552.90454	49.7964

Totals : 3.71685e4 1291.16760

LC1260 30/11/2019 15:24:09 Page 1 of 2

Supplementary Figure 181. HPLC data of **6s**

Data File F:\DATA\JFENG\DATA\FJ-11-33(AD,20%,1.0).D
Sample Name: FJ-11-33(AD,20%,1.0)

===

Acq. Operator :

Location : 1

Injection Date : 20/10/2019 11:55:00
Acq. Method : DEF_LC.M
Analysis Method : F:\METHOD\ZK.M
Last changed : 14/07/2019 15:34:15 by
Additional Info : Peak(s) manually integrated

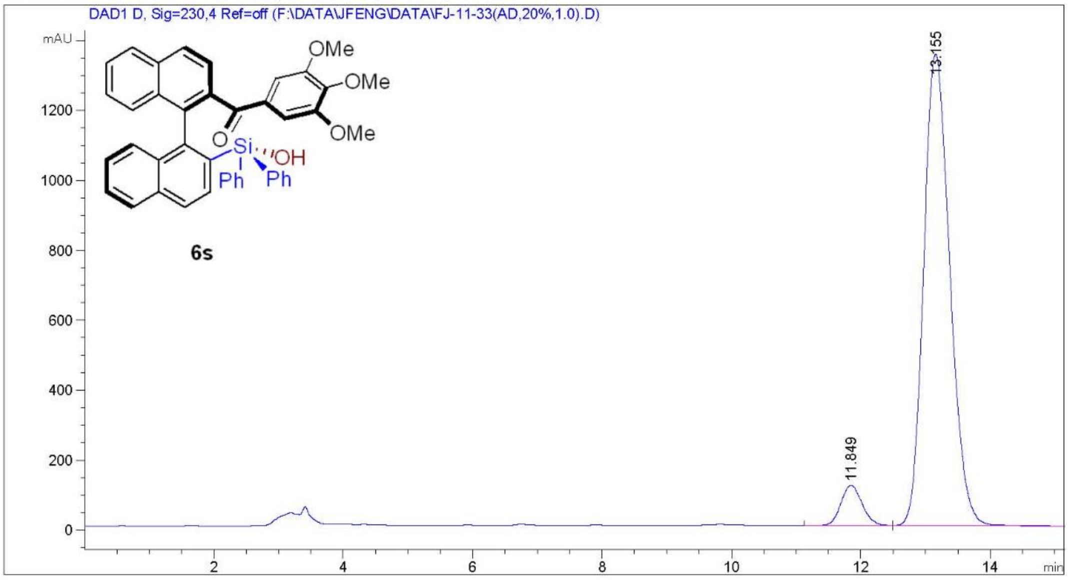

===

Area Percent Report

===

Sorted By : Signal
Multiplier : 1.0000
Dilution : 1.0000
Use Multiplier & Dilution Factor with ISTDs

Signal 1: DAD1 D, Sig=230,4 Ref=off

Peak #	RetTime [min]	Type	Width [min]	Area [mAU*s]	Height [mAU]	Area %
1	11.849	BV	0.3525	2643.01733	115.72359	6.5381
2	13.155	VBA	0.4349	3.77820e4	1348.45288	93.4619

Totals : 4.04250e4 1464.17647

===

*** End of Report ***

LC1260 30/11/2019 15:23:08 Page 1 of 1

Supplementary Figure 182. HPLC data of **6s**

Data File F:\DATA\JFENG\DATA\SL-1-21-(AD,20%,1.0).D
Sample Name: SL-1-21-(AD,20%,1.0)

===
Acq. Operator :
Location : 1
Injection Date : 06/11/2019 09:24:35
Acq. Method : DEF_LC.M
Analysis Method : F:\METHOD\ZK.M
Last changed : 14/07/2019 15:34:15 by
Additional Info : Peak(s) manually integrated

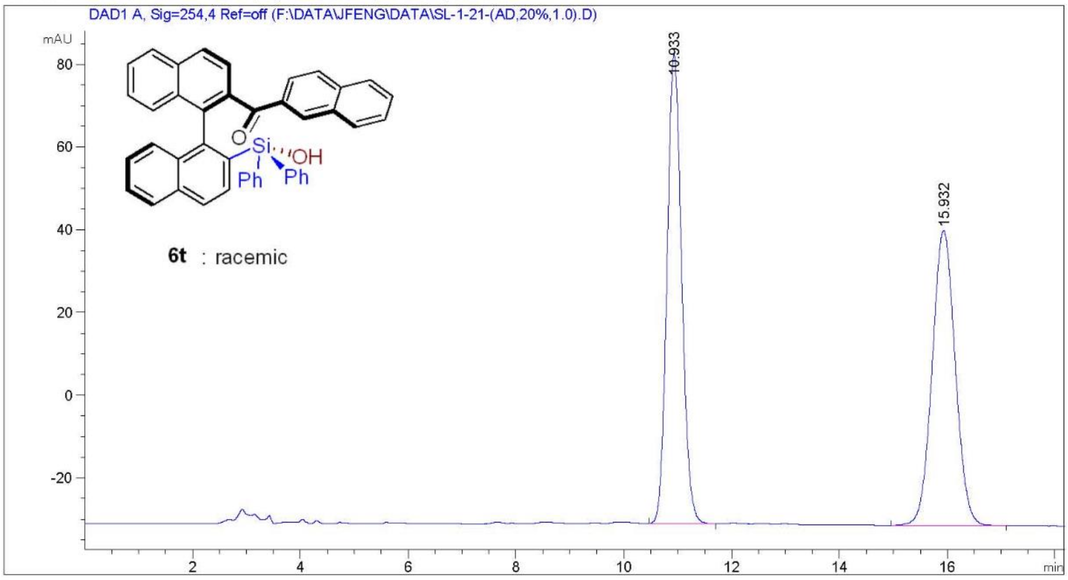

===
Area Percent Report
===

Sorted By : Signal
Multiplier : 1.0000
Dilution : 1.0000
Use Multiplier & Dilution Factor with ISTDs

Signal 1: DAD1 A, Sig=254,4 Ref=off

Peak #	RetTime [min]	Type	Width [min]	Area [mAU*s]	Height [mAU]	Area %
1	10.933	BB	0.2913	2131.81250	113.39069	50.1496
2	15.932	BB	0.4606	2119.09131	71.33137	49.8504

Totals : 4250.90381 184.72206

===
*** End of Report ***

LC1260 30/11/2019 15:31:59 Page 1 of 1

Supplementary Figure 183. HPLC data of **6t**

Data File F:\DATA\JFENG\DATA\FJ-11-60-(AD,20%,1.0).D
Sample Name: FJ-11-60-(AD,20%,1.0)

===

Acq. Operator :
Sample Operator :
Acq. Instrument : LC1260 Location : 1
Injection Date : 06/11/2019 09:45:46
Inj Volume : No inj
Acq. Method : C:\Chem32\1\Methods\DEF_LC.M
Last changed : 06/11/2019 09:17:52 by
(modified after loading)
Analysis Method : F:\METHOD\ZK.M
Last changed : 14/07/2019 15:34:15 by
Additional Info : Peak(s) manually integrated

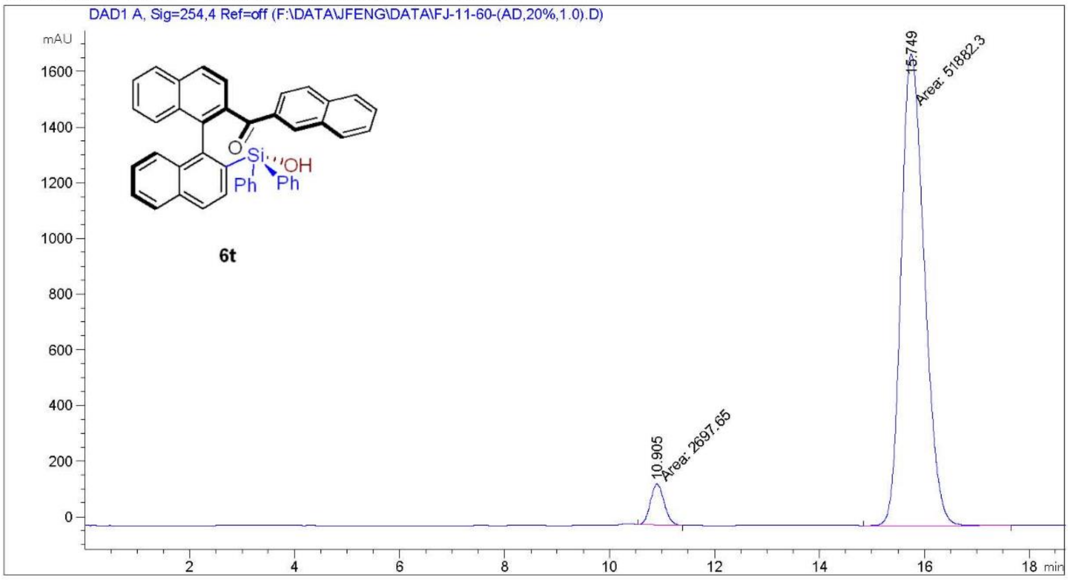

===

Area Percent Report

===

Sorted By : Signal
Multiplier : 1.0000
Dilution : 1.0000
Use Multiplier & Dilution Factor with ISTDs

Signal 1: DAD1 A, Sig=254,4 Ref=off

Peak #	RetTime [min]	Type	Width [min]	Area [mAU*s]	Height [mAU]	Area %
1	10.905	MM	0.3053	2697.65015	147.25496	4.9426
2	15.749	MM	0.5103	5.18823e4	1694.41992	95.0574

Totals : 5.45800e4 1841.67488

Supplementary Figure 184. HPLC data of **6t**

Data File F:\DATA\JFENG\DATA\FJ-11-70-rac-(AD,20%,1.0).D
Sample Name: FJ-11-70-rac-(AD,20%,1.0)

===

Acq. Operator :
Sample Operator :
Acq. Instrument : LC1260 Location : 1
Injection Date : 10/11/2019 16:06:26
Inj Volume : Manually
Acq. Method : F:\METHOD\JFeng.M
Last changed : 10/11/2019 15:53:26 by
(modified after loading)
Analysis Method : F:\METHOD\ZK.M
Last changed : 14/07/2019 15:34:15 by
Additional Info : Peak(s) manually integrated

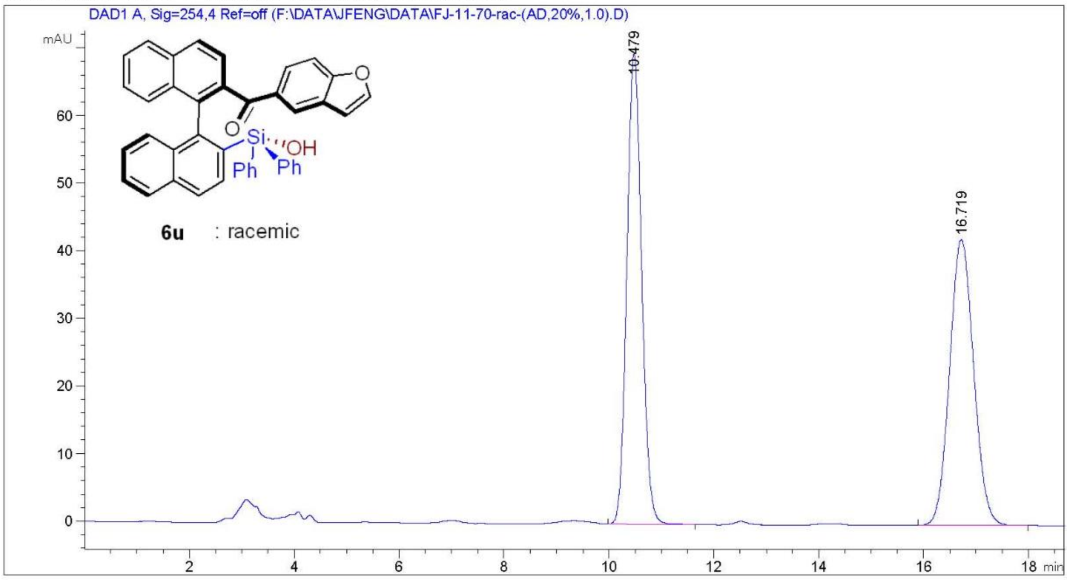

===

Area Percent Report

===

Sorted By : Signal
Multiplier : 1.0000
Dilution : 1.0000
Use Multiplier & Dilution Factor with ISTDs

Signal 1: DAD1 A, Sig=254,4 Ref=off

Peak #	RetTime [min]	Type	Width [min]	Area [mAU*s]	Height [mAU]	Area %
1	10.479	BB	0.3047	1363.57373	69.54488	50.3445
2	16.719	BB	0.4910	1344.91174	42.29040	49.6555

Totals : 2708.48547 111.83528

LC1260 30/11/2019 15:35:28 Page 1 of 2

Supplementary Figure 185. HPLC data of **6u**

Data File F:\DATA\JFENG\DATA\FJ-11-69(AD,20%,1.0).D
Sample Name: FJ-11-69(AD,20%,1.0)

===

Acq. Operator :

Location : 1

Injection Date : 10/11/2019 16:27:31

Acq. Method : JFeng.M

Analysis Method : F:\METHOD\ZK.M

Last changed : 14/07/2019 15:34:15 by

Additional Info : Peak(s) manually integrated

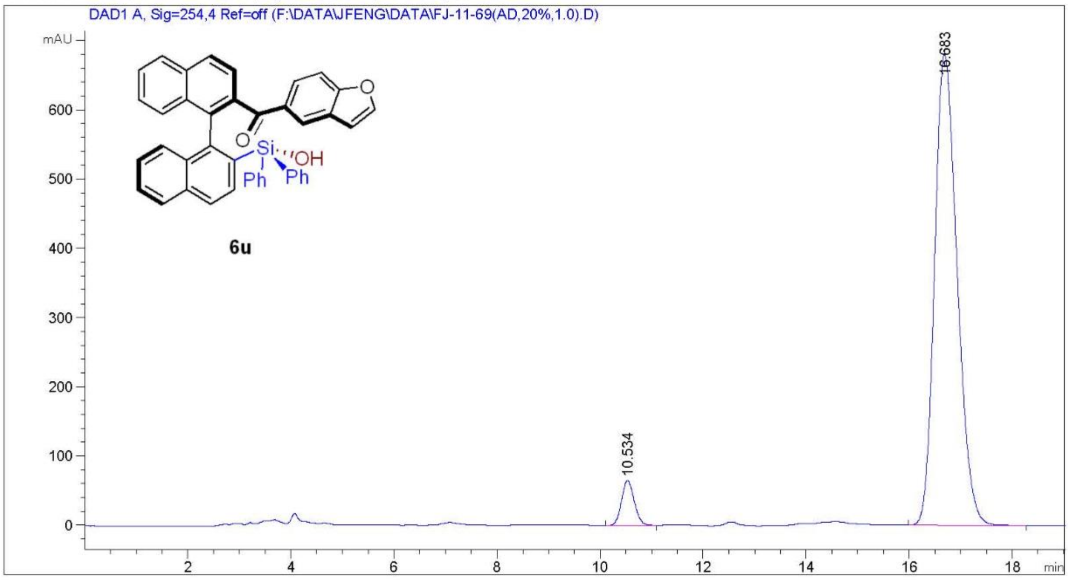

===

Area Percent Report

===

Sorted By : Signal

Multiplier : 1.0000

Dilution : 1.0000

Use Multiplier & Dilution Factor with ISTDs

Signal 1: DAD1 A, Sig=254,4 Ref=off

Peak #	RetTime [min]	Type	Width [min]	Area [mAU*s]	Height [mAU]	Area %
1	10.534	BB	0.2671	1123.68115	65.19385	5.1705
2	16.683	BB	0.4719	2.06087e4	679.31561	94.8295

Totals : 2.17324e4 744.50946

===

*** End of Report ***

LC1260 30/11/2019 15:37:06 Page 1 of 1

Supplementary Figure 186. HPLC data of **6u**

Data File F:\DATA\JFENG\DATA\BXF-1-114-rac(AD,8%,1.0).D
Sample Name: BXF-1-114-rac(AD,8%,1.0)

===

Acq. Operator :

Location : 1

Injection Date : 20/04/2019 21:19:28
Acq. Method : DEF_LC.M
Analysis Method : F:\METHOD\ZK.M
Last changed : 14/07/2019 15:34:15 by
Additional Info : Peak(s) manually integrated

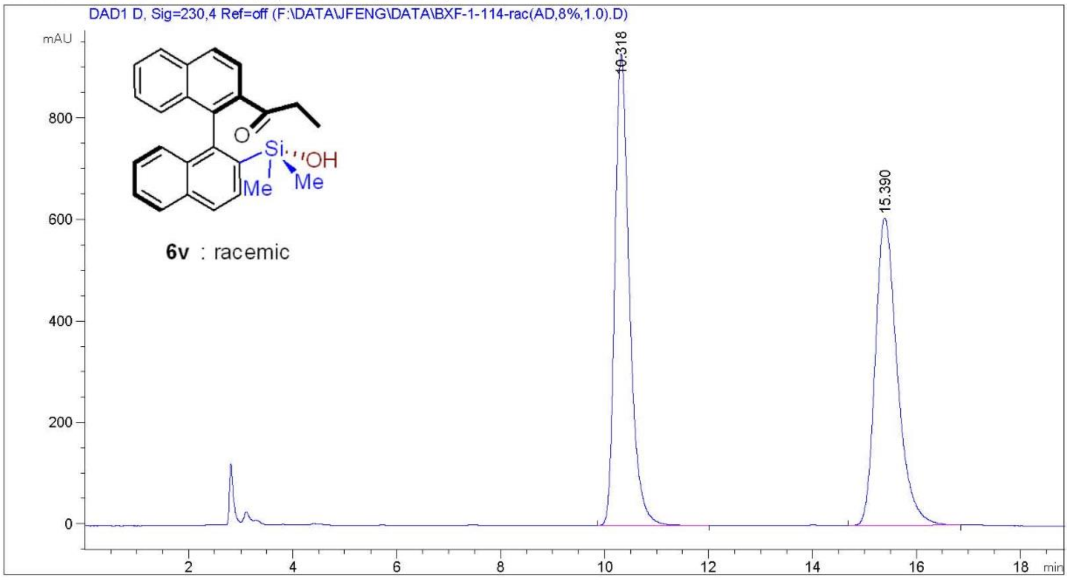

===

Area Percent Report

===

Sorted By : Signal
Multiplier : 1.0000
Dilution : 1.0000
Use Multiplier & Dilution Factor with ISTDs

Signal 1: DAD1 D, Sig=230,4 Ref=off

Peak #	RetTime [min]	Type	Width [min]	Area [mAU*s]	Height [mAU]	Area %
1	10.318	BB	0.2922	1.80134e4	929.00806	50.3522
2	15.390	BB	0.4500	1.77614e4	605.96783	49.6478

Totals : 3.57748e4 1534.97589

===

*** End of Report ***

LC1260 30/11/2019 16:22:56 Page 1 of 1

Supplementary Figure 187. HPLC data of **6v**

Data File F:\DATA\JFENG\DATA\FJ-10-83(AD,8%,1.0).D
Sample Name: FJ-10-83(AD,8%,1.0)

===

Acq. Operator :
Sample Operator :
Acq. Instrument : LC1260 Location : 1
Injection Date : 07/06/2019 20:50:03
Inj Volume : Manually
Acq. Method : F:\METHOD\JFeng.M
Last changed : 07/06/2019 20:44:23 by
(modified after loading)
Analysis Method : F:\METHOD\ZK.M
Last changed : 14/07/2019 15:34:15 by
Additional Info : Peak(s) manually integrated

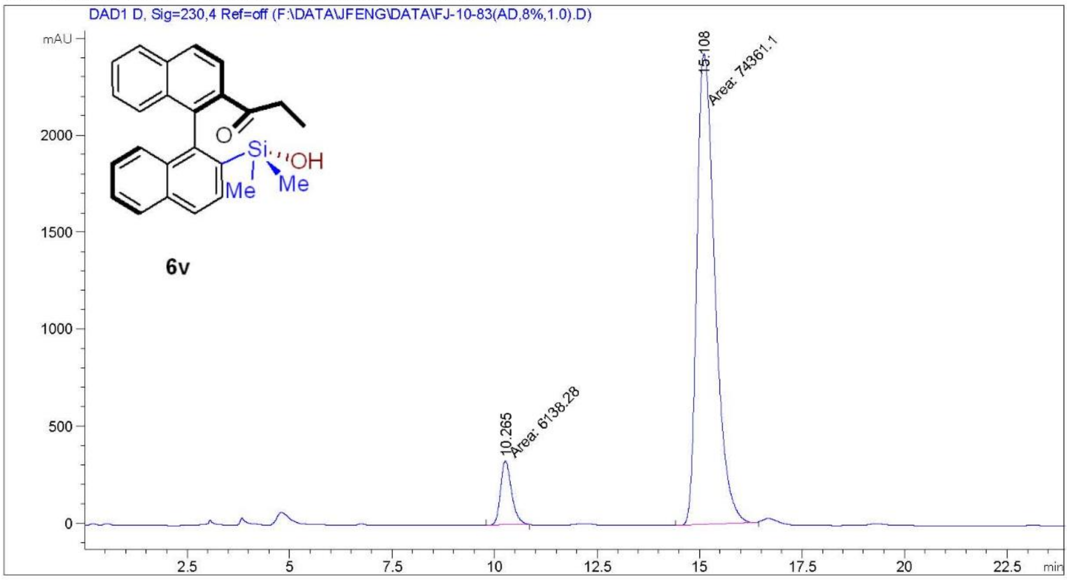

===

Area Percent Report

===

Sorted By : Signal
Multiplier : 1.0000
Dilution : 1.0000
Use Multiplier & Dilution Factor with ISTDs

Signal 1: DAD1 D, Sig=230,4 Ref=off

Peak #	RetTime [min]	Type	Width [min]	Area [mAU*s]	Height [mAU]	Area %
1	10.265	MM	0.3095	6138.28027	330.56366	7.6253
2	15.108	MM	0.5110	7.43611e4	2425.25513	92.3747

Totals : 8.04994e4 2755.81879

Supplementary Figure 188. HPLC data of **6v**

Data File F:\DATA\JFENG\DATA\FJ-12-157-2-rac-(AD,4%,1.0).D
Sample Name: FJ-12-157-2-rac-(AD,4%,1.0)

===

Acq. Operator :

Location : 1

Injection Date : 12/06/2020 09:34:46

Acq. Method : JFeng.M

Analysis Method : C:\Chem32\1\Methods\DEF_LC.M

Last changed : 13/02/2014 23:27:44 by SYSTEM

Additional Info : Peak(s) manually integrated

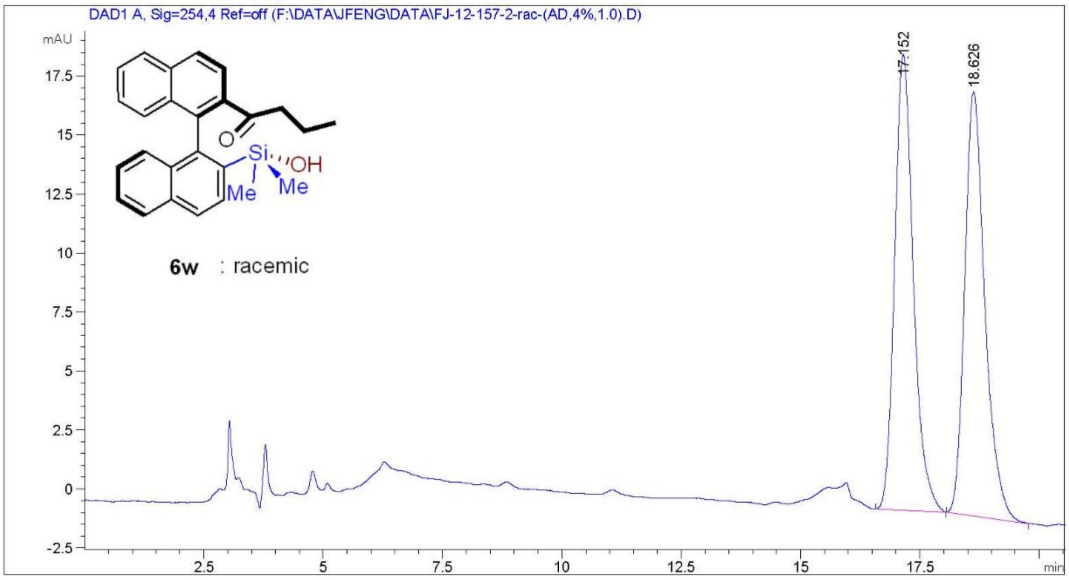

===

Area Percent Report

===

Sorted By : Signal

Multiplier : 1.0000

Dilution : 1.0000

Use Multiplier & Dilution Factor with ISTDs

Signal 1: DAD1 A, Sig=254,4 Ref=off

Peak #	RetTime [min]	Type	Width [min]	Area [mAU*s]	Height [mAU]	Area %
1	17.152	BB	0.4148	524.48938	19.31630	49.8754
2	18.626	BB	0.4422	527.11066	17.96947	50.1246

Totals : 1051.60004 37.28577

===

*** End of Report ***

LC1260 01/07/2020 21:11:42 Page 1 of 1

Supplementary Figure 189. HPLC data of **6w**

```
Data File F:\DATA\JFENG\DATA\FJ-12-161-1-(AD,4%,1.0).D
Sample Name: FJ-12-161-1-(AD,4%,1.0)

=====================================================================
Acq. Operator   :
                                                 Location :   1
Injection Date  : 12/06/2020 20:02:11
Acq. Method     : JFeng.M
Analysis Method : C:\Chem32\1\Methods\DEF_LC.M
Last changed    : 13/02/2014 23:27:44 by SYSTEM
Additional Info : Peak(s) manually integrated
```

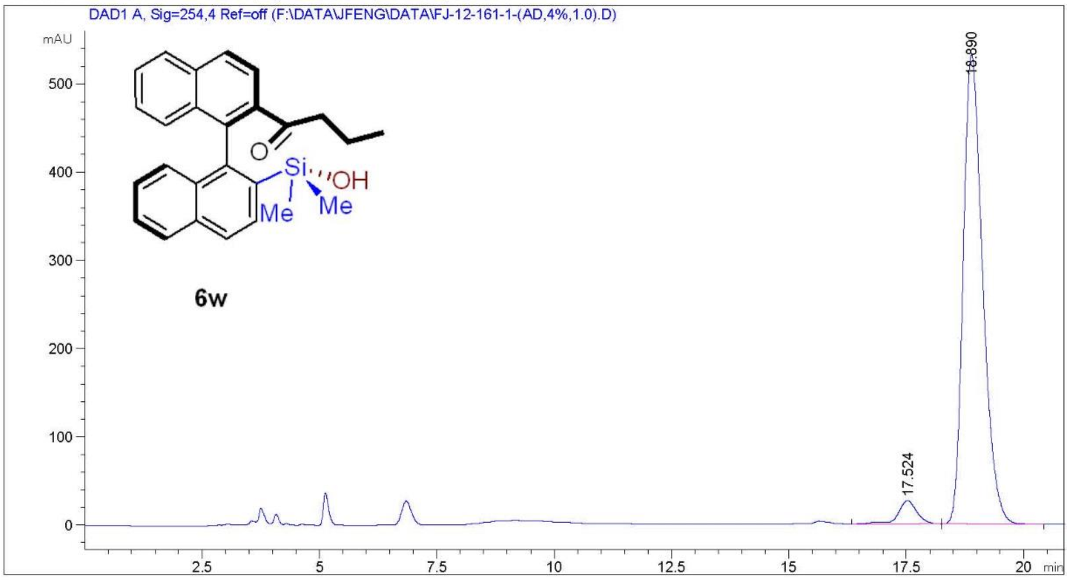


```
=====================================================================
                         Area Percent Report
=====================================================================

Sorted By             :      Signal
Multiplier            :      1.0000
Dilution              :      1.0000
Use Multiplier & Dilution Factor with ISTDs


Signal 1: DAD1 A, Sig=254,4 Ref=off
```

Peak #	RetTime [min]	Type	Width [min]	Area [mAU*s]	Height [mAU]	Area %
1	17.524	BB	0.4212	747.10229	26.47641	4.6546
2	18.890	BB	0.4435	1.53038e4	532.12524	95.3454

Totals : 1.60509e4 558.60165

```
=====================================================================
                         *** End of Report ***
```

LC1260 01/07/2020 21:12:29 Page 1 of 1

Supplementary Figure 190. HPLC data of **6w**

Data File F:\DATA\JFENG\DATA\FJ-10-191-RAC(AD,20%,1.0).D
Sample Name: FJ-10-191-RAC(AD,20%,1.0)

===

Acq. Operator :

Location : 1

Injection Date : 21/09/2019 17:24:02

Acq. Method : JFeng.M

Analysis Method : F:\METHOD\ZK.M

Last changed : 14/07/2019 15:34:15 by

Additional Info : Peak(s) manually integrated

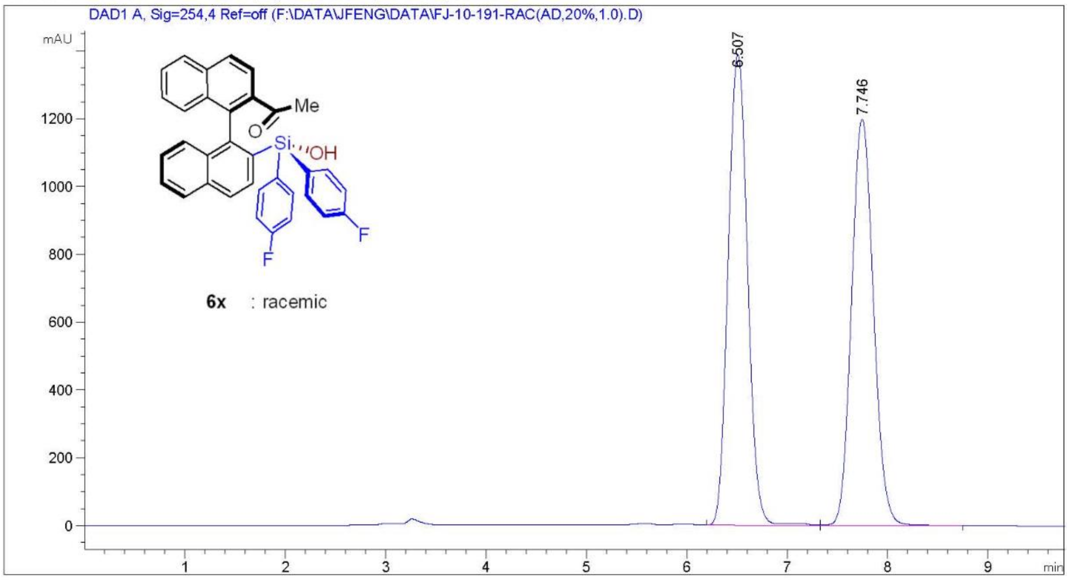

===

Area Percent Report

===

Sorted By : Signal

Multiplier : 1.0000

Dilution : 1.0000

Use Multiplier & Dilution Factor with ISTDs

Signal 1: DAD1 A, Sig=254,4 Ref=off

Peak #	RetTime [min]	Type	Width [min]	Area [mAU*s]	Height [mAU]	Area %
1	6.507	BV R	0.2134	1.85975e4	1388.39795	50.4683
2	7.746	VB	0.2396	1.82523e4	1198.97424	49.5317

Totals : 3.68499e4 2587.37219

===

*** End of Report ***

LC1260 30/11/2019 10:52:58 Page 1 of 1

Supplementary Figure 191. HPLC data of **6x**

Data File F:\DATA\JFENG\DATA\FJ-10-190(AD,20%,1.0).D
Sample Name: FJ-10-190(AD,20%,1.0)

===
Acq. Operator   :
Location :   1
Injection Date : 21/09/2019 17:35:46
Acq. Method   : JFeng.M
Analysis Method : F:\METHOD\ZK.M
Last changed   : 14/07/2019 15:34:15 by
Additional Info : Peak(s) manually integrated

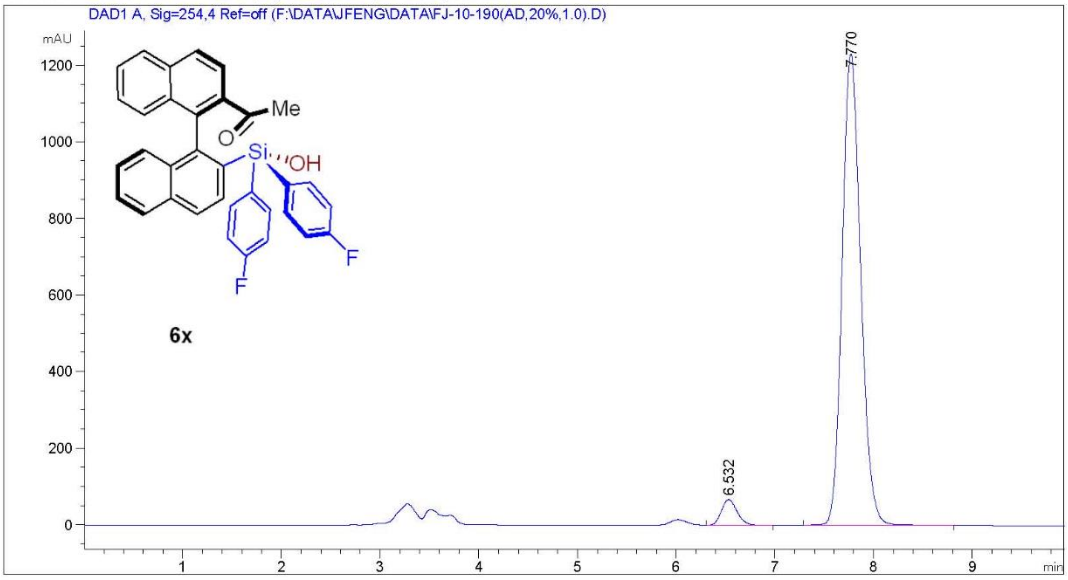

===
Area Percent Report
===

Sorted By : Signal
Multiplier : 1.0000
Dilution : 1.0000
Use Multiplier & Dilution Factor with ISTDs

Signal 1: DAD1 A, Sig=254,4 Ref=off

Peak #	RetTime [min]	Type	Width [min]	Area [mAU*s]	Height [mAU]	Area %
1	6.532	VB	0.1707	746.57880	67.67377	4.4558
2	7.770	BB	0.2018	1.60088e4	1230.35925	95.5442

Totals : 1.67553e4 1298.03302

===
*** End of Report ***

LC1260 30/11/2019 10:53:40 Page 1 of 1

Supplementary Figure 192. HPLC data of **6x**

Data File F:\DATA\JFENG\DATA\bxf-2-45-rac(AD,15%,1.0).D
Sample Name: bxf-2-45-rac(AD,15%,1.0)

===

Acq. Operator :
Sample Operator :
Acq. Instrument : LC1260 Location : 1
Injection Date : 19/09/2019 11:27:22
Inj Volume : No inj
Acq. Method : C:\Chem32\1\Methods\DEF_LC.M
Last changed : 19/09/2019 11:18:37 by
(modified after loading)
Analysis Method : F:\METHOD\ZK.M
Last changed : 14/07/2019 15:34:15 by

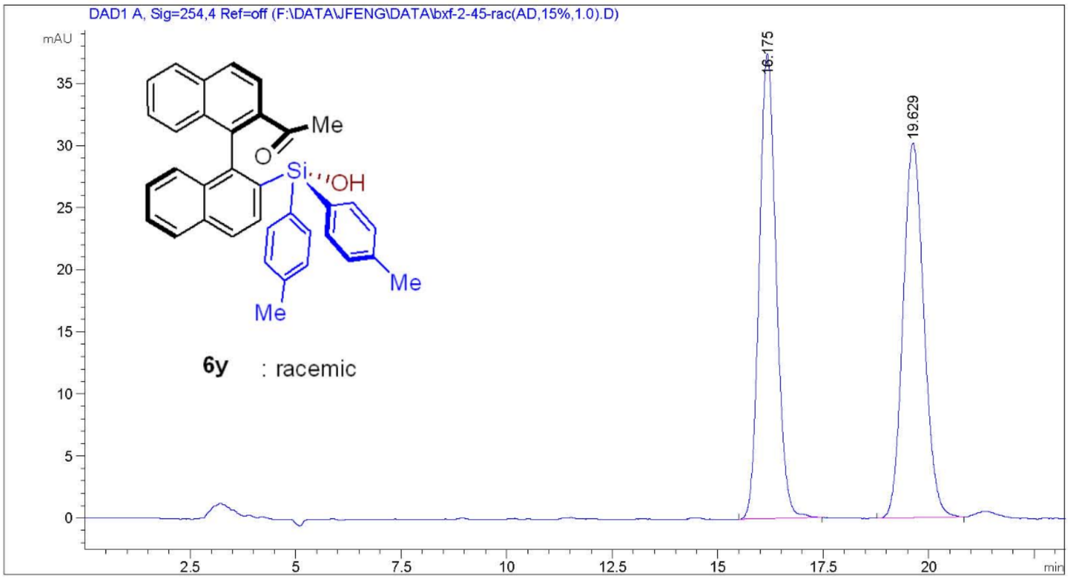

===

Area Percent Report

===

Sorted By : Signal
Multiplier : 1.0000
Dilution : 1.0000
Use Multiplier & Dilution Factor with ISTDs

Signal 1: DAD1 A, Sig=254,4 Ref=off

Peak #	RetTime [min]	Type	Width [min]	Area [mAU*s]	Height [mAU]	Area %
1	16.175	BB	0.4297	1044.59082	37.41811	50.2386
2	19.629	BB	0.5362	1034.66772	30.16058	49.7614

Totals : 2079.25854 67.57869

===

LC1260 30/11/2019 10:40:45 Page 1 of 2

Supplementary Figure 193. HPLC data of **6y**

Data File F:\DATA\JFENG\DATA\bxf-2-44(AD,15%,1.0).D
Sample Name: bxf-2-44(AD,15%,1.0)

===

Acq. Operator   :

Location :   1

Injection Date : 19/09/2019 11:55:04
Acq. Method   : DEF_LC.M
Analysis Method : F:\METHOD\ZK.M
Last changed   : 14/07/2019 15:34:15 by
Additional Info : Peak(s) manually integrated

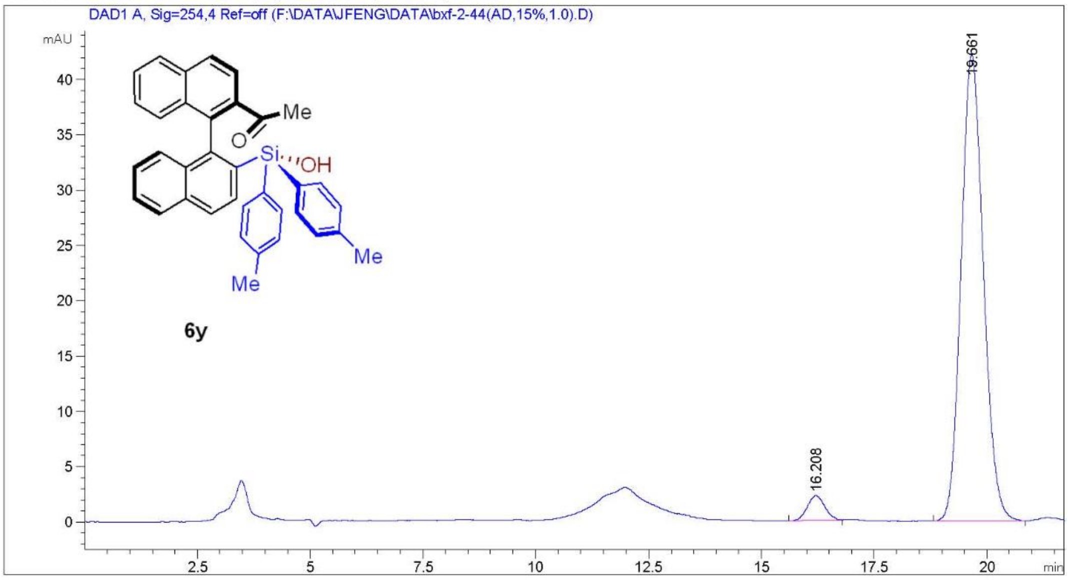

===

Area Percent Report

===

Sorted By : Signal
Multiplier : 1.0000
Dilution : 1.0000
Use Multiplier & Dilution Factor with ISTDs

Signal 1: DAD1 A, Sig=254,4 Ref=off

Peak #	RetTime [min]	Type	Width [min]	Area [mAU*s]	Height [mAU]	Area %
1	16.208	BB	0.4039	60.09991	2.23376	4.0020
2	19.661	BB	0.5250	1441.64819	42.14939	95.9980

Totals : 1501.74810 44.38315

===

*** End of Report ***

LC1260 30/11/2019 10:41:15 Page 1 of 1

Supplementary Figure 194. HPLC data of **6y**

```
Data File F:\DATA\JFENG\DATA\FJ-11-1-rac(AD,20%,1.0).D
Sample Name: FJ-11-1-rac(AD,20%,1.0)

=====================================================================
Acq. Operator   :
Sample Operator :
Acq. Instrument : LC1260                          Location :   1
Injection Date  : 26/09/2019 09:34:12
                                               Inj Volume : Manually
Acq. Method     : F:\METHOD\JFeng.M
Last changed    : 26/09/2019 09:25:34 by
                  (modified after loading)
Analysis Method : F:\METHOD\ZK.M
Last changed    : 14/07/2019 15:34:15 by
Additional Info : Peak(s) manually integrated
```

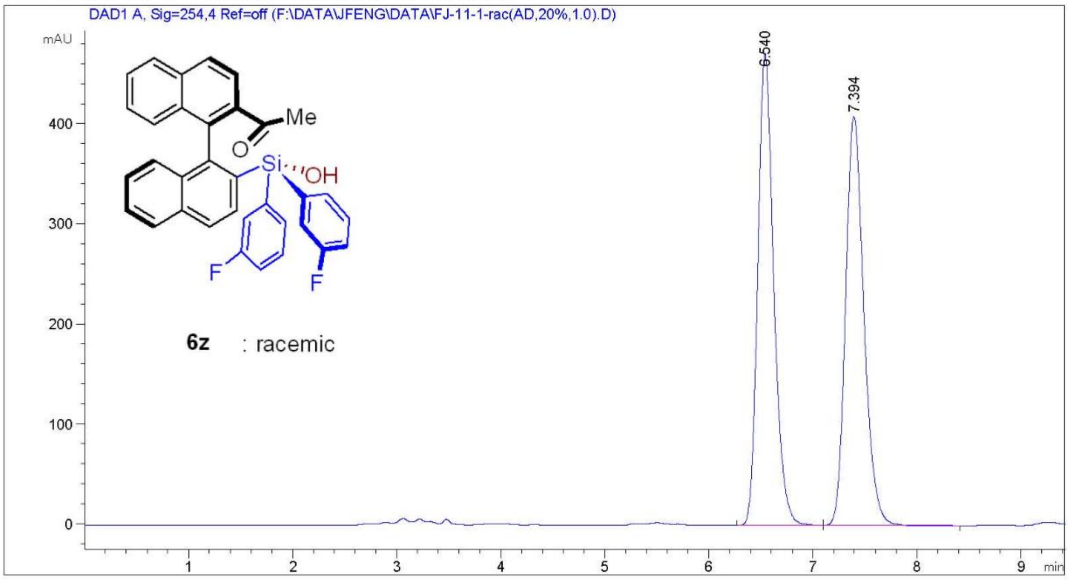


```
=====================================================================
                         Area Percent Report
=====================================================================

Sorted By             :      Signal
Multiplier            :      1.0000
Dilution              :      1.0000
Use Multiplier & Dilution Factor with ISTDs


Signal 1: DAD1 A, Sig=254,4 Ref=off

Peak RetTime Type  Width     Area      Height     Area
  #   [min]        [min]   [mAU*s]     [mAU]        %
----|-------|----|-------|----------|----------|--------|
   1   6.540 BB    0.1618 5002.84619  471.49576  50.2093
   2   7.394 BB    0.1857 4961.13232  408.45023  49.7907

Totals :                  9963.97852  879.94598
```


Supplementary Figure 195. HPLC data of **6z**

Data File F:\DATA\JFENG\DATA\FJ-10-200(AD,20%,1.0).D
Sample Name: FJ-10-200(AD,20%,1.0)

===

Acq. Operator :
Sample Operator :
Acq. Instrument : LC1260 Location : 1
Injection Date : 26/09/2019 09:46:10
Inj Volume : Manually
Acq. Method : F:\METHOD\JFeng.M
Last changed : 26/09/2019 09:25:34 by
(modified after loading)
Analysis Method : F:\METHOD\ZK.M
Last changed : 14/07/2019 15:34:15 by
Additional Info : Peak(s) manually integrated

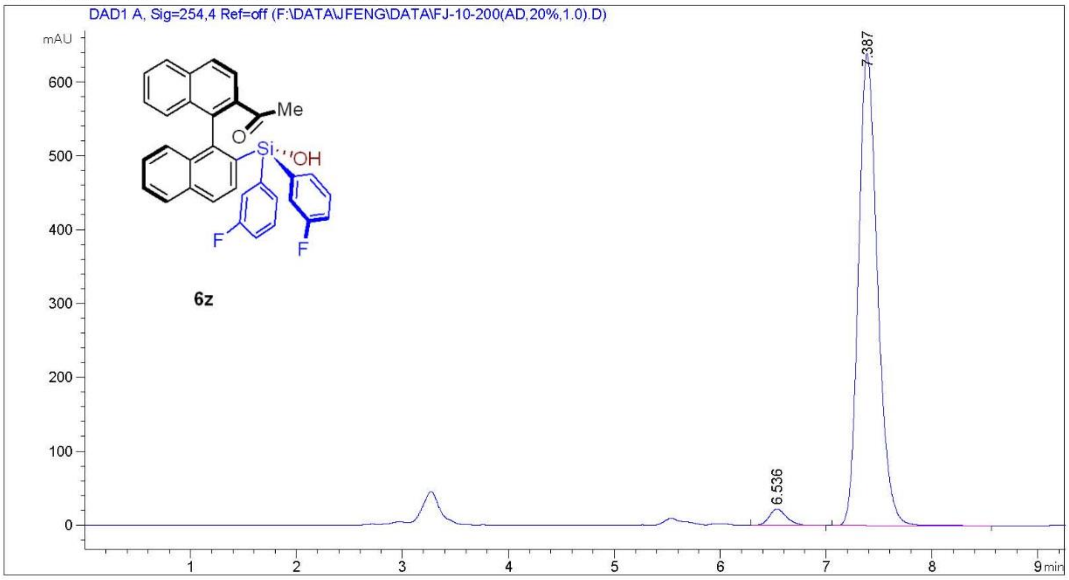

===

Area Percent Report

===

Sorted By : Signal
Multiplier : 1.0000
Dilution : 1.0000
Use Multiplier & Dilution Factor with ISTDs

Signal 1: DAD1 A, Sig=254,4 Ref=off

Peak #	RetTime [min]	Type	Width [min]	Area [mAU*s]	Height [mAU]	Area %
1	6.536	BB	0.1737	251.69478	22.29498	3.0533
2	7.387	BB	0.1919	7991.56152	638.95624	96.9467

Totals : 8243.25630 661.25121

LC1260 30/11/2019 10:56:27 Page 1 of 2

Supplementary Figure 196. HPLC data of **6z**

Data File F:\DATA\JFENG\DATA\FJ-11-3-rac(AD,20%,1.0).D
Sample Name: FJ-11-3-rac(AD,20%,1.0)

===

Acq. Operator :

Location : 1

Injection Date : 02/10/2019 09:38:27
Acq. Method : JFeng.M
Analysis Method : F:\METHOD\ZK.M
Last changed : 14/07/2019 15:34:15 by
Additional Info : Peak(s) manually integrated

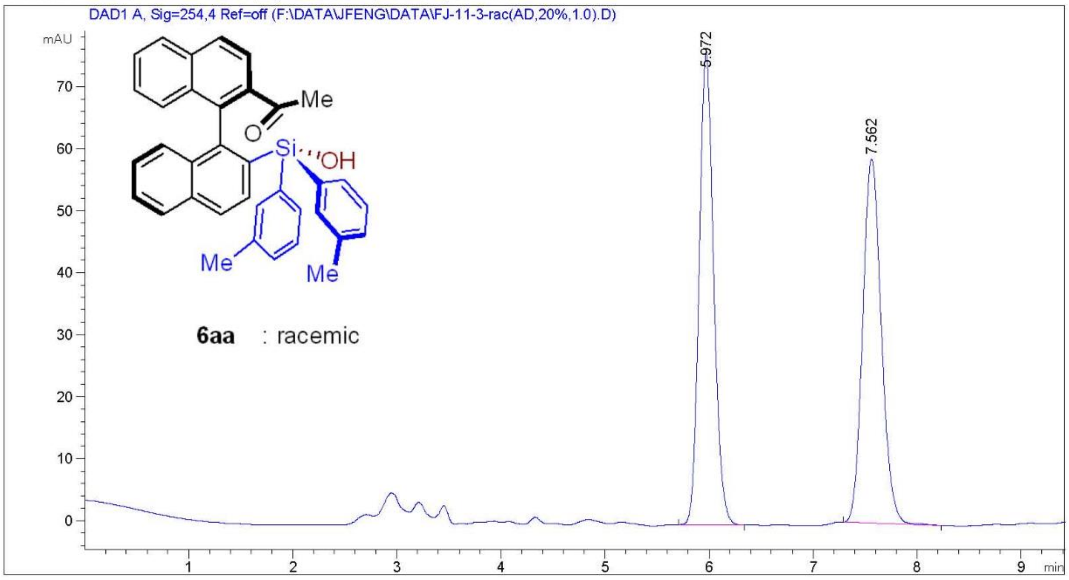

===

Area Percent Report

===

Sorted By : Signal
Multiplier : 1.0000
Dilution : 1.0000
Use Multiplier & Dilution Factor with ISTDs

Signal 1: DAD1 A, Sig=254,4 Ref=off

Peak #	RetTime [min]	Type	Width [min]	Area [mAU*s]	Height [mAU]	Area %
1	5.972	BB	0.1489	734.85309	75.92108	50.2229
2	7.562	BB	0.1906	728.33130	58.76814	49.7771

Totals : 1463.18439 134.68922

===

*** End of Report ***

LC1260 30/11/2019 14:56:15 Page 1 of 1

Supplementary Figure 197. HPLC data of **6aa**

Data File F:\DATA\JFENG\DATA\FJ-11-2(AD,20%,1.0).D
Sample Name: FJ-11-2(AD,20%,1.0)

===

Acq. Operator :
Sample Operator :
Acq. Instrument : LC1260 Location : 1
Injection Date : 02/10/2019 09:54:34
Inj Volume : Manually
Acq. Method : F:\METHOD\JFeng.M
Last changed : 02/10/2019 09:02:21 by
(modified after loading)
Analysis Method : F:\METHOD\ZK.M
Last changed : 14/07/2019 15:34:15 by
Additional Info : Peak(s) manually integrated

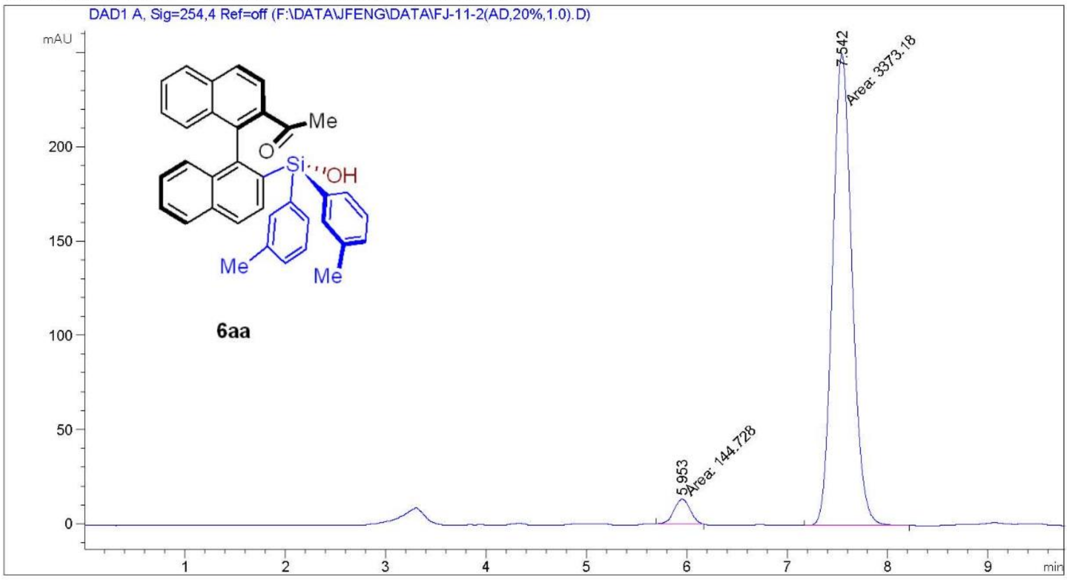

===

Area Percent Report

===

Sorted By : Signal
Multiplier : 1.0000
Dilution : 1.0000
Use Multiplier & Dilution Factor with ISTDs

Signal 1: DAD1 A, Sig=254,4 Ref=off

Peak #	RetTime [min]	Type	Width [min]	Area [mAU*s]	Height [mAU]	Area %
1	5.953	MM	0.1834	144.72801	13.15570	4.1140
2	7.542	MM	0.2246	3373.18140	250.26053	95.8860

Totals : 3517.90941 263.41623

LC1260 30/11/2019 14:59:34 Page 1 of 2

Supplementary Figure 198. HPLC data of **6aa**

Data File F:\DATA\JFENG\DATA\BXF-2-134(AD,20%,1.0).D
Sample Name: BXF-2-134(AD,20%,1.0)

===

Acq. Operator :

Location : 1

Injection Date : 11/11/2019 19:48:42
Acq. Method : DEF_LC.M
Analysis Method : F:\METHOD\ZK.M
Last changed : 14/07/2019 15:34:15 by
Additional Info : Peak(s) manually integrated

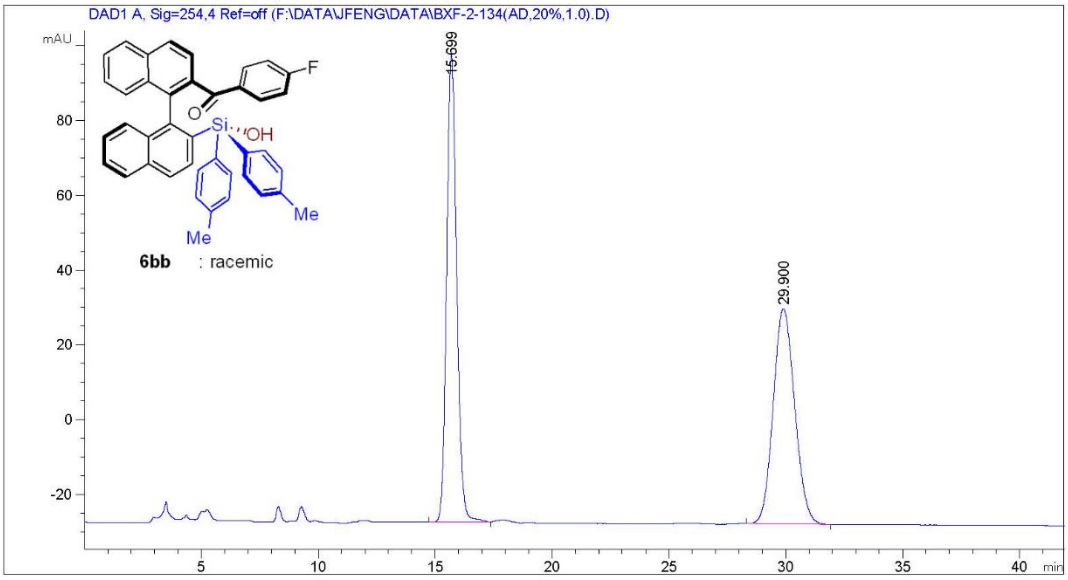

===
Area Percent Report
===

Sorted By : Signal
Multiplier : 1.0000
Dilution : 1.0000
Use Multiplier & Dilution Factor with ISTDs

Signal 1: DAD1 A, Sig=254,4 Ref=off

Peak #	RetTime [min]	Type	Width [min]	Area [mAU*s]	Height [mAU]	Area %
1	15.699	BB	0.4588	3717.13770	125.04779	50.3423
2	29.900	BB	0.9877	3666.59521	57.52039	49.6577

Totals : 7383.73291 182.56818

===
*** End of Report ***

LC1260 30/11/2019 10:38:41 Page 1 of 1

Supplementary Figure 199. HPLC data of **6bb**

Data File F:\DATA\JFENG\DATA\BXF-2-133(AD,20%,1.0).D
Sample Name: BXF-2-133(AD,20%,1.0)

===

Acq. Operator :
Sample Operator :
Acq. Instrument : LC1260 Location : 1
Injection Date : 11/11/2019 20:34:26
Inj Volume : No inj
Acq. Method : C:\Chem32\1\Methods\DEF_LC.M
Last changed : 11/11/2019 19:40:53 by
(modified after loading)
Analysis Method : F:\METHOD\ZK.M
Last changed : 14/07/2019 15:34:15 by
Additional Info : Peak(s) manually integrated

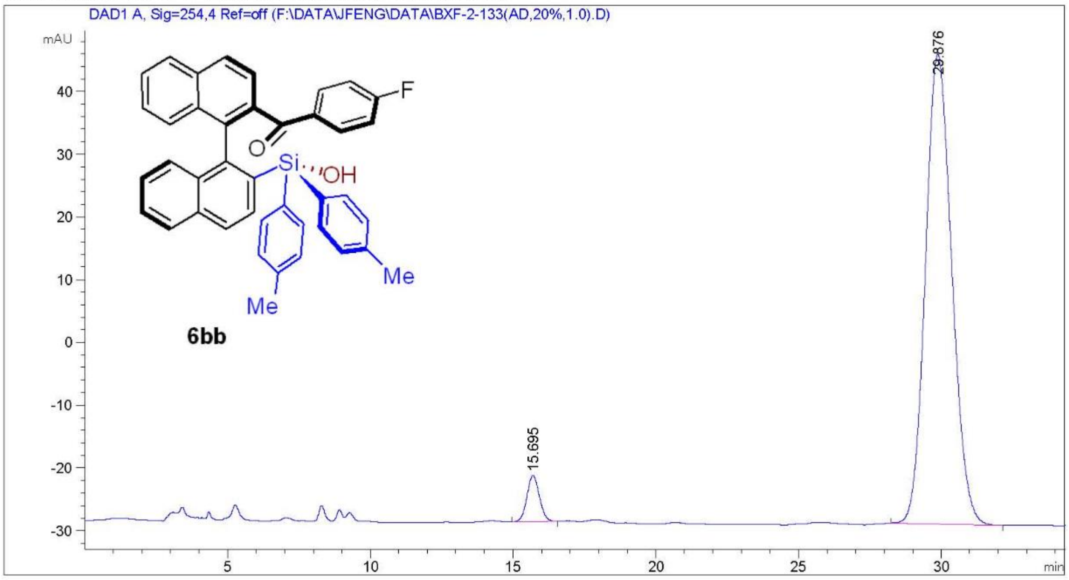

===

Area Percent Report

===

Sorted By : Signal
Multiplier : 1.0000
Dilution : 1.0000
Use Multiplier & Dilution Factor with ISTDs

Signal 1: DAD1 A, Sig=254,4 Ref=off

Peak #	RetTime [min]	Type	Width [min]	Area [mAU*s]	Height [mAU]	Area %
1	15.695	BB	0.4574	219.95226	7.34449	4.3885
2	29.876	BB	0.9784	4792.11133	74.90202	95.6115

Totals : 5012.06358 82.24650

LC1260 30/11/2019 10:39:21 Page 1 of 2

Supplementary Figure 200. HPLC data of **6bb**

Data File F:\DATA\JFENG\DATA\FJ-11-11-rac(AD,20%,1.0).D
Sample Name: FJ-11-11-rac(AD,20%,1.0)

===

Acq. Operator :
Sample Operator :
Acq. Instrument : LC1260 Location : 1
Injection Date : 02/10/2019 09:14:50
Inj Volume : Manually
Acq. Method : F:\METHOD\JFeng.M
Last changed : 02/10/2019 09:02:21 by
(modified after loading)
Analysis Method : F:\METHOD\ZK.M
Last changed : 14/07/2019 15:34:15 by
Additional Info : Peak(s) manually integrated

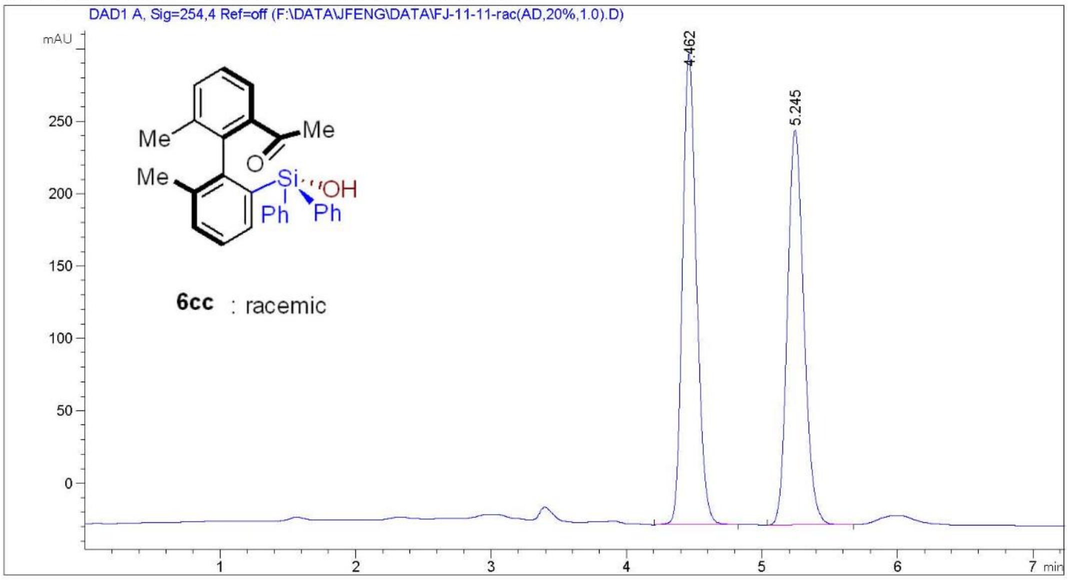

===

Area Percent Report

===

Sorted By : Signal
Multiplier : 1.0000
Dilution : 1.0000
Use Multiplier & Dilution Factor with ISTDs

Signal 1: DAD1 A, Sig=254,4 Ref=off

Peak #	RetTime [min]	Type	Width [min]	Area [mAU*s]	Height [mAU]	Area %
1	4.462	BB	0.1117	2335.01074	325.37936	50.2081
2	5.245	BB	0.1310	2315.65405	273.00372	49.7919

Totals : 4650.66479 598.38309

LC1260 30/11/2019 15:02:07 Page 1 of 2

Supplementary Figure 201. HPLC data of **6cc**

Data File F:\DATA\JFENG\DATA\FJ-11-10(AD,20%,1.0).D
Sample Name: FJ-11-10(AD,20%,1.0)

===
Acq. Operator :
Location : 1
Injection Date : 02/10/2019 09:26:55
Acq. Method : JFeng.M
Analysis Method : F:\METHOD\ZK.M
Last changed : 14/07/2019 15:34:15 by
Additional Info : Peak(s) manually integrated

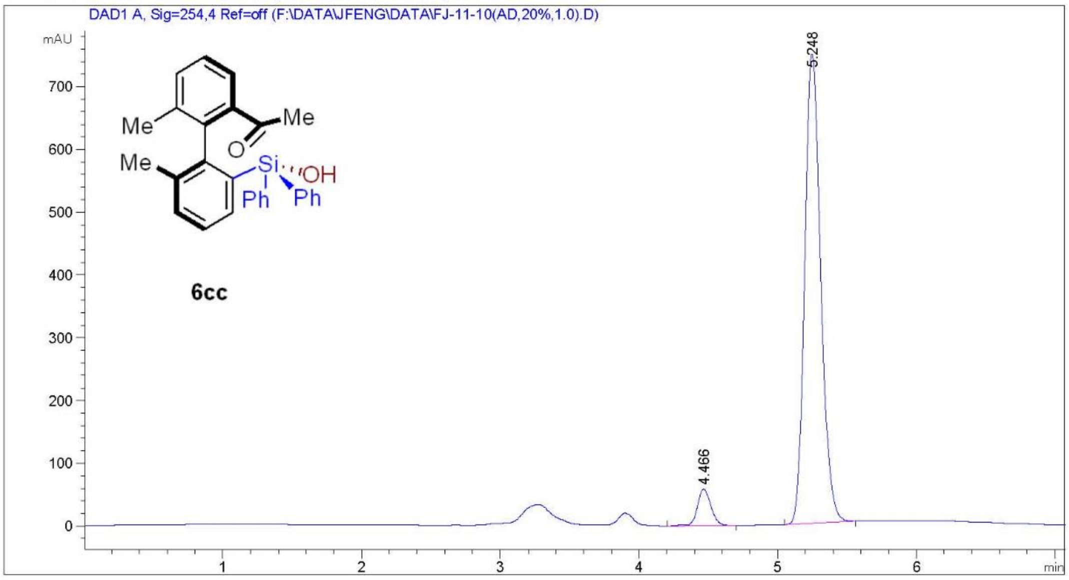

===
Area Percent Report
===

Sorted By : Signal
Multiplier : 1.0000
Dilution : 1.0000
Use Multiplier & Dilution Factor with ISTDs

Signal 1: DAD1 A, Sig=254,4 Ref=off

Peak #	RetTime [min]	Type	Width [min]	Area [mAU*s]	Height [mAU]	Area %
1	4.466	BB	0.1040	391.48196	58.51941	6.1529
2	5.248	BB	0.1231	5971.06201	748.19598	93.8471

Totals : 6362.54398 806.71540

===
*** End of Report ***

LC1260 30/11/2019 15:03:40 Page 1 of 1

Supplementary Figure 202. HPLC data of **6cc**

Data File F:\DATA\JFENG\DATA\BXF-2-139(AD,20%,1.0).D
Sample Name: BXF-2-139(AD,20%,1.0)

===

Acq. Operator :

Location : 1

Injection Date : 13/11/2019 20:24:19
Acq. Method : DEF_LC.M
Analysis Method : F:\METHOD\ZK.M
Last changed : 14/07/2019 15:34:15 by
Additional Info : Peak(s) manually integrated

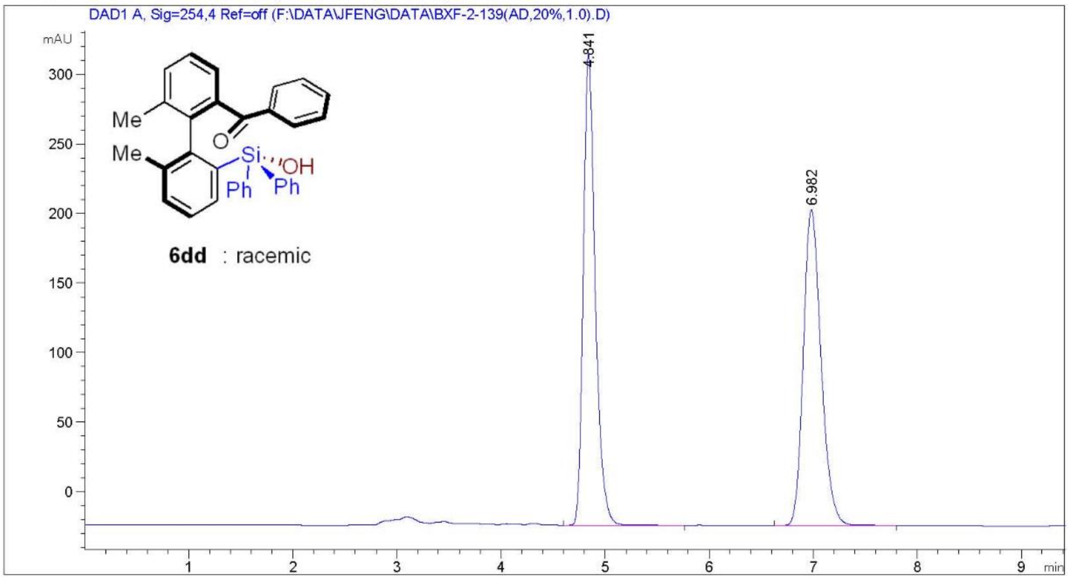

===

Area Percent Report

===

Sorted By : Signal
Multiplier : 1.0000
Dilution : 1.0000
Use Multiplier & Dilution Factor with ISTDs

Signal 1: DAD1 A, Sig=254,4 Ref=off

Peak #	RetTime [min]	Type	Width [min]	Area [mAU*s]	Height [mAU]	Area %
1	4.841	BB	0.1188	2694.25537	338.59076	50.0765
2	6.982	BB	0.1819	2686.01855	227.27237	49.9235

Totals : 5380.27393 565.86313

===

*** End of Report ***

LC1260 30/11/2019 10:26:09 Page 1 of 1

Supplementary Figure 203. HPLC data of **6dd**

Data File F:\DATA\JFENG\DATA\BXF-2-138-2(AD,20%,1.0).D
Sample Name: BXF-2-138-2(AD,20%,1.0)

===

Acq. Operator :
Sample Operator :
Acq. Instrument : LC1260 Location : 1
Injection Date : 14/11/2019 13:30:51
Inj Volume : No inj
Acq. Method : C:\Chem32\1\Methods\DEF_LC.M
Last changed : 14/11/2019 13:24:42 by
(modified after loading)
Analysis Method : F:\METHOD\ZK.M
Last changed : 14/07/2019 15:34:15 by
Additional Info : Peak(s) manually integrated

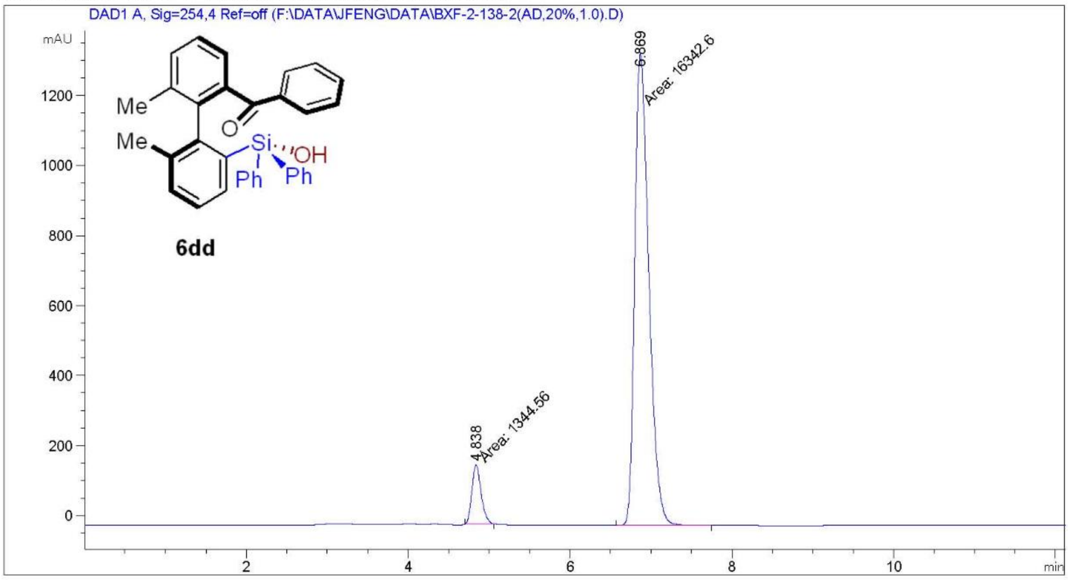

===
Area Percent Report
===

Sorted By : Signal
Multiplier : 1.0000
Dilution : 1.0000
Use Multiplier & Dilution Factor with ISTDs

Signal 1: DAD1 A, Sig=254,4 Ref=off

Peak #	RetTime [min]	Type	Width [min]	Area [mAU*s]	Height [mAU]	Area %
1	4.838	MM	0.1328	1344.55688	168.68443	7.6019
2	6.869	MM	0.2021	1.63426e4	1347.47070	92.3981
Totals :				1.76871e4	1516.15514	

LC1260 30/11/2019 10:29:12 Page 1 of 2

Supplementary Figure 204. HPLC data of **6dd**

Data File F:\DATA\JFENG\DATA\BXF-2-150(AD,20%,1.0).D
Sample Name: BXF-2-150(AD,20%,1.0)

===
Acq. Operator :
Location : 1
Injection Date : 19/11/2019 15:40:14
Acq. Method : DEF_LC.M
Analysis Method : F:\METHOD\ZK.M
Last changed : 14/07/2019 15:34:15 by
Additional Info : Peak(s) manually integrated

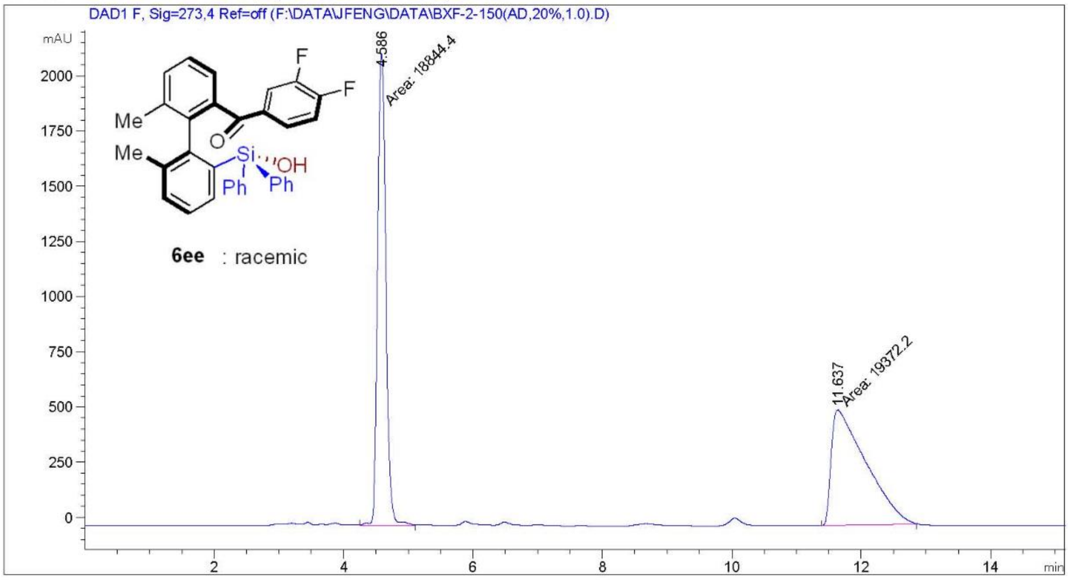

===
Area Percent Report
===

Sorted By : Signal
Multiplier : 1.0000
Dilution : 1.0000
Use Multiplier & Dilution Factor with ISTDs

Signal 1: DAD1 F, Sig=273,4 Ref=off

Peak #	RetTime [min]	Type	Width [min]	Area [mAU*s]	Height [mAU]	Area %
1	4.586	MM	0.1469	1.88444e4	2137.47729	49.3094
2	11.637	MM	0.6176	1.93722e4	522.74225	50.6906

Totals : 3.82166e4 2660.21954

===
*** End of Report ***

LC1260 30/11/2019 10:35:20 Page 1 of 1

Supplementary Figure 205. HPLC data of **6ee**

Data File F:\DATA\JFENG\DATA\BXF-2-149(AD,20%,1.0).D
Sample Name: BXF-2-149(AD,20%,1.0)

===
Acq. Operator :
Sample Operator :
Acq. Instrument : LC1260 Location : 1
Injection Date : 19/11/2019 16:01:08
Inj Volume : No inj
Acq. Method : C:\Chem32\1\Methods\DEF_LC.M
Last changed : 19/11/2019 15:33:49 by
(modified after loading)
Analysis Method : F:\METHOD\ZK.M
Last changed : 14/07/2019 15:34:15 by
Additional Info : Peak(s) manually integrated

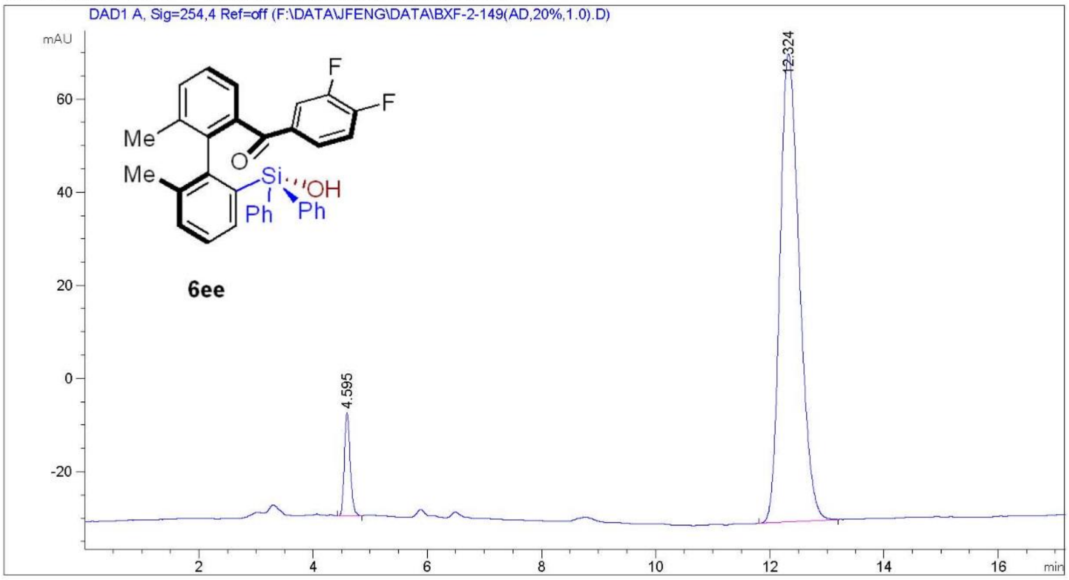

===
Area Percent Report
===

Sorted By : Signal
Multiplier : 1.0000
Dilution : 1.0000
Use Multiplier & Dilution Factor with ISTDs

Signal 1: DAD1 A, Sig=254,4 Ref=off

Peak #	RetTime [min]	Type	Width [min]	Area [mAU*s]	Height [mAU]	Area %
1	4.595	BB	0.1078	154.87248	22.10028	6.1456
2	12.324	BB	0.3647	2365.19214	100.43896	93.8544

Totals : 2520.06462 122.53924

Supplementary Figure 206. HPLC data of **6ee**

Data File F:\DATA\JFENG\DATA\SL-1-16(AD,20%,1.0).D
Sample Name: SL-1-16(AD,20%,1.0)

===

Acq. Operator :

Location : 1

Injection Date : 26/10/2019 16:22:16
Acq. Method : DEF_LC.M
Analysis Method : F:\METHOD\ZK.M
Last changed : 14/07/2019 15:34:15 by
Additional Info : Peak(s) manually integrated

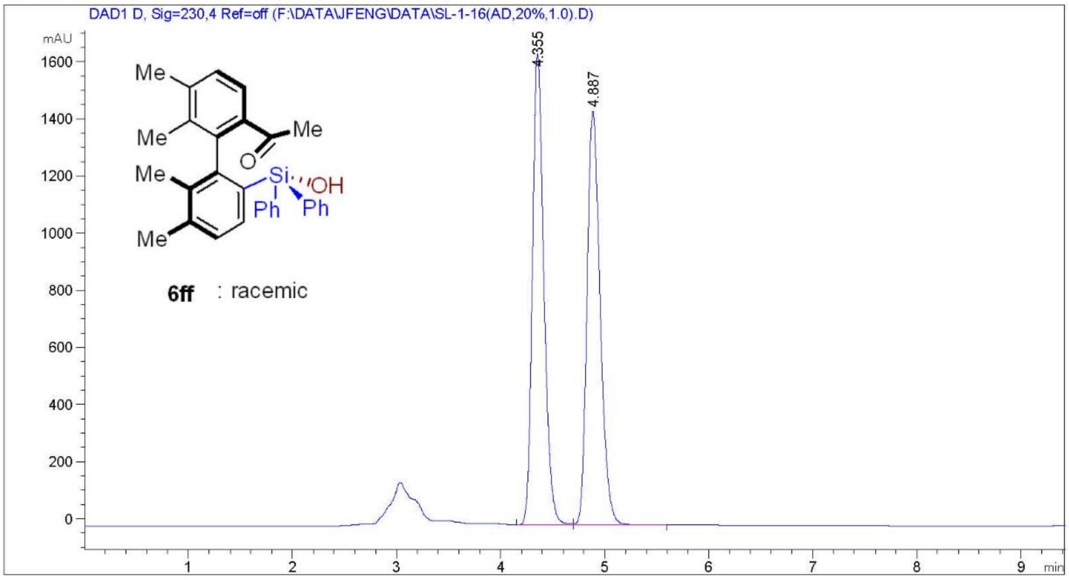

===

Area Percent Report

===

Sorted By : Signal
Multiplier : 1.0000
Dilution : 1.0000
Use Multiplier & Dilution Factor with ISTDs

Signal 1: DAD1 D, Sig=230,4 Ref=off

Peak #	RetTime [min]	Type	Width [min]	Area [mAU*s]	Height [mAU]	Area %
1	4.355	BV	0.1168	1.25693e4	1650.55920	49.7878
2	4.887	VB	0.1340	1.26764e4	1449.27954	50.2122

Totals : 2.52456e4 3099.83875

===

*** End of Report ***

LC1260 30/11/2019 15:28:27 Page 1 of 1

Supplementary Figure 207. HPLC data of **6ff**

Data File F:\DATA\JFENG\DATA\FJ-11-480(AD,20%,1.0).D
Sample Name: FJ-11-480(AD,20%,1.0)

===

Acq. Operator :
Sample Operator :
Acq. Instrument : LC1260 Location : 1
Injection Date : 26/10/2019 16:47:40
Inj Volume : No inj
Acq. Method : C:\Chem32\1\Methods\DEF_LC.M
Last changed : 26/10/2019 16:16:32 by
(modified after loading)
Analysis Method : F:\METHOD\ZK.M
Last changed : 14/07/2019 15:34:15 by
Additional Info : Peak(s) manually integrated

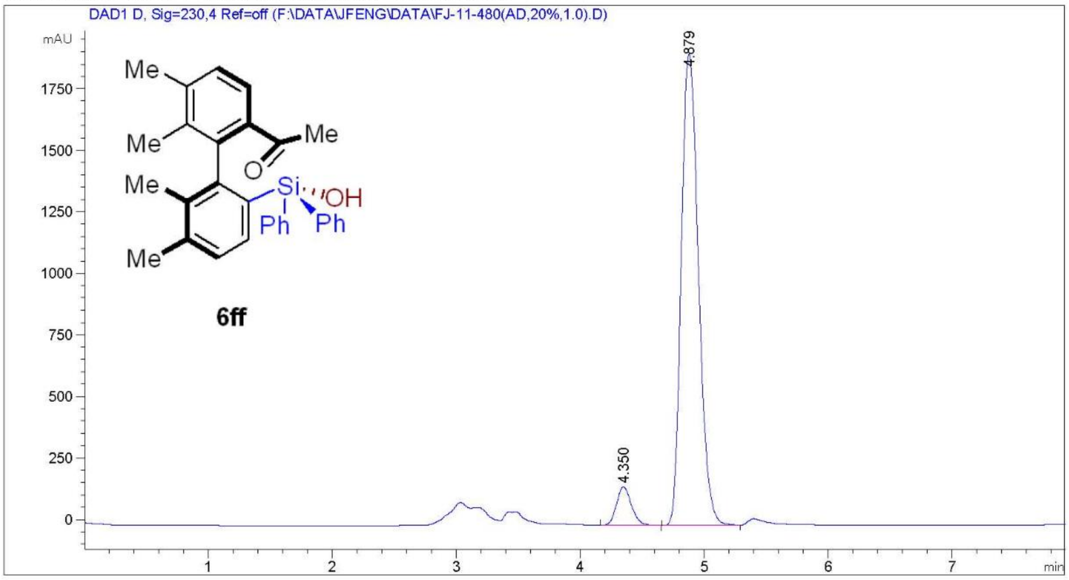

===
Area Percent Report
===

Sorted By : Signal
Multiplier : 1.0000
Dilution : 1.0000
Use Multiplier & Dilution Factor with ISTDs

Signal 1: DAD1 D, Sig=230,4 Ref=off

Peak #	RetTime [min]	Type	Width [min]	Area [mAU*s]	Height [mAU]	Area %
1	4.350	BB	0.1280	1282.92566	155.90511	6.5373
2	4.879	BB	0.1518	1.83418e4	1913.80811	93.4627

Totals : 1.96247e4 2069.71321

LC1260 30/11/2019 15:27:55 Page 1 of 2

Supplementary Figure 208. HPLC data of **6ff**

Data File F:\DATA\BXF\DATA\bxf-3-87(AD,90%,1.0).D
Sample Name: bxf-3-87(AD,90%,1.0)

===

Acq. Operator :

Location : 1

Injection Date : 05/06/2020 20:06:16
Acq. Method : DEF_LC.M
Analysis Method : C:\Chem32\1\Methods\DEF_LC.M
Last changed : 13/02/2014 23:27:44 by SYSTEM
Additional Info : Peak(s) manually integrated

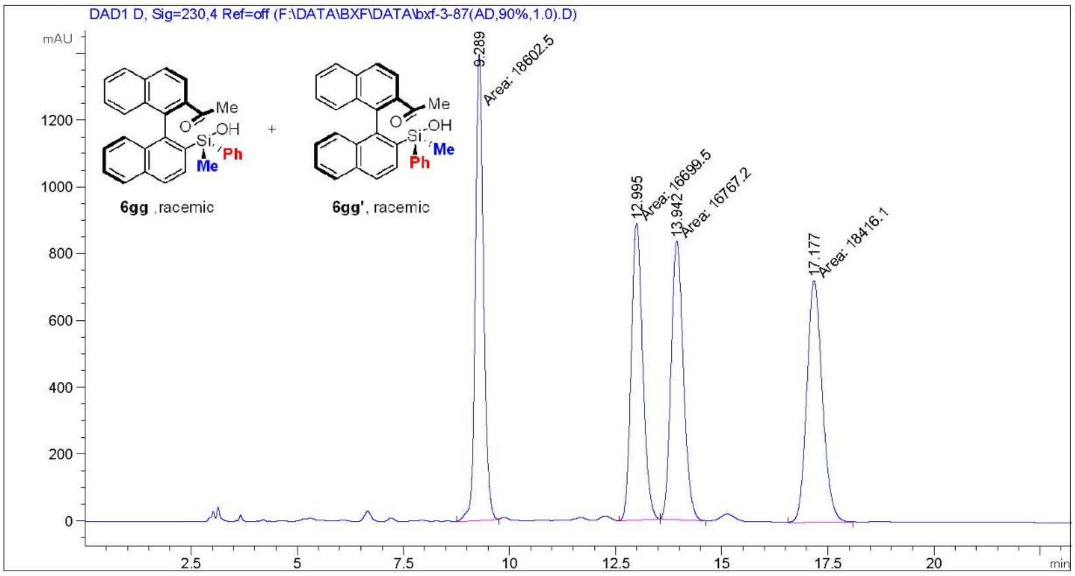

===

Area Percent Report

===

Sorted By : Signal
Multiplier : 1.0000
Dilution : 1.0000
Use Multiplier & Dilution Factor with ISTDs

Signal 1: DAD1 D, Sig=230,4 Ref=off

Peak #	RetTime [min]	Type	Width [min]	Area [mAU*s]	Height [mAU]	Area %
1	9.289	MM	0.2221	1.86025e4	1396.24304	26.3920
2	12.995	MM	0.3132	1.66995e4	888.73657	23.6922
3	13.942	MM	0.3352	1.67672e4	833.79187	23.7882
4	17.177	MM	0.4242	1.84161e4	723.56079	26.1276

Totals : 7.04853e4 3842.33228

===

*** End of Report ***

LC1260 02/07/2020 09:06:10 Page 1 of 1

Supplementary Figure 209. HPLC data of a mixture of **6gg** and **6gg’**

```
Data File F:\DATA\BXF\DATA\bxf-3-97(AD,90%,1.0).D
Sample Name: bxf-3-97(AD,90%,1.0)

=====================================================================
Acq. Operator   :
Sample Operator :
Acq. Instrument : LC1260                          Location :   1
Injection Date  : 05/06/2020 20:30:54
                                                Inj Volume : No inj
Acq. Method     : C:\Chem32\1\Methods\DEF_LC.M
Last changed    : 05/06/2020 19:59:36 by 系统
                  (modified after loading)
Analysis Method : C:\Chem32\1\Methods\DEF_LC.M
Last changed    : 13/02/2014 23:27:44 by SYSTEM
Additional Info : Peak(s) manually integrated
```

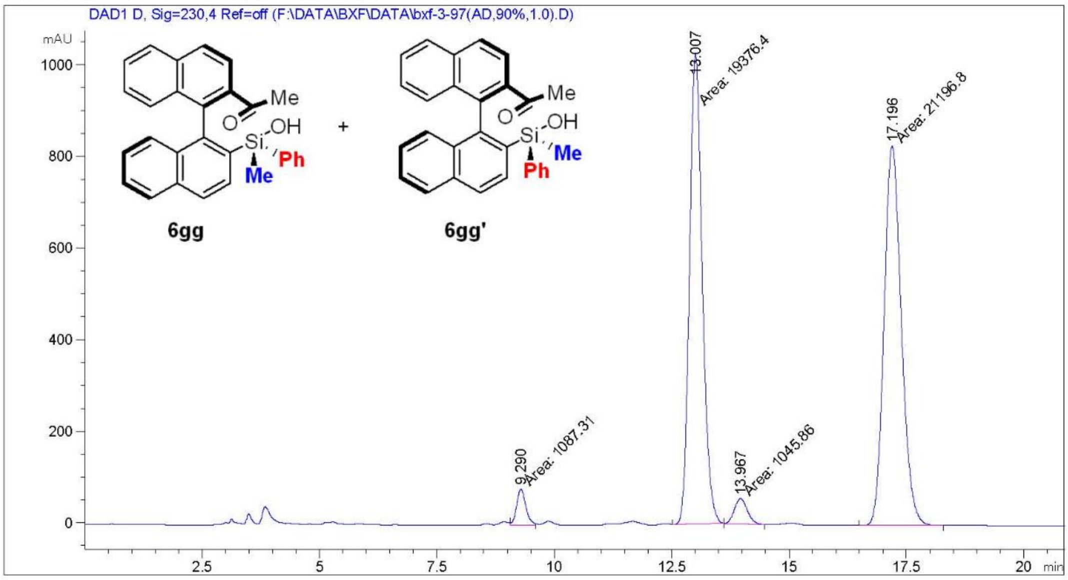


```
=====================================================================
                         Area Percent Report
=====================================================================

Sorted By             :      Signal
Multiplier            :      1.0000
Dilution              :      1.0000
Use Multiplier & Dilution Factor with ISTDs


Signal 1: DAD1 D, Sig=230,4 Ref=off
```

Peak #	RetTime [min]	Type	Width [min]	Area [mAU*s]	Height [mAU]	Area %
1	9.290	MM	0.2274	1087.30542	79.70558	2.5460
2	13.007	MM	0.3147	1.93764e4	1026.02612	45.3712
3	13.967	MM	0.3160	1045.86145	55.16383	2.4490
4	17.196	MM	0.4265	2.11968e4	828.32391	49.6338
Totals :				4.27063e4	1989.21945	

Supplementary Figure 210. HPLC data of a mixture of **6gg** and **6gg’**

Data File F:\DATA\JFENG\DATA\FJ-12-129-rac(AD,20%,1.0).D
Sample Name: FJ-12-129-rac(AD,20%,1.0)

===
Acq. Operator :
Location : 1
Injection Date : 30/05/2020 19:23:31
Acq. Method : DEF_LC.M
Analysis Method : C:\Chem32\1\Methods\DEF_LC.M
Last changed : 13/02/2014 23:27:44 by SYSTEM
Additional Info : Peak(s) manually integrated

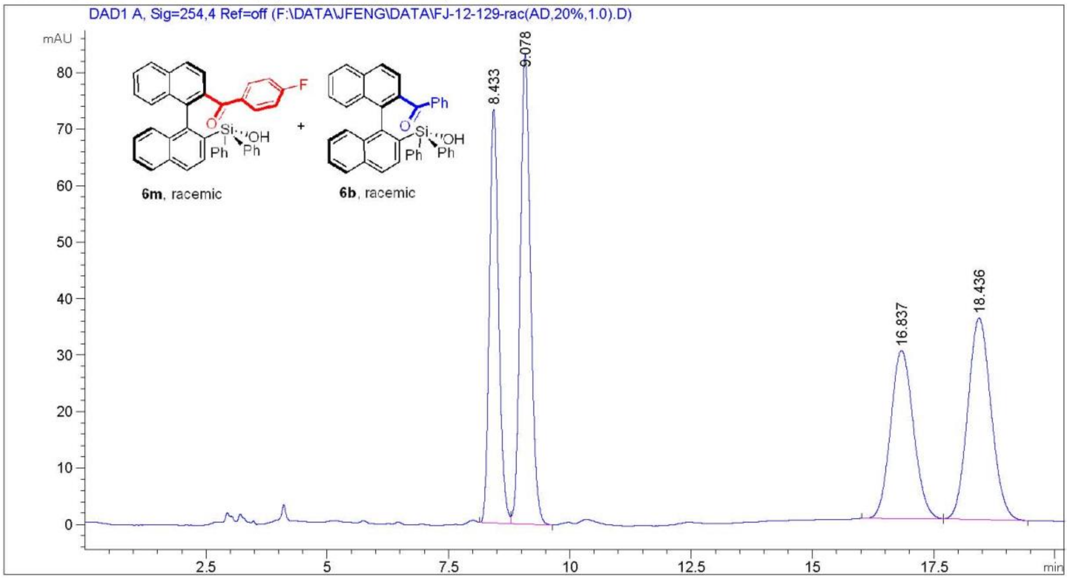

===
Area Percent Report
===

Sorted By : Signal
Multiplier : 1.0000
Dilution : 1.0000
Use Multiplier & Dilution Factor with ISTDs

Signal 1: DAD1 A, Sig=254,4 Ref=off

Peak #	RetTime [min]	Type	Width [min]	Area [mAU*s]	Height [mAU]	Area %
1	8.433	BV	0.2053	975.61163	73.31185	22.3421
2	9.078	VB	0.2249	1219.14490	83.20212	27.9191
3	16.837	BB	0.5125	976.81873	29.79408	22.3697
4	18.436	BB	0.5209	1195.12402	35.66485	27.3690

Totals : 4366.69928 221.97290

===
*** End of Report ***

LC1260 02/07/2020 08:57:37 Page 1 of 1

Supplementary Figure 211. HPLC data of a mixture of **6m** and **6b**

Data File F:\DATA\JFENG\DATA\FJ-12-129-(1)-(AD,20%,1.0).D
Sample Name: FJ-12-129-(1)-(AD,20%,1.0)

===

Acq. Operator :
Sample Operator :
Acq. Instrument : LC1260 Location : 1
Injection Date : 30/05/2020 19:45:47
Inj Volume : No inj
Acq. Method : C:\Chem32\1\Methods\DEF_LC.M
Last changed : 30/05/2020 19:17:08 by
(modified after loading)
Analysis Method : C:\Chem32\1\Methods\DEF_LC.M
Last changed : 13/02/2014 23:27:44 by SYSTEM
Additional Info : Peak(s) manually integrated

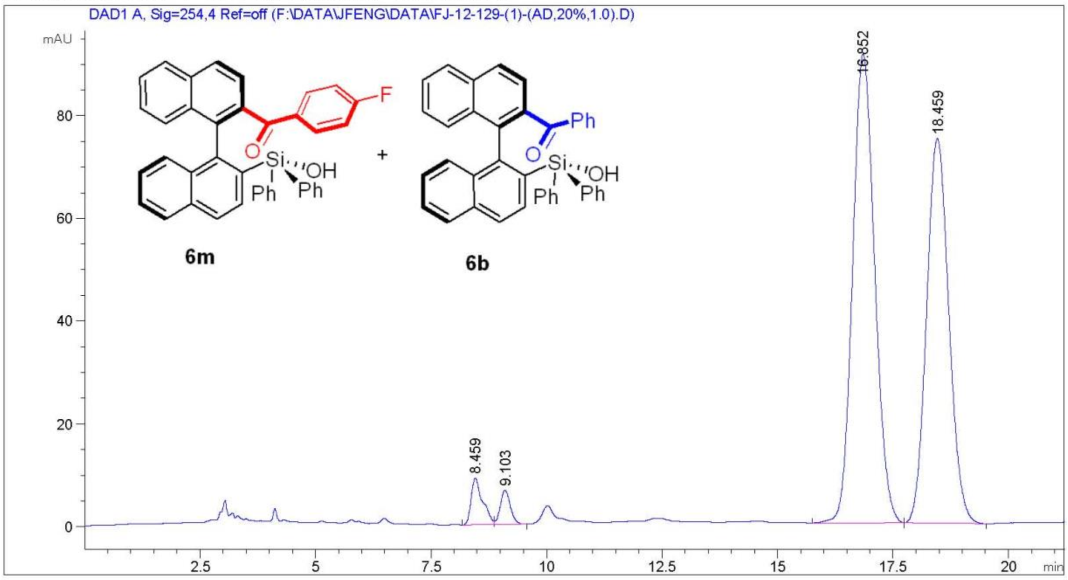

===

Area Percent Report

===

Sorted By : Signal
Multiplier : 1.0000
Dilution : 1.0000
Use Multiplier & Dilution Factor with ISTDs

Signal 1: DAD1 A, Sig=254,4 Ref=off

Peak #	RetTime [min]	Type	Width [min]	Area [mAU*s]	Height [mAU]	Area %
1	8.459	BV	0.2472	157.42493	9.12679	2.7260
2	9.103	VB	0.2299	100.14957	6.71967	1.7342
3	16.852	BB	0.5137	3016.47290	91.25153	52.2334
4	18.459	BB	0.5197	2500.93750	74.86879	43.3064

Totals : 5774.98490 181.96678

LC1260 02/07/2020 09:01:22 Page 1 of 2

Supplementary Figure 212. HPLC data of a mixture of **6m** and **6b**

Data File F:\DATA\JFENG\DATA\FJ-11-169-race-(1)(AD,10%,1.0).D
Sample Name: FJ-11-169-race-(1)(AD,10%,1.0)

===

Acq. Operator :
Sample Operator :
Acq. Instrument : LC1260 Location : 1
Injection Date : 01/03/2020 15:18:05
Inj Volume : Manually
Acq. Method : F:\METHOD\JFeng.M
Last changed : 01/03/2020 14:45:42 by
(modified after loading)
Analysis Method : F:\METHOD\JFeng.M
Last changed : 19/09/2014 20:49:49 by
Additional Info : Peak(s) manually integrated

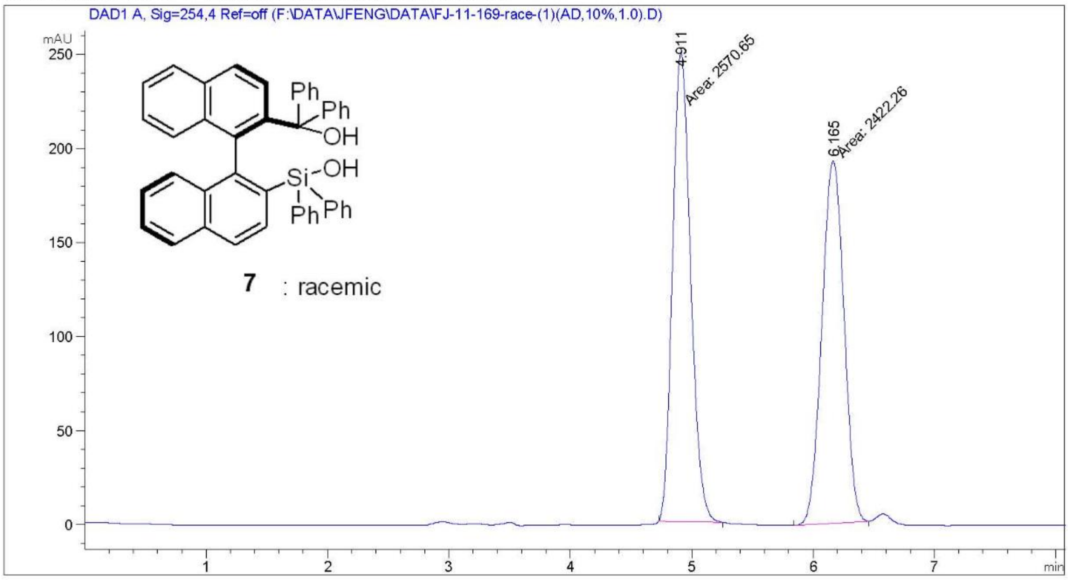

===

Area Percent Report

===

Sorted By : Signal
Multiplier : 1.0000
Dilution : 1.0000
Use Multiplier & Dilution Factor with ISTDs

Signal 1: DAD1 A, Sig=254,4 Ref=off

Peak #	RetTime [min]	Type	Width [min]	Area [mAU*s]	Height [mAU]	Area %
1	4.911	MM	0.1720	2570.65186	249.05777	51.4860
2	6.165	MM	0.2095	2422.26465	192.74477	48.5140

Totals : 4992.91650 441.80254

LC1260 01/03/2020 17:26:57 Page 1 of 2

Supplementary Figure 213. HPLC data of **7**

Data File F:\DATA\JFENG\DATA\FJ-11-169-chiral-0226(AD,10%,1.0).D
Sample Name: FJ-11-169-chiral-0226(AD,10%,1.0)

===

Acq. Operator :
Sample Operator :
Acq. Instrument : LC1260 Location : 1
Injection Date : 26/02/2020 15:16:53
Inj Volume : Manually
Acq. Method : F:\METHOD\JFeng.M
Last changed : 26/02/2020 14:48:03 by
(modified after loading)
Analysis Method : F:\METHOD\JFeng.M
Last changed : 19/09/2014 20:49:49 by
Additional Info : Peak(s) manually integrated

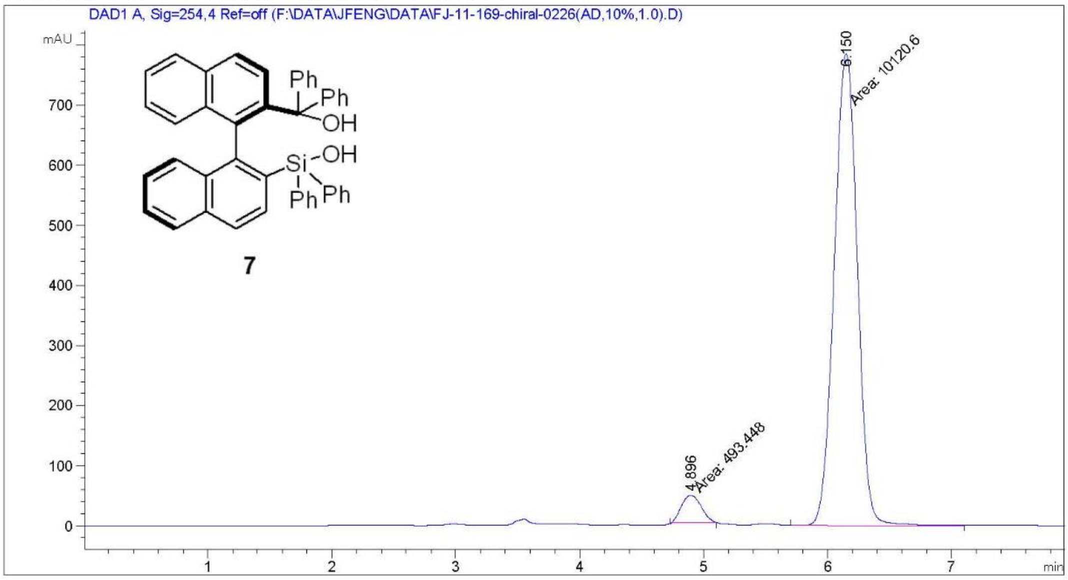

===

Area Percent Report

===

Sorted By : Signal
Multiplier : 1.0000
Dilution : 1.0000
Use Multiplier & Dilution Factor with ISTDs

Signal 1: DAD1 A, Sig=254,4 Ref=off

Peak #	RetTime [min]	Type	Width [min]	Area [mAU*s]	Height [mAU]	Area %
1	4.896	MM	0.1824	493.44849	45.10007	4.6490
2	6.150	MM	0.2149	1.01206e4	784.92267	95.3510

Totals : 1.06141e4 830.02274

Supplementary Figure 214. HPLC data of **7**

Data File F:\DATA\JFENG\DATA\FJ-11-123-rac-(2)(AD,10%,1.0).D
Sample Name: FJ-11-123-rac-(2)(AD,10%,1.0)

===

Acq. Operator :

Location : 1

Injection Date : 01/03/2020 17:23:51

Acq. Method : JFeng.M

Analysis Method : F:\METHOD\JFeng.M

Last changed : 19/09/2014 20:49:49 by

Additional Info : Peak(s) manually integrated

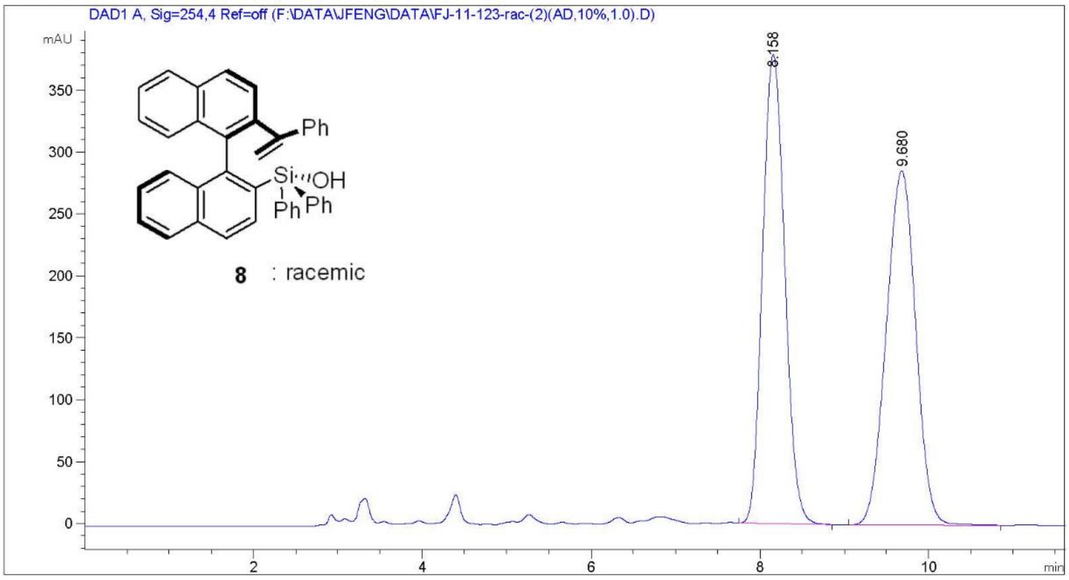

===

Area Percent Report

===

Sorted By : Signal

Multiplier : 1.0000

Dilution : 1.0000

Use Multiplier & Dilution Factor with ISTDs

Signal 1: DAD1 A, Sig=254,4 Ref=off

Peak #	RetTime [min]	Type	Width [min]	Area [mAU*s]	Height [mAU]	Area %
1	8.158	BB	0.2881	6883.85938	378.69330	49.8741
2	9.680	BB	0.3824	6918.61914	285.77731	50.1259

Totals : 1.38025e4 664.47061

===

*** End of Report ***

LC1260 01/03/2020 17:36:20 Page 1 of 1

Supplementary Figure 215. HPLC data of **8**

Data File F:\DATA\JFENG\DATA\FJ-11-123-chiral-(8)(AD,10%,1.0).D
Sample Name: FJ-11-123-chiral-(8)(AD,10%,1.0)

===

Acq. Operator :
Sample Operator :
Acq. Instrument : LC1260 Location : 1
Injection Date : 01/03/2020 17:09:06
Inj Volume : Manually
Acq. Method : F:\METHOD\JFeng.M
Last changed : 01/03/2020 16:47:42 by
(modified after loading)
Analysis Method : F:\METHOD\JFeng.M
Last changed : 19/09/2014 20:49:49 by
Additional Info : Peak(s) manually integrated

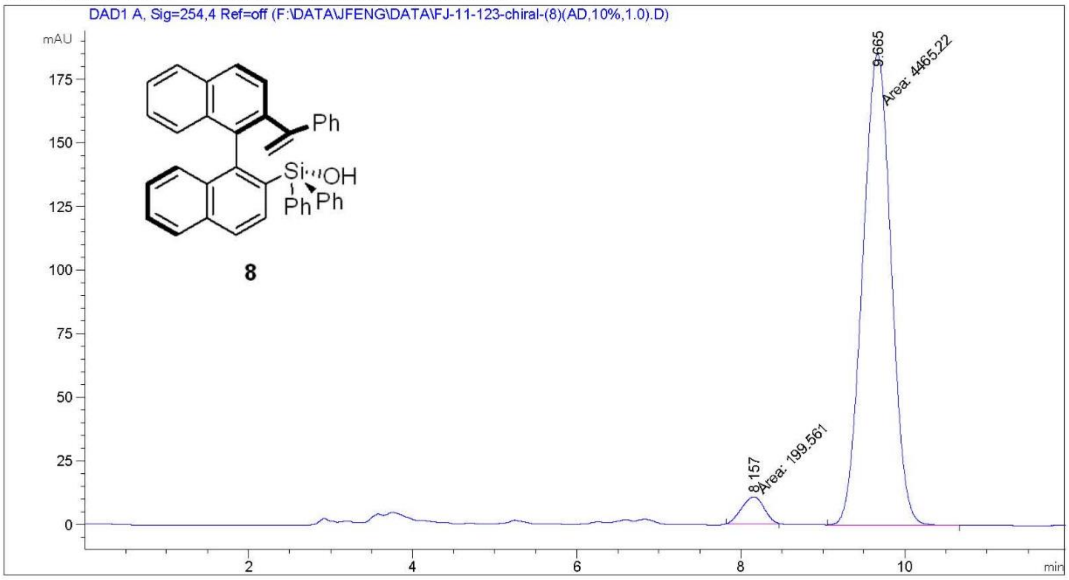

===

Area Percent Report

===

Sorted By : Signal
Multiplier : 1.0000
Dilution : 1.0000
Use Multiplier & Dilution Factor with ISTDs

Signal 1: DAD1 A, Sig=254,4 Ref=off

Peak #	RetTime [min]	Type	Width [min]	Area [mAU*s]	Height [mAU]	Area %
1	8.157	MM	0.3126	199.56143	10.63889	4.2780
2	9.665	MM	0.4017	4465.22070	185.24721	95.7220

Totals : 4664.78214 195.88610

Supplementary Figure 216. HPLC data of **8**

```
Data File F:\DATA\JFENG\DATA\FJ-11-170-rac-0225(AD,6%,1.0).D
Sample Name: FJ-11-170-rac-0225(AD,6%,1.0)

=====================================================================
Acq. Operator   :
Sample Operator :
Acq. Instrument : LC1260                          Location :    1
Injection Date  : 25/02/2020 10:48:00
                                               Inj Volume : Manually
Acq. Method     : F:\METHOD\JFeng.M
Last changed    : 25/02/2020 10:45:25 by
                  (modified after loading)
Analysis Method : F:\METHOD\JFeng.M
Last changed    : 19/09/2014 20:49:49 by
Additional Info : Peak(s) manually integrated
```

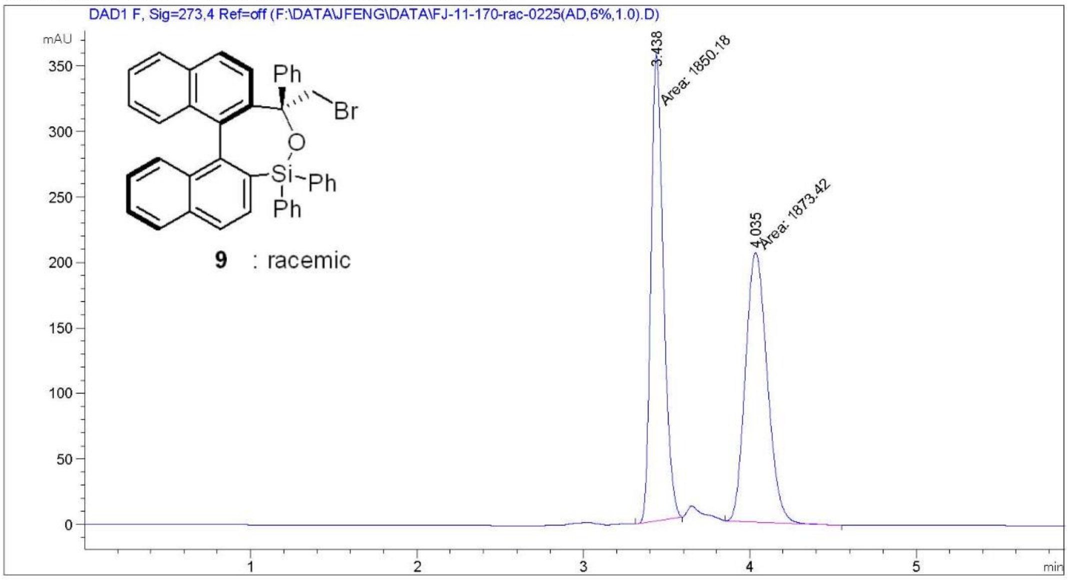


```
=====================================================================
                        Area Percent Report
=====================================================================

Sorted By           :      Signal
Multiplier          :      1.0000
Dilution            :      1.0000
Use Multiplier & Dilution Factor with ISTDs


Signal 1: DAD1 F, Sig=273,4 Ref=off

Peak RetTime Type  Width     Area      Height     Area
  #   [min]        [min]   [mAU*s]     [mAU]        %
----|-------|----|-------|----------|----------|--------|
   1   3.438 MM    0.0863 1850.17505  357.46512  49.6878
   2   4.035 MM    0.1516 1873.42151  206.02863  50.3122

Totals :                  3723.59656  563.49374
```

LC1260 03/03/2020 16:26:07   Page   1 of 2

Supplementary Figure 217. HPLC data of **9**

Data File F:\DATA\JFENG\DATA\FJ-11-170-chiral-0225(AD,6%,1.0).D
Sample Name: FJ-11-170-chiral-0225(AD,6%,1.0)

===

Acq. Operator :

Location : 1

Injection Date : 25/02/2020 10:57:15

Acq. Method : JFeng.M

Analysis Method : F:\METHOD\JFeng.M

Last changed : 19/09/2014 20:49:49 by

Additional Info : Peak(s) manually integrated

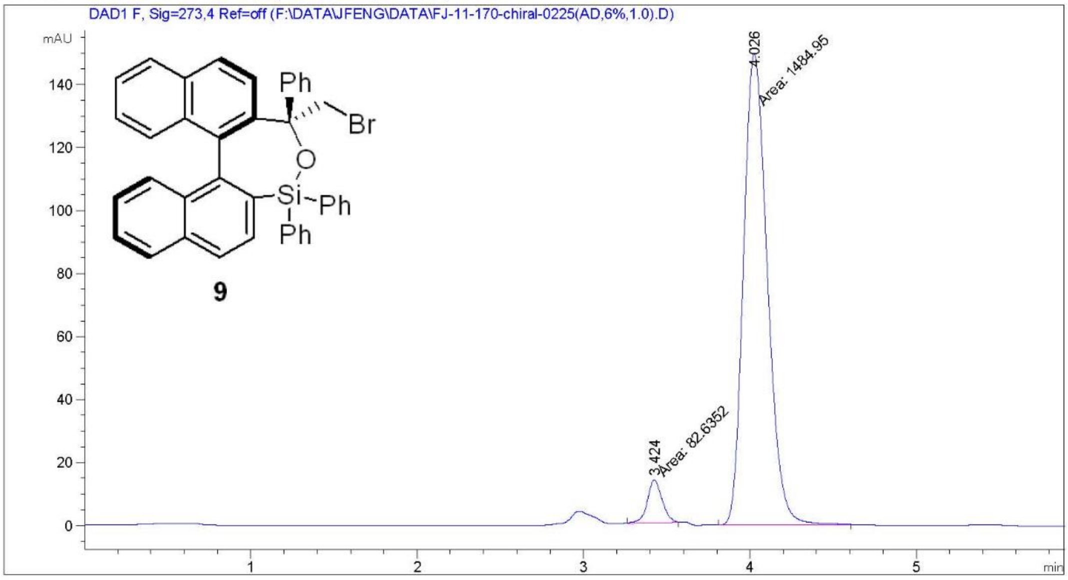

===

Area Percent Report

===

Sorted By : Signal

Multiplier : 1.0000

Dilution : 1.0000

Use Multiplier & Dilution Factor with ISTDs

Signal 1: DAD1 F, Sig=273,4 Ref=off

Peak #	RetTime [min]	Type	Width [min]	Area [mAU*s]	Height [mAU]	Area %
1	3.424	MM	0.1019	82.63519	13.52122	5.2715
2	4.026	MM	0.1654	1484.94800	149.61452	94.7285

Totals : 1567.58319 163.13574

===

*** End of Report ***

LC1260 03/03/2020 16:27:59 Page 1 of 1

Supplementary Figure 218. HPLC data of **9**

```
Data File F:\DATA\JFENG\DATA\FJ-12-195-a-race(OD,5%,1.0).D
Sample Name: FJ-12-195-a-RACE(OD,5%,1.0)

=====================================================================
Acq. Operator   :
                                                Location :   1
Injection Date  : 27/06/2020 16:02:11
Acq. Method     : DEF_LC.M
Analysis Method : C:\Chem32\1\Methods\DEF_LC.M
Last changed    : 13/02/2014 23:27:44 by SYSTEM
Additional Info : Peak(s) manually integrated
```

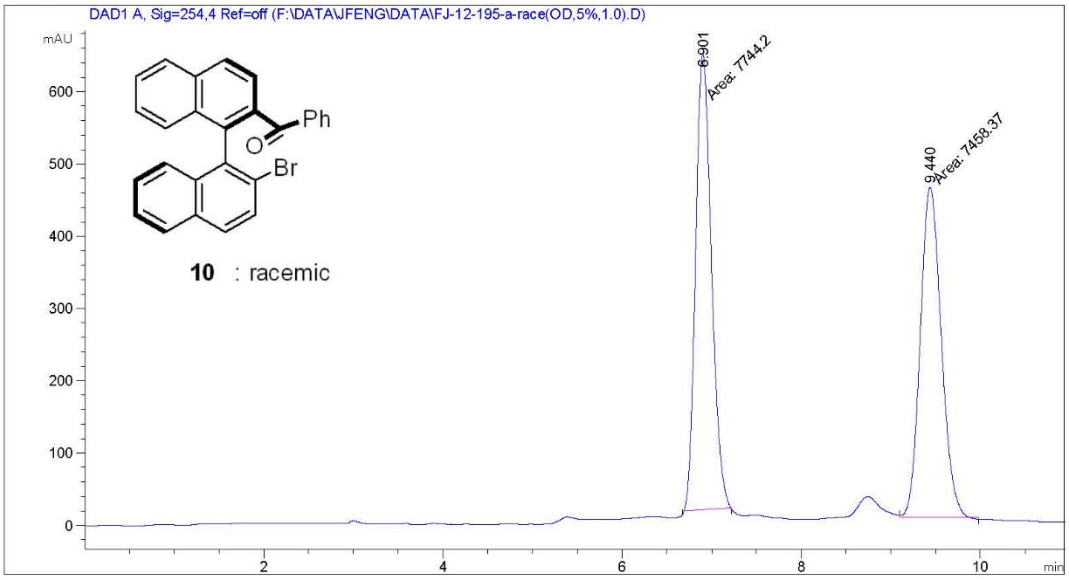


```
=====================================================================
                        Area Percent Report
=====================================================================

Sorted By             :      Signal
Multiplier            :      1.0000
Dilution              :      1.0000
Use Multiplier & Dilution Factor with ISTDs


Signal 1: DAD1 A, Sig=254,4 Ref=off
```

Peak #	RetTime [min]	Type	Width [min]	Area [mAU*s]	Height [mAU]	Area %
1	6.901	MM	0.2046	7744.19629	630.75885	50.9401
2	9.440	MM	0.2720	7458.36865	456.97809	49.0599
Totals :				1.52026e4	1087.73694	

```
=====================================================================
                        *** End of Report ***
```

LC1260 01/07/2020 21:13:52 Page 1 of 1

Supplementary Figure 219. HPLC data of **10**

Data File F:\DATA\JFENG\DATA\FJ-12-199-(OD,5%,1.0).D
Sample Name: FJ-12-199-(OD,5%,1.0)

===

Acq. Operator :
Sample Operator :
Acq. Instrument : LC1260 Location : 1
Injection Date : 27/06/2020 16:15:48
Inj Volume : No inj
Acq. Method : C:\Chem32\1\Methods\DEF_LC.M
Last changed : 27/06/2020 15:54:59 by
(modified after loading)
Analysis Method : C:\Chem32\1\Methods\DEF_LC.M
Last changed : 13/02/2014 23:27:44 by SYSTEM
Additional Info : Peak(s) manually integrated

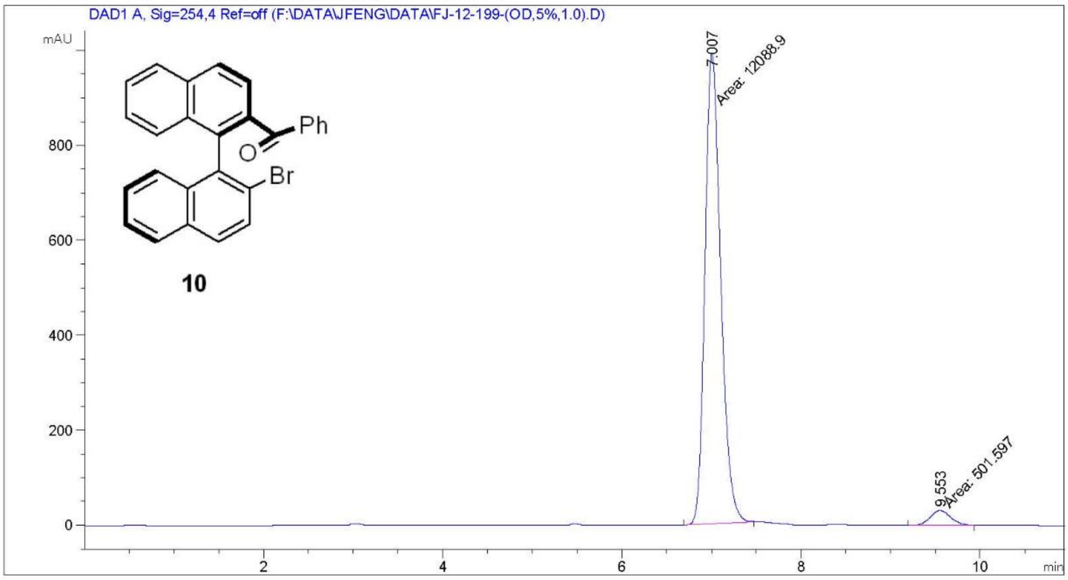

===

Area Percent Report

===

Sorted By : Signal
Multiplier : 1.0000
Dilution : 1.0000
Use Multiplier & Dilution Factor with ISTDs

Signal 1: DAD1 A, Sig=254,4 Ref=off

Peak #	RetTime [min]	Type	Width [min]	Area [mAU*s]	Height [mAU]	Area %
1	7.007	MM	0.2034	1.20889e4	990.55432	96.0161
2	9.553	MM	0.2690	501.59738	31.07435	3.9839

Totals : 1.25905e4 1021.62867

Supplementary Figure 220. HPLC data of **10**

Data File F:\DATA\JFENG\DATA\FJ-12-198-race(OD,5%,1.0).D
Sample Name: FJ-12-198-race(OD,5%,1.0)

===

Acq. Operator :

Location : 1

Injection Date : 28/06/2020 09:24:05
Acq. Method : JFeng.M
Analysis Method : C:\Chem32\1\Methods\DEF_LC.M
Last changed : 13/02/2014 23:27:44 by SYSTEM
Additional Info : Peak(s) manually integrated

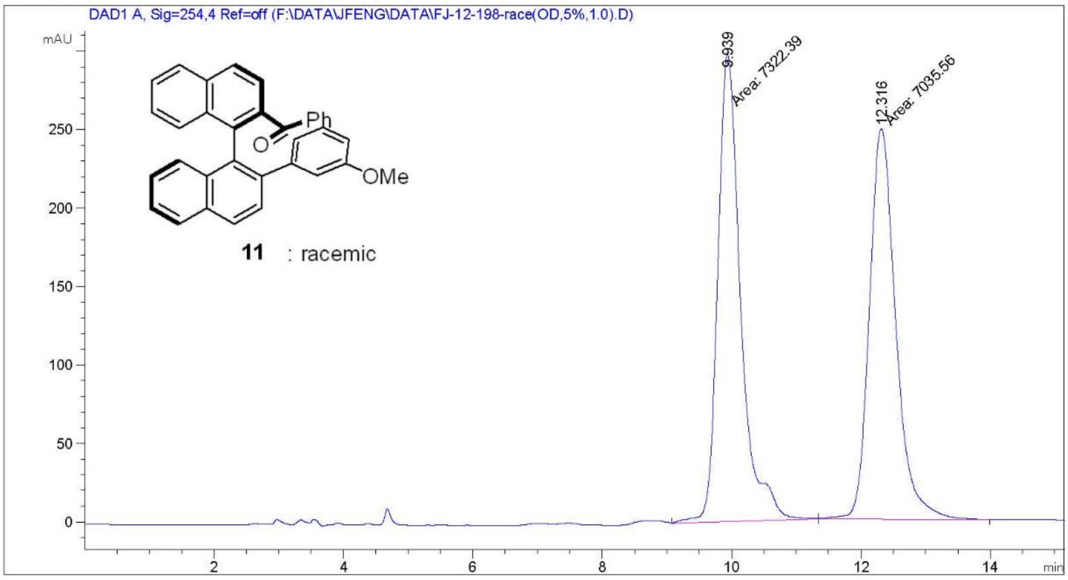

===

Area Percent Report

===

Sorted By : Signal
Multiplier : 1.0000
Dilution : 1.0000
Use Multiplier & Dilution Factor with ISTDs

Signal 1: DAD1 A, Sig=254,4 Ref=off

Peak #	RetTime [min]	Type	Width [min]	Area [mAU*s]	Height [mAU]	Area %
1	9.939	MM	0.4101	7322.38721	297.60254	50.9989
2	12.316	MM	0.4715	7035.55566	248.70322	49.0011

Totals : 1.43579e4 546.30576

===

*** End of Report ***

LC1260 01/07/2020 21:16:01 Page 1 of 1

Supplementary Figure 221. HPLC data of **11**

Data File F:\DATA\JFENG\DATA\FJ-12-200-(OD,5%,1.0).D
Sample Name: FJ-12-200-(OD,5%,1.0)

===
Acq. Operator :
Location : 1
Injection Date : 28/06/2020 09:42:18
Acq. Method : JFeng.M
Analysis Method : C:\Chem32\1\Methods\DEF_LC.M
Last changed : 13/02/2014 23:27:44 by SYSTEM
Additional Info : Peak(s) manually integrated

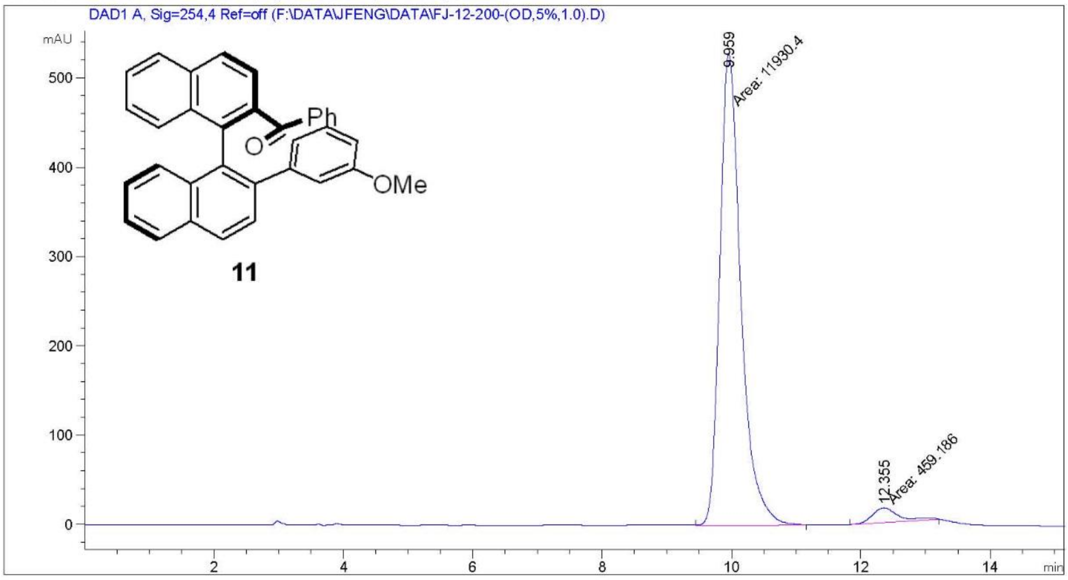

===
Area Percent Report
===

Sorted By : Signal
Multiplier : 1.0000
Dilution : 1.0000
Use Multiplier & Dilution Factor with ISTDs

Signal 1: DAD1 A, Sig=254,4 Ref=off

Peak #	RetTime [min]	Type	Width [min]	Area [mAU*s]	Height [mAU]	Area %
1	9.959	MM	0.3773	1.19304e4	527.06537	96.2938
2	12.355	MM	0.4676	459.18649	16.36664	3.7062

Totals : 1.23896e4 543.43201

===
*** End of Report ***

LC1260 01/07/2020 21:17:25 Page 1 of 1

Supplementary Figure 222. HPLC data of **11**

Data File F:\DATA\JFENG\DATA\FJ-12-132-race-(1)(AD,20%,1.0).D
Sample Name: FJ-12-132-race-(1)(AD,20%,1.0)

===

Acq. Operator :

Location : 1

Injection Date : 29/06/2020 21:26:16

Acq. Method : JFeng.M

Analysis Method : C:\Chem32\1\Methods\DEF_LC.M

Last changed : 13/02/2014 23:27:44 by SYSTEM

Additional Info : Peak(s) manually integrated

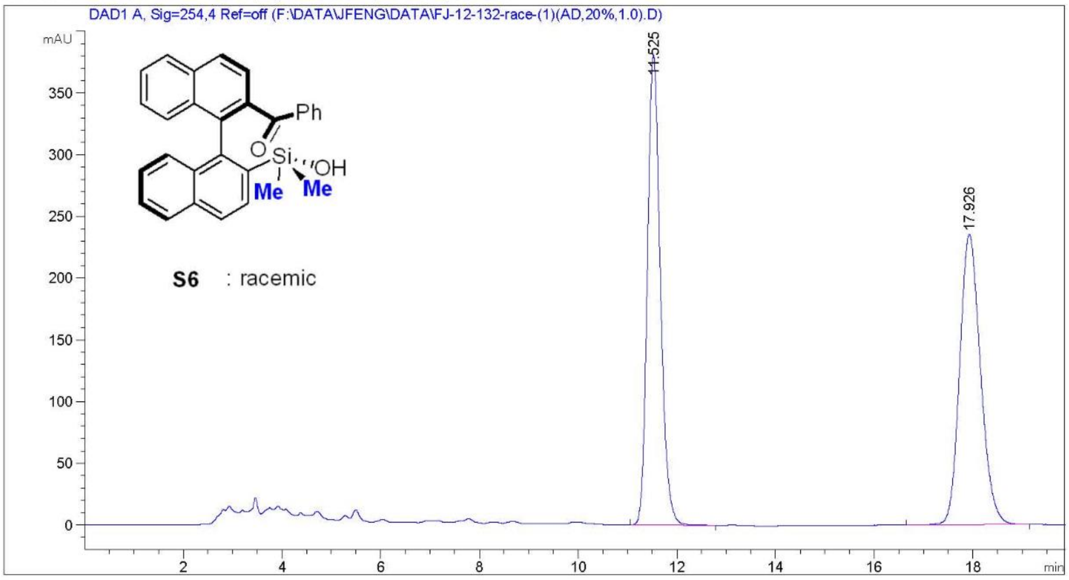

===

Area Percent Report

===

Sorted By : Signal

Multiplier : 1.0000

Dilution : 1.0000

Use Multiplier & Dilution Factor with ISTDs

Signal 1: DAD1 A, Sig=254,4 Ref=off

Peak #	RetTime [min]	Type	Width [min]	Area [mAU*s]	Height [mAU]	Area %
1	11.525	BB	0.2767	6883.16504	381.08438	49.9439
2	17.926	BB	0.4543	6898.61523	235.11957	50.0561

Totals : 1.37818e4 616.20395

===

*** End of Report ***

LC1260 01/07/2020 21:10:00 Page 1 of 1

Supplementary Figure 223. HPLC data of **S6**

Data File F:\DATA\JFENG\DATA\FJ-12-197(AD,20%,1.0).D
Sample Name: FJ-12-197(AD,20%,1.0)

===

Acq. Operator :
Sample Operator :
Acq. Instrument : LC1260 Location : 1
Injection Date : 29/06/2020 21:48:05
Inj Volume : Manually
Acq. Method : F:\METHOD\JFeng.M
Last changed : 29/06/2020 21:10:13 by
(modified after loading)
Analysis Method : C:\Chem32\1\Methods\DEF_LC.M
Last changed : 13/02/2014 23:27:44 by SYSTEM
Additional Info : Peak(s) manually integrated

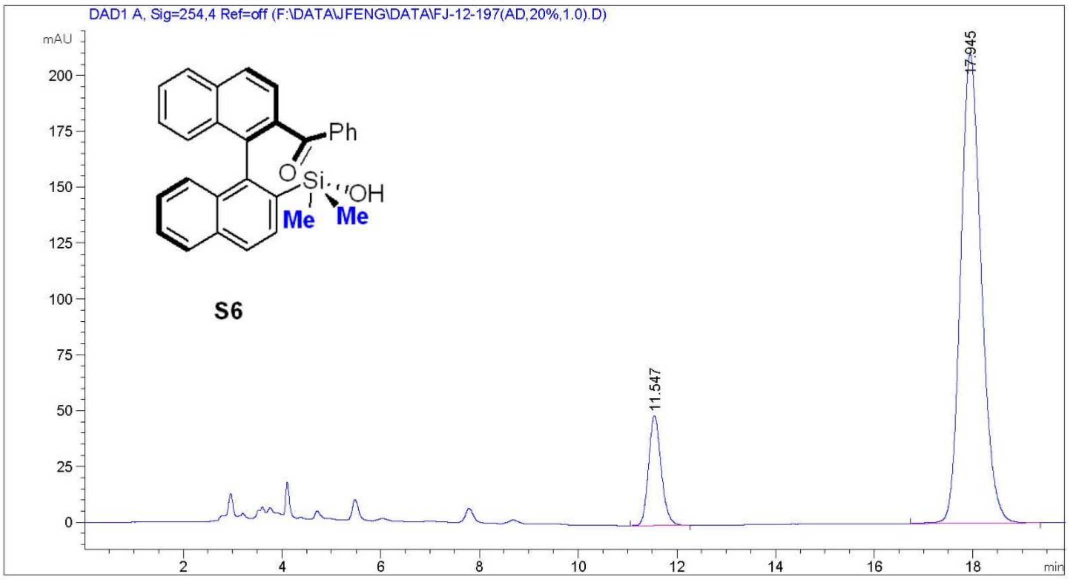

===

Area Percent Report

===

Sorted By : Signal
Multiplier : 1.0000
Dilution : 1.0000
Use Multiplier & Dilution Factor with ISTDs

Signal 1: DAD1 A, Sig=254,4 Ref=off

Peak #	RetTime [min]	Type	Width [min]	Area [mAU*s]	Height [mAU]	Area %
1	11.547	BB	0.2694	863.20203	49.03444	12.4029
2	17.945	BB	0.4469	6096.48047	209.86026	87.5971

Totals : 6959.68250 258.89470

LC1260 01/07/2020 21:10:55 Page 1 of 2

Supplementary Figure 224. HPLC data of **S6**

Supplementary References

1. Wu, B., Yoshikai, N. Conversion of 2-Iodobiaryls into 2,2’-Diiodobiaryls via Oxidation-Iodination Sequences: A Versatile Route to Ladder-Type Heterofluorenes *Angew. Chem. Int. Ed*. **54**, 8736– 8739 (2015).
2. Zhao, K., Duan, L., Xu, S., Jiang, J., Fu, Y., Gu, Z. Enhanced Reactivity by Torsional Strain of Cyclic Diaryliodonium in Cu-Catalyzed Enantioselective Ring-Opening Reaction *Chem*, **4**, 599-612 (**2018**).
3. Liao, Y., Liang, C. One-pot synthesis of thioesters with sodium thiosulfate as a sulfur surrogate under transition metal-free conditions. *Org. Biomol. Chem*., **16**, 1871-1881 (2018)
4. Leifert, D., Studer, A. 9-Silafluorenes via Base-Promoted Homolytic Aromatic Substitution (BHAS) – The Electron as a Catalyst *Org. Lett.* **17**, 386–389 (2015).
5. Frisch, M. J., Trucks, G., W. Schlegel, H. B., Scuseria, G. E., Robb, M. A., Cheeseman, J. R., Scalmani, G., Barone, V., Petersson, G. A., Nakatsuji, H., Li, X., Caricato, M., Marenich, A. V., Bloino, J., Janesko, B. G., Gomperts, R., Mennucci, B., Hratchian, H. P., Ortiz, J. V., Izmaylov, A. F., Sonnenberg, J. L., Williams-Young, D.,Ding, F., Lipparini, F., Egidi, F., Goings, J., Peng, B., Petrone, A., Henderson, T., Ranasinghe, D., Zakrzewski, V. G., Gao, J., Rega, N., Zheng, G.,, Liang, W., Hada, M., Ehara, M., Toyota, K., Fukuda, R., Hasegawa, J., Ishida, M., Nakajima, T., Honda, Y., Kitao, O., Nakai, H., Vreven, T., Throssell, K., Montgomery, J. A., Peralta, Jr., J. E., Ogliaro, F., Bearpark, M. J., Heyd, J. J., Brothers, E. N., Kudin, K. N., Staroverov, V. N., Keith, T. A., Kobayashi, R., Normand, J., Raghavachari, K., Rendell, A. P., Burant, J. C., Iyengar, S. S., Tomasi, J., Cossi, M., Millam, J. M., Klene, M., Adamo, C., Cammi, R., Ochterski, J. W., Martin, R. L., Morokuma, K., Farkas, O., Foresman, J. B. and Fox. D. J. *Gaussian 16, RevisionC.03*, Gaussian, Inc., Wallingford CT (2016).
6. Miehlich, B., Savin, A., Stoll, H. and Preuss. H. Results obtained with the correlation-energy density functionals of Becke and Lee, Yang and Parr *Chem. Phys. Lett.* **157**, 200-206 (1989).
7. Lee, C., Yang, W., and Parr. R. G. Development of the Colle-Salvetti correlation-energy formula into a functional of the electron density *Phys. Rev. B* **37**, 785-789 (1988).
8. Grimme, S., Antony, J., Ehrlich, S., and Krieg. H. A consistent and accurate ab initio parameterization of density functional dispersion correction (DFT-D) for the 94 elements H-Pu *J. Chem. Phys*. **132**, 154104 (2010).
9. Grimme, S., Ehrlich, S., and Goerigk. L., Effect of the damping function in dispersion corrected density functional theory *J. Comp. Chem.* **32**, 1456-1465 (2011).
10. Pople, J. A., Krishnan, R., Schlegel, H. B. and Binkley. J. S. Electron Correlation Theories and Their Application to the Study of Simple Reaction Potential Surfaces *Int. J. Quantum Chem.* **14**, 545-560 (1978).
11. Krishnan, R., Binkley, J. S., Seeger, R., and Pople. J. A. Self-consistent molecular orbital methods. XX. A basis set for correlated wave functions *J. Chem. Phys*. **72**, 650-654 (1980).
12. Fukui. K. The path of chemical-reactions - The IRC approach *Acc. Chem. Res*. **14**, 363-368 (1981).
13. Schaefer, A., Horn, H. and Ahlrichs. R. Fully optimized contracted Gaussian-basis sets for atoms Li to Kr *J. Chem. Phys.* **97**, 2571-2577 (1992).
14. Schaefer, A., Huber, C., and Ahlrichs. R. Fully optimized contracted Gaussian-basis sets of

triple zeta valence quality for atoms Li to Kr *J. Chem. Phys.* **100**, 5829-5835 (1994).
15. Weigend, F., and Ahlrichs. R. Balanced basis sets of split valence, triple zeta valence and quadruple zeta valence quality for H to Rn: Design and assessment of accuracy *Phys. Chem. Chem. Phys*. **7**, 3297-3305 (2005).
16. Weigend. F. Accurate Coulomb-fitting basis sets for H to Rn *Phys. Chem. Chem. Phys.* **8**, 1057-1065 (2006).
17. Goerigk, L. and Grimme. S. Efficient and Accurate Double-Hybrid-Meta-GGA Density Functionals–Evaluation with the Extended GMTKN30 Database for General Main Group Thermochemistry, Kinetics, and Noncovalent Interactions *J. Chem. Theory Comput*. **7**, 291-309 (2011).
18. Grimme. S. Semiempirical hybrid density functional with perturbative second-order correlation *J. Chem. Phys.* **124**, 034108 (2006).
19. Schwabe, T. and Grimme. S. Double-hybrid density functionals with long-range dispersion corrections: higher accuracy and extended applicability *Phys. Chem. Chem. Phys.* **9**, 3397-3406 (2007).
