# Supplementary material for: Catalytic asymmetric C–Si bond activation via torsional strain-promoted Rh-catalyzed aryl-Narasaka acylation
Source: Nat Commun. 2020 Sep 7;11:4449. doi: 10.1038/s41467-020-18273-3 (PMC7477585; doi:10.1038/s41467-020-18273-3)
Supplement: Supplementary file 3 — Description of Additional Supplementary Files [file 41467_2020_18273_MOESM3_ESM.pdf]

Description of Additional Supplementary Files

File Name: Supplementary Data 1

Description: This file contains Cartesian coordinates for all optimized geometries, including **4c**, **H_2**, **4c-HH**, **(*R*)-4a**, **4a-HH**, **4c'**, **4c'-H**, **4c''**, **4a'**, **4a'-H**, **4a''**, **TS-rs**, **(*S*)-4a**.
