# Supplementary material for: Catalytic asymmetric C–Si bond activation via torsional strain-promoted Rh-catalyzed aryl-Narasaka acylation
Source: Nat Commun. 2020 Sep 7;11:4449. doi: 10.1038/s41467-020-18273-3 (PMC7477585; doi:10.1038/s41467-020-18273-3)
Supplement: Supplementary file 4 — Supplementary Data 1 [file 41467_2020_18273_MOESM4_ESM.zip › 250280_2_supp_4829996_qrq2fr (1).docx]

**Cartesian coordinates for all optimized geometries:**

**4c**

0 1

C -1.54746100 -2.12497800 -0.00012200

C -2.93462100 -1.99903900 0.00008800

C -3.53108700 -0.73962200 0.00021600

C -2.73270900 0.40457300 0.00009100

C -1.34405900 0.29843600 -0.00011800

C -0.74323500 -0.98364400 -0.00019300

H -3.55355300 -2.88919500 0.00015700

H -4.61150200 -0.65117500 0.00041400

H -3.20518600 1.38201100 0.00014400

C 0.74318500 -0.98366000 -0.00020800

C 1.54736700 -2.12502400 -0.00016400

C 1.34405300 0.29840600 -0.00013300

C 2.93453200 -1.99914200 0.00001900

C 2.73270900 0.40447600 0.00005500

C 3.53104300 -0.73975000 0.00014900

H 3.55343200 -2.88932100 0.00010000

H 3.20522600 1.38189500 0.00008600

H 4.61146100 -0.65134400 0.00032600

C 0.00019700 2.68645000 1.54311400

H -0.00104900 2.06880100 2.44399300

H 0.88462700 3.32977400 1.57220200

H -0.88247700 3.33217300 1.57117100

C -0.00003700 2.68695900 -1.54290100

H -0.88451900 3.33021400 -1.57204800

H 0.88252100 3.33287300 -1.57054600

H 0.00154400 2.06955800 -2.44394600

H -1.10234900 -3.11318800 -0.00024400

H 1.10221800 -3.11321800 -0.00026500

Si 0.00002400 1.61239300 -0.00006400

**H_2_**

0 1

H 0.00000000 0.00000000 0.37226500

H 0.00000000 0.00000000 -0.37226500

**4c-HH**

0 1

C 1.47059600 -0.24268400 1.18946100

C 0.80060300 -0.86828100 0.13363000

C 1.55449400 -1.45230100 -0.88964000

C 2.94434500 -1.38507700 -0.87306000

C 3.60179600 -0.74102000 0.17319800

C 2.86108100 -0.17628400 1.20872000

H 0.89383200 0.18215900 2.00150800

H 1.04163900 -1.93720000 -1.71222900

H 3.51477300 -1.82955500 -1.68053900

H 4.68403500 -0.68564300 0.18431200

H 3.36564500 0.31173400 2.03475400

C -0.68658200 -0.90660800 0.09625100

C -1.44375500 0.27452600 -0.05780900

C -1.33032900 -2.14274800 0.21053200

C -2.84288700 0.15785100 -0.06878500

C -2.71868000 -2.22914500 0.20125300

H -0.73050400 -3.03874700 0.32467800

C -3.47964000 -1.07232700 0.06758400

H -3.44703000 1.04894500 -0.20377400

H -3.20176600 -3.19461600 0.29972800

H -4.56225100 -1.12766600 0.05775300

Si -0.71218200 1.99473700 -0.36711300

H -1.85480500 2.77170500 -0.92991100

C 0.68623600 1.97844700 -1.62590700

H 1.59086900 1.52444400 -1.21807500

H 0.40268700 1.42007800 -2.52148500

H 0.92419600 3.00280900 -1.92803800

C -0.16540500 2.87609000 1.20844000

H -0.01904900 3.94119700 1.00489300

H -0.91927100 2.78584800 1.99494400

H 0.77631600 2.47555600 1.58785100

(*R*)-**4a**

0 1

C -2.64529300 1.61794500 0.71199900

C -3.48120100 0.52783600 0.70427100

C -3.01288200 -0.72653400 0.24397900

C -1.63418100 -0.88323800 -0.12512700

C -0.74321500 0.21822700 0.09243700

C -1.27986500 1.47605600 0.39433300

H -4.93641500 -1.69287800 0.38466800

H -3.03889300 2.59544100 0.97155900

H -4.52414400 0.62390300 0.98633400

C -3.90589400 -1.81590700 0.06949300

C -1.26387400 -2.08348300 -0.79145800

C -2.16459600 -3.10122200 -0.98521400

C -3.49325800 -2.98296300 -0.52032000

H -0.25930900 -2.18503000 -1.17390800

H -1.85575700 -3.99879300 -1.50830400

H -4.18965600 -3.80148000 -0.66053400

C 0.74318700 0.21825000 -0.09252200

C 1.63419700 -0.88318500 0.12507100

C 1.27979200 1.47607500 -0.39454300

C 3.01287500 -0.72648200 -0.24410900

C 1.26394400 -2.08336800 0.79155100

C 2.64520100 1.61794700 -0.71230400

C 3.90592300 -1.81580600 -0.06952300

C 3.48113300 0.52785900 -0.70454200

C 2.16470700 -3.10104900 0.98542100

H 0.25938600 -2.18489200 1.17401400

H 3.03876700 2.59542800 -0.97197500

C 3.49334900 -2.98279000 0.52047500

H 4.93643300 -1.69278800 -0.38473800

H 4.52404700 0.62392500 -0.98671400

H 1.85593000 -3.99855900 1.50865000

H 4.18979000 -3.80125200 0.66079400

Si -0.00002800 2.78965700 -0.00001600

C 0.55690000 3.86374400 1.43920000

H 1.39772900 4.50112600 1.14995600

H -0.25355100 4.51598800 1.77755100

H 0.87322300 3.24583000 2.28234400

C -0.55690600 3.86435500 -1.43877200

H -1.39752400 4.50182700 -1.14911300

H 0.25363300 4.51653700 -1.77702600

H -0.87354500 3.24685000 -2.28209800

**4a-HH**

0 1

C 3.26861800 1.22645200 -0.83315700

C 3.17347600 -0.05809500 -0.24546300

C 2.16858600 2.03959200 -0.91446900

C 1.91004100 -0.49196600 0.26316400

C 4.29187200 -0.92472100 -0.14983400

C 0.89643500 1.63576700 -0.41609600

H 2.26330100 3.01559800 -1.37814100

C 1.82387600 -1.78202400 0.85133000

C 0.78202200 0.38038000 0.16644500

C 4.17324300 -2.16670400 0.42187100

H 5.24608900 -0.58570100 -0.53851000

C 2.92567400 -2.59773000 0.92735000

H 0.87175400 -2.11638200 1.24217200

H 5.03503900 -2.82057300 0.48913400

H 2.84098700 -3.57940700 1.37883200

C -0.53617900 -0.08208700 0.69242100

C -1.36900500 -0.93165100 -0.09827700

C -0.96529100 0.32366000 1.93688900

C -2.64612300 -1.32345600 0.41156900

C -0.98345300 -1.38105900 -1.38739600

C -2.22623100 -0.06609000 2.44021900

H -0.31955500 0.95503200 2.53517800

C -3.48099600 -2.14823300 -0.38495300

C -3.05000700 -0.86865600 1.69255900

C -1.81607100 -2.17907200 -2.13267100

H -0.01942200 -1.08169700 -1.77760200

H -2.53712000 0.27461300 3.42092700

C -3.07778300 -2.56737600 -1.62799900

H -4.44921000 -2.44018300 0.00719000

H -4.02128600 -1.16821000 2.07083600

H -1.50645400 -2.51392600 -3.11582700

H -3.72562500 -3.19617800 -2.22757900

H 4.22658000 1.55447500 -1.22282000

Si -0.53324500 2.86849800 -0.57970900

H -0.04590000 3.85382700 -1.58903200

C -2.14797700 2.12766700 -1.19781700

H -1.98998200 1.45913800 -2.04670100

H -2.65705300 1.56108300 -0.41651000

H -2.81331200 2.93450300 -1.52008900

C -0.82552500 3.79615600 1.03535600

H 0.10961800 4.19406200 1.43771000

H -1.50828200 4.63550500 0.87302900

H -1.26994500 3.14093300 1.78748600

**4c'**

0 1

C -0.05523800 2.31728700 1.53889300

H -0.11577600 1.71094000 2.44644200

H 0.85154500 2.92490700 1.60355500

H -0.91174600 2.99748100 1.52557900

C 0.05471000 2.31761600 -1.53849300

H -0.85215700 2.92513700 -1.60290900

H 0.91113700 2.99791100 -1.52511800

H 0.11518900 1.71148200 -2.44618800

Si -0.00012000 1.23296600 0.00008600

C -1.52394800 0.12540200 -0.05109500

C -2.68439500 0.42856200 0.67492000

C -1.53776700 -1.02412000 -0.85670600

C -3.81652800 -0.38022000 0.59912600

H -2.70839200 1.30490300 1.31396400

C -2.66567500 -1.83532000 -0.93802300

H -0.64974700 -1.29573600 -1.41828600

C -3.80905400 -1.51379300 -0.20907400

H -4.70195200 -0.12763600 1.17189300

H -2.65295400 -2.71962000 -1.56544100

H -4.68796800 -2.14572800 -0.26852000

C 1.52396200 0.12576100 0.05111200

C 2.68357500 0.42800200 -0.67660400

C 1.53875800 -1.02265300 0.85829300

C 3.81585100 -0.38060300 -0.60095000

H 2.70679800 1.30346500 -1.31687400

C 2.66680600 -1.83366200 0.93948100

H 0.65138300 -1.29356700 1.42123700

C 3.80935600 -1.51305600 0.20881900

H 4.70062200 -0.12874400 -1.17504200

H 2.65484600 -2.71709400 1.56813400

H 4.68838200 -2.14484400 0.26816700

**4c'-H**

0 1

C 2.40517400 0.30723500 -1.54367500

H 1.98494100 -0.13888900 -2.44849800

H 2.21445400 1.38376800 -1.58344100

H 3.48853700 0.15830700 -1.56190700

C 2.40559900 0.31881100 1.54088400

H 3.48890400 0.16964200 1.56059300

H 2.21519600 1.39567600 1.57219000

H 1.98492500 -0.11999900 2.44908300

Si 1.63915700 -0.44976200 0.00158200

C -0.22329000 -0.18221600 0.00103000

C -1.11808200 -1.26127400 0.00373100

C -0.76065800 1.11463700 -0.00265700

C -2.49639000 -1.05607500 0.00276300

H -0.73368200 -2.27606600 0.00666300

C -2.13597500 1.32643500 -0.00368300

H -0.09855200 1.97556500 -0.00472700

C -3.00744900 0.23878600 -0.00099000

H -3.17030200 -1.90550500 0.00489400

H -2.52921100 2.33691900 -0.00656300

H -4.07931700 0.40104100 -0.00184700

H 1.87233400 -1.92182000 0.00699800

**4c''**

0 1

C 1.21107600 0.68938400 0.00000300

C 0.00839900 1.39361600 -0.00001800

C -1.20262500 0.70401900 0.00001700

C -1.21110900 -0.68948600 -0.00000200

C -0.00845100 -1.39353100 -0.00001200

C 1.20271400 -0.70402400 0.00001200

H 2.15323900 1.22595100 0.00001800

H -2.13829000 1.25184000 0.00003100

H -2.15330600 -1.22584400 0.00000500

H -0.01494400 -2.47774200 -0.00003400

H 2.13827800 -1.25186700 0.00002200

H 0.01500200 2.47779400 -0.00004200

**4a'**

0 1

C -6.11787300 -0.60624400 0.73495400

C -5.04000800 0.22002400 0.93831400

C -3.81822200 0.00261600 0.25225900

C -3.72308100 -1.09480700 -0.65749600

C -4.85294500 -1.92970000 -0.84499800

C -6.02302000 -1.69193000 -0.16594700

H -2.78116700 1.66268200 1.13877800

H -7.04687600 -0.42977400 1.26456800

H -5.10883800 1.05394200 1.62877800

C -2.68624200 0.83720100 0.43985900

C -2.49581700 -1.30927700 -1.33606200

H -4.77775000 -2.76242600 -1.53602700

H -6.88052300 -2.33715500 -0.31805700

C -1.42275300 -0.48212000 -1.12688300

C -1.49752500 0.62211700 -0.22824000

H -2.41878300 -2.14621800 -2.02235600

H -0.49058800 -0.67900100 -1.64639900

Si -0.00000200 1.74037500 -0.00000100

C 0.23741900 2.81868000 -1.52449900

H -0.64778200 3.43946800 -1.68693900

H 1.09796400 3.48538500 -1.41793300

H 0.38674400 2.21053300 -2.42047300

C -0.23744000 2.81870800 1.52447400

H -0.38677400 2.21058100 2.42046000

H 0.64775400 3.43950700 1.68691100

H -1.09798700 3.48540500 1.41787900

C 2.68623100 0.83719100 -0.43988000

C 3.81821600 0.00261300 -0.25227600

C 1.49752900 0.62213100 0.22825300

C 3.72309500 -1.09477800 0.65752100

C 5.03998600 0.21999600 -0.93836500

C 1.42277700 -0.48207400 1.12693700

C 4.85296300 -1.92966400 0.84502700

C 2.49584600 -1.30922300 1.33612100

C 6.11785600 -0.60626400 -0.73500000

H 5.10880100 1.05389000 -1.62886100

H 0.49062400 -0.67893700 1.64648100

C 6.02302300 -1.69191800 0.16594200

H 4.77778300 -2.76236600 1.53608700

H 2.41882700 -2.14614000 2.02244700

H 7.04684700 -0.42981400 -1.26464100

H 6.88052900 -2.33713800 0.31805600

H 2.78114000 1.66264800 -1.13883000

**4a'-H**

0 1

Si -2.78612200 -0.56189300 -0.00001500

C -3.65059000 0.08842500 1.54197700

H -3.61173200 1.18115800 1.57787700

H -4.70285300 -0.20934500 1.56020400

H -3.17343900 -0.29174500 2.44868400

C -3.65044200 0.08867000 -1.54199200

H -3.17321700 -0.29142100 -2.44869500

H -4.70276500 -0.20887000 -1.56045000

H -3.61134000 1.18140600 -1.57770000

C 0.03921900 -0.96708900 -0.00003400

C 1.40598000 -0.58674900 -0.00002400

C -0.98104000 -0.03671900 -0.00001000

C 1.73557000 0.80340700 0.00003100

C 2.45596100 -1.53974000 -0.00005700

C -0.62949400 1.34479100 0.00003300

C 3.10060400 1.18472600 0.00006200

C 0.68004500 1.75111500 0.00005700

C 3.76946700 -1.13912800 -0.00002900

H 2.20240400 -2.59443000 -0.00010900

H -1.41356300 2.09580900 0.00004400

C 4.09472200 0.23692700 0.00003400

H 3.34786600 2.24088200 0.00010600

H 0.92786200 2.80751300 0.00009800

H 4.56358600 -1.87670100 -0.00005500

H 5.13517500 0.54056000 0.00006100

H -2.81016400 -2.05186100 -0.00007800

H -0.19212400 -2.02826800 -0.00006100

**4a''**

0 1

C 2.42754400 0.70714400 0.00000200

C 1.24222200 1.40025100 -0.00000300

C 0.00001400 0.71538300 -0.00000100

C -0.00001600 -0.71537700 -0.00000100

C 1.24220100 -1.40025500 -0.00000300

C 2.42752800 -0.70716500 0.00000200

H -1.23955100 2.48511100 -0.00000600

H 3.37027300 1.24219600 0.00000500

H 1.23958700 2.48509900 -0.00000100

C -1.24220600 1.40026200 0.00000000

C -1.24222500 -1.40025800 0.00000000

H 1.23955800 -2.48510400 0.00000000

H 3.37025300 -1.24222400 0.00000500

C -2.42754000 -0.70714800 0.00000000

C -2.42752500 0.70716200 0.00000300

H -1.23957500 -2.48510400 -0.00000400

H -3.37028200 -1.24218000 0.00000300

H -3.37025400 1.24221300 0.00000200

**TS-rs**

0 1

C 2.52771200 -3.24656600 0.41079200

C 1.58906000 -2.26010000 0.59495800

C 1.75000000 -0.92692500 0.13631500

C 3.06907200 -0.63300300 -0.38021900

C 4.00370900 -1.67770500 -0.60822500

C 3.73878600 -2.97189100 -0.25049200

H 2.33584000 -4.23903200 0.80205700

H 0.73846200 -2.51259300 1.18750200

C 0.77623900 0.15330000 0.20564700

C 3.48650000 0.69773500 -0.59329300

H 4.96024700 -1.41145300 -1.04446700

H 4.46528900 -3.75716200 -0.42208900

C 2.63829300 1.72085900 -0.27498400

C 1.30460600 1.45194300 0.07841300

H 4.48752200 0.88348100 -0.96620700

H 2.96894200 2.75078700 -0.36398000

C -0.77623900 0.15330000 0.20564800

C -1.75000000 -0.92692500 0.13631500

C -1.30460600 1.45194300 0.07841500

C -3.06907200 -0.63300300 -0.38021800

C -1.58906000 -2.26010100 0.59495800

C -2.63829300 1.72085900 -0.27498200

C -4.00370900 -1.67770500 -0.60822500

C -3.48650000 0.69773500 -0.59329100

C -2.52771200 -3.24656600 0.41079200

H -0.73846200 -2.51259400 1.18750200

H -2.96894200 2.75078700 -0.36397800

C -3.73878600 -2.97189100 -0.25049200

H -4.96024700 -1.41145200 -1.04446700

H -4.48752200 0.88348200 -0.96620600

H -2.33584000 -4.23903300 0.80205600

H -4.46528800 -3.75716100 -0.42209000

Si 0.00000000 2.76216600 0.22880100

C -0.00000200 4.00965300 -1.17615100

H -0.88188500 4.65540200 -1.12927900

H 0.88188500 4.65539800 -1.12928500

H -0.00000600 3.50210200 -2.14326600

C 0.00000200 3.64348100 1.89049500

H 0.88385600 4.27929400 1.99586600

H -0.88385100 4.27929500 1.99586800

H 0.00000300 2.91956500 2.70819800

(*S*)-**4a**

0 1

C -2.16924100 3.09792700 -0.98642500

C -1.26722400 2.08118700 -0.79347200

C -1.63547300 0.88088600 -0.12606300

C -3.01366300 0.72271300 0.24432300

C -3.90808800 1.81106700 0.07049000

C -3.49723500 2.97845300 -0.51988900

H -1.86207100 3.99562500 -1.51030100

H -0.26357300 2.18344400 -1.17813100

C -0.74293300 -0.21928800 0.09157000

C -3.48009800 -0.53208600 0.70533200

H -4.93816500 1.68701700 0.38672600

H -4.19461500 3.79625400 -0.65951800

C -2.64286200 -1.62117200 0.71270900

C -1.27779200 -1.47778700 0.39418300

H -4.52267500 -0.62936500 0.98835400

H -3.03510400 -2.59905800 0.97288000

C 0.74346600 -0.21723200 -0.09339300

C 1.63289900 0.88544700 0.12459800

C 1.28188300 -1.47451400 -0.39474400

C 3.01223400 0.73015800 -0.24280600

C 1.26046100 2.08564400 0.78988700

C 2.64775500 -1.61471100 -0.71123200

C 3.90396000 1.82046500 -0.06738400

C 3.48245200 -0.52370300 -0.70251500

C 2.15985400 3.10439100 0.98440400

H 0.25504300 2.18664400 1.17024000

H 3.04275200 -2.59174000 -0.97045900

C 3.48932600 2.98725200 0.52152900

H 4.93501200 1.69843200 -0.38122100

H 4.52582000 -0.61867700 -0.98340000

H 1.84934700 4.00200900 1.50644900

H 4.18469700 3.80654000 0.66246100

Si 0.00372200 -2.78978400 -0.00031100

C -0.55163400 -3.86444400 -1.43955400

H 0.25999600 -4.51539300 -1.77769700

H -1.39175200 -4.50294100 -1.15067700

H -0.86843500 -3.24672900 -2.28276500

C 0.56181000 -3.86310900 1.43886400

H -0.24813100 -4.51621400 1.77690900

H 1.40355000 -4.49942500 1.14987500

H 0.87704700 -3.24472600 2.28216300
